# Supplementary material for: Recommendations on Complementary Feeding as a Tool for Prevention of Non-Communicable Diseases (NCDs)—Paper Co-Drafted by the SIPPS, FIMP, SIDOHaD, and SINUPE Joint Working Group
Source: Nutrients. 2022 Jan 7;14(2):257. doi: 10.3390/nu14020257 (PMC8778394; doi:10.3390/nu14020257)
Supplement: Supplementary file 1 [file nutrients-14-00257-s001.zip › nutrients-1513088-supplementary/Supplementary File S1.pdf]

APPENDIX 1

Recommendations 1-7

A1.a. Macro and micronutrient requirements from 6 to 24 months.

Key Questions

- ***Does an energy intake above age-recommended levels from 6 to 24 months of age result in different short- and long-term nutritional and metabolic outcomes compared to an intake that meets recommended levels?***

- ***Does a carbohydrate intake above age-recommended levels from 6 to 24 months of age result in different short- and long-term nutritional and metabolic outcomes compared to an intake that meets recommended levels?***

- ***Does a protein intake above age-recommended levels from 6 to 24 months of age result in different short- and long-term nutritional and metabolic outcomes compared to an intake that meets recommended levels?***

- ***Does a lipid intake above age-recommended levels from 6 to 24 months of age result in different short- and long-term nutritional and metabolic outcomes compared to an intake that meets recommended levels?***

a.
P In the healthy infant, in the period 6-24 months,
I an energy intake above the recommended levels for age
C compared with an intake corresponding to recommended levels
O results in different short- and long-term nutritional and metabolic outcomes?

b.
P In the healthy infant, in the period 6-24 months,
I a carbohydrate intake above the recommended levels for age
C compared with an intake corresponding to recommended levels
O results in different short- and long-term nutritional and metabolic outcomes?

c.
P In the healthy infant, in the period 6-24 months,
I a protein intake above the recommended levels for age
C compared with an intake corresponding to recommended levels
O results in different short- and long-term nutritional and metabolic outcomes?

d
P In the healthy infant, in the period 6-24 months,
I a lipid intake above the recommended levels for age
C compared with an intake corresponding to recommended levels
O results in different short- and long-term nutritional and metabolic outcomes?

KEYWORDS

Population

A. Infant
B. Child
C. Adolescent
D. Young adult

Exposure Factors / Comparison

MeSH Terms/ Text word: weaning; wean*; diet; food; beverages; infant nutritional physiological phenomena; meals; food and beverages; infant food; eating; diet; diets; supplementary feeding Solid food

A. Complementary feeding
B. "Proteins" OR proteins[Text Word]
C. "lipids" OR lipids[Text Word]
D. "sugars" OR sugars[Text Word]
E. "carbohydrates" OR Carbohydrates[Text Word]
F. "Dietary Carbohydrates"[Mesh]
G. "dietary fats" OR dietary fats[Text Word]
H. "dietary proteins" OR dietary proteins[Text Word]
I. "Energy Intake"[Mesh]

Outcomes

A. Overnutrition
B. Obesity
C. Growth
D. Body Size
E. Body Height
F. Diabetes Mellitus (diabetic patient)
G. Noncommunicable Diseases
H. Nutritional and Metabolic Diseases (metabolic disorder)
I. Pediatric Obesity (childhood obesity)
J. Overweight
K. Body Mass Index (Z-Score)
L. Body Weight Changes
M. Body Weight
N. Body Composition
O. Nutritional Status
P. Growth and Development ('growth, development and aging')
Q. Fat body (Fat pad)
R. Adipose tissue
S. Body fat
T. Adiposity rebound

Guidelines Research

Temporal limitation: 2015-2021.

PUBMED https://www.ncbi.nlm.nih.gov/pubmed/

#1

(Complementary OR supplementary OR wean* OR transition* OR introduc* OR "Infant Nutritional Physiological Phenomena" OR weaning OR (Solid food*) OR solids OR "infant food" OR infant feed*) AND ("Proteins" OR proteins OR "lipids" OR lipids OR "sugars" OR sugars OR "carbohydrates" OR Carbohydrates OR "Dietary Carbohydrates" OR "dietary fats" OR dietary fats OR "dietary proteins" OR dietary proteins OR "Energy Intake"[Mesh]) AND ("Obesity" OR "Pediatric Obesity" OR "Overweight" OR "Body Mass Index" OR "Body Weight Changes" OR "Body Weight" OR "Body Composition" OR "Nutritional Status" OR "Growth and Development" OR "Growth" OR "fat body" OR "adipose tissue" OR body fat OR "adiposity rebound" OR "Diabetes Mellitus" OR "Nutritional and Metabolic Diseases" OR "Noncommunicable Diseases"[Mesh]) AND "2015/03/07"[PDat]:"2021/03/14"[PDat] AND ("infant" OR "Child" OR "Adolescent" OR "Young Adult"[Mesh])

Filters applied: Guideline, Practice Guideline, in the last 5 years. Clear all

EMBASE https://www.embase.com

#1

Query

('overnutrition'/exp OR 'growth, development and aging'/exp OR 'obesity'/exp OR 'childhood obesity'/exp OR 'body size'/exp OR 'body weight'/exp OR 'body height'/exp OR 'body mass'/exp OR 'body mass index z score'/exp OR 'body weight change'/exp OR 'body composition'/exp OR 'nutritional status'/exp OR 'adipose tissue'/exp OR 'adiposity rebound'/exp OR 'diabetic patient'/exp OR 'non communicable disease'/exp OR 'metabolic disorder'/exp) AND ('weaning'/exp OR 'wean*' OR 'complementary feeding'/exp OR 'supplementary feeding'/exp OR 'solid food'/exp OR 'dietary intake'/exp OR 'caloric intake'/exp OR 'protein intake'/exp OR 'amino acid intake'/exp OR 'carbohydrate intake'/exp OR 'sugar intake'/exp OR 'fat intake'/exp OR 'lipid intake') AND ([adolescent]/lim OR [child]/lim OR [infant]/lim OR [preschool]/lim OR [school]/lim OR [young adult]/lim) AND 'practice guideline'/de AND [2015-2021]/py

UPTODATE https://www.uptodate.com/home

Society Guideline Links: *complementary feeding*

SOCIETY GUIDELINE LINKS: ***Complementary feeding, Weaning, Alimentary – nutrition,***

National Guideline Clearinghouse (NGC) https://www.ahrq.gov/gam/index.html

Canadians Medical Association (CMA) https://www.cma.ca/clinicalresources/practiceguidelines

National Guideline Centre (NGC) - National Institute of Health and Care Excellence (NICE) https://www.rcplondon.ac.uk/about-us/what-we-do/national-guideline-centre-ngc

Scottish Intercollegiate Guidelines Network (SIGN) https://www.sign.ac.uk/our-guidelines.html

Australian Clinical Practice Guidelines (ACPG) https://www.clinicalguidelines.gov.au/

New Zealand Guidelines Group (NZGG) https://www.health.govt.nz/about-ministry/ministry-health-websites/new-zealand-guidelines-group

American Academy of Pediatrics (AAP) https://www.aap.org/en-us/Pages/Default.aspx
DateRange (01/01/2013-03/19/2019) AND ((complementary feeding) OR (weaning)) AND (Guideline)
North American Society for Pediatric Gastroenterology, Hepatology and Nutrition (NASPGHAN) https://www.naspghan.org/

European Society for Paediatric Gastroenterology Hepatology and Nutrition (ESPGHAN) http://www.espghan.org/

Geneva Foundation for Medical Education and Research (GFMER) https://www.gfmer.ch/Guidelines/Allattamento_it/Allattamento_alimentazione_complementare.htm

Società Italiana di Nutrizione Umana (SINU) http://www.sinu.it

Società Italiana di Pediatria (SIP) http://www-sip.it/

Società Italiana di Pediatria Preventiva e Sociale (SIPPS) https://www.sipps.it/

Società Italiana di Nutrizione Pediatrica (SINUPE) https://www.sip.it/2017/09/21/sinupe-societa-italiana-di-nutrizione-pediatrica/

Società Italiana di Endocrinologia e Diabetologia Pediatrica (SIEDP) http://www.siedp.it/pagina/84/linee+guida%2C+raccomandazioni+e+consensus

Sysitematic Review Research

Temporal Limitation: 2011/15-2021

PUBMED

#1

systematic[sb] AND (((((("Infant Nutritional Physiological Phenomena"[Mesh]) AND "Overnutrition"[Mesh]) OR "Growth"[Mesh]) OR "Diabetes Mellitus"[Mesh]) OR "Nutritional and Metabolic Diseases"[Mesh]) OR "Noncommunicable Diseases"[Mesh])

Filters applied: Meta-Analysis, Systematic Reviews, in the last 5 years, Child: birth-18 years.

#2

(Complementary OR supplementary OR wean* OR transition* OR introduc* OR "Infant Nutritional Physiological Phenomena" OR weaning OR (Solid food*) OR solids OR “infant food” OR infant feed*) AND ("Proteins" OR proteins OR "lipids" OR lipids OR "sugars" OR sugars OR "carbohydrates" OR Carbohydrates OR "Dietary Carbohydrates" OR "dietary fats" OR dietary fats OR "dietary proteins" OR dietary proteins OR "Energy Intake"[Mesh]) AND ("Obesity" OR "Pediatric Obesity" OR "Overweight" OR "Body Mass Index" OR "Body Weight Changes" OR "Body Weight" OR "Body Composition" OR "Nutritional Status" OR "Growth and Development" OR "Growth" OR "fat body" OR "adipose tissue" OR body fat OR “adiposity rebound” OR "Diabetes

Mellitus" OR "Nutritional and Metabolic Diseases" OR "Noncommunicable Diseases"[Mesh]) AND "2015/03/07"[PDat]:"2021/03/14"[PDat] AND ("infant" OR "Child" OR "Adolescent" OR "Young Adult"[Mesh])

Filters activated: Meta-Analysis, Systematic Reviews, published in the last 5 years

#3

(Complementary OR supplementary OR wean* OR transition* OR introduc* OR "Infant Nutritional Physiological Phenomena"[MeSH Terms] OR weaning OR ((Solid food*) OR solids)) OR “infant food” OR infant feed*) AND ("Proteins" OR proteins OR "lipids" OR lipids OR "sugars" OR sugars OR "carbohydrates" OR Carbohydrates OR "Dietary Carbohydrates" OR "dietary fats" OR dietary fats OR "dietary proteins" OR dietary proteins OR "Energy Intake"[MeSH Terms]) AND ((((((((((((("Obesity" OR "Pediatric Obesity"[MeSH Terms]) OR "Overweight"[MeSH Terms]) OR "Body Mass Index"[MeSH Terms]) OR "Body Weight Changes"[MeSH Terms]) OR "Body Weight"[MeSH Terms]) OR "Body Composition"[MeSH Terms]) OR "Nutritional Status"[MeSH Terms]) OR "Growth and Development"[MeSH Terms]) OR "Growth"[MeSH Terms]) OR "fat body"[MeSH Terms]) OR "adipose tissue"[MeSH Terms]) OR body fat[Text Word]) OR “adiposity rebound” OR "Diabetes Mellitus"[MeSH Terms]) OR "Nutritional and Metabolic Diseases"[MeSH Terms]) OR "Noncommunicable Diseases"[MeSH Terms]) AND "2015/06/13"[PDat]: "2021/03/14"[PDat] AND ("infant" OR "Child" OR "Adolescent" OR "Young Adult"[Mesh])

Filters activated: Systematic Reviews, Meta-Analysis, published in the last 5 years

EMBASE

#1

Query

('overnutrition'/exp OR 'growth, development and aging'/exp OR 'obesity'/exp OR 'childhood obesity'/exp OR 'body size'/exp OR 'body weight'/exp OR 'body height'/exp OR 'body mass'/exp OR 'body mass index z score'/exp OR 'body weight change'/exp OR 'body composition'/exp OR 'nutritional status'/exp OR 'adipose tissue'/exp OR 'adiposity rebound'/exp OR 'diabetic patient'/exp OR 'non communicable disease'/exp OR 'metabolic disorder'/exp) AND ('weaning'/exp OR 'wean*' OR 'complementary feeding'/exp OR 'supplementary feeding'/exp OR 'solid food'/exp OR 'dietary intake'/exp OR 'caloric intake'/exp OR 'protein intake'/exp OR 'amino acid intake'/exp OR 'carbohydrate intake'/exp OR 'sugar intake'/exp OR 'fat intake'/exp OR 'lipid intake') AND ([adolescent]/lim OR [child]/lim OR [infant]/lim OR [preschool]/lim OR [school]/lim OR [young adult]/lim) AND ('meta analysis'/de OR 'systematic review'/de) AND [2015-2021]/py

COCHRANE LIBRARY

#1

MeSH descriptor - Infant Nutritional Physiological Phenomena- Infant Nutritional Physiological Phenomena in Title Abstract Keyword - with Cochrane Library publication date Between Jan 2010 and March 2021, in Cochrane Reviews (Word variations have been searched)

#2

Weaning OR "Complementary feeding" in Title Abstract Keyword - with Cochrane Library publication date Between Jan 2010 and March 2021, in Cochrane Reviews (Word variations have been searched)

#3

(Infant Nutritional Physiological Phenomena OR complementary feeding) AND ("Proteins" OR proteins OR "lipids" OR lipids OR "sugars" OR sugars OR "carbohydrates" OR Carbohydrates OR "Dietary Carbohydrates" OR "dietary fats" OR dietary fats OR "dietary proteins" OR dietary proteins OR "Energy Intake”) AND ("Obesity" OR "Pediatric Obesity" OR "Overweight" OR "Body Mass Index" OR "Body Weight Changes" OR "Body Weight" OR "Body Composition" OR "Nutritional Status" OR "Growth and Development" OR "Growth" OR "fat body" OR "adipose tissue" OR body fat OR “adiposity rebound” OR "Diabetes Mellitus" OR "Nutritional and Metabolic Diseases" OR "Noncommunicable Diseases”)" with Publication Year from 2012 to 2021, with Cochrane Library publication date Between Jan 2012 and March 2021, in Trials (Word variations have been searched)

Studies Research

PUBMED

#1

((((((("Infant Nutritional Physiological Phenomena"[Mesh]) AND "Overnutrition"[Mesh]) OR "Growth"[Mesh]) OR "Diabetes Mellitus"[Mesh]) OR "Nutritional and Metabolic Diseases"[Mesh]) OR "Noncommunicable Diseases"[Mesh])

Filters applied: Clinical Trial, Multicenter Study, Observational Study, Randomized Controlled Trial, in the last 5 years, Infant: birth-23 months

#2

(Complementary OR supplementary OR wean* OR transition* OR introduc* OR "Infant Nutritional Physiological Phenomena" OR weaning OR (Solid food*) OR solids OR “infant food” OR infant feed*) AND ("Proteins" OR proteins OR "lipids" OR lipids OR "sugars" OR sugars OR "carbohydrates" OR Carbohydrates OR "Dietary Carbohydrates" OR "dietary fats" OR dietary fats OR "dietary proteins" OR dietary proteins OR "Energy Intake"[Mesh]) AND ("Obesity" OR "Pediatric Obesity" OR "Overweight" OR "Body Mass Index" OR "Body Weight Changes" OR "Body Weight" OR "Body Composition" OR "Nutritional Status" OR "Growth and Development" OR "Growth" OR "fat body" OR "adipose tissue" OR body fat OR “adiposity rebound” OR "Diabetes Mellitus" OR "Nutritional and Metabolic Diseases" OR "Noncommunicable Diseases"[Mesh]) AND

"2015/03/07"[PDat]:"2021/03/15"[PDat] AND ("infant" OR "Child" OR "Adolescent" OR "Young Adult"[Mesh])
Filters applied: Clinical Trial, Multicenter Study, Observational Study, Randomized Controlled Trial, from 2013/3/7 - 2021/3/15.

#3

(Complementary OR supplementary OR wean* OR transition* OR introduc* OR "Infant Nutritional Physiological Phenomena"[MeSH Terms] OR weaning OR ((Solid food*) OR solids)) OR “infant food” OR infant feed*) AND ("Proteins" OR proteins OR "lipids" OR lipids OR "sugars" OR sugars OR "carbohydrates" OR Carbohydrates OR "Dietary Carbohydrates" OR "dietary fats" OR dietary fats OR "dietary proteins" OR dietary proteins OR "Energy Intake"[MeSH Terms]) AND ((((((((((((("Obesity" OR "Pediatric Obesity"[MeSH Terms]) OR "Overweight"[MeSH Terms]) OR "Body Mass Index"[MeSH Terms]) OR "Body Weight Changes"[MeSH Terms]) OR "Body Weight"[MeSH Terms]) OR "Body Composition"[MeSH Terms]) OR "Nutritional Status"[MeSH Terms]) OR "Growth and Development"[MeSH Terms]) OR "Growth"[MeSH Terms]) OR "fat body"[MeSH Terms]) OR "adipose tissue"[MeSH Terms]) OR body fat[Text Word]) OR “adiposity rebound” OR "Diabetes Mellitus"[MeSH Terms]) OR "Nutritional and Metabolic Diseases"[MeSH Terms]) OR "Noncommunicable Diseases"[MeSH Terms]) AND "2013/03/13"[PDat]: "2021/03/15"[PDat] AND ("infant" OR "Child" OR "Adolescent" OR "Young Adult"[Mesh])

Filters applied: Clinical Trial, Multicenter Study, Observational Study, Randomized Controlled Trial, Infant: birth-23 months, from 2013/3/7 - 2021/3/15.

EMBASE

('overnutrition'/exp OR 'growth, development and aging'/exp OR 'obesity'/exp OR 'childhood obesity'/exp OR 'body size'/exp OR 'body weight'/exp OR 'body height'/exp OR 'body mass'/exp OR 'body mass index z score'/exp OR 'body weight change'/exp OR 'body composition'/exp OR 'nutritional status'/exp OR 'adipose tissue'/exp OR 'adiposity rebound'/exp OR 'diabetic patient'/exp OR 'non communicable disease'/exp OR 'metabolic disorder'/exp) AND ('weaning'/exp OR 'wean*' OR 'complementary feeding'/exp OR 'supplementary feeding'/exp OR 'solid food'/exp OR 'dietary intake'/exp OR 'caloric intake'/exp OR 'protein intake'/exp OR 'amino acid intake'/exp OR 'carbohydrate intake'/exp OR 'sugar intake'/exp OR 'fat intake'/exp OR 'lipid intake') AND [2010-2021]/py AND ('case control study'/de OR 'cohort analysis'/de OR 'controlled study'/de OR 'cross sectional study'/de OR 'intervention study'/de OR 'major clinical study'/de OR 'observational study'/de OR 'prospective study'/de OR 'randomized controlled trial'/de) AND ('article'/it OR 'article in press'/it) AND ('body composition analyzer'/dv OR 'body weight scale'/dv OR 'growth chart'/dv OR 'sphygmomanometer'/dv OR 'x ray bone densitometer'/dv) AND ('clinical trial'/lnk OR 'clinicaltrials gov'/lnk OR 'disease management'/lnk OR 'epidemiology'/lnk OR 'etiology'/lnk OR 'prevention'/lnk OR 'therapy'/lnk) AND ([adolescent]/lim OR [child]/lim OR [infant]/lim OR [preschool]/lim OR [school]/lim OR [young adult]/lim)

COCHRANE LIBRARY

#1

535 Trials matching "#1 - (Infant Nutritional Physiological Phenomena OR complementary feeding) AND ("Proteins" OR proteins OR "lipids" OR lipids OR "sugars" OR sugars OR "carbohydrates" OR

Carbohydrates OR "Dietary Carbohydrates" OR "dietary fats" OR dietary fats OR "dietary proteins" OR dietary proteins OR "Energy Intake”) AND ("Obesity" OR "Pediatric Obesity" OR "Overweight" OR "Body Mass Index" OR "Body Weight Changes" OR "Body Weight" OR "Body Composition" OR "Nutritional Status" OR "Growth and Development" OR "Growth" OR "fat body" OR "adipose tissue" OR body fat OR “adiposity rebound” OR "Diabetes Mellitus" OR "Nutritional and Metabolic Diseases" OR "Noncommunicable Diseases”)" with Publication Year from 2012 to 2021, with Cochrane Library publication date Between Jan 2012 and March 2021, in Trials (Word variations have been searched)

Figure a1.1. Guidelines search flow diagram.

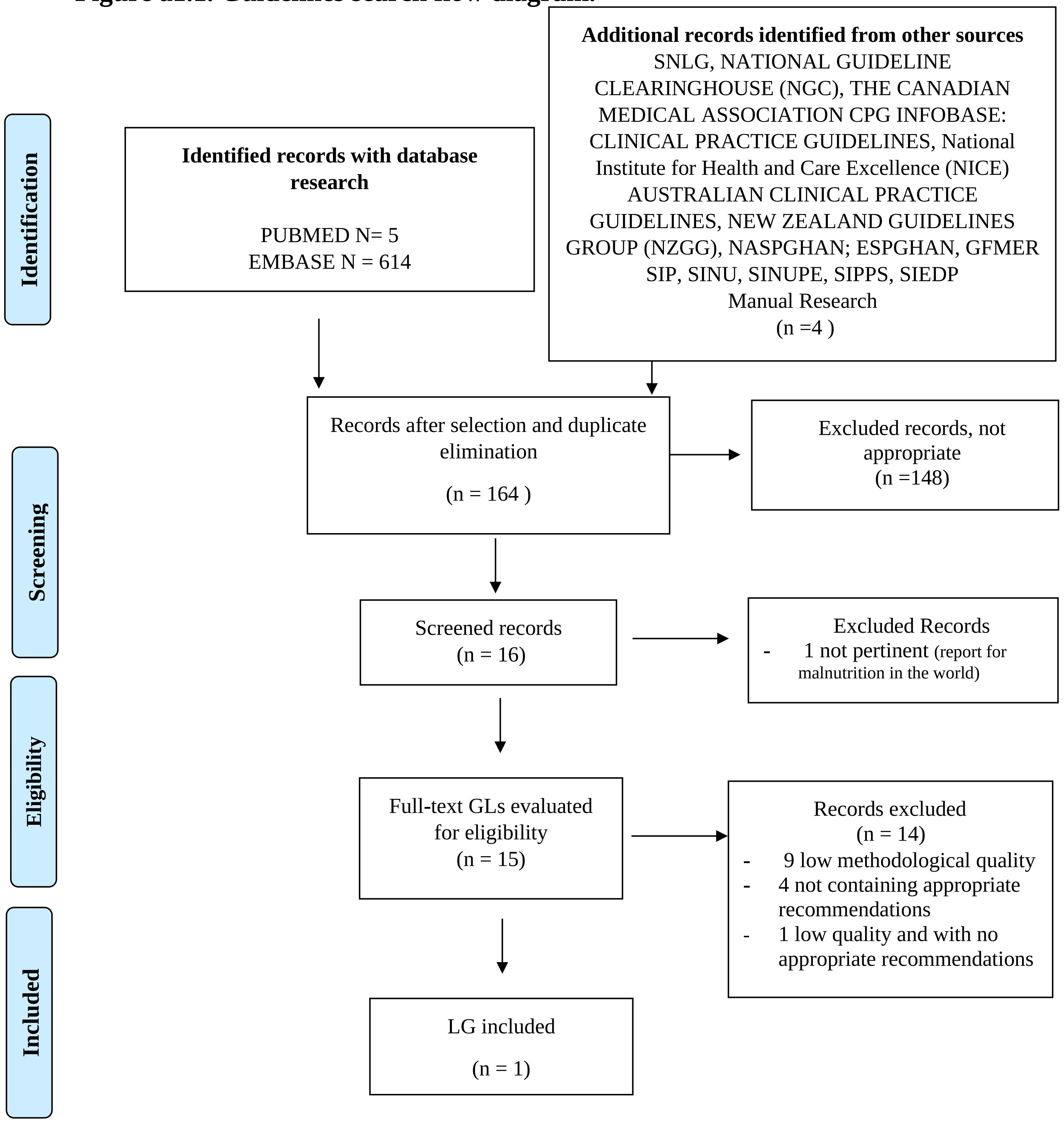

Figure a1.2. SRs search flow diagram

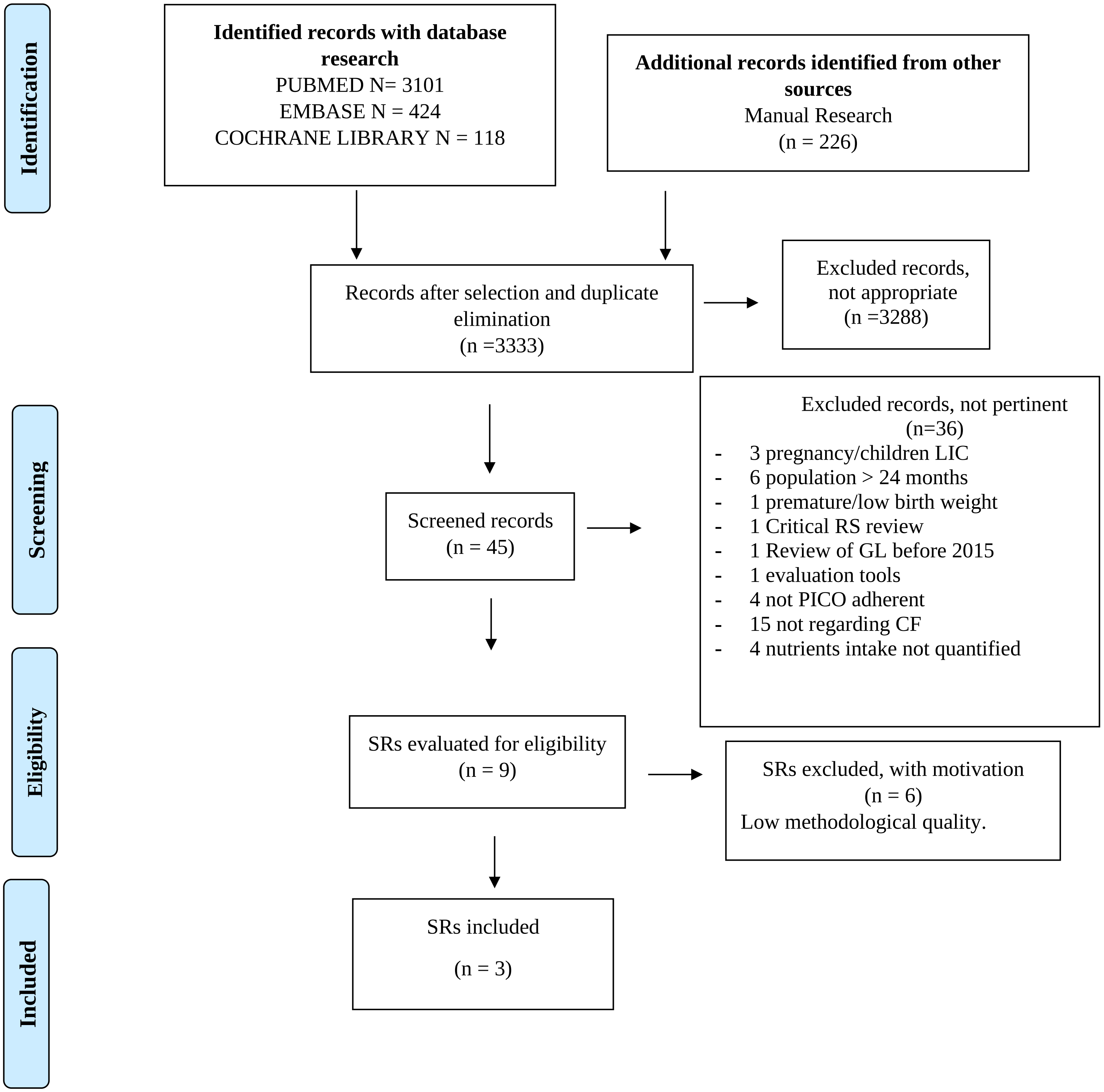

Figure a1.3. Studies search flow diagram.

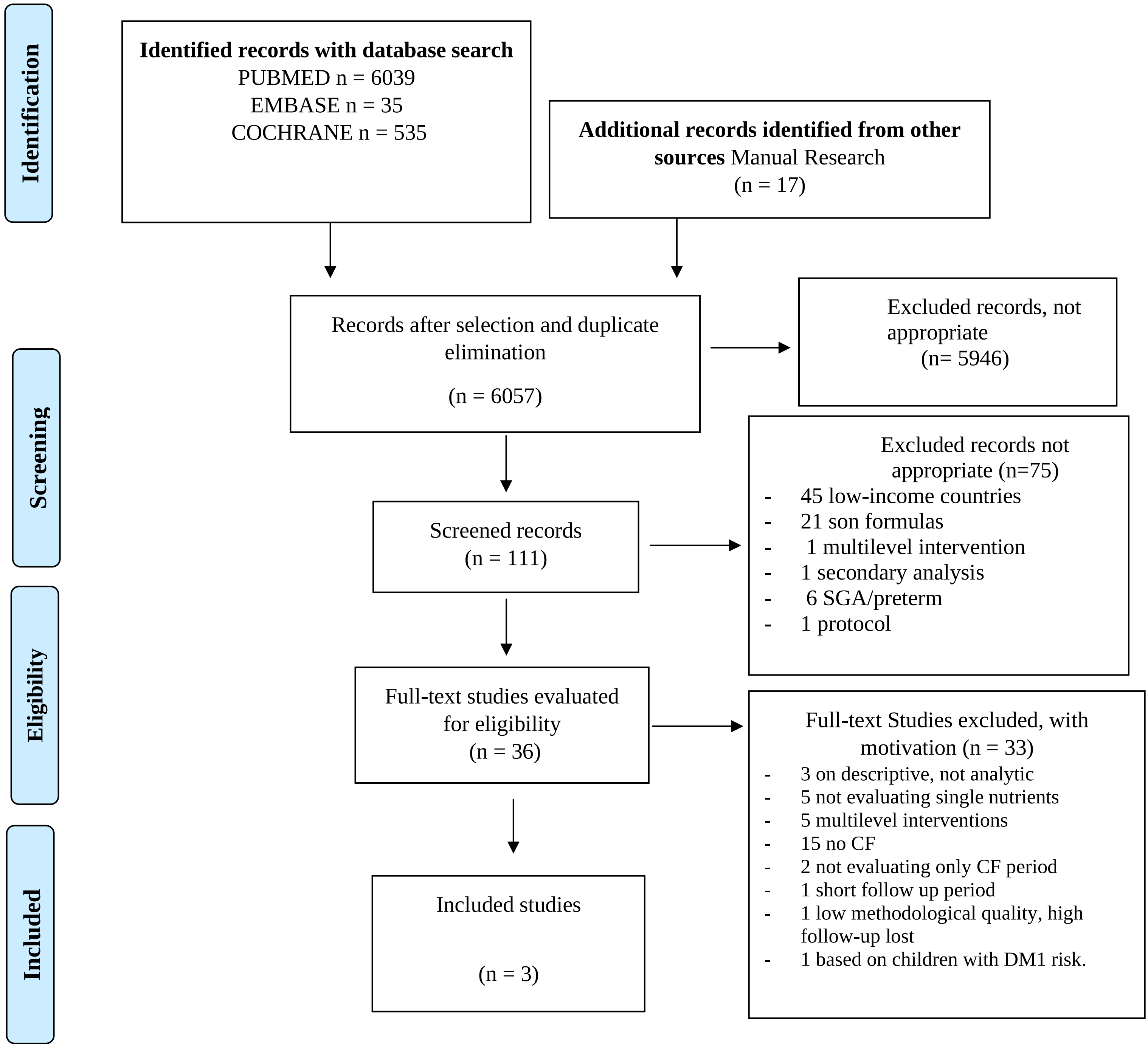

A1.a. METHODOLOGICAL EVALUATION

Table a1.1. Appraisal of the Clinical Guidelines and Documents

Guidelines and Clinical Documents	Methodological Evaluation			
	**Multidisciplinary panel**	**Systematic evidence research**	**Grading of recommendations**	**GL overall assessment**
SIEDP-SIP 2018 [1]	Yes	No, only MEDLINE	Yes	Moderate methodological quality

Table a1.2. Clinical Guidelines and Documents excluded.

GL Excluded	Multidisciplinary panel	Systematic evidence research	Grading of recommendations	Reason for exclusion
Alvisi et al. 2015 [2]	Limited to Pediatricians and Nutritionists.	No	No	Review document, with recommendations for clinical guidance.<br>Low methodological quality.
Canada's Dietary Guidelines 2018 [3]	Limited to Nutritionists and Public Health Experts.	No	No	Low methodological quality.<br>No relevant recommendations.
Dereń et al. 2019. EAP ECOG [4]	No	No	No	Low methodological quality.
EFSA 2013 Scientific opinion [5]	No	No	No	Low methodological quality regarding response to questions.
Fewtrell et al. 2017. ESPGHAN Complementary feeding [6]	No	Declared but not published	No	Low methodological quality.
Fidler Mis ESPGHAN 2017 Sugar in Infants, Children, and Adolescents [7]	No	Declared but not published	No	Low methodological quality.
WHO 2015 Sugar intake [8]	Yes	Yes	Yes	No appropriate Recommendations.

Heyman et al. 2017 AAP - Fruit Juice [9]	No	No	No	Low methodological quality.
Kastorini et al. 2019 – Greece GL [10]	Yes	Declared but not published	Declared but not published	Low methodological quality.<br>No appropriate recommendations.
Koletzko et al. 2019 The Early Nutrition Project Recommendations [11]	Yes	Yes but not explicated (Previously Published RS used: For the Questions in this Consensus Patro-Golab Et Al. 2016)	No Consent Vote	Low methodological quality.
NICE 2015 Preventing excess weight gain [12]	===	====	===	Interventions Following the CF Period.
Romero-Velardea et al. 2016. Alimentation complementaria [13]	Limited to Pediatrician and nutrition Experts	No	No	Low methodological quality.
Schwarzenberg et al. 2018. AAP Policy Statement [14]	No	No	No	Low methodological quality.
USDA 2015-2020 [15]	Yes	Yes	Related to quality of Evidence	No appropriate recommendations.

Table a1.3. Appraisal of the Systematic Reviews

**AMSTAR 2**	Hörnell et al. 2013 [16]	Pearce et al. 2013 [17]	Ferrè et al. 2021 [18]
**1. Did the research questions and inclusion criteria for the review include the components of PICO?**<br><br>**(Yes/No)**	Yes	Yes	Yes
**2. Did the report of the review contain an explicit statement that the review methods were established before the conduct of the review and did the report justify any significant deviations from the protocol?**<br><br>**(Yes/Partial Yes/No)**	Yes	No	Partial Yes
**3. Did the review authors explain their selection of the study designs for inclusion in the review?**<br><br>**(Yes/No)**	Yes	Yes	Yes
**4. Did the review authors use a comprehensive literature search strategy?**<br><br>**(Yes/Partial Yes/No)**	Partial Yes	Partial Yes	Partial Yes
**5. Did the review authors perform study selection in duplicate?**<br><br>**(Yes/No)**	Yes	Yes	Yes
**6. Did the review authors perform data extraction in duplicate?**<br><br>**(Yes/No)**	Yes	Yes	Yes
**7. Did the review authors provide a list of excluded studies and justify the exclusions?**<br><br>**(Yes/Partial Yes/No)**	Yes	No	Yes
**8. Did the review authors describe the included studies in adequate detail?**	Yes	Partial Yes	Partial Yes

**(Yes/Partial Yes/No)**			
**9. Did the review authors use a satisfactory technique for assessing the risk of bias (RoB) in individual studies that were included in the review?**<br><br>**(Yes/Partial Yes/No/Includes only NRSI-RCT)**	RCT - Partial Yes<br><br>NRSI - Yes	NRSI - Yes	RCT - Partial Yes<br><br>NRSI - Partial Yes
**10. Did the review authors report on the sources of funding for the studies included in the review?**<br><br>**(Yes/No)**	No	No	No
**11. If meta-analysis was performed did the review authors use appropriate methods for statistical combination of results?**<br><br>**(Yes / No / No meta-analysis conducted)**	No meta-analysis conducted.	No meta-analysis conducted.	No meta-analysis conducted.
**12. If meta-analysis was performed, did the review authors assess the potential impact of RoB in individual studies on the results of the meta-analysis or other evidence synthesis?**<br><br>**(Yes / No / No meta-analysis conducted)**	No meta-analysis conducted	No meta-analysis conducted	No meta-analysis conducted
**13. Did the review authors account for RoB in individual studies when interpreting/ discussing the results of the review?**<br><br>**(Yes/No)**	Yes	Yes	Yes
**14. Did the review authors provide a satisfactory explanation for, and discussion of, any heterogeneity observed in the results of the review?**<br><br>**(Yes/No)**	Yes	Yes	Yes
**15. If they performed quantitative synthesis did the review authors carry out an adequate investigation of publication bias (small study bias) and discuss its likely impact on the results of the review?**<br><br>**(Yes / No / No meta-analysis conducted)**	No meta-analysis conducted	No meta-analysis conducted	No meta-analysis conducted

**16. Did the review authors report any potential sources of conflict of interest, including any funding they received for conducting the review?**<br><br>**(Yes/No)**	Yes	Yes	Yes
**OVERALL EVALUATION**	**Moderate quality.**	**Moderate quality.**	**Moderate quality.**

Table a1.4. Appraisal of the SR's Overview

COCHRANE TOOL FOR REVIEW OVERVIEWS.		Patro-Gołąb et al. 2016 [19]
Objectives	To summarize evidence from systematic reviews of the effects of interventions.	Appropriate
Selection Criteria	Describe inclusion and exclusion criteria for reviews.	Appropriate
Research	Typically search for only relevant (Cochrane) Intervention reviews.	Appropriate (RCT and Observational Studies)
Data Collection	From included systematic reviews.	Not Appropriate (Narrative reports of outcomes and inclusion error for RS by Hooper et al. 2015 - age > 24 months)
Limits evaluation	For included systematic reviews.	Appropriate
Quality of Evidence	So far as possible should be based on assessments reported in the included systematic reviews.	Appropriate
Analysis	Summary of review results; additional analyses may be undertaken for comparisons across reviews, typically indirect comparisons of multiple interventions.	Not Appropriate /insufficient (Narrative summary of results with no comparison between SRs. Error in reporting of results by Hooper et al.)
**Global evaluation**	**Low methodological quality, Excluded.**	

Table a1.5. SRs excluded with motivation.

SRs excluded	Reason for exclusion
Askie et al. 2020 [20]	Not appropriate (evaluates health education interventions on the mother)
Azaïs-Braesco et al. 2017 [21]	Not appropriate (survey reviews in 11 countries)
Bailey et al. 2018 [22]	Not appropriate. (Data on nutrients intake)
Blake-Lamb et al. 2016 [23]	Low methodological quality. Not appropriate. (Not evaluating single nutrient intake).
Borg et al. 2019 [24]	Not appropriate. (Data on nutrients intake)

Brown et al. 2019 [25]	Reporting aggregate data 1-5 years.
Das et al. 2019 [26]	Not appropriate. (in LIC, Low-Income Countries)
Eichler et al. 2019 [27]	Not appropriate. (children aged 5-15 years)
English et al. 2019 [28]	(Primary studies quantify food consumption, not the intake of specific nutrients.)
Grote et al. 2014 [29]	Low methodological quality. (Narrative review)
Hooper et al. 2015 [30]	Not appropriate. (children aged > 24 mo)
Huang et al. 2019 [31]	Not appropriate. (studies on adult population)
Larqué et al. 2019 [32]	Low methodological quality. (Narrative review)
Laving et al. 2018 [33]	Low methodological quality. (Narrative review)
Lin et al. 20et al. 20 [34]	Not appropriate. (nutrients supplementation in preterm and SGA children)
Kikuchi et al. 2018 [35]	Not appropriate. (not including CF studies)
Malik et al. 2013 [36]	Not appropriate. (not including CF studies)
Mazarello Paes et al. 2015 [37]	Not appropriate. (not including CF studies)
Mela et al. 2018 [38]	Not appropriate. (Perspective on the topic: sugar, total, added, or free?)
Micha et al. 2018 [39]	Not appropriate. (assesses dietary habits, not including studies on CF).
Naude et al. 2018 [40]	Not appropriate. (age 2-18 years)
Obbagy et al. 2019 [41]	Not appropriate. (primary studies quantify meat intake, not protein)
Ojha et al. 2020 [42]	Not appropriate. (Evaluates the efficiency of health education to caregivers)
Park et al. 2019 [43]	Not appropriate. (LIC)
Pieścik-Lech et al. 2017 [44]	Not appropriate. (CF studies are not included)
Pimpin et al. 2019 [45]	Not appropriate. (premature children, low weight or malnourished)
Pitangueira et al. 2015 [46]	Low methodological quality. Not appropriate. (CF not evaluated)
Redsell et al. 2015 [47]	Not appropriate. (not evaluate the number of nutrients)

Reilly et al. 2017 [48]	Not appropriate. (SR critical review)
Roberts et al. 2017 [49]	Not appropriate. (children aged > 2 years)
Roberts et al. 2017 [50]	Not appropriate. (children aged > 2 years, assesses fiscal measures)
Shulkin et al. 2018 [51]	Data from CF studies combined with data from supplementation studies at birth.
Smithers et al. 2011 [52]	Not appropriate. (Assesses dietary indices. Not specifically on CF)
Stanhope et al. 2017 [53]	Low methodological quality. (Narrative review). Not appropriate. (CF studies are not included)
Stewart et al. 2020 [54]	Not appropriate. (LIC children)
Tang et al. 2019 [55]	Low methodological quality. (Narrative review). Not appropriate. (primary studies quantify meat intake, not protein intake)
Ter Borg et al. 2019 [56]	Not appropriate. (not PICO adherent)
Vargas-Garcia et al. 2017 [57]	Not appropriate. (not PICO adherent)
Wang et al. 2013 – AHRQ [58]	Not appropriate. (age population 2-18 years)
Zalewski et al. 2015 [59]	Low methodological quality. (SR of GL before 2015)
Zhou et al. 2014 [60]	Not appropriate. (CF not evaluated)

Tablea1.6. Appraisal of the Studies

	Newcastle Quality Assessment Scale COHORT STUDIES								
	**Selection**				**Comparability**	**Outcome**			
**Study**	**Representativeness of the exposed cohort**	**Selection of the non exposed cohort**	**Ascertainment of exposure**	**Demonstration that outcome of interest was not present at the start of the study**	**Comparability of cohorts based on the design or analysis**	**Assessment of outcome**	**Was follow-up long enough for outcomes to occur?**	**Adequacy of follow up of cohorts**	**Total**
Jen et al. 2019 [61]	1a	1a	1b	1a	1a<br>1b	1a	1a	1a	9
Nguyen et al. 2020 [62]	aa	1a	1b	1a	1a<br>1b	1a	1a	1a	9
Voortman et al. 2016 [63]	1a	1a	1b	1a	1a<br>1b	1a	1a	1a	9

Table a1.7. Excluded studies with reasons for exclusion.

Excluded	Reason for exclusion
Au et al. 2018 [64]	Not appropriate (only descriptive)
Bailey et al. 2018 [65]	Not appropriate (usual intake estimated and primary food source for added sugars)
Beyerlein et al. 2017 [66]	Not appropriate (children at risk for DM1)
Breeze et al. 2018 [67]	Not appropriate (assesses trade policy intervention)
Campbell et al. 2016 [68]	Not appropriate (Evaluates different interventions. Single nutrients not evaluated)
Enö Persson et al. 2018 [69]	Not appropriate (No CF)
Gross et al. 2016 [70]	Not appropriate (No CF)
Gui et al. 2017 [71]	Not appropriate (No CF. conducted in China)
Ha et al. 2017 [72]	Not appropriate (for the outcomes considered)
Han et al. 2013 [73]	Not appropriate (for the outcomes considered. No CF)
Herran et al. 2018 [74]	Not appropriate (Inequalities. No CF.)
Hesketh et al. 2020 [75]	Not appropriate (Evaluates the effectiveness of a multilevel health education intervention to caregivers.)
Kostecka et al. 2020 [76]	Not appropriate (assesses factors inhibiting and facilitating the implementation of GL).
Lim et al. 2018 [77]	Not appropriate (only descriptive)
Morgen et al. 2018 [78]	Low methodological quality (high loss at follow-up, differences in baseline characteristics between study participants and those lost to follow-up.)
Mustila et al. 2018 [79]	Not appropriate (multilevel counseling intervention)
Nguyen et al. 2020 [80]	Not appropriate (dietetical intervention, nutrients not differentiated)
Nier et al. 2018 [81]	Not appropriate (only on obese children fructose consumption)
Niinikoski et al. 2012 [82]	Not quantify for carbohydrate intake in the CF period, but the whole duration of follow-up up to 9 years.
Okubo et al. 2015 [83]	Not appropriate (Does not assess individual nutrients but the overall Diet Quality Index (DQI))
Pan et al. 2014 [84]	Low quality (Nutrients not quantified, high loss at follow-up).
Papanikolaou et al. 2018 [85]	Not appropriate (assesses nutrient patterns associated with both consumption/nonconsumption of eggs)
Pettigrew et al. 2015 [86]	Not appropriate (No CF)
Pigeot et al. 2016 [87]	Not appropriate (No CF. In German)
Pimpin et al. 2016 [88]	Low quality (High lost at follow up >50% and partial data)
Reifsnider et al. 2018 [89]	Not appropriate (No CF)
Rigo et al. 2018 [90]	Not appropriate (No CF)
Scully et al. 2017 [91]	Not appropriate (No CF, mothers' perception of commonly consumed pre-school drinks)
Sonneville et al. 2015 [92]	Low quality (Nutrients not quantified, high loss at follow-up)
Tang et al. 2014 [93]	Very short follow-up (from 5 to 9 mo of age)
Vard et al. 2019 [94]	Not appropriate (No CF)
Wasser et al. 2017 [95]	Not appropriate (multi-level complex intervention.)
Zuccotti et al. 2014 [96]	Not appropriate (only descriptive)

A1.a. RECOMMENDATIONS OF THE GLs, RESULTS OF THE SRs AND STUDIES

- ***Does an energy intake above age-recommended levels from 6 to 24 months of age result in different short- and long-term nutritional and metabolic outcomes compared to an intake that meets recommended levels?***<br><br>- ***Does a carbohydrate intake above age-recommended levels from 6 to 24 months of age result in different short- and long-term nutritional and metabolic outcomes compared to an intake that meets recommended levels?***<br><br>- ***Does a protein intake above age-recommended levels from 6 to 24 months of age result in different short- and long-term nutritional and metabolic outcomes compared to an intake that meets recommended levels?***<br><br>- ***Does a lipid intake above age-recommended levels from 6 to 24 months of age result in different short- and long-term nutritional and metabolic outcomes compared to an intake that meets recommended levels?***	**a.**<br>**P In the healthy infant, in the period 6-24 months,**<br>**I an energy intake above the recommended levels for age**<br>**C compared with an intake corresponding to recommended levels**<br>**O results in different short- and long-term nutritional and metabolic outcomes?**<br><br>**b.**<br>**P In the healthy infant, in the period 6-24 months,**<br>**I a carbohydrate intake above the recommended levels for age**<br>**C compared with an intake corresponding to recommended levels**<br>**O results in different short- and long-term nutritional and metabolic outcomes?**<br><br>**c.**<br>**P In the healthy infant, in the period 6-24 months,**<br>**I a protein intake above the recommended levels for age**<br>**C compared with an intake corresponding to recommended levels**<br>**O results in different short- and long-term nutritional and metabolic outcomes?**<br><br>**d**<br>**P In the healthy infant, in the period 6-24 months,**<br>**I a lipid intake above the recommended levels for age**<br>**C compared with an intake corresponding to recommended levels**<br>**O results in different short- and long-term nutritional and metabolic outcomes?**

Table a1.8. Clinical Guidelines and Guidance Documents Appraisal.

Guidelines and Clinical Guidance Documents	Recommendations	Grading
SIEDP-SIP 2018 [1]	Protein intake is suggested to be below 15% of daily energy in the first 2 years of life.<br><br>Reducing lipid intake in the first 2 years of life to percentages appropriate to those of adulthood is not recommended.<br><br>The use of sweet drinks in the first 2 years of life should be avoided.	Evidence level I, strength of recommendation B. Ref. #349, 357-360<br><br>Evidence level II, strength of recommendation D. Ref #361<br><br>Evidence level III, strength of recommendation A. #362

Table a1.9. Characteristics, Results, and Conclusions of the included SRs.

**Systematic Reviews**	**Population and Purpose of SRs.**	**Results**	**Conclusions**
Hörnell et al. 2013 [16]<br>Bibliography closed February 2012	The aim is to evaluate the effects of different levels of protein intake in infancy on health, in healthy full-term children living in northern European countries.	37 studies included: 8 non-randomized controlled trials, 19 prospective cohort studies, 10 cross-sectional studies.<br><br>Study quality assessment based on AHRQ (Agency for Healthcare Research and Quality) criteria.<br><br>**A** Studies have an acceptably low level of bias and the results are considered valid.<br>**B** Studies may have some bias, but not enough to invalidate the results.<br>**C** Studies have a significant bias that may invalidate the results.<br><br>Studies adhering to PICOs<br><br>**Growth, BMI:**<br><br>*Cohort studies*<br><br>Gunnarsdottir et al. (3), graded B<br>Children in the highest quartile of protein intake (E%) at age 9-12 mo had a significantly higher BMI at 6 years compared to the lowest and second-lowest quartiles (17.8±2.4 vs 15.6±1.0 and 15.3±0.8 - P = 0.039 and P = 0.01, respectively).<br>Energy intake was not different between the groups.<br>Günther et al. (33), graded B,<br>Higher habitual protein intake between 12 and 24 mo was associated with a higher BMI-SDS at the time of AR in girls, but not in boys.<br>Günther et al. (34), graded A,<br>The relationship between different protein intakes at 6-24 mo and BMI and body fat percentage (% BF) at 7 years was evaluated using data from the DONALD study in Germany.<br>A sustained high protein intake at 12-18 and 24 mo was independently related to a higher mean BMI SDS and % BF at 7 years and a higher risk of having a BMI or % BF above the 75th percentile.<br>There was no association with protein intake at 6 mo.<br>Günther et al. (35), graded A<br>The age groups of 12 mo and 5-6 years were identified as critical periods in which higher intakes of total and animal, but not vegetable, protein were positively related to body fat at 7 years. E% animal protein at 12 mo was positively associated with BMI SDS at 7 years.<br><br>Hoppe et al. (36), graded B,<br>Danish 10-year-old children (n = 142 at 9 mo, n = 105 at 10 years) in relation to protein intake at 9 mo.	**Growth, BMI:** Convincing evidence (grade 1) that higher protein intake in infancy and early childhood is associated with greater growth and/or higher body mass index during childhood.

		Overall, 7.8% of boys and 7.5% of girls were overweight, none were obese. Protein intake (E%, g/day but not g/kg/day) at 9 mo was predictive of weight and height at 10 years.<br>Öhlund et al. (4), graded B<br>Protein intake in particular, but also total energy and carbohydrate intake at 17/18 mo and 4 years, were positively associated with BMI at 4 years.<br>Scaglioni et al. (38), graded B<br>The prevalence of overweight at age 5 was strongly associated with parental overweight (P=0.0001), and overweight children had a higher protein intake at age 1 than non-overweight children (22E% vs 20E%, P = 0.024).<br>*Cross-sectional*<br>No relevant studies.<br>**<u>Adiposity rebound</u>**<br>*Cohort studies*<br>Dorosty et al. (32), graded B<br>no evidence of an association between protein intake at 18 mo, or any other dietary variable, and the timing of AR.<br>Günther et al. (33), graded B,<br>no coherent relationship between habitual protein intake in early childhood and the timing of AR, but higher usual protein intake between 12 and 24 mo was associated with higher BMI-SDS in girls, but not in boys.<br>**<u>Glucose/insulin</u>**: no relevant studies.<br>**<u>Hypertension:</u>** no relevant studies.	
Pearce et al. 2013 [17]<br>Bibliography closed<br>End June 2012	Evaluate the evidence on the impact of different foods administered during the complementary feeding period on BMI or body composition in children aged 4-12 years.<br>Only include observational studies, evaluated with NOS, on healthy children.<br>Exposure: Nutrient intake in the CF period up to and including 12 mo.<br>Outcomes: BMI, BMI z-score, and body composition at 4-12 years, not self-reported.<br>Study data: 1959-2009.	10 articles met the selection criteria, with numbers ranging from 90 to 881 participants.<br>4/10 examined the impact of macronutrient or energy intake in childhood and its effect on childhood BMI or % BF (body fat).<br>**<u>BMI</u>**<br>Gunnarsdottir et al.<br>Günther et al.<br>Günther et al.<br>Hoppe et al.	There is some evidence that <u>high energy intake</u> in early infancy may lead to higher BMI and BF% during infancy, but this is data from a single study on formula composition (Ong et al. 23): not conclusive.<br>There is limited evidence on protein intake at 4-12 mo. <u>High protein intake</u> (% energy) at 12 mo of age and high animal and LV protein intake (% energy) at 12 mo may result in higher BMI and % BF during infancy.
Ferrè et al. 2021 [18]<br>Bibliography closed	To evaluate the effects of protein intake during the second year of life on weight and	Included 14 references from 9 studies	There is moderate evidence for an association between protein intake during the second year

30 May 2020	fat mass gain and the subsequent risk of developing obesity later in childhood.<br><br>Including all studies with this aim, research closure 30.05.2020.<br><br>Population: healthy children, without growth problems, born at term, living in developed countries.<br><br>Exposure: protein intake during the second year of life.<br><br>Outcomes:<br><br>% body fat (% BF), body fat mass (FM) (kilograms) or body fat mass index (BMI) (kilograms/meter2),<br><br>body mass index (BMI) (kilograms/meter2 or z score) and<br><br>Risk of excess weight (risks of overweight and obesity) (RR, OR, or frequency<br><br>Secondary outcomes: growth rate, adiposity rebound.	**Growth**<br><br>Outcome not considered<br><br>**Risk of obesity/overweight**<br><br>Günther et al. (32).<br><br>The DONALD cohort supported the hypothesis of an increased risk of being overweight by having a persistently higher protein intake during the first two years of life (compared to only the first year of life). The results of the Gemini cohort did not support this hypothesis. The contradictory results could be due to several factors: the Gemini cohort was performed on twins and included preterm infants, thus possibly low birth weight infants with different growth patterns than the target population.<br><br>Furthermore, in the Gemini cohort, protein intake was assessed at a mean age of 21 mo, within a range of 17-34 mo.<br><br>Therefore, some of the intake data were obtained during the third year of life instead of the second. In addition, these results were obtained at a single point in time, which could not be representative of what happened during the second year, which could hinder the extrapolation of conclusions.<br><br>**Adiposity rebound**<br><br>Several studies have investigated possible mechanisms supporting the association between the extent of protein intake in the 2nd year of life and early adiposity rebound.<br>Rolland-Cachera et al. (31)<br>Protein intake (% energy vs. protein) at 2 years of age was the start of AR ($r = 0.2$, $p = 0.02$).<br>Children with early AR (before 4 years of age) had a higher protein intake at 2 years of age than those children showing late AR (after 8 years of age) (16.6 2.1% vs. 14.9 2.1%, $p < 0.01$).<br><br>Dorosty et al (44)<br>Günther et al (45)<br><br>The available evidence consistently supports the hypothesis that protein intake during the second year of life may lead to an earlier onset of AR.<br>The DONALD and Gemini cohorts provided contradictory results. This could be due to several reasons: on the one hand, children in the Gemini cohort had a higher proportion of preterm infants and low birth weight infants who might have a different growth pattern and/or maturation rate.<br>The predicted AR as a consequence of increased protein intake during the second year of life was not confirmed by Dorosty et al. [44] in the ALSPAC cohort.<br>Another possible confounding factor could be the speed of sexual development.<br><br>**BMI**	of life and increased body fat at 2 years. The evidence supporting an increased risk of overweight or obesity at later ages was inconclusive.<br><br>Overall, the quality of evidence on effects on overweight and obesity is limited and based on observational cohort studies.<br><br>Definitive conclusions can be derived from randomized controlled intervention studies. In the Cochrane database of registered clinical trials, there is one ongoing RCT with more than 1600 children already recruited as infants (TOMI study, formula, sponsored by Nestlé, Clinical trials.gov, NTC02907502).

		Rolland-Cachera et al. (31)<br>Newborns in the highest quintile of protein intake at 2 years of age showed earlier AR and a greater increase in BMI after 4 years, showing a higher BMI at 8 years of age.<br><br>Günther et al. (32)<br>Günther et al. (33)<br><br>Karaolis-Danckert et al. (34)<br>Higher sustained protein intake during the first 2 years of life modulated the BMI z-score at 2 years (= 0.36 ±0.13, p = 0.005 for the HH group compared to the HL) but did not affect the longitudinal change in BMI z-score between 2 and 5 years.<br><br>Children with rapid growth (0-2 years) showed a higher BMI z-score (-0.016± 0.99 versus 0.41 ±0.90, p < 0.001) at 5 years. However, the distribution of fast-growing children was similar between the groups of high and low protein intakes during the first 2 years (H-H vs. H-L).<br>(high loss at follow-up).<br><br>Cowin et al. (35)<br>Protein intake at 18 mo of age was not associated with changes in BMI z score at 31 mo but was associated with height (r = 0.176)<br>(high loss at follow-up)<br>Garden et al. 2011 (36)<br>Protein intake (g/day) at 18 mo of age was associated with a higher BMI z-score at 8 years of age (10 g/day protein intake was associated with an increased BMI z-score in BMI 0.47 SD. Meat intake was also associated with BMI z-score and waist circumference at 8 years.<br>(high loss at follow-up)<br>Garden et al. 2012 (37)<br>Different sex-specific BMI growth trajectories (normal, persistent early increase, and late increase) up to the age of 11.5 years were not associated with different protein intakes at 18 mo of age.<br>(high loss at follow-up)<br><br>Öhlund et al. (38)<br><br>Pimpin et al. (39)<br>Protein intake at 21 ±1.2 mo above 16.3% of total energy intake was associated with greater weight gain up to 5 years of age (= 0.330 kg, CI 95% 0.182-0.478 for highest vs. lowest quintile).<br>(The cohort included 43.5% preterm infants).<br><br>Morgen et al. (41)	

		Protein intake from dairy products at 18 mo (for 5 g/day) increased the BMI z-score at 7 years (: 0.012, 95% CI: 0.003,0.021; p = 0.007)<br>Protein intake from meat and fish at 18 mo (by 2 g/day) increased BMI = 0.010 SD (95% CI: 0.004, 0.017; p = 0.003) or 0.013 (95% CI: 0.005, 0.020; p = 0.002) at 7 and 11 years, respectively.<br>(High drop-out rate (72%). Very little information on dietary registration.<br>Anthropometric data at 7 and 11 years reported by parents.)<br><br>The available evidence from the study cohorts suggests a possible association between protein intake during the second year of life and an increase in BMI z score thereafter. There was little evidence of this association due to the heterogeneity and considerable bias observed in the cohort studies, especially for ref.31,32,36,38,39,41.	

Table a1.10. Characteristics and Results of Included Studies.

Study	Study Design	Population	Test	Primary Outcome	Secondary Outcomes	Follow-up	Results
Nguyen et al. 2020 [62]	Cohort prospective	3629 children from *Generation R Study*, a population-based cohort study from fetal life onwards in Rotterdam, The Netherlands.	Dietary intake in early childhood was assessed using a validated semi-quantitative food frequency questionnaire (FFQ) at the age of 12.9 (12.7-19.9) mo of the child.<br>Nutrient intake was calculated using the Dutch food composition table.<br>Validation of the FFQ about three calls, 24 hours apart, in a representative sample of 32 Dutch children, showed a moderate to good ranking for nutrient intake.<br>The children's dietary intake was then assessed at the age of 8 years and the dietary quality score, a measure of adherence to age-specific dietary guidelines, was calculated.	To evaluate the association of carbohydrate intake at 12 mo with metabolic parameters such as BMI, *fat mass index* (FMI) (fat mass(kg)/height(m)2, and *fat-free mass index* (FFMI) (fat-free mass(kg)/height(m)2 at 10 years.<br>(FFMI) fat-free mass(kg)/height(m)2 at 10 years.	To evaluate the association between carbohydrate intake at 12 mo, examining the role of different macronutrient substitutions, on triglyceride, total cholesterol, HDL-cholesterol, and insulin concentrations.	10 years	The total carbohydrate intake at the age of 12.9 mo was 191.7 ± 58.9 g/day, which corresponds to 58.3 ± 6.1 E%.<br>A (cm) (n = 2984 =82%) 118.2 ± 5.2<br>P (kg) (n = 2984) 21.8 (20.2 - 24.0)<br>Body mass index (kg/m2) (n = 2984) 15.7 (15.10-16.7)<br>The results do not support that carbohydrate intake or its subtypes in early childhood is associated with different BMI/FFMI, but suggest that higher carbohydrate intake in childhood, particularly monosaccharides and disaccharides, may be associated with higher triglyceride levels and lower HDL.
Voortman et al. 2016 [63]	Cohort prospective	2911/3629 (80%) children from *Generation R Study*, a population-based cohort study from fetal life onwards in Rotterdam, The Netherlands.	See **Nguyen 2020**<br><br>To assess the effect of proteins independently of their energy content, the dietary intake of proteins and other macronutrients was adjusted for total energy intake using the nutrient residue method.	To study the associations between protein intake at age 1 and BMI, fat mass index (FMI), and lean mass index (FFMI) at age 6.	Assess these associations in a subgroup of children for protein intake at age 2 years; examine whether the associations differed by gender, ethnicity, children's genetic	6 years	At the age of 1 year, children's mean (± s.d.) daily protein intake was 41.2 g (± 12.9), which corresponds to 12.9% of their total energy intake.<br>Although this amount is higher than recommended for this age group, it is similar to the protein intake observed in other Western pediatric populations.

					risk score for BMI, birth weight or catch-up growth, or by protein source.		The mean animal protein intake was 8.1 E% (± 2.4) and the mean vegetable protein intake was 4.7 E% (± 1.4). Boys had a higher absolute protein and energy intake than girls, but the relative protein intake was the same for boys and girls (12.9 E%).<br><br><u>Macronutrient composition of the diet</u><br>Total protein intake, mean(E%) 12.9 (2.4)<br>Total fat intake (E%) 28.6 (5.6)<br>Total carbohydrate intake (E%) 58.4 (6.0)<br><br><u>Child characteristics at the 6-year visit - mean (SD)</u><br><br>A (cm) = 118.2 (5.2)<br>P (kg) = 22.4 (3.4)<br>Body mass index (kgm-2) = 16.0 (1.6)<br>Fat mass index (kgm-2) = 3.8 (1.2)<br>Fat-free mass index (kgm-2) = 11.9 (0.9)
Jen et al. 2019 [61]	Cohort prospective	573/3629 (98%) children from *Generation R Study*, a population-based cohort study from fetal life onwards in Rotterdam, The Netherlands.	See **Voortman 2016**<br>Children's dietary intake was updated to their average age of 8.0 years	Evaluate longitudinal associations of total protein intake and protein from different sources in childhood with growth and detailed measures of body composition up to the age of 10 years.	To examine whether these associations were independent of protein intake and general dietary quality in late childhood.	10 years	The mean ± SD total protein intake at the age of 1 year was 42.1 ± 13.4 g/day, corresponding to approximately 4.1 g/kg/bodyweight based on the study population averages. The mean animal protein intake was 26.5 ± 10.6 g/day and the mean vegetable protein intake was 15.1 ± 5.9 g/day. The proportion of total energy intake derived from protein intake increased from 12.9 ± 2.4% at 1 year to 16.5 ± 2.2% at 8 years.<br><br><u>Child characteristics at the 10-year growth assessment</u><br>Age (years) = 9.7 ± 0.3<br>Height (cm) = 141.6 ± 6.5<br>Weight (kg) = 34.7 ± 6.7<br>BMI (kg / m2) = 17.2 ± 2.5<br><br>In this large prospective population-based cohort study, higher protein intake, mainly from animal food sources, in childhood was persistently associated with adiposity until the age of 10 years.

							These associations were independent of protein intake or diet quality in late childhood, suggesting that an excessively high protein diet in childhood may have a lasting impact on adiposity risk. A more appropriate protein intake during this critical period of development may help in the early prevention of adiposity in childhood.

A1.a. EVIDENCE PROFILE GRADE

Table a1.11. Amount of carbohydrates. Risk of overweight/obesity.

[carbohydrate intake above age-recommended levels, 6-24 months] compared to [intake that meets recommended levels] in order to [different short- and long-term nutritional and metabolic outcomes]

Patient or population: [healthy infant, 6-24 months of age]

Setting: outpatient

Intervention: [carbohydrate intake above the recommended levels for the age group]

Comparator: [adequate intake]

Certainty assessment							Impact	Certainty of evidence	Importance
№ of studies	Study Design	Distortion risk	Lack of reproducibility of results	Lack of generalisability	Inaccuracy	Further considerations			
**Risk of overweight/obesity at 6 patient-years (assessed by: BMI)**									
2 [1,2]	Observational studies	Serious [a]	Not relevant	Not relevant	Not relevant	All plausible residual confounders could reduce the demonstrated effect	Inconsistency of results, conflicting data [a]	⊕⊕◯◯ LOW	Important.

CI: Confidence interval

Explanations

a. Inconsistency of results: Pan et al. document a higher risk of obesity at 6 years of age (17.0% vs 8.6%), in Nguyen et al. results do not support that intake of carbohydrates or its subtypes in early childhood is associated with different BMI.

References

1. Pan et al. 2014 [84]

2. Nguyen et al. 2020 [62]

Table a1.12. Amount of protein. Risk of overweight/obesity.

[protein intake above age-recommended levels, 6-24 months] compared to [intake that meets recommended levels] in order to [different short- and long-term nutritional and metabolic outcomes]

Patient or population: [healthy infant, 6-24 months of age]

Setting: outpatient

Intervention: [protein intake above the recommended levels for the age group]

Comparator: [adequate intake]

Certainty assessment							Impact	Certainty of evidence	Importance
№ of studies	Study Design	Distortion risk	Lack of reproducibility of results	Lack of generalisability	Inaccuracy	Further considerations			

Risk of overweight/obesity (follow-up: average 6-10 patient-years; assessed by: BMI)

16 [1,2,3,4,5,a]	Observational studies	Serious [b,c]	Not relevant	Not relevant	Not relevant	all residual plausible confounders would suggest a spurious effect, whereas no effect was observed	There is evidence of an association between protein intake during the second year of life and increased BMI at 6-10 years. However, the evidence supporting an increased risk of overweight or obesity at later ages is inconclusive, both because of its limited quality and because the results are conflicting.[b,c]	⨁⨁◯◯ LOW	Important.

Early adiposity rebound (follow up: interval 4 to 8 patient-years; evaluated with: % of children with adiposity rebound before the age of 4 years)

3 [6]	Observational studies	Serious [b]	Not relevant	Not relevant	Not relevant	all residual plausible confounders would suggest a spurious effect, whereas no effect was observed	Conflicting results: in 2 studies (Dorosty et al. and Günther et al.) there was no association between protein intake at 18 mo or any other dietary variable, and the timing of RA. in 1 study (Rolland-Cachera et al.) children with early RA (before 4 years of age) had a higher protein intake at 2 years of age than those children showing late RA (after 8 years of age) (16.6 ± 2.1% vs 14.9 ± 2.1%, p<0.01).	⨁⨁◯◯ LOW	Important

CI: Confidence interval

Explanation

a. Further References
   - Pearce et al. 2013 [17]
   - Patro-Golab et al. 2016 [19]
   - Weng 2012 et al. [58]

b. Inconsistency: conflicting results.

c. Low methodological quality due to: 1. high loss to follow-up, often >50%, 2. uncertain quantification of the exposure factor (amount of nutrient intake per period of supplementary feeding), 3. uncertain detection of outcomes (self-reported P and A), 4. failure to assess important confounding factors, particularly total energy intake after 2 years of age, percentage of a nutrient considered concerning total energy intake, physical activity.

References

1. Foterek K, et al. 2016. [97]

2. Voortman et al. 2016 [63]

3. Jen et al. 2019 [61]

4. Gunnarsdottir,et,al.,(3),Günther,et,al.,2006, (33),Günther,et,al.,2007,(34),Hoppe,et,al.,(36),Öhlund,et,al.,(4),Scaglioni,et,al.,(38)

In Hörnell et al. 2013 [16]

5. Garden,,et,al.,2011,(36),,Garden,,et,al.,2012,(37),,Cowin,,et,al.,(35),,Rolland-Cachera,et,al. (31), Karaolis-Danckert,,et,al.,(34), Pimpin,,et,al.(39), Morgen,et,al. (41).

In Ferrè et al. 2021 [18]

6. Dorosty,et,al,(44),Günther,et,al,(45),Rolland-Cachera,et,al.. (31)

In Ferrè et al. 2021 [18]

Table a1.13. Amount of lipid. Risk of overweight/obesity.

[lipid intake above age-recommended levels, 6-24 months] compared to [intake that meets recommended levels] in order to [different short- and long-term nutritional and metabolic outcomes]

Patient or population: [healthy infant, 6-24 months of age]

Setting: outpatient

Intervention: [lipid intake above the recommended levels for the age group]

Comparator: [adequate intake]

Certainty assessment							Impact	Certainty of evidence	Importance
№ of studies	Study Design	Distortion risk	Lack of reproducibility of results	Lack of generalisability	Inaccuracy	Further considerations			
**Long-term risk of overweight/obesity (follow-up: average 14 years; assessed by: BMI)**									

1 [1]	Randomized Studies	Very serious [a]	Not relevant	Not relevant	Not relevant	all residual plausible confounders would suggest a spurious effect, whereas no effect was observed	the STRIP study, on 1062 infants from 7 mo of age, at high cardiovascular risk, who were fed a low lipid diet (30-35% of calories/day, with a good saturated/unsaturated fat ratio). At 14 years there were no differences in BMI between the diet group and the control group without lipid restriction. [b]	⊕⊕⊕◯ Moderate	Important

CI: Confidence interval

Explanations

a. Exposure is not relevant to PICO on CF: it does not quantify intake during the period of CF, but throughout follow-up up to 9 years.

b. the STRIP study, on 1062 infants from 7 mo of age, at high cardiovascular risk, on a low lipid diet (30-35% of calories/day, with a good saturated/unsaturated fat ratio). At 14 years there were no differences in BMI between the diet group and the control group without lipid restriction.

References

1. Niinikoski et al. 2007 [98]

Appendix 1a. References

1. Alvisi P, Brusa S, Alboresi S, et al. Recommendations on complementary feeding for healthy, full-term infants. Ital J Pediatr. 2015 Apr 28;41:36.
2. Canada's Dietary Guidelines for Health Professionals and Policy Makers. https://food-guide.canada.ca/static/assets/pdf/CDG-EN-2018.pdf
3. Dereń K, Weghuber D, Caroli M, et al. Consumption of Sugar-Sweetened Beverages in Paediatric Age: A Position Paper of the European Academy of Paediatrics and the European Childhood Obesity Group. Ann Nutr Metab. 2019;74(4):296-302.
4. Scientific Opinion on nutrient requirements and dietary intakes of infants and young children in the European Union. EFSA Journal 2013;11(10):3408
5. Fewtrell M, Bronsky J, Campoy C, et al. Complementary Feeding: A Position Paper by the European Society for Paediatric Gastroenterology, Hepatology, and Nutrition (ESPGHAN). Committee on Nutrition. J Pediatr Gastroenterol Nutr. 2017 Jan;64(1):119-132
6. Fidler Mis N, Braegger C, Bronsky J, et al. Sugar in Infants, Children and Adolescents: A Position Paper of the European Society for Paediatric Gastroenterology, Hepatology and Nutrition Committee on Nutrition. J Pediatr Gastroenterol Nutr. 2017;65(6):681-696.
7. Guideline: Sugars intake for adults and children. Geneva: World Health Organization; 2015.
8. Heyman MB, Abrams SA, AAP Section on Gastroenterology, Hepatology, and Nutrition, AAP Committee on Nutrition. Fruit Juice in Infants, Children, and Adolescents: Current Recommendations. Pediatrics. 2017;139(6):e20170967
9. Kastorini CM, Critselis E, Zota D, Coritsidis AL, Nagarajan MK, Papadimitriou E, Belogianni K, Benetou V, Linos A; Greek National Dietary Guidelines Scientific Team. National Dietary Guidelines of Greece for children and adolescents: a tool for promoting healthy eating habits. Public Health Nutr. 2019 Oct;22(14):2688-2699.
10. Koletzko B, Godfrey KM, Poston L, et al. Nutrition During Pregnancy, Lactation and Early Childhood and its Implications for Maternal and Long-Term Child Health: The Early Nutrition Project Recommendations. Ann Nutr Metab. 2019;74(2):93-106. doi:10.1159/000496471
11. NICE guideline 2015. Preventing excess weight gain. nice.org.uk/guidance/ng7
12. Romero-Velardea E, Villalpando-Carrión S, Pérez-Lizaur AB, et al. Consenso para las prácticas de alimentación complementaria en lactantes sanos. Bol Med Hosp Infant Mex. 2016;73(5):338--356
13. Schwarzenberg SJ, Georgieff MK, AAP Committee on Nutrition. Advocacy for Improving Nutrition in the First 1000 Days To Support Childhood Development and Adult Health. Pediatrics. 2018;141(2):e20173716
14. U.S. Department of Health and Human Services and U.S. Department of Agriculture. 2015–2020 Dietary Guidelines for Americans. 8th Edition. December 2015. Available at http://health.gov/dietaryguidelines/2015/guidelines/.
15. Valerio G, Saggese G, Maffeis C, et al. Diagnosi, trattamento e prevenzione dell'obesità del bambino e dell'adolescente. http://www.siedp.it/files/Doc.ConsensusObesita__2017.pdf
16. Hörnell A, Lagström H, Lande B, Thorsdottir I. Protein intake from 0 to 18 years of age and its relation to health: a systematic literature review for the 5th Nordic Nutrition Recommendations. Food Nutr Res. 2013;57:10.3402/fnr.v57i0.21083.
17. Pearce J, Langley-Evans SC. The types of food introduced during complementary feeding and risk of childhood obesity: a systematic review. Int J Obes (Lond). 2013;37(4):477-485.
18. Ferré N, Luque V, Closa-Monasterolo R, Zaragoza-Jordana M, Gispert-Llauradó M, Grote V, Koletzko B, Escribano J. Association of Protein Intake during the Second Year of Life with Weight Gain-Related Outcomes in Childhood: A Systematic Review. Nutrients. 2021 Feb 10;13(2):583
19. Patro-Gołąb B, Zalewski BM, Kołodziej M, et al. Nutritional interventions or exposures in infants and children aged up to 3 years and their effects on subsequent risk of overweight, obesity and

body fat: a systematic review of systematic reviews [published correction appears in Obes Rev. 2018 Nov;19(11):1620]. Obes Rev. 2016;17(12):1245-1257.
20. Askie LM, Espinoza D, Martin A, Daniels LA, Mihrshahi S, Taylor R, Wen LM, Campbell K, Hesketh KD, Rissel C, Taylor B, Magarey A, Seidler AL, Hunter KE, Baur LA. Interventions commenced by early infancy to prevent childhood obesity-The EPOCH Collaboration: An individual participant data prospective meta-analysis of four randomized controlled trials. Pediatr Obes. 2020 Jun;15(6):e12618.
21. Azaïs-Braesco V, Sluik D, Maillot M, Kok F, Moreno LA. A review of total & added sugar intakes and dietary sources in Europe. Nutr J. 2017 Jan 21;16(1):6.
22. Bailey, R. L., Fulgoni, V. L., Cowan, A. E., & Gaine, P. C. (2018). Sources of Added Sugars in Young Children, Adolescents, and Adults with Low and High Intakes of Added Sugars. Nutrients, 10(1), 102. https://doi.org/10.3390/nu10010102
23. Blake-Lamb TL, Locks LM, Perkins ME, Woo Baidal JA, Cheng ER, Taveras EM. Interventions for Childhood Obesity in the First 1,000 Days A Systematic Review. Am J Prev Med. 2016;50(6):780-789.
24. Ter Borg, S., Koopman, N., & Verkaik-Kloosterman, J. (2019). Food Consumption, Nutrient Intake and Status during the First 1000 days of Life in the Netherlands: a Systematic Review. Nutrients, 11(4), 860. https://doi.org/10.3390/nu11040860
25. Brown T, Moore TH, Hooper L, et al. Interventions for preventing obesity in children. Cochrane Database Syst Rev. 2019;7(7):CD001871.
26. Das JK, Salam RA, Hadi YB, et al. Preventive lipid-based nutrient supplements given with complementary foods to infants and young children 6 to 23 months of age for health, nutrition, and developmental outcomes. Cochrane Database Syst Rev. 2019;5(5):CD012611.
27. Eichler K, Hess S, Twerenbold C, Sabatier M, Meier F, Wieser S. Health effects of micronutrient fortified dairy products and cereal food for children and adolescents: A systematic review. PLoS One. 2019;14(1):e0210899.
28. English LK, Obbagy JE, Wong YP, et al. Types and amounts of complementary foods and beverages consumed and growth, size, and body composition: a systematic review [published correction appears in Am J Clin Nutr. 2019 Oct 1;110(4):1041]. Am J Clin Nutr. 2019;109(Suppl_7):956S-977S.
29. Grote V, Theurich M. Complementary feeding and obesity risk. Curr Opin Clin Nutr Metab Care. 2014;17(3):273-277.
30. Hooper L, Abdelhamid A, Bunn D, Brown T, Summerbell CD, Skeaff CM. Effects of total fat intake on body weight. Cochrane Database Syst Rev. 2015;(8):CD011834. Published 2015 Aug 7.
31. Huang L, Lin JS, Aris IM, Yang G, Chen WQ, Li LJ. Circulating Saturated Fatty Acids and Incident Type 2 Diabetes: A Systematic Review and Meta-Analysis. Nutrients. 2019;11(5):998.
32. Larqué E, Labayen I, Flodmark CE, et al. From conception to infancy - early risk factors for childhood obesity. Nat Rev Endocrinol. 2019;15(8):456-478.
33. Laving AR, Hussain SR, Atieno DO. Overnutrition: Does Complementary Feeding Play a Role?. Ann Nutr Metab. 2018;73 Suppl 1:15-18.
34. Lin L, Amissah E, Gamble GD, Crowther CA, Harding JE. Impact of macronutrient supplements on later growth of children born preterm or small for gestational age: A systematic review and meta-analysis of randomized and quasirandomised controlled trials. PLoS Med. 2020 May 26;17(5):e1003122.
35. Kikuchi K, Ayer R, Okawa S, et al. Interventions integrating non-communicable disease prevention and reproductive, maternal, newborn, and child health: A systematic review. Biosci Trends. 2018;12(2):116-125.
36. Malik VS, Pan A, Willett WC, Hu FB. Sugar-sweetened beverages and weight gain in children and adults: a systematic review and meta-analysis. Am J Clin Nutr. 2013 Oct;98(4):1084-102.

37. Mazarello Paes V, Hesketh K, O'Malley C, et al. Determinants of sugar-sweetened beverage consumption in young children: a systematic review. Obes Rev. 2015;16(11):903-913.
38. Mela DJ, Woolner EM. Perspective: Total, Added, or Free? What Kind of Sugars Should We Be Talking About? Adv Nutr. 2018 Mar 1;9(2):63-69.
39. Micha R, Karageorgou D, Bakogianni I, Trichia E, Whitsel LP, Story M, Peñalvo JL, Mozaffarian D. Effectiveness of school food environment policies on children's dietary behaviors: A systematic review and meta-analysis. PLoS One. 2018 Mar 29;13(3):e0194555.
40. Naude CE, Visser ME, Nguyen KA, Durao S, Schoonees A. Effects of total fat intake on body weight in children. Cochrane Database Syst Rev. 2018;7(7):CD012960.
41. Obbagy JE, English LK, Psota TL, et al. Complementary feeding and micronutrient status: a systematic review. Am J Clin Nutr. 2019;109(Suppl_7):852S-871S.
42. Ojha S, Elfzzani Z, Kwok TC, Dorling J. Education of family members to support weaning to solids and nutrition in later infancy in term-born infants. Cochrane Database Syst Rev. 2020 Jul 25;7(7):CD012241.
43. Park JJH, Harari O, Siden E, Dron L, Zannat NE, Singer J, Lester RT, Thorlund K, Mills EJ. Interventions to improve linear growth during complementary feeding period for children aged 6-24 months living in low- and middle-income countries: a systematic review and network meta-analysis. Gates Open Res. 2020 Sep 24;3:1660.
44. Pieścik-Lech M, Chmielewska A, Shamir R, Szajewska H. Systematic Review: Early Infant Feeding and the Risk of Type 1 Diabetes. J Pediatr Gastroenterol Nutr. 2017;64(3):454-459.
45. Pimpin L, Kranz S, Liu E, et al. Effects of animal protein supplementation of mothers, preterm infants, and term infants on growth outcomes in childhood: a systematic review and meta-analysis of randomized trials. Am J Clin Nutr. 2019;110(2):410-429.
46. Pitangueira JC, Rodrigues Silva L, Costa PR. The effectiveness of intervention programs in the prevention and control of obesity in infants: a systematic review. Nutr Hosp. 2015;31(4):1455-1464
47. Redsell SA, Edmonds B, Swift JA, et al. Systematic review of randomized controlled trials of interventions that aim to reduce the risk, either directly or indirectly, of overweight and obesity in infancy and early childhood [published correction appears in Matern Child Nutr. 2016 Jul;12(3):643]. Matern Child Nutr. 2016;12(1):24-38.
48. Reilly JJ, Martin A, Hughes AR. Early-Life Obesity Prevention: Critique of Intervention Trials During the First One Thousand Days. Curr Obes Rep. 2017;6(2):127-133.
49. Roberts JL, Stein AD. The Impact of Nutritional Interventions beyond the First 2 Years of Life on Linear Growth: A Systematic Review and Meta-Analysis. Adv Nutr. 2017;8(2):323-336.
50. Roberts KE, Ells LJ, McGowan VJ, Machaira T, Targett VC, Allen RE, Tedstone AE. A rapid review examining purchasing changes resulting from fiscal measures targeted at high sugar foods and sugar-sweetened drinks. Nutr Diabetes. 2017 Dec 15;7(12):302.
51. Shulkin M, Pimpin L, Bellinger D, et al. n-3 Fatty Acid Supplementation in Mothers, Preterm Infants, and Term Infants and Childhood Psychomotor and Visual Development: A Systematic Review and Meta-Analysis. J Nutr. 2018;148(3):409-418.
52. Smithers LG, Golley RK, Brazionis L, Lynch JW. Characterizing whole diets of young children from developed countries and the association between diet and health: a systematic review. Nutr Rev. 2011;69(8):449-467.
53. Stanhope KK, Kay C, Stevenson B, Gazmararian JA. Measurement of obesity prevention in childcare settings: A systematic review of current instruments. Obes Res Clin Pract. 2017;11(5 Suppl 1):52-89.
54. Stewart CP, Wessells KR, Arnold CD, et al. Lipid-based nutrient supplements and all-cause mortality in children 6-24 months of age: a meta-analysis of randomized controlled trials. Am J Clin Nutr. 2020;111(1):207-218.
55. Tang M. The impact of complementary feeding foods of animal origin on growth and the risk of overweight in infants. Anim Front. 2019;9(4):5-11.

56. Ter Borg S, Koopman N, Verkaik-Kloosterman J. Food Consumption, Nutrient Intake and Status during the First 1000 days of Life in the Netherlands: a Systematic Review. Nutrients. 2019;11(4):860.
57. Vargas-Garcia EJ, Evans CEL, Prestwich A, Sykes-Muskett BJ, Hooson J, Cade JE. Interventions to reduce consumption of sugar-sweetened beverages or increase water intake: evidence from a systematic review and meta-analysis. Obes Rev. 2017 Nov;18(11):1350-1363.
58. Wang Y, Wu Y, Wilson RF, et al. Childhood Obesity Prevention Programs: Comparative Effectiveness Review and Meta-Analysis. Rockville (MD): Agency for Healthcare Research and Quality (US); June 2013.
59. Zalewski BM, Patro B, Veldhorst M, et al. Nutrition of infants and young children (one to three years) and its effect on later health: A systematic review of current recommendations (EarlyNutrition project). Crit Rev Food Sci Nutr. 2017;57(3):489-500.
60. Zhou YE, Emerson JS, Levine RS, Kihlberg CJ, Hull PC. Childhood obesity prevention interventions in childcare settings: systematic review of randomized and nonrandomized controlled trials. Am J Health Promot. 2014;28(4):e92-e103.
61. Jen V, Braun KVE, Karagounis LG, et al.. Longitudinal association of dietary protein intake in infancy and adiposity throughout childhood. Clin Nutr. 2019 Jun;38(3):1296-1302.
62. Nguyen AN, Santos S, Braun KVE, et al. Carbohydrate Intake in Early Childhood and Body Composition and Metabolic Health: Results from the Generation R Study. Nutrients. 2020 Jun 30;12(7):1940.
63. Voortman T, Braun KV, Kiefte-de Jong JC, et al. Protein intake in early childhood and body composition at the age of 6 years: The Generation R Study. Int J Obes (Lond). 2016 Jun;40(6):1018-25.
64. Au LE, Gurzo K, Paolicelli C, Whaley SE, Weinfield NS, Ritchie LD. Diet Quality of US Infants and Toddlers 7-24 Months Old in the WIC Infant and Toddler Feeding Practices Study-2 [published correction appears in J Nutr. 2020 Apr 1;150(4):971]. J Nutr. 2018;148(11):1786-1793.
65. Bailey RL, Fulgoni VL, Cowan AE, Gaine PC. Sources of Added Sugars in Young Children, Adolescents, and Adults with Low and High Intakes of Added Sugars. Nutrients. 2018 Jan 17;10(1):102.
66. Beyerlein A, Uusitalo UM, Virtanen SM, et al. Intake of Energy and Protein is Associated with Overweight Risk at Age 5.5 Years: Results from the Prospective TEDDY Study. Obesity (Silver Spring). 2017;25(8):1435-1441.
67. Breeze P, Womack R, Pryce R, Brennan A, Goyder E. The impact of a local sugar-sweetened beverage health promotion and price increase on sales in public leisure center facilities. PLoS One. 2018 May 30;13(5):e0194637.
68. Campbell KJ, Hesketh KD, McNaughton SA, et al. The extended Infant Feeding, Activity and Nutrition Trial (InFANT Extend) Program: a cluster-randomized controlled trial of an early intervention to prevent childhood obesity. BMC Public Health. 2016;16:166. Published 2016 Feb 18.
69. Enö Persson J, Bohman B, Tynelius P, Rasmussen F, Ghaderi A. Prevention of Childhood Obesity in Child Health Services: Follow-Up of the PRIMROSE Trial. Child Obes. 2018;14(2):99-105.
70. Gross RS, Mendelsohn AL, Gross MB, Scheinmann R, Messito MJ. Randomized Controlled Trial of a Primary Care-Based Child Obesity Prevention Intervention on Infant Feeding Practices. J Pediatr. 2016;174:171-177.e2.
71. Gui ZH, Zhu YN, Cai L, Sun FH, Ma YH, Jing J, Chen YJ. Sugar-Sweetened Beverage Consumption and Risks of Obesity and Hypertension in Chinese Children and Adolescents: A National Cross-Sectional Analysis. Nutrients. 2017 Nov 30;9(12):1302.
72. Ha DH, Do LG, Spencer AJ, Thomson WM, Golley RK, Rugg-Gunn AJ, Levy SM, Scott JA. Factors Influencing Early Feeding of Foods and Drinks Containing Free Sugars-A Birth Cohort Study. Int J Environ Res Public Health. 2017 Oct 23;14(10):1270.

73. Han E, Powell LM. Consumption patterns of sugar-sweetened beverages in the United States. J Acad Nutr Diet. 2013 Jan;113(1):43-53.
74. Herran OF, Patiño GA, Gamboa EM. Consumption of sweetened-beverages and poverty in Colombia: when access is not an advantage. BMC Public Health. 2018 Jan 15;18(1):136.
75. Hesketh KD, Salmon J, McNaughton SA, Crawford D, Abbott G, Cameron AJ, Lioret S, Gold L, Downing KL, Campbell KJ. Long-term outcomes (2 and 3.5 years post-intervention) of the INFANT early childhood intervention to improve health behaviors and reduce obesity: cluster randomised controlled trial follow-up. Int J Behav Nutr Phys Act. 2020 Jul 25;17(1):95.
76. Jen V, Braun KVE, Karagounis LG, Nguyen AN, Jaddoe VWV, Schoufour JD, Franco OH, Voortman T. Longitudinal association of dietary protein intake in infancy and adiposity throughout childhood. Clin Nutr. 2019 Jun;38(3):1296-1302.
77. Kostecka M, Jackowska I, Kostecka J. Factors Affecting Complementary Feeding of Infants. A Pilot Study Conducted after the Introduction of New Infant Feeding Guidelines in Poland. Nutrients. 2020 Dec 28;13(1):61.
78. Lim SX, Toh JY, van Lee L, et al. Food Sources of Energy and Macronutrient Intakes among Infants from 6 to 12 Months of Age: The Growing Up in Singapore Towards Healthy Outcomes (GUSTO) Study. Int J Environ Res Public Health. 2018;15(3):488. Published 2018 Mar 10.
79. Morgen CS, Ängquist L, Baker JL, et al. Breastfeeding and complementary feeding in relation to body mass index and overweight at ages 7 and 11 y: a path analysis within the Danish National Birth Cohort. Am J Clin Nutr. 2018;107(3):313-322.
80. Mustila T, Raitanen J, Keskinen P, Luoto R. A pragmatic controlled trial to prevent childhood obesity within a risk group at maternity and child health-care clinics: results up to six years of age (the VACOPP study). BMC Pediatr. 2018;18(1):89. Published 2018 Feb 27.
81. Nguyen A.N., Jen V., Jaddoe V.W.V., et al. Diet quality in early and mid-childhood in relation to trajectories of growth and body composition. Clinical Nutrition 2020 39:3 (845-852)
82. Nier A, Brandt A, Conzelmann IB, Özel Y, Bergheim I. Non-Alcoholic Fatty Liver Disease in Overweight Children: Role of Fructose Intake and Dietary Pattern. Nutrients. 2018 Sep 19;10(9):1329.
83. Niinikoski H, Ruottinen S. Is carbohydrate intake in the first years of life related to future risk of NCDs?. Nutr Metab Cardiovasc Dis. 2012;22(10):770-774. Okubo H, Crozier SR, Harvey NC, et al. Diet quality across early childhood and adiposity at 6 years: the Southampton Women's Survey. Int J Obes (Lond). 2015;39(10):1456-1462.
84. Pan L, Li R, Park S, Galuska DA, Sherry B, Freedman DS. A longitudinal analysis of sugar-sweetened beverage intake in infancy and obesity at 6 years. Pediatrics. 2014;134 Suppl 1(Suppl 1):S29-S35.
85. Papanikolaou Y, Fulgoni VL 3rd. Egg Consumption in Infants is Associated with Longer Recumbent Length and Greater Intake of Several Nutrients Essential in Growth and Development. Nutrients. 2018;10(6):719. Published 2018 Jun 4.
86. Pettigrew S, Jongenelis M, Chapman K, Miller C. Factors influencing the frequency of children's consumption of soft drinks. Appetite. 2015 Aug;91:393-8.
87. Pigeot I, De Henauw S, Ahrens W. Die IDEFICS-Primärprävention als gutes Praxisbeispiel [The IDEFICS primary prevention as a good practice example]. Bundesgesundheitsblatt Gesundheitsforschung Gesundheitsschutz. 2016;59(11):1385-1393.
88. Pimpin L, Jebb S, Johnson L, et al.. Dietary protein intake is associated with body mass index and weight up to 5 y of age in a prospective cohort of twins. Am J Clin Nutr. 2016;103(2):389-397.
89. Reifsnider E, McCormick DP, Cullen KW, et al. Randomized Controlled Trial to Prevent Infant Overweight in a High-Risk Population. Acad Pediatr. 2018;18(3):324-333.
90. Rigo M, Willcox J, Spence A, Worsley A. Mothers' Perceptions of Toddler Beverages. Nutrients. 2018 Mar 19;10(3):374.

91. Scully M, Morley B, Niven P, Crawford D, Pratt IS, Wakefield M. Factors associated with high consumption of soft drinks among Australian secondary-school students. Public Health Nutr. 2017 Sep;20(13):2340-2348.
92. Sonneville KR, Long MW, Rifas-Shiman SL, Kleinman K, Gillman MW, Taveras EM. Juice and water intake in infancy and later beverage intake and adiposity: could juice be a gateway drink?. Obesity (Silver Spring). 2015;23(1):170-176.
93. Tang M, Krebs NF. High protein intake from meat as complementary food increases growth but not adiposity in breastfed infants: a randomized trial. Am J Clin Nutr. 2014;100(5):1322-1328.
94. Vard B., Vatankhah Z., Riahi R, et al. Association of early life factors with weight disorders and abdominal obesity in children and adolescents: The CASPIAN-V study. Mediterranean Journal of Nutrition and Metabolism 2019 12:2 (173-185).
95. Wasser HM, Thompson AL, Suchindran CM, et al. Family-based obesity prevention for infants: Design of the "Mothers & Others" randomized trial. Contemp Clin Trials. 2017;60:24-33.
96. Zuccotti GV, Cassatella C, Morelli A, et al. Nutrient intake in Italian infants and toddlers from North and South Italy: the Nutrintake 636 study. Nutrients. 2014;6(8):3169-3186. Published 2014 Aug 8.
97. Foterek K, Hilbig A, Kersting M, et al. Age and time trends in the diet of young children: results of the Donald study,Eur J Nutr 2016, 55:611-20.
98. Niinikoski H, Lagström H,Jokinen E,et al. Impact of repeated dietary counseling between infancy and 14 years of age on dietary intakes and serum lipids and lipoproteins The STRIP Study. Circulation 2007, 116:1032-40.

Recommendation 8

A1.b. Salt and hypertension

- Can excessive salt intake during the CF period lead to hypertension later in life?

P. In a healthy infant aged 6-24 months
I lower or higher intakes of salt with the complementary diet
C. compared to the recommended intake
O. leads to a different risk of hypertension at later ages

Systematic reviews research

PubMed

("Sodium Chloride"[Mesh] OR "salt"[All Fields]) OR "Sodium, Dietary"[Mesh] OR "Sodium Chloride, Dietary"[Mesh]) AND "Hypertension"[Mesh] AND ((Meta-Analysis[ptyp] OR systematic[sb]) AND ("*2009/10/16"[PDat] : "2021/06/25*"[PDat]) AND "humans"[MeSH Terms] AND ("infant"[MeSH Terms] OR "child"[MeSH Terms] OR "adolescent"[MeSH Terms])

EMBASE

('sodium chloride'/exp OR 'salt intake'/exp) AND 'hypertension'/exp AND [2017-2021]/py AND ('meta analysis'/de OR 'systematic review'/de) AND ([child]/lim OR [infant]/lim OR [young adult]/lim)

COCHRANE

hypertension AND *sodium chloride* in Title Abstract Keyword - with Cochrane Library publication date Between Jun 2009 and Mar 2021, in Cochrane Reviews (Word variations have been searched)

Studies Research

PubMed

#1

(("Sodium Chloride"[Mesh] OR "salt"[All Fields]) OR "Sodium, Dietary"[Mesh] OR "Sodium Chloride, Dietary"[Mesh]) AND "Hypertension"[Mesh] AND ((Clinical Trial[ptyp] OR Observational Study[ptyp] OR Pragmatic Clinical Trial[ptyp] OR Randomized Controlled Trial[ptyp]) AND ("2017/03/01"[PDAT] : "2019/03/15"[PDAT]) AND "humans"[MeSH Terms] AND ("infant"[MeSH Terms] OR "child"[MeSH Terms] OR "adolescent"[MeSH Terms]))

#2

(("Sodium Chloride"[Mesh] OR "Sodium, Dietary"[Mesh] OR "Sodium Chloride, Dietary"[Mesh]) OR "salt"[All Fields]) AND "Hypertension"[Mesh] AND ("Infant Nutritional Physiological Phenomena"[Mesh] OR "Weaning"[Mesh]))

EMBASE

('sodium chloride'/exp OR 'salt intake'/exp) AND 'hypertension'/exp AND [2009-2021]/py AND ('case control study'/de OR 'cohort analysis'/de OR 'controlled study'/de OR 'cross sectional study'/de OR 'intervention study'/de OR 'major clinical study'/de OR 'observational study'/de OR 'prospective study'/de OR 'randomized controlled trial'/de) AND ([child]/lim OR [infant]/lim OR [young adult]/lim)

COCHRANE

hypertension AND sodium chloride in Title Abstract Keyword - in Cochrane Reviews, Trials with 'Child Health' in Cochrane Groups (Word variations have been searched) Cochrane Library publication date from Mar 2017 to Mar 2021in Trials with Child Health in Cochrane Groups (Word variations have been searched)

Figure a1.4. Guidelines search flow diagram (from General hypertension)

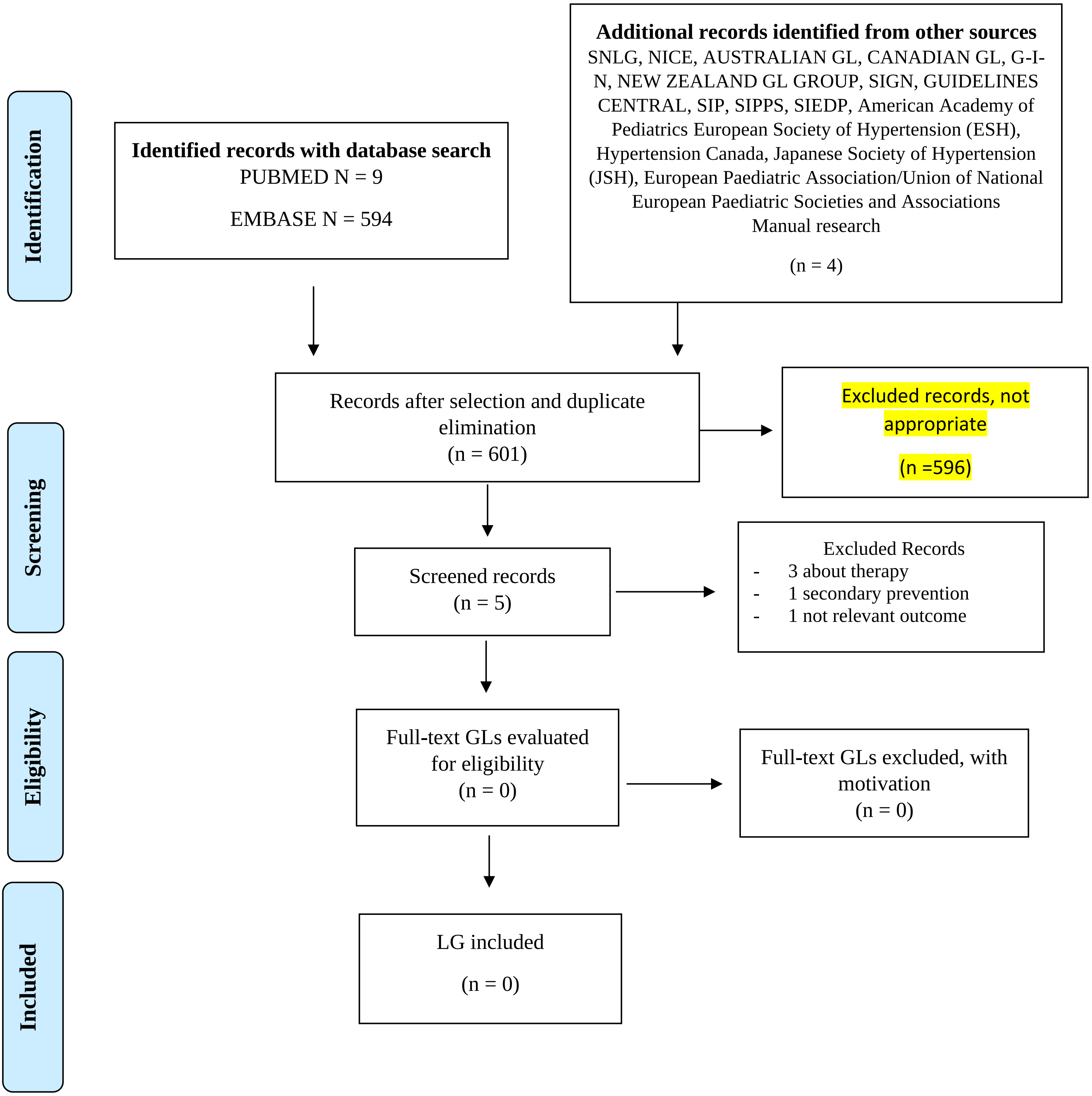

Figure a1.5. **SRs search flow diagram**

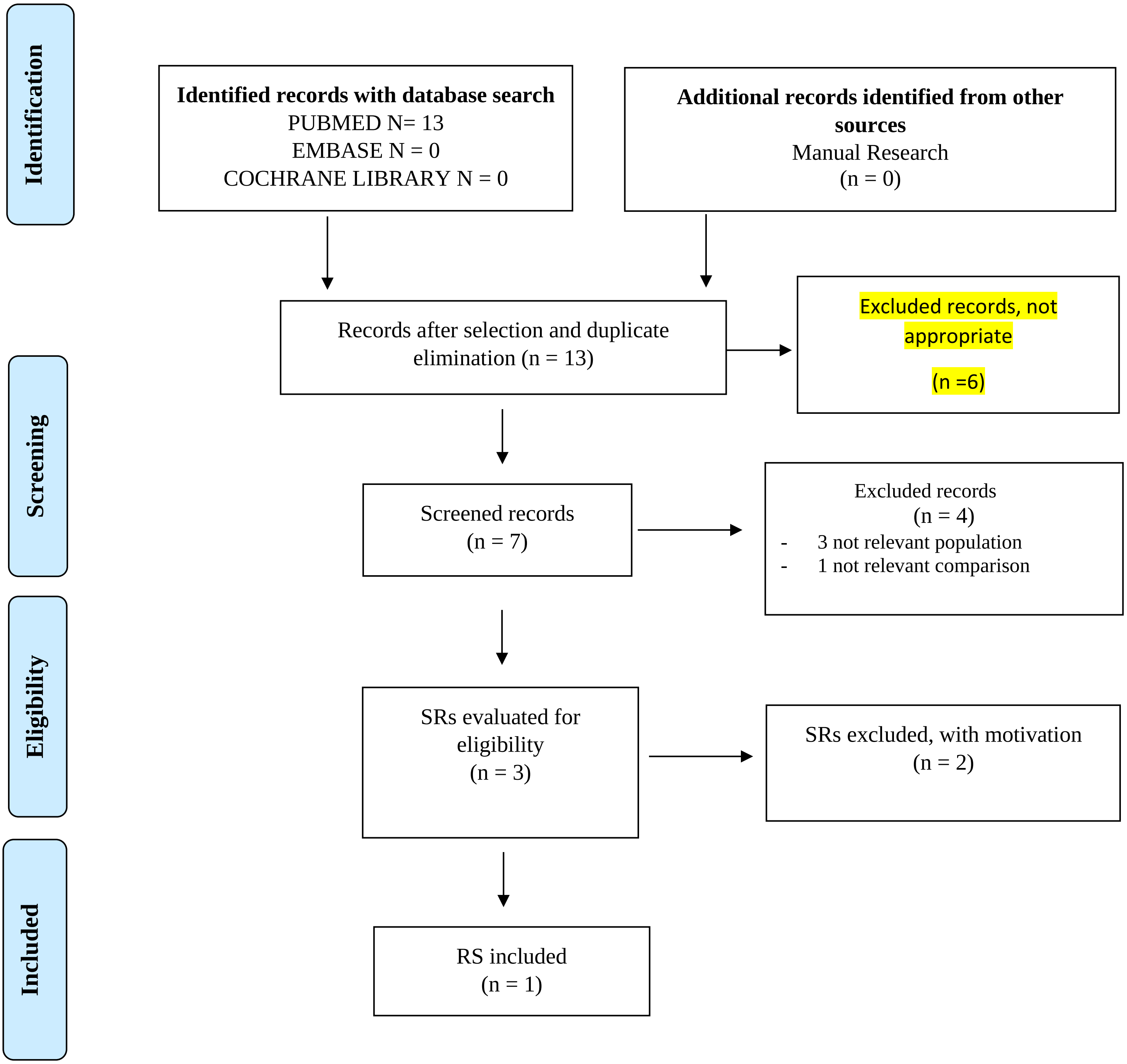

Figure a1.6. Studies search flow diagram.

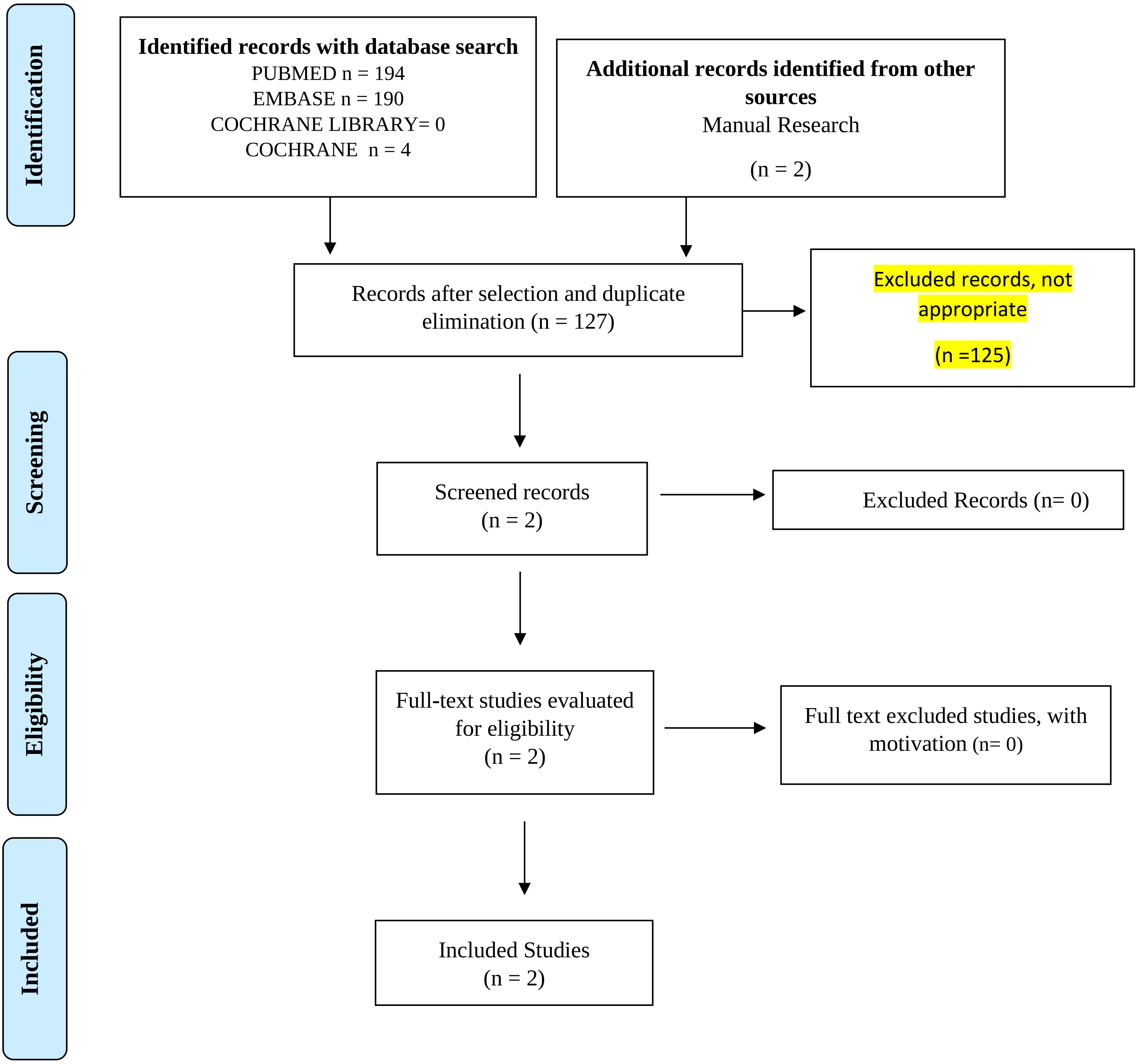

A1.b. METHODOLOGICAL EVALUATION

Appraisal of the Clinical Guidelines and Documents

None included

Table a1.14. Appraisal of the Systematic Reviews.

**AMSTAR 2**	Leyvraz et al. 2018 [1]
**1. Did the research questions and inclusion criteria for the review include the components of PICO?** **(Yes/No)**	No
**2. Did the report of the review contain an explicit statement that the review methods were established before the conduct of the review and did the report justify any significant deviations from the protocol?** **(Yes/Partial Yes/No)**	Yes
**3. Did the review authors explain their selection of the study designs for inclusion in the review?** **(Yes/No)**	Yes
**4. Did the review authors use a comprehensive literature search strategy?** **(Yes/Partial Yes/No)**	Yes
**5. Did the review authors perform study selection in duplicate?** **(Yes/No)**	Yes
**6. Did the review authors perform data extraction in duplicate?** **(Yes/No)**	Yes
**7. Did the review authors provide a list of excluded studies and justify the exclusions?** **(Yes/Partial Yes/No)**	No
**8. Did the review authors describe the included studies in adequate detail?** **(Yes/Partial Yes/No)**	Partial Yes
**9. Did the review authors use a satisfactory technique for assessing the risk of bias (RoB) in individual studies that were included in the review?** **(Yes/Partial Yes/No/Includes only NRSI-RCT)**	Yes

	Yes
**10. Did the review authors report on the sources of funding for the studies included in the review?**<br><br>**(Yes/No)**	No
**11. If meta-analysis was performed did the review authors use appropriate methods for statistical combination of results?**<br><br>**(Yes / No / No meta-analysis conducted)**	Yes
**12. If meta-analysis was performed, did the review authors assess the potential impact of RoB in individual studies on the results of the meta-analysis or other evidence synthesis?**<br><br>**(Yes / No / No meta-analysis conducted)**	Yes
**13. Did the review authors account for RoB in individual studies when interpreting/ discussing the results of the review?**<br><br>**(Yes/No)**	Yes
**14. Did the review authors provide a satisfactory explanation for, and discussion of, any heterogeneity observed in the results of the review?**<br><br>**(Yes/No)**	Yes
**15. If they performed quantitative synthesis did the review authors carry out an adequate investigation of publication bias (small study bias) and discuss its likely impact on the results of the review?**<br><br>**(Yes / No / No meta-analysis conducted)**	Yes
**16. Did the review authors report any potential sources of conflict of interest, including any funding they received for conducting the review?**<br><br>**(Yes/No)**	Yes
**OVERALL EVALUATION**	**Good Quality**

Table a1.15. SRs excluded with motivation.

**SRs excluded**	**Reason for exclusion**

Strazzullo et al. 2012 [2]	Includes only 2 pediatric studies, but on infants and with follow-up less than the first 6 months of life (Hofman 1983 and Pomeranz 2002; also contained in the included RS of Leyvraz 2018).
Zalewski et al. 2017 [3]	Review of recommendations from the previous RS.

Table a1.16. Appraisal of the Studies.

	Newcastle Quality Assessment Scale CROSS-SECTIONAL STUDIES							
	Selection				Comparability	Outcome		
**Study**	**Representativeness of the sample**	**Sample size:**	**Non-respondents**	**Ascertainment of the exposure (max 2)**	**Comparability between groups, confounders are controlled (Maximum 2 stars)**	**Outcome evaluation (max 2)**	**Statistical test**	**Total**
Lakatos et al. 2015 [4]	0 c	0b	1a	1a	1a<br>1b	2a	1a	**7**

Figure 1.7 Risk of bias graph: review authors' judgments about each risk of bias item presented as percentages across all included studies [5]

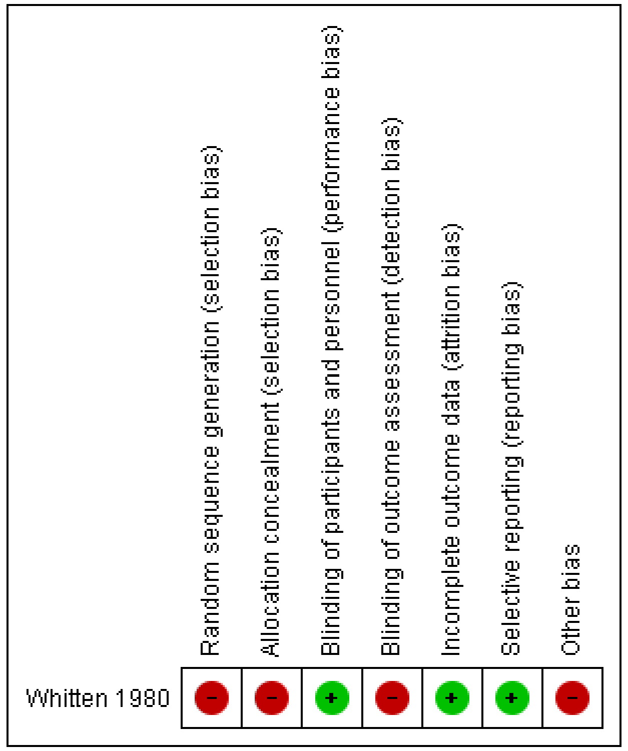

Table a1.17. Excluded studies with motivation.

**Excluded studies.**	**Reason for exclusion**
Schachter et al. 1979 (cohort perspective) [6]	A cohort followed only up to 6 months
Pomeranz et al. 2002 (RCT) [7]	Infants in the first 6 months of life<br>Comparison of low-sodium and high-sodium formula
Hofman et al. 1983 (RCT) [8]	Infants in the first 6 months of life<br>Comparison of low-sodium formula and normal-sodium formula
Dominguez Cancino et al. 2017 (cross-sectional) [9]	Prevalence study >15 years of age
Chmielewski et al. 2018 (cross-sectional; Survey) [10]	Study on subjects >12 years of age

A1.b. RECOMMENDATIONS OF THE GLs, RESULTS OF THE SRs AND STUDIES

Can excessive salt intake during the Complementary Feeding period lead to hypertension later in life?	**P.** In a healthy infant aged 6-24 months<br>**I** lower or higher intakes of salt with the complementary diet<br>**C.** compared to the recommended intake<br>**O.** leads to a different risk of hypertension at later ages

Table a1.18. Characteristics, Results, and Conclusions of SRs

Systematic Review	Population and purpose of the SR	Results	Conclusions
Leyvraz et al. 2018 [1] Bibliography closed March 2017<br><br>RS of RCTs and observational studies (only 6 studies with subjects < 2 years old)	Children or adolescents (0-18 years) without selection for sex, initial weight, and baseline blood pressure level. Studies involving children and adults were included only if data for children could be extracted separately.<br><br>Primary objective: to demonstrate whether there is an association between sodium intake and blood pressure in children and adolescents.<br><br>Secondary objective: to assess whether this association differs according to age and body weight.	A meta-analysis of intervention studies (11 papers, after elimination of outliers) showed an increase in systolic blood pressure at the end of follow-up of 0.6 mmHg (IC95% 0.5-08) and diastolic blood pressure of 1.2 mmHg (IC95% 0.4-1.9).<br><br>Differences, as assessed by subgroup meta-analysis of regression coefficients, were greatest in overweight children, in those with lower potassium intakes, and, for systolic blood pressure, in children under one year of age (but with excessively large IC95%) and preadolescents and adolescents.	Experimental and observational studies with sodium intake and high-quality blood pressure measurement methods consistently showed that sodium intake was associated with increased systolic and diastolic blood pressure. The association between sodium intake and blood pressure tended to be stronger among overweight children and children with a low potassium intake. Exploratory dose-response gradient analysis showed an almost linear increase in systolic and diastolic blood pressure within the usual range of daily sodium intake.

Table a1.19. Characteristics and Results of Included Studies.

Study	Study Design	Population	Test	Primary Outcome	Secondary Outcomes	Follow-up	Results
Whitten et al. 1980 [5]	In parallel groups, controlled	27 healthy infants enrolled at 3 months of age.<br>One group with controlled Na+ intake of 2 mEq/100 Kcal, one group 9 mEq/100 Kcal	One group with controlled Na+ intake of 2 mEq/100 Kcal, one group 9 mEq/100 Kcal	Blood pressure at 4 and 8 months	Blood pressure at 8 years of age	5 months (last measured at 8 months of age)	Non-significant differences in systolic and diastolic pressures at all ages.
Lakatos et al. 2015 [4]	Cross-sectional	N = 200<br><br>Age 1-18 years (7 under 3 years)	Na+ and K+ intakes (from urinary excretion determination/24 hours)	Measurement of Sodium and Potassium intakes<br>Relationships between Sodium and Potassium Intake and Hypertension, with adjustments for age, sex, and BMI	/	/	The correlation between dietary salt intake and systolic blood pressure was not significant after logistic regression analysis.

A1.b. EVIDENCE PROFILE GRADE

Table a1.20. Quantity of salt. Risk of Hypertension.

[higher salt intake with CF, in a healthy infant aged 6-24 months] than [recommended intake] for [leads to a different risk of hypertension at later ages].

Patient or population: In a healthy infant aged 6-24 months

Setting: Outpatient

Intervention: [increased salt intake with Supplementary Feeding].

Comparator: [recommended intake]

Certainty assessment							Impact	Certainty of evidence	Importance
№ of studies	Study Design	Distortion risk	Lack of reproducibility of results	Lack of generalisability	Inaccuracy	Further considerations			

Blood pressure (exposure time: interval 5 months to 8 years; assessed with: mm Hg).

Certainty assessment							Impact	Certainty of evidence	Importance
**№ of studies**	**Study Design**	**Distortion risk**	**Lack of reproducibility of results**	**Lack of generalisability**	**Inaccuracy**	**Further considerations**			
2 [1,2]	Observational studies [a]	Serious [a]	Not relevant	Not relevant	Not relevant	All plausible residual confounders could reduce the demonstrated effect	In both studies: non-significant differences in systolic and diastolic pressures, at all ages	⊕⊕◯◯ LOW	Important

CI: Confidence interval

Explanations

a. Whitten 1980 non-randomized controlled clinical trial

References

1. Whitten et al. 1980 [5]
2. Lakatos et al. 2015 [4]

Appendix 1b. References

1. Leyvraz M, Chatelan A, da Costa BR, Taffé P, Paradis G, Bovet P, Bochud M, Chiolero A. Sodium intake and blood pressure in children and adolescents: a systematic review and meta-analysis of experimental and observational studies. Int J Epidemiol. 2018 Dec 1;47(6):1796-1810
2. Strazzullo P, Campanozzi A, Avallone S. Does salt intake in the first two years of life affect the development of cardiovascular disorders in adulthood? Nutr Metab Cardiovasc Dis. 2012 Oct;22(10):787-92.
3. Zalewski BM, Patro B, Veldhorst M et al. Nutrition of infants and young children (one to three years) and its effect on later health: A systematic review of current recommendations (EarlyNutrition project). Crit Rev Food Sci Nutr. 2017 Feb 11;57(3):489-500
4. Lakatos O, Gyorke Z, Sulyok E. Sodium and potassium intake in Hungarian children and adolescents: comparison of two cross-sectional studies. Acta Aliment 2015;44:139–49.
5. Whitten CF, Stewart RA. The effect of dietary sodium in infancy on blood pressure and related factors. Studies of infants fed salted and unsalted diets for five months at eight months and eight years of age. Acta Paediatr Scand Suppl. 1980;279:1-17.
6. Chmielewski J, Carmody JB. Dietary sodium, dietary potassium, and systolic blood pressure in US adolescents. J Clin Hypertens (Greenwich). 2017 Sep;19(9):904-909.
7. Domínguez Cancino K, Paredes Escobar MC. [Impact of high salt consumption of blood pressure on a non-hypertensive population]. Rev Med Chil. 2017 Dec;145(12):1597-1604
8. Hofman A, Hazebroek A, Valkenburg HA. A randomized trial of sodium intake and blood pressure in newborn infants. JAMA 1983;250:370–73.
9. Pomeranz A, Dolfin T, Korzets Z, Eliakim A, Wolach B. Increased sodium concentrations in drinking water increase blood pressure in neonates. J Hypertens 2002;20:203–07
10. Schachter J, Kuller LH, Perkins JM, Radin ME. Infant blood pressure and heart rate: relation to ethnic group (black or white), nutrition and electrolyte intake. Am J Epidemiol 1979; 110:205–18.
