# Supplementary material for: Recommendations on Complementary Feeding as a Tool for Prevention of Non-Communicable Diseases (NCDs)—Paper Co-Drafted by the SIPPS, FIMP, SIDOHaD, and SINUPE Joint Working Group
Source: Nutrients. 2022 Jan 7;14(2):257. doi: 10.3390/nu14020257 (PMC8778394; doi:10.3390/nu14020257)
Supplement: Supplementary file 1 [file nutrients-14-00257-s001.zip › nutrients-1513088-supplementary/Supplementary File S3.pdf]

APPENDIX 3

Recommendations 14-18

A3. Cow milk: when, how much, and metabolic consequences

Key questions

- Does feeding with cow's milk before 12 months of age, compared to formula feeding, lead to different nutritional and metabolic outcomes in the short and long term?

- Does feeding with unmodified cow's milk after 12 months of age, compared to growth formula, result in negative short- and long-term metabolic effects?

a.
P In a healthy infant not fed with breast milk

I the feeding with unmodified cow's milk before the 12th month

C compared to formula intake

O results in different nutritional and metabolic outcomes in the short and long term?

b.
P In healthy children aged 12 to 24 months
I the intake of unmodified cow's milk
C compared to formula (growth) feeding?
O results in different short- and long-term nutritional and metabolic outcomes?

KEYWORDS

Population

A. Infant
B. Child
C. Adolescent

Exposure Factors / Comparison

<u>MeSH Terms/ Text word</u>:

A. bottle feeding
B. bottle fed
C. breastfeeding
D. human milk
E. Cattle milk

F. Cow milk
G. bovine milk
H. cows milk
I. milk consumption
J. weaning
K. Complementary feeding

Outcomes

A. Nutritional Status
B. Nutrition Assessment
C. Growth
D. Growth and Development
E. Metabolism
F. Noncommunicable diseases
G. Infant Nutrition Disorders
H. Diabetes mellitus
I. Body height
J. Body weight
K. Iron deficiency anemia
L. Diabetes mellitus

Guidelines search

Temporal limitation: 2014-2021

PUBMED https://www.ncbi.nlm.nih.gov/pubmed/

#1

("Weaning"[All Fields] OR "Infant Nutritional Physiological Phenomena"[MeSH Terms] OR "complementary feeding"[All Fields]) AND (("cattle"[MeSH Terms] OR "cattle"[All Fields] OR "cow"[All Fields]) AND ("milk, human"[MeSH Terms] OR ("milk"[All Fields] AND "human"[All Fields]) OR "human milk"[All Fields] OR "milk"[All Fields] OR "milk"[MeSH Terms])) AND AND (guideline[Filter]) AND 2011/01/01:2021/03/14[Date - Publication] AND "humans"[MeSH Terms] AND "infant"[MeSH Terms])

#2

(("cattle"[MeSH Terms] OR "cattle"[All Fields] OR "cow"[All Fields]) AND ("milk, human"[MeSH Terms] OR ("milk"[All Fields] AND "human"[All Fields]) OR "human milk"[All Fields] OR "milk"[All Fields] OR "milk"[MeSH Terms])) AND ((y_5[Filter]) AND (guideline[Filter]))

#3

(((("cattle milk"[All Fields] OR "Milk"[MeSH Terms] OR "bovine milk"[All Fields] OR "cows milk"[All Fields] OR "cow milk"[All Fields] OR "milk consumption"[All Fields]) AND ("Nutritional Status"[MeSH Terms] OR "Nutrition Assessment"[MeSH Terms] OR "Growth"[MeSH Terms] OR "Growth and Development"[MeSH Terms] OR "Metabolism"[MeSH Terms] OR ("noncommunicable diseases"[MeSH Terms] OR "Infant Nutrition Disorders"[MeSH Terms]))) NOT ("Premature Birth"[MeSH Terms] OR "infant, premature"[MeSH Terms])) AND ("humans"[MeSH Terms] AND ("infant"[MeSH Terms] OR "child"[MeSH Terms] OR "adolescent"[MeSH Terms]))) AND (guideline[Filter] OR practiceguideline[Filter])

EMBASE https://www.embase.com

#1

'complementary feeding'/exp OR 'weaning'/exp AND 'cow milk'/exp OR 'bottle feeding'/exp

AND 'practice guideline'/de AND (2016:py OR 2017:py OR 2018:py OR 2019:py OR 2020:py OR 2021:py)

#2

'cow milk'/exp OR 'bottle feeding'/exp AND 'iron deficiency anemia'/exp OR 'diabetes mellitus'/exp OR 'body height'/exp OR 'body weight'/exp AND 'practice guideline'/de AND (2016:py OR 2017:py OR 2018:py OR 2019:py OR 2020:py OR 2021:py)

UPTODATE https://www.uptodate.com/home

Society Guideline Links: *breastfeeding and infant nutrition*; *cow-milk*

SOCIETY GUIDELINE LINKS: *complementary feeding, weaning, cow milk, breast feeding*

National Guideline Clearinghouse (NGC) https://www.ahrq.gov/gam/index.html

Canadians Medical Association (CMA) https://www.cma.ca/clinicalresources/practiceguidelines

National Guideline Centre (NGC) - National Institute of Health and Care Excellence (NICE) https://www.rcplondon.ac.uk/about-us/what-we-do/national-guideline-centre-ngc

Scottish Intercollegiate Guidelines Network (SIGN) https://www.sign.ac.uk/our-guidelines.html

Australian Clinical Practice Guidelines (ACPG) https://www.clinicalguidelines.gov.au/

New Zealand Guidelines Group (NZGG) https://www.health.govt.nz/about-ministry/ministry-health-websites/new-zealand-guidelines-group

American Academy of Pediatrics (AAP) https://www.aap.org/en-us/Pages/Default.aspx

DateRange (01/01/2013-03/19/2019) AND ((complementary feeding) OR (weaning)) AND (Guideline)

North American Society for Pediatric Gastroenterology, Hepatology and Nutrition (NASPGHAN) https://www.naspghan.org/

European Society for Paediatric Gastroenterology Hepatology and Nutrition (ESPGHAN) http://www.espghan.org/

Società Italiana di Nutrizione Umana (SINU) http://www.sinu.it

Società Italiana di Pediatria (SIP) http://www-sip.it/

Società Italiana di Pediatria Preventiva e Sociale (SIPPS) https://www.sipps.it/

Società Italiana di Nutrizione Pediatrica (SINUPE) https://www.sip.it/2017/09/21/sinupe-societa-italiana-di-nutrizione-pediatrica/

Società Italiana di Gastroenterologia Epatologia e Nutrizione Pediatrica (SIGENP) http://www.sigenp.org

Systematic Reviews search

COCHRANE LIBRARY

#1

"complementary feeding " in Title Abstract Keyword - with Publication Year from 2011 to 2021, with Cochrane Library publication date Between Jan 2011 and Jan 2021 Cochrane Review matching

PUBMED

#1

("cattle"[MeSH Terms] OR "cattle"[All Fields] OR "cow"[All Fields]) AND ("milk, human"[MeSH Terms] OR ("milk"[All Fields] AND "human"[All Fields]) OR "human milk"[All Fields] OR "milk"[All Fields] OR "milk"[MeSH Terms]) AND systematic[sb]

EMBASE

#1

(complementary AND 'feeding'/exp OR 'weaning'/exp) AND 'cow milk'/exp OR 'bottle feeding'/exp

1 AND ('meta analysis'/de OR 'systematic review'/de) AND (2011:py OR 2012:py OR 2013:py OR 2014:py OR 2015:py OR 2016:py OR 2017:py OR 2018:py OR 2019:py OR 2020:py OR 2021:py)

Studies Research

PUBMED

#1

(("cattle"[MeSH Terms] OR "cattle"[All Fields] OR "cow"[All Fields]) AND ("milk, human"[MeSH Terms] OR ("milk"[All Fields] AND "human"[All Fields]) OR "human milk"[All Fields] OR "milk"[All Fields] OR "milk"[MeSH Terms])) AND ((y_5[Filter]) AND (randomizedcontrolledtrial[Filter]))

EMBASE

#1

(complementary AND 'feeding'/exp OR 'weaning'/exp) AND 'cow milk'/exp OR 'bottle feeding'/exp

AND ('controlled clinical trial'/de OR 'randomized controlled trial'/de) AND (2015:py OR 2016:py OR 2017:py OR 2018:py OR 2019:py OR 2020:py OR 2021:py)

COCHRANE LIBRARY

#1

" complementary feeding " in Title Abstract Keyword - with Publication Year from 2016 to 2021, with Cochrane Library publication date Between Jan 2016 and Jan 2021, in Trials (Word variations have been searched)

Figure a3.1. Guidelines search flow diagram

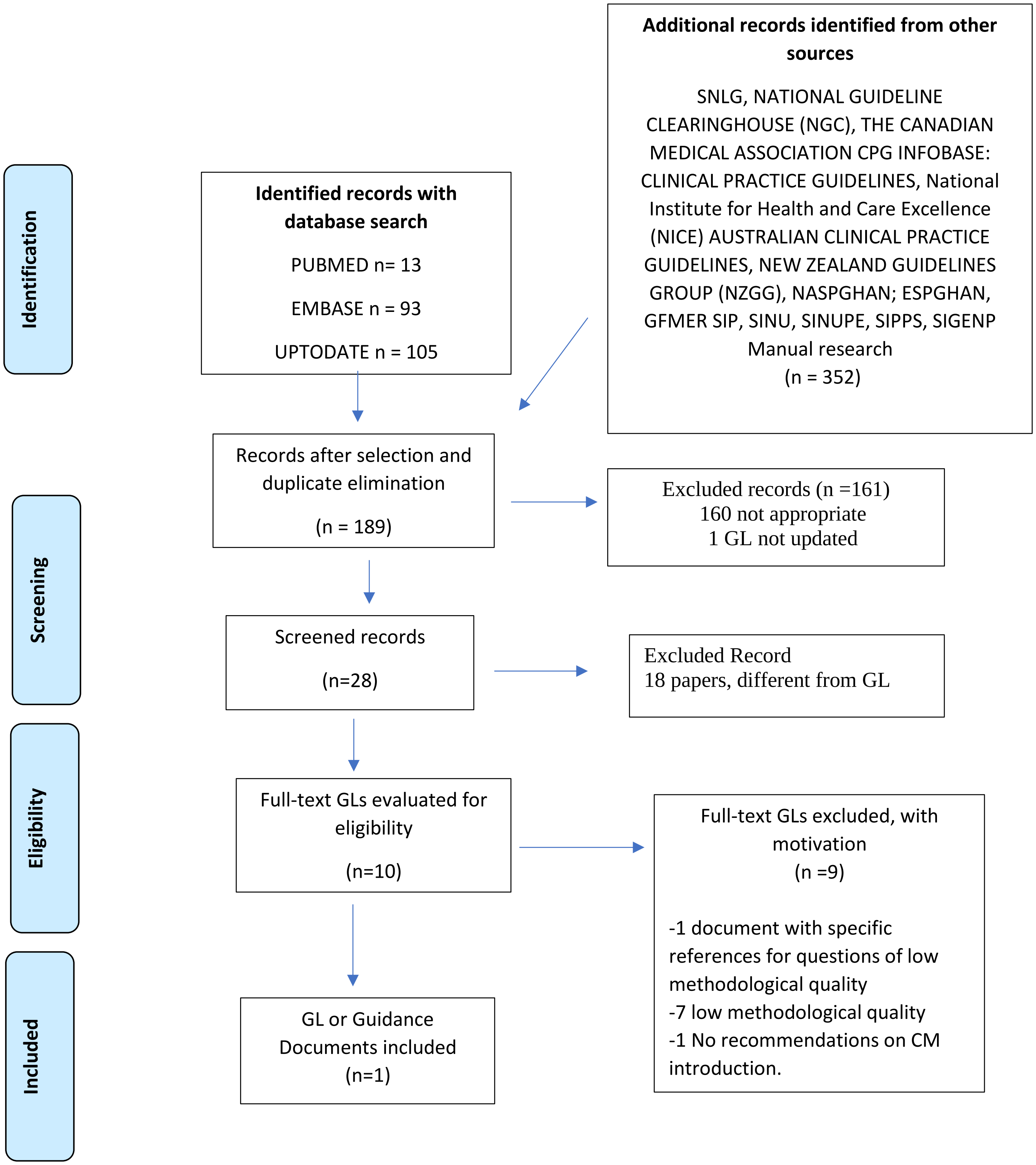

Figure a3.2. SRs search flow diagram.

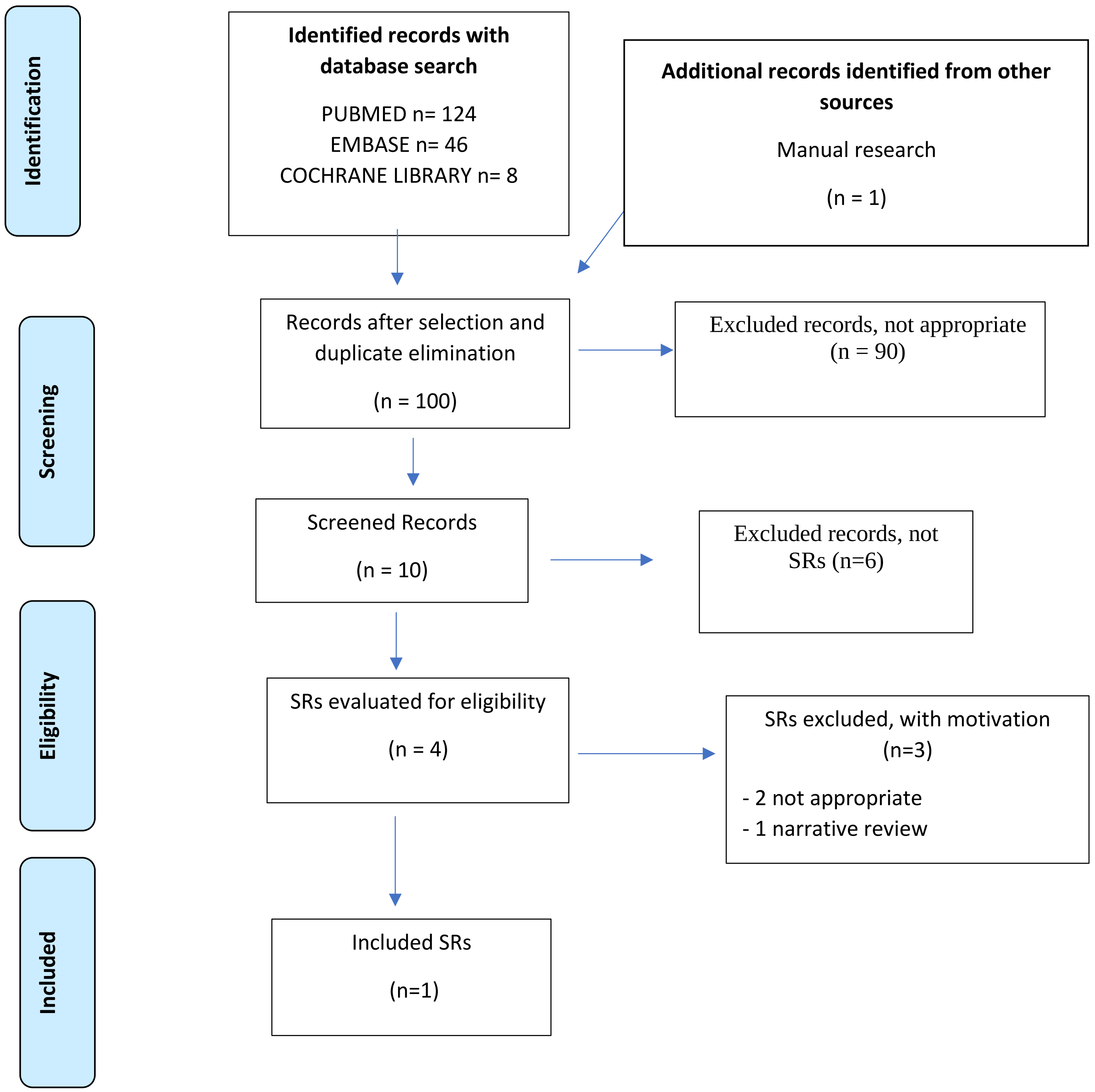

Figure a3.3. Studies search flow diagram.

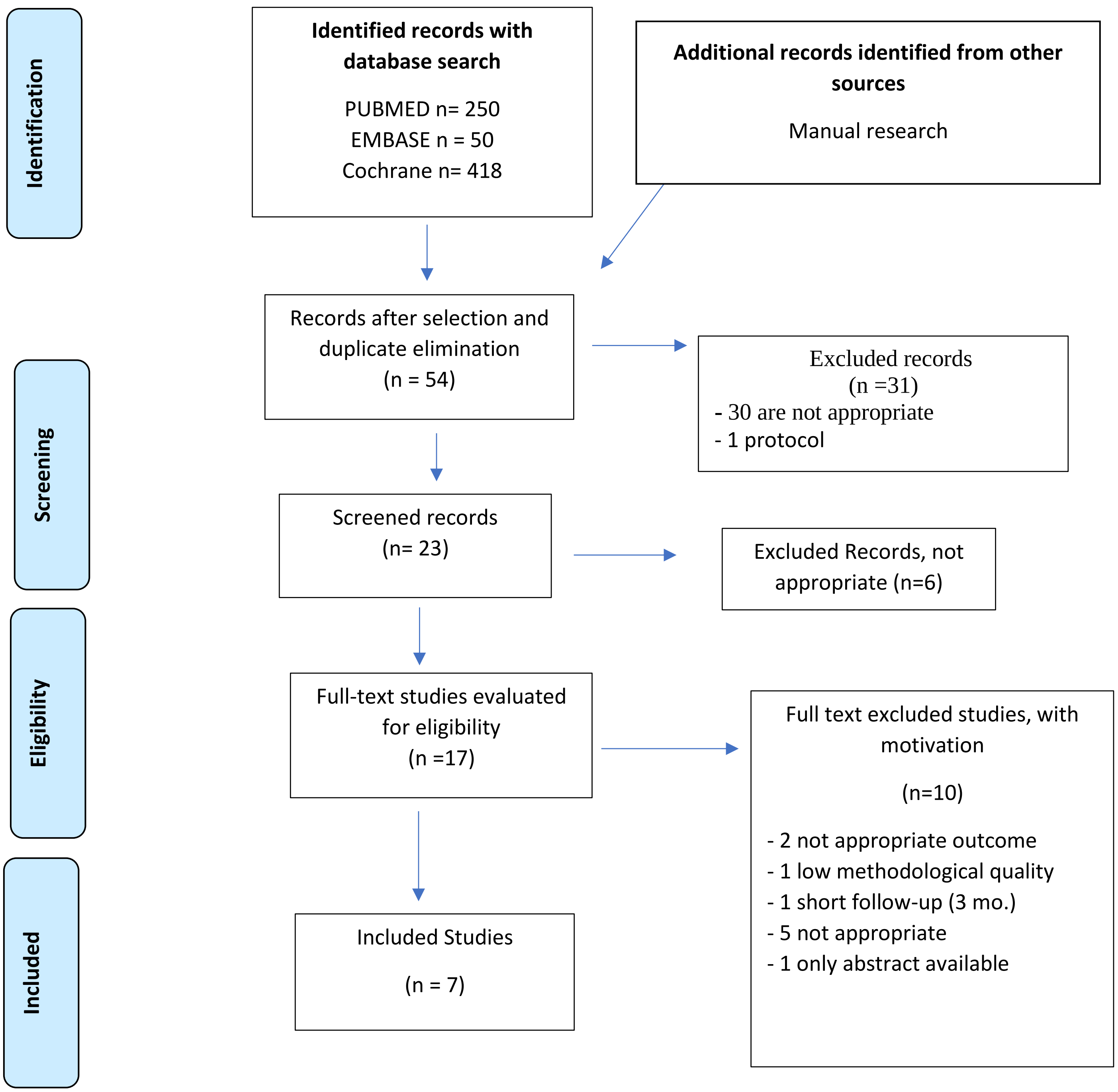

3. METHODOLOGICAL ASSESSMENT

Table a3.1. Appraisal of the Clinical Guidelines and Documents.

**Guidelines and Documents**	**Methodological Evaluation**			
	**Multidisciplinary panel**	**Systematic evidence research**	**Grading of recommendations**	**GL overall assessment**
EFSA 2013<br>Scientific opinion [1]	Yes	Yes<br>Explicit sources of Government Agencies and Organizations.	No<br>Narrative explanation of available data.	Moderate methodological quality.

Table a3.2. Clinical Guidelines and Documents excluded.

**GL Excluded**	**Multidisciplinary panel**	**Systematic evidence research**	**Grading of recommendations**	**Reason for exclusion**
Alvisi et al. 2015 [2]	Limited to Pediatricians and Nutritionists.	No	No	Review document, with recommendations for clinical guidance.<br>Low methodological quality.
Davanzo et al. 2015. Breastfeeding [3]	No	No	No	Low methodological quality.
Polfuss et al. 2020 Childhood Obesity Parte 1 [4]	No	Yes	No	Low methodological quality.
Davis et al. 2021 Childhood Obesity Parte 2 [5]	No	SI	No	Low methodological quality.
Romero-Velardea et al. 2016. Alimentation complementaria [6]	Limited to Pediatricians and Nutritionists.	No	No	Low methodological quality.
Fewtrell et al. 2017. ESPGHAN. Complementary feeding [7]	No	Declared but not published.	No	Low methodological quality.
Hojsak. ESPGHAN et al. 2018. Young Child Formula [8]	No	Declared but not published.	No	Low methodological quality.
Valerio Consensus SIEDP-SIP et al. 2017<br>Obesità [9]	Yes	No, only MEDLINE	Yes	No recommendations on CM introduction.

Koletzko et al. 2019 The Early Nutrition Project Recommendations [10]	Yes	Yes (Used previously published RS: for questions in this Consensus Agostoni et al. 2011.)	Consensus Vote.	Reference documents are outdated narrative reviews of low methodological quality.

Table a3.3. Appraisal of the Systematic Reviews.

AMSTAR 2	Griebler et al. 2015 [11]
**1. Did the research questions and inclusion criteria for the review include the components of PICO?** **(Yes/No)**	Yes
**2. Did the report of the review contain an explicit statement that the review methods were established before the conduct of the review and did the report justify any significant deviations from the protocol?** **(Yes/Partial Yes/No)**	Yes
**3. Did the review authors explain their selection of the study designs for inclusion in the review?** **(Yes/No)**	Yes
**4. Did the review authors use a comprehensive literature search strategy?** **(Yes/Partial Yes/No)**	Yes, Partial
**5. Did the review authors perform study selection in duplicate?** **(Yes/No)**	Yes
**6. Did the review authors perform data extraction in duplicate?** **(Yes/No)**	Yes
**7. Did the review authors provide a list of excluded studies and justify the exclusions?** **(Yes/Partial Yes/No)**	No

**8. Did the review authors describe the included studies in adequate detail?**<br><br>**(Yes/Partial Yes/No)**	Yes
**9. Did the review authors use a satisfactory technique for assessing the risk of bias (RoB) in individual studies that were included in the review?**<br><br>**(Yes/Partial Yes/No/Includes only NRSI-RCT)**	Yes, but not reported in detail.
**10. Did the review authors report on the sources of funding for the studies included in the review?**<br><br>**(Yes/No)**	No
**11. If meta-analysis was performed did the review authors use appropriate methods for statistical combination of results?**<br><br>**(Yes / No / No meta-analysis conducted)**	No, a combination of 4 studies with different designs.
**12. If meta-analysis was performed, did the review authors assess the potential impact of RoB in individual studies on the results of the meta-analysis or other evidence synthesis?**<br><br>**(Yes / No / No meta-analysis conducted)**	No
**13. Did the review authors account for RoB in individual studies when interpreting/ discussing the results of the review?**<br><br>**(Yes/No)**	Yes
**14. Did the review authors provide a satisfactory explanation for, and discussion of, any heterogeneity observed in the results of the review?**<br><br>**(Yes/No)**	Yes
**15. If they performed quantitative synthesis did the review authors carry out an adequate investigation of publication bias**	Yes

**(small study bias) and discuss its likely impact on the results of the review?**<br><br>**(Yes / No / No meta-analysis conducted)**	
**16. Did the review authors report any potential sources of conflict of interest, including any funding they received for conducting the review?**<br><br>**(Yes/No)**	Yes
**OVERALL EVALUATION**	**MODERATE QUALITY***<br><br>***** Overall opinion on RS excluding meta-analysis of 4 studies.

Table a3.4. SRs excluded with motivation.

SRs excluded	Reason for exclusion
EFSA 2019 [12]	No appropriate recommendations were reported.
Vanderhout et al. 2019 [13]	Not appropriate.
Verduci et al. 2019 [14]	Narrative review. Not appropriate.

Table a3.5. Appraisal of the Studies

a3.5.1.

	**Newcastle Quality Assessment Scale**<br>**COHORT STUDIES**								
	**Selection**				**Comparability**	**Outcome**			
**Study**	**Representativeness of the exposed cohort**	**Selection of the non exposed cohort**	**Ascertainment of exposure**	**Demonstration that outcome of interest was not present at the start of the study**	**Comparability of cohorts based on the design or analysis**	**Assessment of outcome**	**Was follow-up long enough for outcomes to occur**	**Adequacy of follow up of cohorts**	**Total**
Hopkins et al. 2015 [15]	1a	1a	1b	1a	2 a,b (adjusted for maternal education, maternal smoking in pregnancy, and parity)	1a	1a	0c (49 mo. =79.6%<br>7 aa=76.3%<br>10 aa= 69%	8/9
Lamb et al. 2015 [16]	1a	1a	1b	1a	2 a,b (Adjusted for total caloric intake, food frequency questionnaire type, family history of T1D, and ethnicity. Also adjusted for HLA-DR genotype.)	1a	1a	1b (73.4%)	9/9

a3.5.2.

	**Newcastle Quality Assessment Scale**<br>**CROSS-SECTIONAL STUDIES**							
	**Selection**				**Comparability**	**Outcome**		
**Study**	**Representativeness of the sample**	**Sample size:**	**Non-respondents**	**Ascertainment of the exposure (max 2)**	**Comparability between groups, confounders are controlled (Maximum 2 stars)**	**Outcome evaluation (max 2)**	**Statistical test**	**Total**
Ferrara et al. 2014 [17]	1a	0b	1a	1b	1a	2b	1a	**7/8**

a3.5.3.

Newcastle Quality Assessment Scale

CASE-CONTROL STUDIES

	**Selection**				**Comparability**	**Exposure**	
**Study**	**Adequate case definition**	**Case Representativeness**	**Selection of Controls (community)**	**Definition of Controls (no outcome)**	**Comparability of cases and controls based on the design or analysis.**	**Ascertainment of exposure**	**a** [illegible]
Villagran-Garcia et al. 2015 [18]	1a (DM1 diagnosis)	0 b (not shown)	1a	1a	2 a,b (DM1 and age controls)	1b (Structured and validated questionnaires administered by experienced staff)	[illegible]
Awadalla et al. 2017 [19]	1a (DM1 diagnosis)	1a (random selection.)	1a	1a	2a,b (DM1 and age controls)	1b (structured and validated questionaries)	[illegible]

RCT

Figure a3.4. Risk of bias summary: review authors' judgments about each risk of bias item for each included study. [20-21]

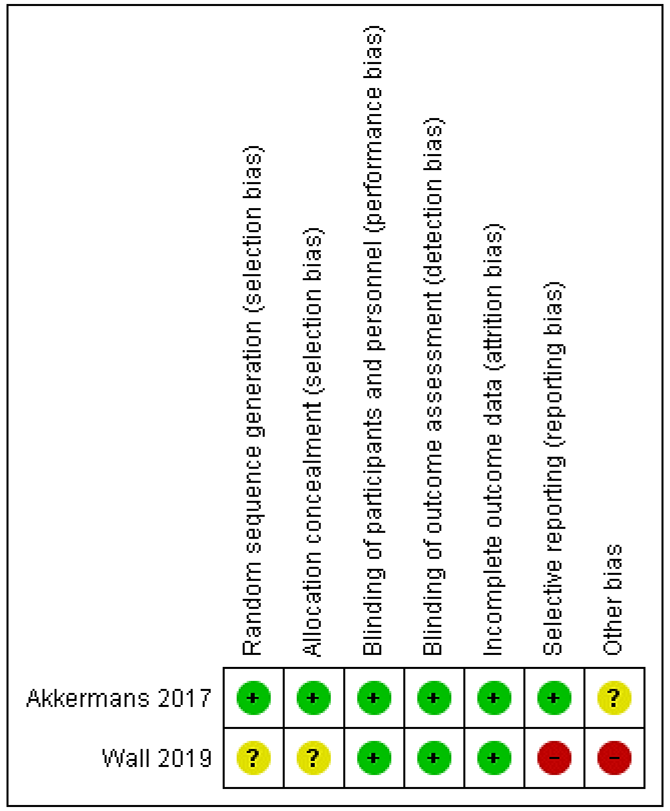

Figure a3.5. Risk of bias graph: review authors' judgments about each risk of bias item presented as percentages across all included studies.

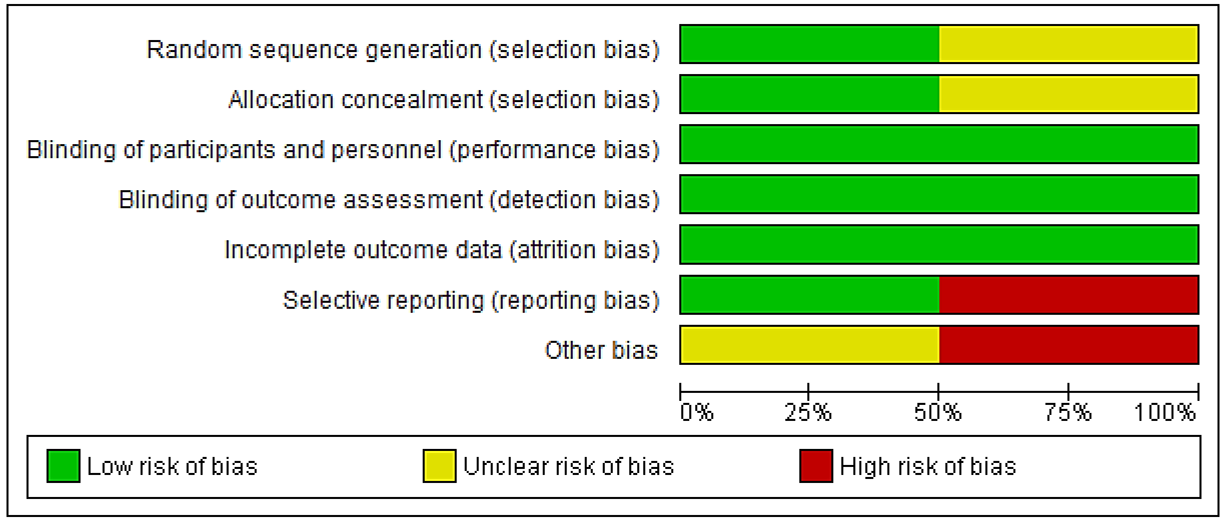

Table a3.6. Excluded studies with motivation.

Excluded studies	Reason for exclusion
Larnkjaer et al. 2009 [22]	Follow up too short for clinically relevant outcomes (3 mo).
Lovell et al. 2018 [23]	Not appropriate outcomes.
Lovell et al. 2019 [24]	Low methodological quality (post-hoc analysis of 83/160 randomized patients).
Socha et al. 2011 [25]	Not appropriate. Low and high protein formulas.
Urashima et al. 2019 [26]	Not appropriate. Outcome: APLV prevention.
Ghisolfi et al. 2013 [27]	Not appropriate (assesses nutrient intakes)
Parkin et al. 2016 [28]	Not appropriate (Evaluated the amount of CM intake, not the age of intake or comparison to the formula).
Saldan et al. 2017 [29]	Cross-sectional. Not appropriate (assesses socioeconomic factors promoting breastfeeding).
Soczynska et al. 2019 [30]	Only abstract available.
Szymlek-Gay et al. 2009 [31]	Not appropriate (Assesses only iron and not anemia. Sponsored by Heinz Wattie's New Zealand Ltd).

A3. RECOMMENDATIONS OF THE GLs, RESULTS OF THE SRs AND STUDIES

**a.**<br>- ***Does feeding with cow's milk before 12 months of age, compared to formula feeding, lead to different nutritional and metabolic outcomes in the short and long term?***	**P** In a healthy infant not fed with breast milk<br>**I** the feeding with unmodified cow's milk before the 12th month<br>**C** compared to formula intake<br>**O** results in different nutritional and metabolic outcomes in the short and long term?

Table a3.7. Included Guidelines and other Documents: Recommendations and Grading.

Guidelines – other Documents		Recommendations	Grading
EFSA 2013<br>Scientific opinion [1]		6.5<br>6,5,1<br>In the first year of life, follow-on formulas provide a safe alternative to breast milk. The use of the whole CM in large quantities is not recommended.	No

Table a3.8. Included SRs: Characteristics, Results, and Conclusions.

Systematic Review	Population and purpose of the SR	Results	Conclusions
Griebler et al. 2015 [11]<br>C.B.<br>July 2013	Effects of the introduction of animal milk before the age of 12 months on metabolism/nutritional status (intestinal blood loss, iron deficiency anemia, dehydration, obesity, osteoporosis, type 1 diabetes, growth retardation, gastrointestinal diseases, allergies such as eczema and asthma), compared to formula milk made according to EU guidelines.<br><br>Total of 2007 cases and 8455 controls.	23 included studies: 1 RCT, 4 nonrandomized controlled trials, 7 prospective cohort studies, 3 retrospective studies, 8 case-control studies.<br><br>Anemia: 9 studies including 1 RCT, 4 non-RCT, 2 prospective cohort studies, 2 retrospective studies<br><br>Diabetes type 1: 7 case-control studies<br><br>Asthma: 2 prospective cohort studies<br><br>Growth: 1 RCT, 1 prospective cohort study<br><br>Psychomotor development: 1 RCT<br><br>Atopic dermatitis: 3 prospective cohort studies<br><br>Intestinal blood loss: 3 RCTs, 1 prospective cohort study.	Anemia: 8 of 9 studies concluded that the introduction of CM before 12 months exposes to iron deficiency anemia.<br><br>The only study with contradictory data included children in CM or low Iron formula and all supplemented with iron. More anemic if CM < 6 months and lower ferritin for higher amounts of CM.<br><br>Diabetes: 6 studies concluded that the risk of diabetes does not increase. 1 study identified a lower risk.<br><br>Asthma: no increased risk (one study concluded that frequent rather than occasional intake had a protective effect).<br><br>Growth: RCT concluded that there was no significant difference in weight/age z-score, height/age, weight/height between the Cow Milk group and formula group. A prospective study identified an increase in BMI for high Cow Milk intake.<br><br>Psychomotor development: no difference.<br><br>Atopic dermatitis: inconclusive results.<br><br>Intestinal blood loss: no association.

Table a3.9. Included studies: Characteristics and Results.

Study	Study design	Population (sample size, baseline characteristics)	Intervention/exposure	Primary Outcome	Reported effect	Secondary Outcomes	Follow-up
Ferrara et al. 2014 [17]	Retrospective cross-sectional study.	1250 children aged 8 to 36 mo.. Examined period 1980-2010.	Evaluation of some determinants such as family economic status, weight/height, time of LV introduction, iron supplementation, time of complementary feeding beginning.	Assessment of iron metabolism and prevalence of iron deficiency anemia.			Positive association: Between low income (OR: 4; 95% CI: 1.16-0.04), age of CMP introduction <12 mo. (OR: 6.8; 95% CI: 1.55-0.11), age of weaning >6 mo. (OR:2.5; 95% CI: 0.694-0.106), lack of iron supplementation (OR:17; 95% CI: 1.63-0.83), overweight (OR 5.5; 95% CI: 0.85-0.55), and ID.
Hopkins et al. 2015 [15]	Prospective cohort study	1112 children (at 8 mo) were divided into three groups: 141 in the group with Breast Milk (BM), 824 in the Infant Formula (IF) group, and 147 Cow Milk (CM).	Three groups (of which two were divided into two subgroups) of children receiving BM or IF (< or > of 600ml/d) or CM (< or > of 600ml/d) at the age of 8 mo.	Evaluation of differences between energy intake and subsequent change in growth and BMI in infants breastfed with BM rather than low or high amounts of CM or IF.		From 8 mo up to 10 yrs.	Infants receiving >600 mL/day of CM had greater weight than breastfed infants from 8 mo to 10 yrs. The greatest difference in weight was at 18 mo (0.70 SDS; 95% CI: 0.41, 1.00 SDS; P = ,0.0001). They were also taller and had a higher BMI. Children on CM compared with those on BM receive more energy with a difference of 739 kJ/d (95% CI: 453, 1024 kJ/d; P = .001) at 8 mo. They took in more fat and more protein with a difference of 16.8 g (95% CI: 13.6- 19.9 g) at 8 mo.. The differences were maintained but were smaller for children consuming <600 mL CM/day.
Villagran-Garcia et al. 2015 [18]	Case-control study.	5 patients with diabetes and 75 controls.	Three groups of children receiving BM or CM or IF.	Association between early introduction of CM and type 1 diabetes.	Assessment of duration of breastfeeding in subjects with diabetes compared with controls (data inferred from outcome paragraph).	Up to 16 yrs old.	The risk of diabetes was nearly 4 times higher in infants who received CM before 12 mo. and after 6 mo. compared to breastfed infants. The risk is also high for subjects taking IF after 6 mo. but lower than for CM. The mean age of CM

							intake was shorter in subjects with diabetes (2.4 ± 2.2 mo) than in controls (3.1 ± 2.3 mo), but the difference was not statistically significant p=0.09.
Lamb et al. 2015 [16]	Prospective cohort study.	Approximately 2550 subjects (2607 from tab 1, + those with a family risk). Siblings and children of diabetic patients enrolled between birth and 8 yr. Children born between 1993 and 2006 screened on cord blood for HLA at risk. Lost 772 enrolled.	Assessment of CM introduction age, amount of protein, and lactose intake of CM.	In children with increased risk: the amount of milk increased the risk of developing pancreatic cell autoantibodies and subsequently diabetes.	- to evaluate if the age of CM introduction leads to increased consumption of CM and increases the risk of autoimmunity and diabetes.<br>- to evaluate whether the increased risk of autoimmunity and diabetes is greater in children taking more CM with low/moderate HLA-related risk.	Group enrolled at birth, at 9-15, and 24 mo.. Then also subsequently as for the second group with familiarity: once a year from 2 and 15 years (optional until 25aa).	The amount of CM protein consumed does not increase the risk of pancreatic cell autoimmunity or diabetes.<br>The amount of CM protein intake was related to an increased risk of autoimmunity in subjects with low-to-moderate risk of HLA-related diabetes.<br>Increased intake of CM protein is only slightly correlated with age of dietary CM introduction but this was related to an increased risk of developing autoimmunity.<br>The amount of protein intake related to an increased risk of developing diabetes in subjects with positive autoimmunity.<br>The risk of developing autoimmunity depends on the amount of CM protein intake but was HLA related, the risk of developing diabetes in subjects with autoimmunity depends on the amount of protein intake but was not HLA related.<br>Improving the definition of autoimmunity against pancreatic cells (68 pts vs 143 pts) there was no correlation with CM (amount, age of introduction) even according to HLA correlated risk.

Awadalla et al. 2017 [19]	Case-control study.	408 children were enrolled, of which 204 were diagnosed with T1DM and 204 controls.	Collection of medical history data with a questionnaire at the time of diabetes diagnosis.	Assessing environmental risk factors associated with the development of T1DM among children in Egypt.	Evaluation of the age of CM introduction as a risk factor for T1DM.	Prospective enrollment, data collected retrospectively.	The introduction of CM into children's diets represents a risk factor for T1DM (aOR=6.37, 95%CI:3.23-12. 58) From sex-adjusted logistic regression analysis on statistically significant maternal, neonatal, and child environmental factors predictive of DM1 only (residence, DM1 family history, mode of delivery, breastfeeding, vitamin D supplementation, physical activity): introduction of cow's milk in the first year of life (positive vs. negative) ORa= 19.94 (95%CI: 8.73-45.53).

b.<br>- ***Does feeding with unmodified cow's milk after 12 months of age, compared to growth formula, result in negative short- and long-term metabolic effects?***	**P** In healthy children aged 12 to 24 mo.<br>**I** the intake of unmodified cow's milk<br>**C** compared to formula (growth) feeding?<br>**O** results in different short- and long-term nutritional and metabolic outcomes?

Table a3.10. Included Guidelines and other Documents: Recommendations and Grading.

Guidelines – other Documents		Recommendations	Grading
EFSA 2013<br>Scientific opinion [1]		<u>6.5</u><br><u>6,5,1</u><br>After one year of age, although the use of follow-on formulas or YCG richer in DHA, iron, Vitamin D compared to whole LV is preferred, the use of LV is no longer discouraged.	No

Table a3.11. Included studies: Characteristics and Results.

Study (First Author, Year, Country/Setting)	Study design	Population (sample size, baseline characteristics)	Intervention/exposure	Primary Outcome	Reported effect	Secondary Outcomes	Follow-up
Akkermans et al. 2017 [20]	Double-blind randomized controlled trial.	318 children aged 12 to 36 mo, 158 in the active IF group and 160 in the control (CM) group.	Blood sampling at time zero and the end of 20 weeks. Follow-up visits at time 0, at 10 weeks, and the end.	To study the effect of a fortified IF administered for 20 weeks on ferritin concentrations of healthy children aged 12 to 36 mo. compared with the use of unfortified CM.	To evaluate the effect on the prevalence of iron deficiency anemia, iron, and vitamin D3 concentration.	20 weeks.	The differences between the two groups in the value of blood iron and 25(OH)D was 6.6 mg/L (95% CI: 1.4, 11.7 mg/L; P = 0.013) and 16.4 nmol/L (95% C 9.5, 21.4 nmol/L; P = 0.001) for the CM and IF fortified, respectively. The probability of anemia (OR= 0.42; 95% CI:0.18, 0.95; P = 0.036) and 25(OH)D deficiency (OR=0.22; 95% CI: 0.01, 0.51 = 0.001) was higher in the CM group.
Wall et al. 2019 [21]	Multicenter randomized controlled prospective double-blind study.	160 subjects were recruited in Auckland and Brisbane at 1 yr and randomized into 2 groups. Included 67/80 per group in the final analysis.	Unmodified CM or low-protein IF with added Iron, Vit D, and probiotics administered between 12 and 24 mo..	Effect at two years of age on body fat composition.	Effect at 24 mo. on anthropometric parameters.	From 12 to 24 mo of age.	Significant differences were observed in zL and L between the intervention and control groups at 6 mo but not at 12 mo.. There were no significant differences in weight, BMI, zBMI, and zP-L score a 6 mo. and 12 mo., but these measurements were consistently lower in the intervention group than in the control group at both time points.

3. EVIDENCE PROFILE GRADE

Table a3.12. Comparison: CM introduction before 12 months vs formula.

[CM introduction before 12 months] compared with [formula up to 12 months] for [different nutritional and metabolic outcomes, short- and long-term].

Patient or population: [different nutritional and metabolic outcomes, short- and long-term].

Setting: Outpatient.

Intervention: [CM introduction before 12 mo.].

Comparator: [formula up to 12 mo.].

Certainty assessment							Impact	Certainty of evidence	Importance
№ of studies	Study Design	Distortion risk	Lack of reproducibility of results	Lack of generalis ability	Inaccuracy	Further considerations			
**Weight**									

[CM introduction before 12 months] compared with [formula up to 12 months] for [different nutritional and metabolic outcomes, short- and long-term].

Patient or population: [different nutritional and metabolic outcomes, short- and long-term].

Setting: Outpatient.

Intervention: [CM introduction before 12 mo.].

Comparator: [formula up to 12 mo.].

Certainty assessment							Impact	Certainty of evidence	Importance
№ of studies	Study Design	Distortion risk	Lack of reproducibility of results	Lack of generalis ability	Inaccuracy	Further considerations			
1 [1]	randomized trials	serious [a]	not relevant	serious [b]	not relevant	none	100 children<br>No significant differences in weight/age z-score, height/age, and weight/height between the CM group and the formula group.	⊕⊕◯◯ LOW	CRITICAL

BMI

2 [2,3]	Observational studies	not relevant	serious [c]	serious [b]	not relevant	all plausible residual confounders could reduce the demonstrated effect.	1493 + 1112 children. Increased BMI for high CM intake.<br><br>Children consuming >600 mL/day of CM had higher weight than breastfed children from 8m to 10 yr. The greatest difference in weight was at 18 mo (0.70 SDS; 95% CI: 0.41, 1.00 SDS; P = ,0.0001). They were also taller and had a higher BMI.	⊕◯◯◯ VERY LOW	IMPORTANT

Anemia (evaluation with gr/dl)

10 [4,5]	Observational studies [d]	serious [e]	not relevant	not relevant	not relevant	strong association<br><br>all plausible residual confounders could reduce the demonstrated effect<br><br>dose-response gradient	9 of 10 studies conclude that introduction of CM before 12 mo exposes to iron deficiency anemia.<br><br>1 study: positive association with age of CM introduction <12 mo (OR: 6.8; 95% CI: 1.55-0.11).<br><br>The only study with conflicting data included children in CM or low-iron formula and all supplemented with iron. More anemia if CM < 6 mo. and lower ferritin for higher amounts of CM.	⊕⊕⊕⊕<br><br>HIGH	CRITICAL

Type 1 Diabetes Mellitus (evaluation with: blood sugar (mg/dl)

10 [5,6,7,8]	Observational studies	serious [f]	not relevant	not relevant	not relevant	all plausible residual confounders could reduce the demonstrated effect	16,001 children. 6 studies concluded no increased risk of diabetes. 1 study identified a low risk<br>1 study: introduction of cow's milk in the first year of life (positive vs. negative) ORa= 19.94 (95%CI: 8.73-45.53)<br>1 study: risk of diabetes was almost 4 times higher in children who took CM before 12 mo and after 6 mo than in breastfed children<br>1 study: risk of developing diabetes in subjects with autoimmunity depended on the amount of protein intake but was not HLA related. Improving the definition of autoimmunity against pancreatic cells (68 pcs vs 143) no correlation with CM was found.	⨁⨁◯◯ LOW	IMPORTANT

CI: Confidence interval

Explanations

a. Lack of blindness. No ITT analysis.

b. population composed largely of ethnic minorities and low socioeconomic level.

c. Discordant results with other studies.

d. 1 RCT, 4 nRCT, 2 observational cohort studies., 2 retrospectives

e. In 3 studies unadjusted for potential confounding factors, prognostic factors were not accurately measured in 2 studies.

f. In half of the studies, results were not adjusted for possible confounding factors. In all studies, prognostic factors were not reported.

References

1. 2015), Daly,1996 (in,Griebler) .

2. 2015), Wiley,2010 (in,Griebler) .

3. 2015, Hopkins.

4. 2014, Ferrara.

5. 2015, RS,Griebler.

6. 2015, Lamb.

7. 2017, Awadalla.

8. 2015, Villagran-Garcia.

Table a3.13. Comparison: CM introduction after 12 months vs. YCF formula.

[CM introduction after 12 mo] compared with [YCF formula] for [different nutritional and metabolic outcomes, short- and long-term]

Patient or population: [different nutritional and metabolic outcomes, short- and long-term].

Setting: Outpatient.

Intervention: [CM introduction before 12 mo.].

Comparison: [YCF]

Certainty assessment							Impact				Certainty of evidence	Importance
№ of studies	Study Design	Distortion risk	Lack of reproducibility of results	Lack of generalisability	Inaccuracy	Further considerations						
**Length (follow up: 12 mo.; evaluated with: cm)**												
1 [1]	randomized trials	Not relevant	serious [a]	Not relevant	Not relevant	none	67	67	**Adjusted difference -0.40** (-0.96 a 0.17)	**-- for 1.000** (from -- to --)	⊕⊕⊕◯ MODERATE	CRITICAL

Certainty assessment							Impact	Certainty of evidence	Importance
№ of studies	Study Design	Distortion risk	Lack of reproducibility of results	Lack of generalisability	Inaccuracy	Further considerations			

Weight (follow up: 12 mo.; evaluated with: Kg)

1 [1]	randomized trials	Not relevant	serious [a]	not relevant	not relevant	none	73	70	**Adjusted difference -0.19** (-0.04 a 0.03)	**-- for 1.000** (from -- to --)	⊕⊕⊕◯ MODERATE	CRITICAL

BMI (follow up: 12 mo.)

1 [1]	randomized trials	Not relevant	serious [a]	not relevant	not relevant	none	67	67	**Adjusted difference -0.14** (-0.44 a 0.16)	**-- for 1.000** (from -- to --)	⊕⊕⊕◯ MODERATE	IMPORTANT

zBMI (follow up: 12 mo.)

Certainty assessment							Impact	Certainty of evidence	Importance
№ of studies	Study Design	Distortion risk	Lack of reproducibility of results	Lack of generalisability	Inaccuracy	Further considerations			

1 [1]	randomized trials	Not relevant	serious [a]	not relevant	not relevant	none	67	67	**Adjusted difference -0.11** (-0.32 a 0.10)	**-- for 1.000** (from -- to --)	⊕⊕⊕◯ MODERATE	IMPORTANT

Iron deficiency anemia (follow up: 12 mo.; evaluation with gr/dl)

1 [2]	randomized trials	Not relevant	serious [a]	Not relevant	Not relevant	none [a]	158	160	**OR 0.42** (0.18 a 0.95)	**0 minus per 1.000** (from 0 minus to 0 minus)	⊕⊕⊕◯ MODERATE	IMPORTANT

CI: Confidence interval; **OR:** Odds ratio

Explanations

a. single study.

References

1. 2019, Wall.

2. 2017, Akkermans.

Appendix 3. References

1. EFSA NDA Panel. Scientific Opinion on nutrient requirements and dietary intakes of infants and young children in the European Union. EFSA Journal 2013;11(10):3408
2. Alvisi P, Brusa S, Alboresi S, et al. Recommendations on complementary feeding for healthy, full-term infants. Ital J Pediatr. 2015 Apr 28;41:36.
3. Davanzo R, Romagnoli C, and Corsello G. Position Statement on Breastfeeding from the Italian Pediatric Societies. Ital J Pediatr. 2015; 41: 80.
4. Polfuss ML, Duderstadt KG, Kilanowski JF, et al. Childhood Obesity: Evidence-Based Guidelines for Clinical Practice-Part One. J Pediatr Health Care. 2020 May-Jun;34(3):283-290
5. Davis R.L., Quinn M., Thompson M.E., et al. Childhood Obesity: Evidence-Based Guidelines for Clinical Practice—Part Two. Journal of Pediatric Health Care 2021 35:1 (120-131)
6. Romero-Velardea E, Villalpando-Carrión S, Pérez-Lizaur AB, et al. Consenso para las prácticas de alimentación complementaria en lactantes sanos. Bol Med Hosp Infant Mex. 2016;73(5):338---356
7. Fewtrell M, Bronsky J, Campoy C, et al. Complementary Feeding: A Position Paper by the European Society for Paediatric Gastroenterology, Hepatology, and Nutrition (ESPGHAN). Committee on Nutrition. J Pediatr Gastroenterol Nutr. 2017 Jan;64(1):119-132
8. Hojsak I, Bronsky J, Campoy C, et al. Young Child Formula: A Position Paper by the ESPGHAN Committee on Nutrition. JPGN 2018;66: 177–185
9. Valerio G, Saggese G, Maffeis C, et al. Diagnosi, trattamento e prevenzione dell'obesità del bambino e dell'adolescente. http://www.siedp.it/files/Doc.ConsensusObesita__2017.pdf
10. Koletzko B, Godfrey KM, Poston L, et al. Nutrition During Pregnancy, Lactation and Early Childhood and its Implications for Maternal and Long-Term Child Health: The Early Nutrition Project Recommendations. Ann Nutr Metab. 2019;74(2):93-106. doi:10.1159/000496471
11. Griebler U, Bruckmüller MU, Kien C, et al. Health effects of cow's milk consumption in infants up to 3 years of age: a systematic review and meta-analysis. Public Health Nutrition. 2016;19(2):293-307
12. EFSA Panel on Nutrition, Novel Foods, and Food Allergens (NDA). Appropriate age range for introduction of complementary feeding into an infant's diet. EFSA Journal 2019;17(9):5780
13. Vanderhout SM, Aglipay M, Torabi N, et al. Whole milk compared with reduced-fat milk and childhood overweight: a systematic review and meta-analysis. Am J Clin Nutr. 2019 Dec. pii: nqz276. doi: 10.1093/ajcn/nqz276.
14. Verduci E. D'Elios S, Cerrato L, et al. Cow's Milk Substitutes for Children: Nutritional Aspects of Milk from Different Mammalian Species, Special Formula and Plant-Based Beverages. Nutrients 2019, 11, 1739
15. Hopkins D, Steer CD, Northstone K, et al. Effects on childhood body habitus of feeding large volumes of cow or formula milk compared with breastfeeding in the latter part of infancy. Am J Clin Nutr. 2015;102:1096-103
16. Lamb MM, Miller M, Seifert JA, et al. The Effect of Childhood Cow's Milk Intake and HLA-DR Genotype on Risk of Islet Autoimmunity and Type 1 Diabetes: The Diabetes Autoimmunity Study in the Young (DAISY). Pediatr Diabetes. 2015;16(1):31-8
17. Ferrara M, Bertocco F, Ricciardi A, et al. Iron deficiency screening in the first three years of life: a three-decade-long retrospective case study. Hematology. 2014;19(4):239-43
18. Villagrán-García EF, Hurtado-López EF, Vasquez-Garibay EM, et al. Introduction of pasteurized/raw cow's milk during the second semester of life as a risk factor of type 1 diabetes mellitus in school children and adolescents. Nutr Hosp. 2015;32(2):634-7
19. Awadalla NJ, Hegazy AA, Abd El-Salam M, et al. Environmental Factors Associated with Type 1 Diabetes Development: A Case Control Study in Egypt. Int J Environ Res Public Health. 2017;14:615

20. Akkermans MD, Eussen SRBM, Van Der Horst-Graat JM, et al. A micronutrient-fortified young-child formula improves the iron and vitamin D status of healthy young European children: a randomized, double-blind controlled trial. Am J Clin Nutr. 2017;105(2):391-9
21. Wall CR, Hill RJ, Lovell AL, et al. A multicenter, double-blind, randomized, placebo-controlled trial to evaluate the effect of consuming Growing Up Milk “Lite” on body composition in children aged 12-23 mo. Am J Clin Nutr. 2019;109:576-85
22. Larnkjaer A, Hoppe C, Mølgaard C, Michaelsen KF. The effects of whole milk and infant formula on growth and IGF-I in late infancy. Eur J Clin Nutr. 2009;63(8):956-963
23. Lovell AL, Davies PS, Hill RJ, et al. A comparison of the effect of a Growing Up Milk Lite versus Cow's Milk on longitudinal dietary patterns and nutrient intakes in children aged 12 to 23 months: the Growing Up Milk Lite (GUMLi) randomized controlled trial. Br J Nutr. 2018 Dec 26:1-23
24. Lovell AL, Milne T, Jiang Y, et al. Evaluation of the Effect of a Growing up Milk Lite vs. Cow's Milk on Diet Quality and Dietary Intakes in Early Childhood: The Growing up Milk Lite (GUMLi) Randomised Controlled Trial. Nutrients. 2019 Jan 20;11(1).
25. Socha P, Grote V, Gruszfeld D, et al. Milk protein intake, the metabolic-endocrine response, and growth in infancy: data from a randomized clinical trial. Milk protein intake, the metabolic-endocrine response, and growth in infancy: data from a randomized clinical trial.
26. Urashima M, Mezawa H, Okuyama M, Urashima T, Hirano D, Gocho N, Tachimoto H. Primary Prevention of Cow's Milk Sensitization and Food Allergy by Avoiding Supplementation With Cow's Milk Formula at Birth: A Randomized Clinical Trial. JAMA Pediatr. 2019 Dec 1;173(12):1137-1145.
27. Ghisolfi J, Fantino M, Turck D, et al. Nutrient intakes of children aged 1-2 years as a function of milk consumption, cows' milk or growing-up milk. Public Health Nutr. 2013 Mar;16(3):524-34. doi: 10.1017/S1368980012002893. Epub 2012 Jul 4.
28. Parkin PC, DeGroot J, Maguire JL, et al. Severe iron-deficiency anaemia and feeding practices in young children. Public Health Nutrition: 19(4), 716–722
29. Chuproski Saldana P, Venancio SI, Dias Medici Saldiva SR. Et al. Milk consumption in infants under one year of age and variables associated with non-maternal milk consumption. Rev Paul Pediatr. 2017;35(4):407-414
30. Soczynska I, Maguire J, Birken C, et al. Age of introduction to cow milk and childhood growth. Paediatrics & Child Health, Vol. 24, No. S2 June 2019
31. Szymlek-Gay EA, Ferguson EL, Heath AL, et al. Food-based strategies improve iron status in toddlers: a randomized controlled trial 12. Am J Clin Nutr 2009;90:1541–5
