# Supplementary material for: Recommendations on Complementary Feeding as a Tool for Prevention of Non-Communicable Diseases (NCDs)—Paper Co-Drafted by the SIPPS, FIMP, SIDOHaD, and SINUPE Joint Working Group
Source: Nutrients. 2022 Jan 7;14(2):257. doi: 10.3390/nu14020257 (PMC8778394; doi:10.3390/nu14020257)
Supplement: Supplementary file 1 [file nutrients-14-00257-s001.zip › nutrients-1513088-supplementary/Supplementary File S4.pdf]

APPENDIX 4.

Recommendations 19-31

A4. BLW/BLISS and growth

Key questions

-Can using the Baby-Led Weaning (BLW; Self-weaning) method during the Complementary Feeding (AC) period influence, in a positive or negative way, the physical growth process in later ages?

PICOs

A.

P. In a healthy child in the second half of life
I. Baby-Led Weaning method
C. Compared to other feeding models
O. does it involve a different physical growth in later ages?

KEY WORDS
Population

A. No age limit

B. ([infant]/lim OR [child]/lim OR [preschool]/lim

Exposure Factors / Comparison

"self-weaning"[All Fields]
"self weaning"[All Fields]
"baby led weaning"[All Fields]
"baby-led weaning"[All Fields]))
“Infant Nutritional Physiological Phenomena” [MeSH]
“Weaning"[MeSH])

Outcomes

"Growth and Development"[Mesh]
"Growth"[Mesh])
"Growth Charts"[Mesh])
"Body Height"[Mesh])
"Body Weight"[Mesh])
"Body-Weight Trajectory"[Mesh])

"Body Weight Changes"[Mesh]

Guidelines search

PubMed

#1
("Growth and Development"[Mesh] OR "Growth"[Mesh]) OR "Growth Charts"[Mesh] AND ((Practice Guideline[ptyp] OR Guideline[ptyp]) AND "2014/08/11"[PDat] : "2021/03/15"[PDat] AND "humans"[MeSH Terms])

EMBASE

#1
('growth'/exp OR 'growth, development and aging'/exp) AND ('baby led weaning'/exp OR 'baby led weaning' OR 'baby led' OR 'self weaning' OR autoweaning AND ([adolescent]/lim OR [child]/lim OR [infant]/lim OR [preschool]/lim OR [school]/lim OR [young adult]/lim) AND [2014-2021]/py AND 'practice guideline'/de

UPTODATE https://www.uptodate.com/home

Society Guideline Links: *Breastfeeding and infant nutrition*

SOCIETY GUIDELINE LINKS:

National Guideline Clearinghouse (NGC) https://www.ahrq.gov/gam/index.html

Canadians Medical Association (CMA) https://www.cma.ca/clinicalresources/practiceguidelines

National Guideline Centre (NGC) - National Institute of Health and Care Excellence (NICE) https://www.rcplondon.ac.uk/about-us/what-we-do/national-guideline-centre-ngc

Scottish Intercollegiate Guidelines Network (SIGN) https://www.sign.ac.uk/our-guidelines.html

Australian Clinical Practice Guidelines (ACPG) https://www.clinicalguidelines.gov.au/

New Zealand Guidelines Group (NZGG) https://www.health.govt.nz/about-ministry/ministry-health-websites/new-zealand-guidelines-group

American Academy of Pediatrics (AAP) https://www.aap.org/en-us/Pages/Default.aspx

EPA/UNEPSA http://www.epa-unepsa.org/

Guidelines International Network https://g-i-n.net/

Società Italiana di Pediatria (SIP) http://www-sip.it/

Società Italiana di Pediatria Preventiva e Sociale (SIPPS) https://www.sipps.it/

Società Italiana di Endocrinologia e Diabetologia Pediatrica (SIEDP)
http://www.siedp.it/pagina/84/linee+guida%2C+raccomandazioni+e+consensus

Systematic Reviews search

COCHRANE LIBRARY

A. Growth
B. “Child Health/Develop-psych-learn problems” in Title Abstract Keyword
C. “Endocrine & Metabolic” in Title Abstract Keyword
C. “Weaning”
D. “Complementary feeding”
E. “Baby Led Weaning”
F.

Custom date range Topics: **01.01.2014 - 15.03.2021**

PubMed

#1

("self-weaning"[All Fields] OR "self weaning"[All Fields] OR "baby led weaning"[All Fields] OR "baby-led weaning"[All Fields]) **AND** ((((("Growth and Development"[Mesh] OR "Growth"[Mesh]) OR "Growth Charts"[Mesh]) OR "Body Weight Changes"[Mesh]) OR "Body Height"[Mesh]) OR "Body Weight"[Mesh]) OR "Body-Weight Trajectory"[Mesh]) **AND** ((Meta-Analysis[ptyp] OR systematic[sb]) **AND** ("2014/08/13"[PDat] : "2021/03/15"[PDat])

EMBASE

#1

'baby led weaning'/exp OR 'baby led weaning' OR 'baby led' OR 'self weaning' OR autoweaning AND ('growth'/exp OR 'growth, development and aging'/exp OR 'body weight'/exp OR 'body mass'/exp) AND [2014-2021]/py AND ([cochrane review]/lim OR [systematic review]/lim OR [meta analysis]/lim)

Studies search (subsequent to SR of D’Auria et al - 03/2018)

Cochrane al 15-03-21

'baby led weaning'/exp OR 'baby led weaning' OR 'baby led' OR 'self weaning' OR autoweaning in Title Abstract Keyword

PubMed

#1

("self-weaning"[All Fields] OR "self weaning"[All Fields] OR "baby led weaning"[All Fields] OR "baby-led weaning"[All Fields])) **AND** ((((((("Growth and Development"[Mesh]) OR "Growth"[Mesh]) OR "Growth Charts"[Mesh]) OR "Body Height"[Mesh]) OR "Body-Weight Trajectory"[Mesh]) OR "Body Weight Changes"[Mesh]) AND (Clinical Trial[ptyp] OR Comparative Study[ptyp] OR Multicenter Study[ptyp] OR Observational Study[ptyp] OR Pragmatic Clinical Trial[ptyp] OR Randomized Controlled Trial[ptyp]) AND ("2018/03/01"[PDAT]:"2021/03/15"[PDAT])

EMBASE

#1

'baby led weaning'/exp OR 'baby led weaning' OR 'baby led' OR 'self weaning' OR autoweaning AND ('growth'/exp OR 'growth, development and aging'/exp) AND [2018-2021]/py AND ('case control study'/de OR 'clinical trial'/de OR 'cohort analysis'/de OR 'comparative effectiveness'/de OR 'comparative study'/de OR 'controlled clinical trial'/de OR 'controlled study'/de OR 'cross-sectional study'/de OR 'double blind procedure'/de OR 'human'/de OR 'longitudinal study'/de OR 'multicenter study'/de OR 'observational study'/de OR 'prospective study'/de OR 'randomized controlled trial'/de OR 'retrospective study'/de) AND ([child]/lim OR [infant]/lim OR [preschool]/lim)

Figure a4.1. Guidelines search flow diagram

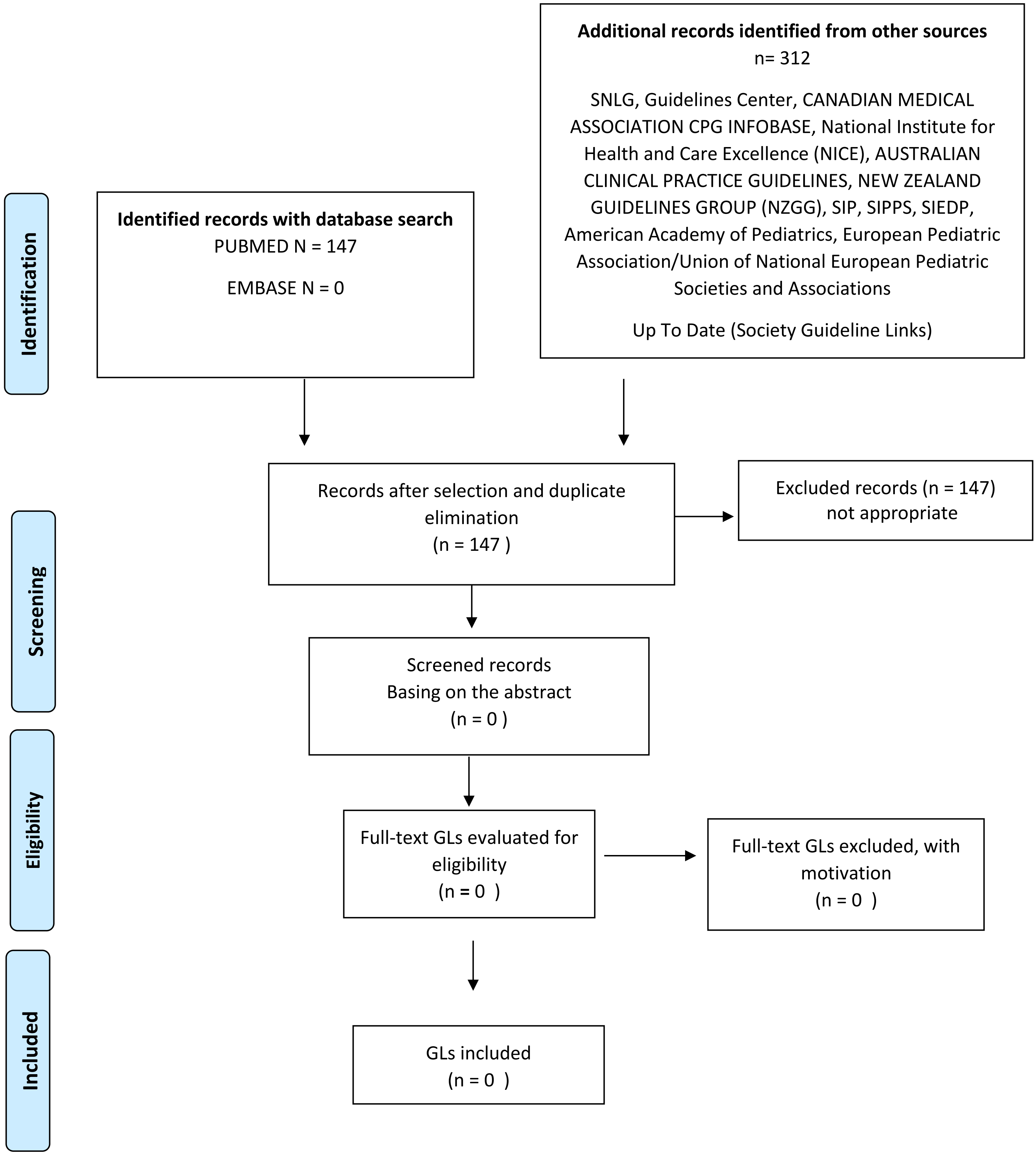

Figure a4.2. SRs search flow diagram

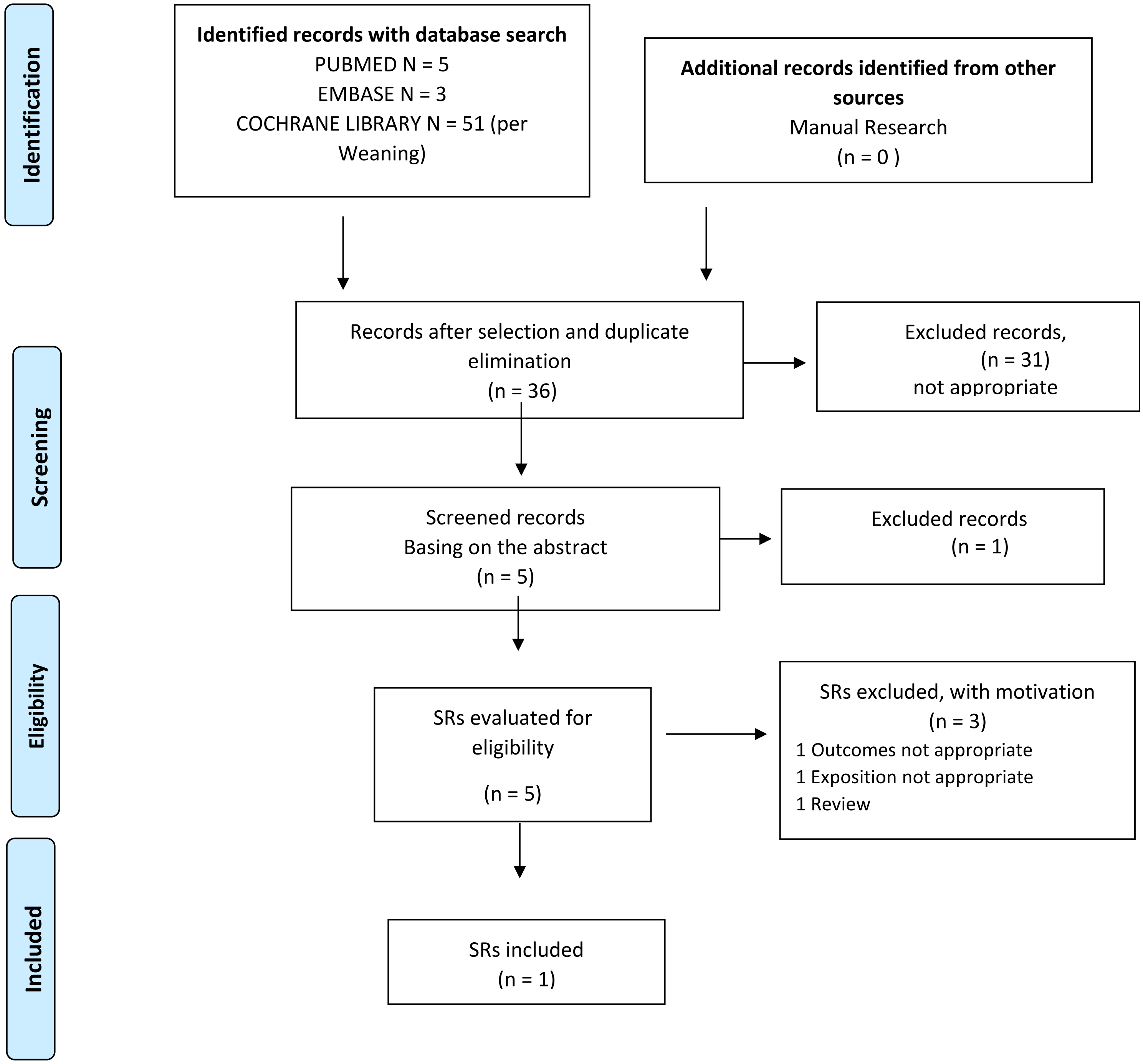

Figure a4.3. Studies search flow diagram.

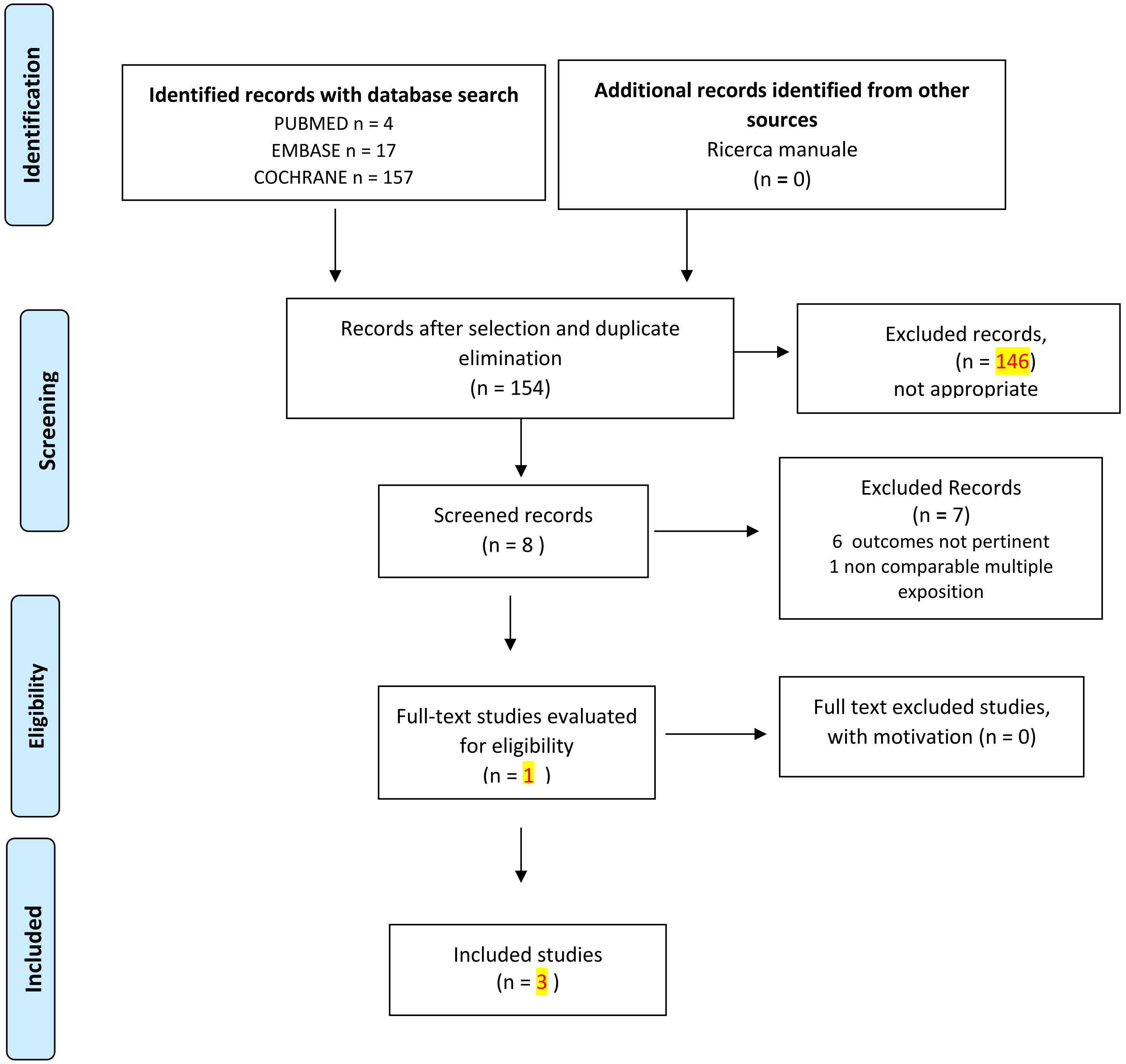

4a. BLW/BLISS and risk of overweight/obesity

- Can the BLW / BLISS method during AC influence, in a positive or negative way, the development of overweight / obesity in later ages?

PICOs

P. A healthy children in the second semester of life
I. the Baby-Led Weaning (or the BLISS method)
C. compared to other model of feeding
O. does it involve a different risk of overweight/obesity in later ages?

KEY WORDS

Population

G. No age limit

H. ([infant]/lim OR [child]/lim OR [preschool]/lim

Exposure Factors / Comparison

"self-weaning"[All Fields]
"self weaning"[All Fields]
"baby led weaning"[All Fields]
"baby-led weaning"

Outcomes

"Body Height"[Mesh])
"Body Weight"[Mesh])
"Body-Weight Trajectory"[Mesh])
"Body Weight Changes"[Mesh]
"Body Composition"[Mesh])
"Nutritional Status"[Mesh]
("Obesity"[Mesh]
"Pediatric Obesity"[Mesh])
"Overweight"[Mesh]

Guidelines search

SOCIETY GUIDELINE LINKS:

National Guideline Clearinghouse (NGC) https://www.ahrq.gov/gam/index.html

Canadians Medical Association (CMA) https://www.cma.ca/clinicalresources/practiceguidelines

National Guideline Centre (NGC) - National Institute of Health and Care Excellence (NICE) https://www.rcplondon.ac.uk/about-us/what-we-do/national-guideline-centre-ngc

Scottish Intercollegiate Guidelines Network (SIGN) https://www.sign.ac.uk/our-guidelines.html

Australian Clinical Practice Guidelines (ACPG) https://www.clinicalguidelines.gov.au/

New Zealand Guidelines Group (NZGG) https://www.health.govt.nz/about-ministry/ministry-health-websites/new-zealand-guidelines-group

American Academy of Pediatrics (AAP) https://www.aap.org/en-us/Pages/Default.aspx

EPA/UNEPSA http://www.epa-unepsa.org/

Guidelines International Network https://g-i-n.net/

European Childhood Obesity Group https://www.ecog-obesity.eu/

Società Italiana di Pediatria (SIP) http://www-sip.it/

Società Italiana di Pediatria Preventiva e Sociale (SIPPS) https://www.sipps.it/

Società Italiana di Endocrinologia e Diabetologia Pediatrica (SIEDP) http://www.siedp.it/pagina/84/linee+guida%2C+raccomandazioni+e+consensus

PUBMED

#1

("self-weaning"[All Fields] OR "self weaning"[All Fields] OR "baby led weaning"[All Fields] OR "baby-led weaning"[All Fields]) AND ((Practice Guideline[ptyp] OR Guideline[ptyp]) AND ("2014/10/02"[PDat] : "2021/03/15"[PDat])

EMBASE

#1

('baby led weaning'/exp OR 'baby led weaning' OR 'baby led' OR 'self weaning' OR autoweaning) AND ('obesity'/exp OR 'body mass'/exp OR overweight) AND [2014-2021]/py

Sistematic Reviews search

COCHRANE LIBRARY

“Endocrine & Metabolic” in Title Abstract Keyword
“Weaning”

“Baby Led Weaning”
'baby-led weaning or BLISS’ in Title Abstract Keyword'
Custom date range Topics: 01.09.2014-15.03.2021

PUBMED

#1

("self-weaning"[All Fields] OR "self weaning"[All Fields] OR "baby led weaning"[All Fields] OR "baby-led weaning"[All Fields]) AND ((Meta-Analysis[ptyp] OR systematic[sb]) AND "2014/10/02"[PDat] : "**2021/03/15**"[PDat])

EMBASE

#1

('baby led weaning'/exp OR 'baby led weaning' OR 'baby led' OR 'self weaning' OR autoweaning) AND ([systematic review]/lim OR [meta analysis]/lim) AND [2014-2021]/py

Primary Studies search (post-RS di D’Auria 2018)

PUBMED

#1

"self-weaning"[All Fields] OR "self weaning"[All Fields] OR "baby led weaning"[All Fields] OR "baby-led weaning"[All Fields] AND ((((((("Obesity"[Mesh] OR "Pediatric Obesity"[Mesh]) OR "Overweight"[Mesh]) OR "Body Mass Index"[Mesh]) OR "Body Weight Changes"[Mesh]) OR "Body Weight"[Mesh]) OR "Body Composition"[Mesh]) OR "Nutritional Status"[Mesh]) AND ("2018/03/01"[PDAT] : "**2020/03/15**"[PDAT])

EMBASE

#1

('baby led weaning'/exp OR 'baby led weaning' OR 'baby led' OR 'self weaning' OR autoweaning) AND ('obesity'/exp OR 'body mass'/exp OR overweight) AND ('clinical trial'/de OR 'comparative study'/de OR 'controlled clinical trial'/de OR 'controlled study'/de OR 'cross-sectional study'/de OR 'observational study'/de OR 'prospective study'/de OR 'randomized controlled trial'/de OR 'randomized controlled trial (topic)'/de) AND [2018-2021]/py

Figure a4.4. Guidelines search flow diagram

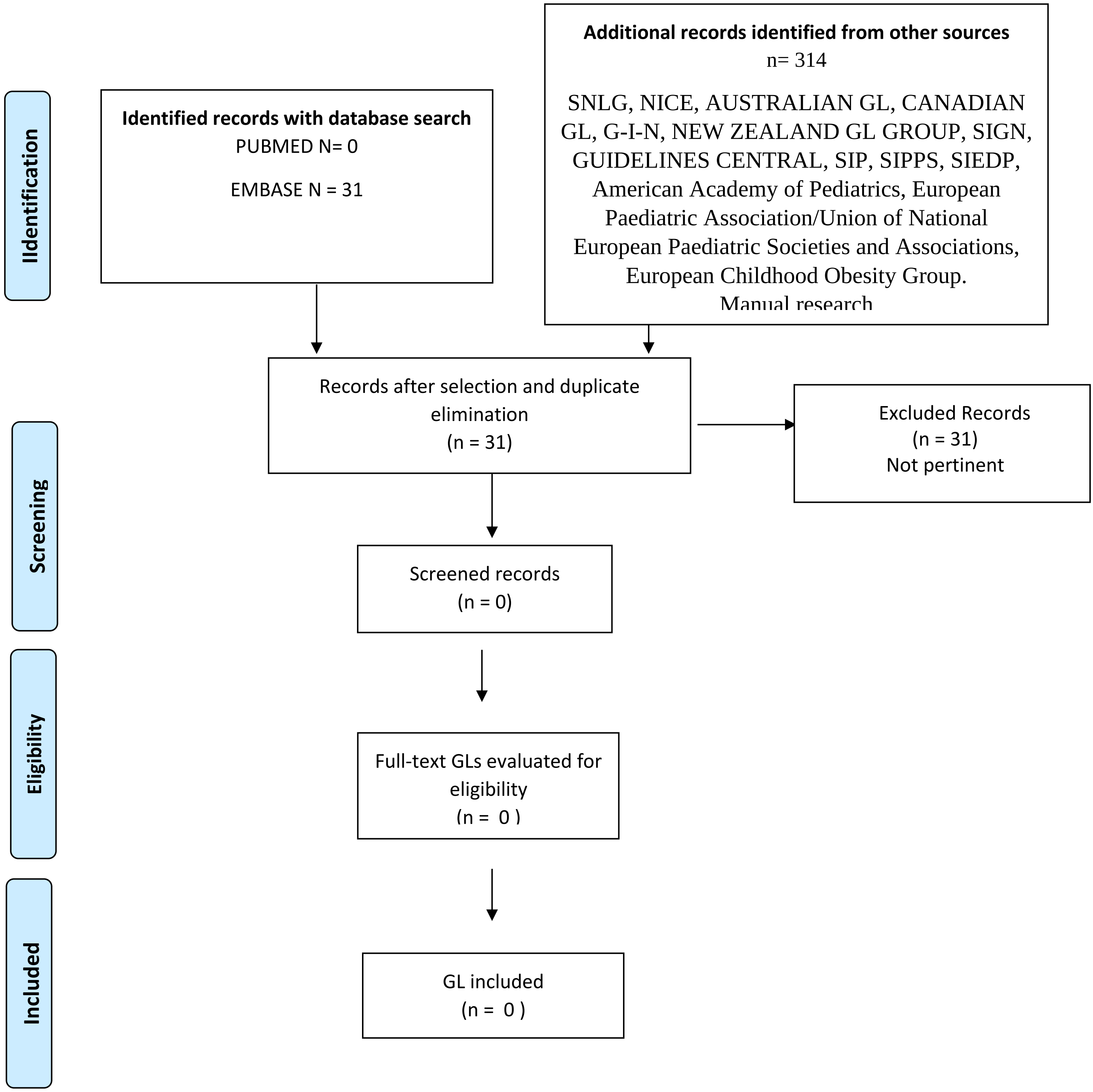

Figure a4.5. SRs search flow diagram

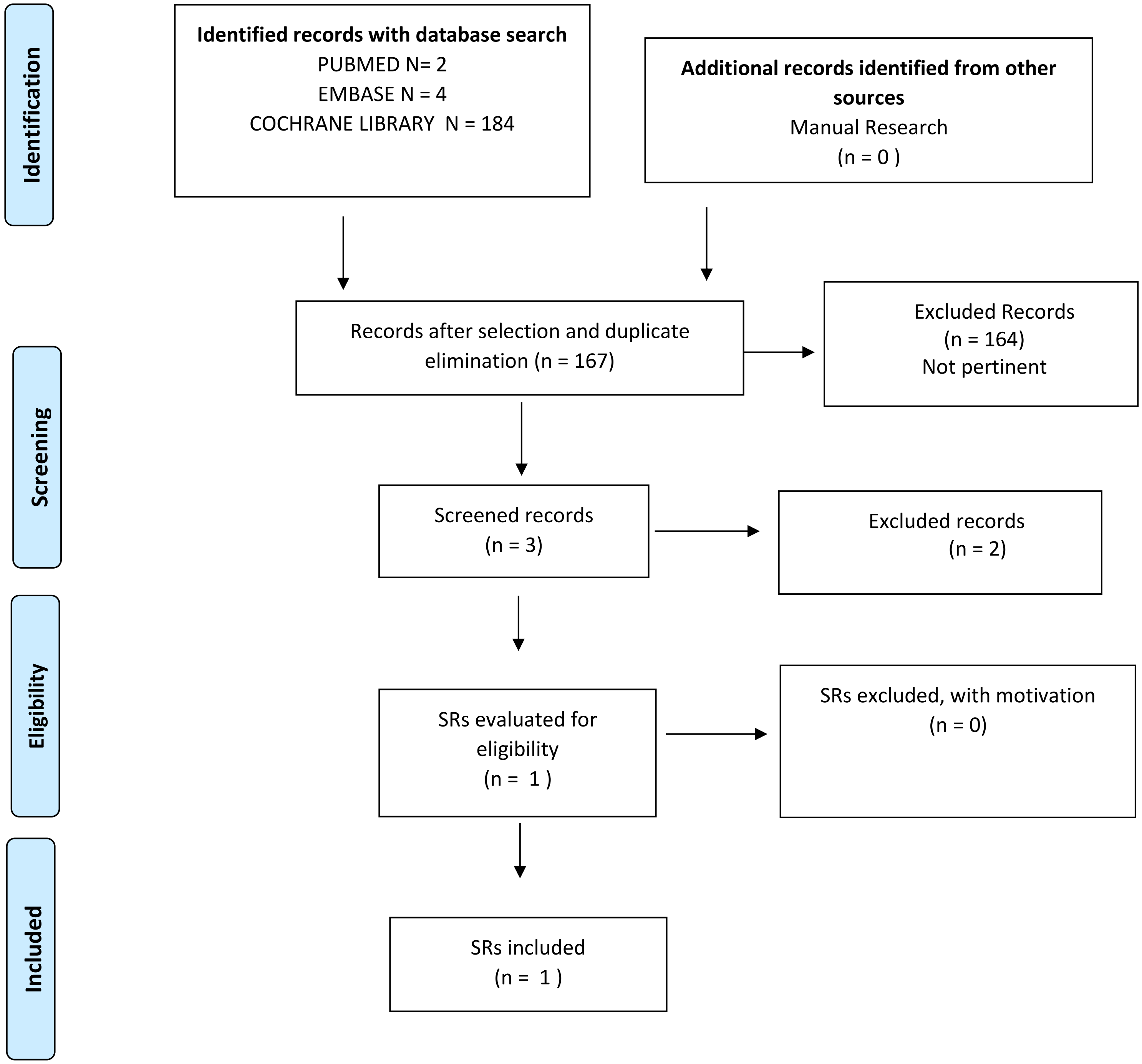

Figure a4.6. Studies search flow diagram.

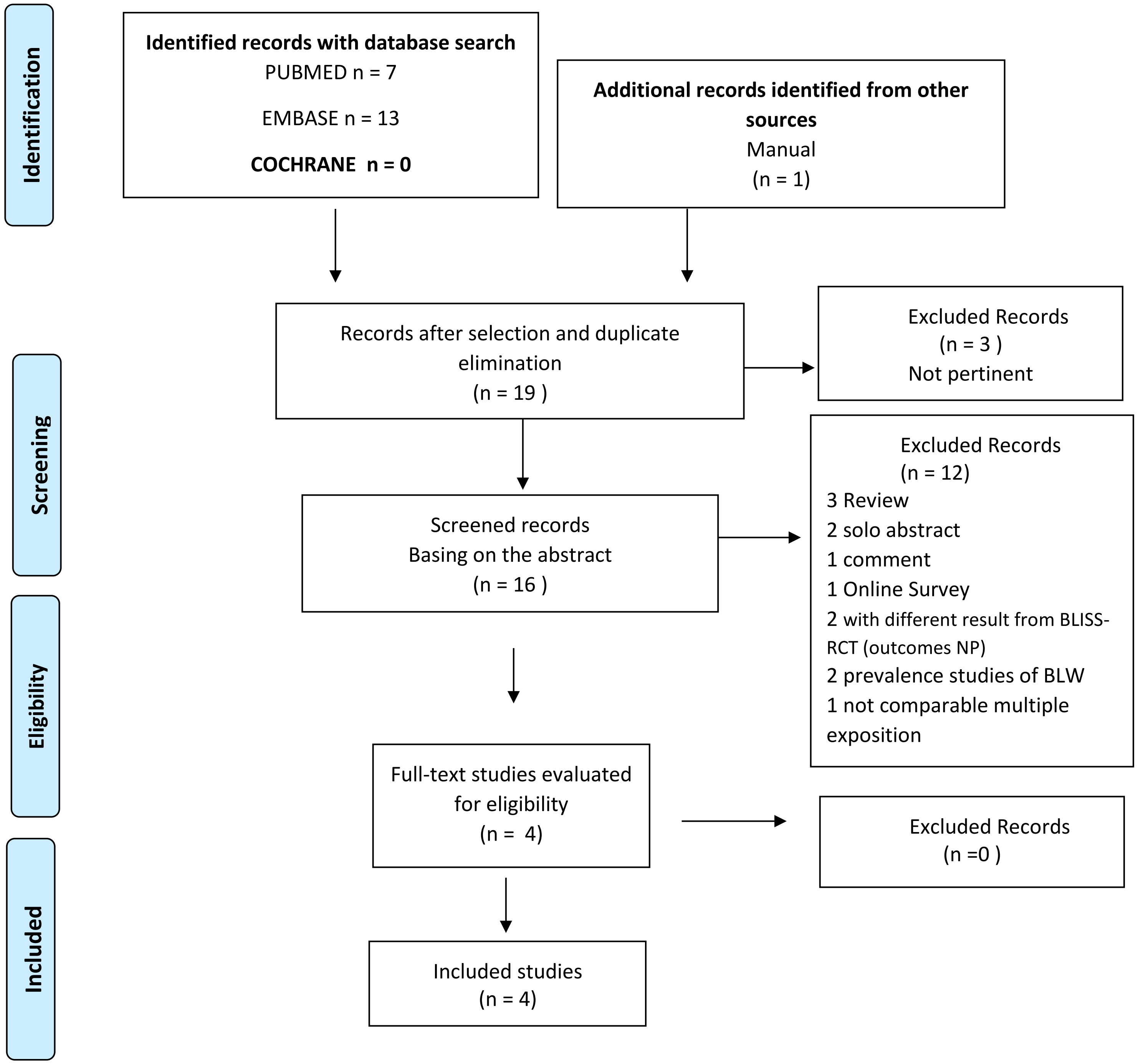

4a. METHODOLOGICAL EVALUATION OF THE EVIDENCES

Table a4a.1. Appraisal of the Systematic Review

AMSTAR 2	D'Auria et a. 2018 [1]
**1. Did the research questions and inclusion criteria for the review include the components of PICO? (Yes/No)**	Yes
**2. Did the report of the review contain an explicit statement that the review methods were established before the conduct of the review and did the report justify any significant deviations from the protocol? (Yes/Partial Yes/No)**	Partial yes
**3. Did the review authors explain their selection of the study designs for inclusion in the review? (Yes/No)**	Yes
**4. Did the review authors use a comprehensive literature search strategy? (Yes/Partial Yes/No)**	Partial yes
**5. Did the review authors perform study selection in duplicate? (Yes/No)**	Yes
**6. Did the review authors perform data extraction in duplicate?(Yes/No)**	Yes
**7. Did the review authors provide a list of excluded studies and justify the exclusions?<br>(Yes/Partial Yes/No)**	Yes
**8. Did the review authors describe the included studies in adequate detail?<br>(Yes/Partial Yes/No)**	Yes
**9. Did the review authors use a satisfactory technique for assessing the risk of bias (RoB) in individual studies that were included in the review?<br>(Yes/Partial Yes/No/Includes only NRSI-RCT)**	YES<br>YES
**10. Did the review authors report on the sources of funding for the studies included in the review?(Yes/No)**	No
**11. If meta-analysis was performed did the review authors use appropriate methods for statistical combination of results? (Yes / No / No meta-analysis conducted)**	No meta-analysis
**12. If meta-analysis was performed, did the review authors assess the potential impact of RoB in individual studies on the results of the meta-analysis or other evidence synthesis? (Yes / No / No meta-analysis conducted)**	No meta-analysis
**13. Did the review authors account for RoB in individual studies when interpreting/ discussing the results of the review? (Yes/No)**	Yes
**14. Did the review authors provide a satisfactory explanation for, and discussion of, any heterogeneity observed in the results of the review? (Yes/No)**	Yes

**15. If they performed quantitative synthesis did the review authors carry out an adequate investigation of publication bias (small study bias) and discuss its likely impact on the results of the review? (Yes / No / No meta-analysis conducted)**	No meta-analysis
**16. Did the review authors report any potential sources of conflict of interest, including any funding they received for conducting the review? (Yes/No)**	Yes
**OVERALL EVALUATION**	**MODERATE QUALITY**

Table a4a.2. SRs excluded with motivation.

ESCLUDED	Reason for exclusion
Harrison et al. 2017 [2]	Outcomes not pertinent
Arikpo et al. 2018 [3]	Exposition not pertinent
Gomez et al. 2020 [4]	Narrative review

Table a4a.3. Appraisal of the Studies

	Newcastle Quality Assessment Scale CROSS-SECTIONAL STUDIES							
	**Selection**				**Comparability**	**Outcome**		
**Study**	**Representativeness of the sample**	**Sample size**	**Non-Response rate**	**Ascertainment of exposure (max 2)**	**Comparability between groups, confounders are controlled (max2)**	**Outcome evaluation (max 2)**	**Statistical test**	**Total**
Townsend et al. 2012 [5]	c	b	1a	c	None	c	1a	**3 Low**
Brown et al. 2015 [6]	1b	b	b	1a	1a, 1b	c	1a	**5 Moderate**

RCTs

Figure a4a.7. Risk of bias summary: review authors' judgements about each risk of bias item for each included study.

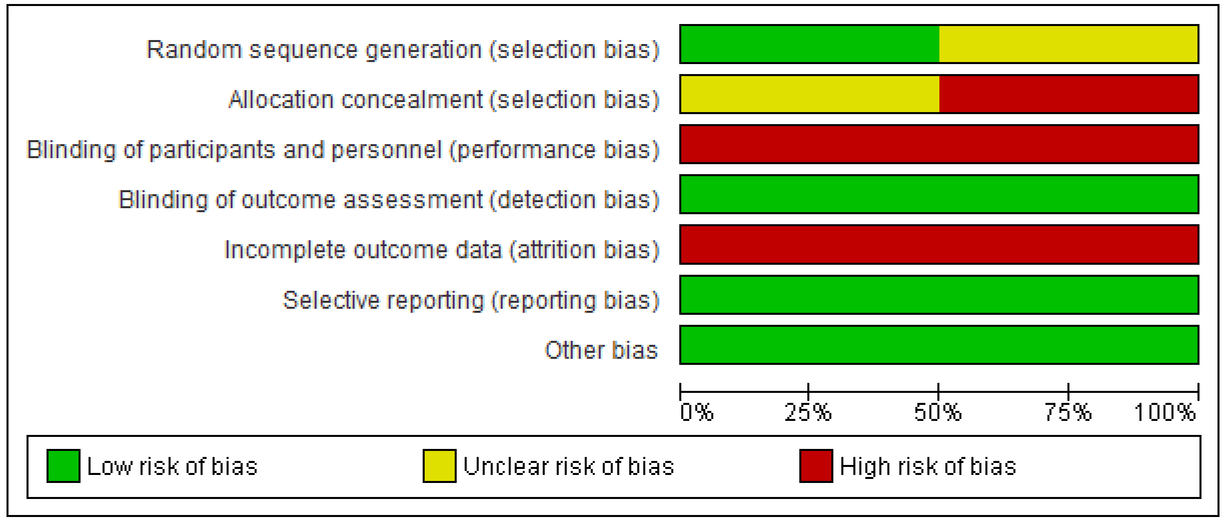

Figure a4a.8. Risk of bias graph: review authors' judgements about each risk of bias item presented as percentages across all included studies [7, 8]

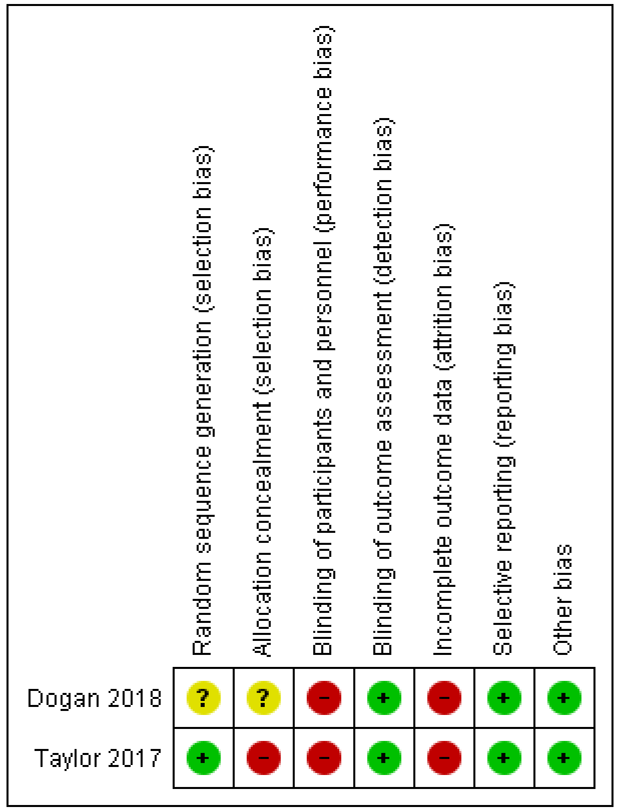

4a. RECOMMENDATIONS OF GLs, RESULTS IN SRs AND STUDIES

A4. BLW/BLISS. Growth and development of overweight/obesity

– ***Can using the Baby-Led Weaning (BLW; Self-weaning) method during the Complementary Feeding (CF) period influence, in a positive or negative way, the physical growth process in later ages?***	**P.** In a healthy child aged 6-24 months<br>**I.** the Baby-Led Weaning (or the BLISS method)<br>**C**. compared to other power models<br>**O**. does it involve a different physical growth?
– ***Can the BLW / BLISS method during CF influence, in a positive or negative way, the development of overweight / obesity in later ages?***	**P.** In a healthy child aged 6-24 months<br>**I.** the Baby-Led Weaning (or the BLISS method)<br>**C**. compared to other power models<br>**O**. does it involve a different risk of overweight/obesity in later age?

Table a4.4. Included SRs: Characteristics, Results, and Conclusions

**Systematic Review**	**Population and purpose of the SR**	**Results**	**Conclusions**
D'Auria et al. 2018 (SR of RCTs and observational studies) [1]	Children in the age of CF fed according to the BLW method, compared with children fed with traditional methods<br><u>Long term health outcomes: auxological parameters</u><br>(risk of suffocation, metabolic parameters, relational indicators)	Apparently in favor of BLW in the 2 observational studies: fewer overweight subjects and higher number of underweight subjects in the medium term.<br>No significant results in the randomized study that used the BLISS method	No valid conclusions possible regarding the influence of BLW on auxological parameters

Table a4.5. Included studies: Characteristics and Results

**Study**	**Study design**	**Population**	**Test**	**Primary Outcome**	**Secondary Outcomes**	**Follow-up**	**Results**
Townsend et al. 2012 [5]	Cross-sectional Data from self-completed questionnaire	N = 155<br>Age 20–78 mo	BLW vs traditional CF	BMIz sore at 20-78 month	/	/	BLW associated with lower weight and less likely t[illegible] be overweight or obese.(p=0.02)

Brown et al. 2015 [6]	Cross-sectional Data from self-completed questionnaire	N = 298<br>age 18–24 mo	BLW vs traditional CF	Weigth at 18-24 month	/	/	BLW associated with lower weight and less likely to be overweight or obese.(p =0.005)
Taylor et al. 2017 (BLISS) [8]	RCT in open	N = 206 healthy women (105<br>BLISS, 101 traditional CF)<br>Outcomes measured until 24 mo	BLISS vs traditional CF	BMI z-score at 12 and 24 mo	Caloric self-regulation<br><br>Caloric intake	24 mo (N = 166)	Mean BMI z-score not significantly different in the groups, at 12 mo and at 24 mo
Dogan et al. 2018 (BLISS) [7]	RCT in open	302 children aged 5-6 mo (156 BLISS, 146 traditional CF)	BLISS vs traditional CF	Weight, length, and CC	Choking, haematological parameters and eating behaviors, at 12 mo	12 mo (N = 280)	Faster weight gain from 6 to 12 mo (p = 0.001) in traditionally fed infants

A4. EVIDENCE PROFILE GRADE

A4. BLW/BLISS. Growth and risk of overweight/obesity

Table a4.6. Growth.

[BLW-BLISS] compared to [other models of CF] in [healthy child, can influence, in positive or negative way, the process of staturo-weight growth in later age]

Patient or population: [healthy child, can influence, in positive or negative way, the process of staturo-weight growth in later age]

Setting: Outpatient

Intervention: [BLW-BLISS]

Comparator: [other models of CF]

Certainty assessment							N of patient		Outcomes		Certain	Importance
N of studies	Study design	Risk of distortion	Lack of reproducibility of results	Lack of generalizability	Imprecision	Further considerations	[BLW-BLISS]	[other models di CF]	Relative (95% CI)	Absolute (95% CI)		
**Growth (BLW – observational studies) (follow up: interval 18 month at 78 month; evaluated with: BMI-BMI z score (% of underweight))**												
2 [1,2]	Observational studies	Serious [a]	Not important	Not important	Serious [a]	all plausible residual confounders would suggest a spurious effect, while no effect was observed	15/255 (5.9%)	4/198 (2.0%)	OR 3.46 (1.14 at 10.56)	**+46 per 1.000** (from +3 to +159)	⨁◯◯◯ VERY LOW	CRITICAL
**Growth (BLISS-RCT) (follow up: medium 24 months; evaluated with: (WHO P/L z-score = -2 SD) % underweight)**												
1 [3]	Randomized studies	serious [b]	Not important	Not important	Not important	High suspect of publication bias [c]	3/142 (2.1%)	0/138 (0.0%)	RR 6.80 (0.35 at 130.52)	**- 0 per 1.000** (from - 0 to -0)	⨁⨁◯◯ LOW	CRITICAL

Table a4.7. Overweight/obesity risk

Overweight/obesity risk (BLW-observational studies) (follow up: interval 18 month to 78 month; evaluated with: BMI-BMI z-score (% obesity overweight))

Certainty assessment							N of patient		Outcomes		Certain	Importance
N of studies	Study design	Risk of distortion	Lack of reproducibility of results	Lack of generalizability	Imprecision	Further considerations	[BLW-BLISS]	[other models di CF]	Relative (95% CI)	Absolute (95% CI)		
2 [1,2]	Observational studies	Serious [a]	Not important	Not important	Serious [a]	High suspect of publication bias all plausible residual confounders could reduce the demonstrated effect [a]	21/226 (9.3%)	41/298 (13.8%)	OR 0.40 (0.23 at 0.70)	**-78 per 1.000** (from -102 to -37)	⊕◯◯◯ VERY LOW	IMPORTANT
**Overweight/obesity risk (BLISS-RCT) (follow up: medium 24 months; evaluated with: WHO P/L z score/BMI z-score (% obesity overweight))**												
2 [3,4]	Randomized studies	Serious [d]	Not important	Not important	Very serious [e]	All plausible residual confounders could reduce the demonstrated effect	5/243 (2.1%)	33/233 (14.2%)	RR 0.12 (0.00 at 7.91)	**-125 per 1.000** (from -- to +979)	⊕⊕◯◯ LOW	IMPORTANT

CI: Confidence interval; **OR:** Odds ratio; **RR:** Risk ratio

Explanations

a. Voluntary recruitment of mothers intending to use the BLW, uncertainty in weight measurement that was entrusted to parents with unspecified frequency, and significant loss of data during the observation period

b. Loss at follow-up at 24 months = 21.4%, lack of blindness in patients and no ITT analysis

c. Single RCT, possible beta error

d. Low methodological quality for% loss at follow-up, lack of blindness, and no ITT analysis

e. Discordant results, high heterogeneity

References

1. Townsend E, et al.. Baby knows best? The impact of weaning style on food preferences and body mass index in early childhood in a case-controlled sample.. BMJ OPEN 2012;2:e000298.; 2012.

2. Brown A, Lee DW,. Early influences on child satiety-responsiveness: the role of weaning style. . Pediatr Obes. 2015 Feb;10(1):57-66; 2015.

3. Dogan E, Yilmaz G,Caylan N,et al.. Baby-led complementary feeding: randomized controlled study. . Pediatr Int. 2018;60(12):1073-1080; 2018.

4. Taylor RW, Williams SM,Fangupo LJ,et al.. Effect of a baby-led approach to complementary feeding on infant growth and overweight: a randomized clinical trial. . JAMA Pediatr. 2017;171:838-46; 2017.

A4. ReCF / nReCF and growth

- ***Can ReCF during CF period influence, positively or negatively, the physical growth process?***

- ***Can nReCF during CF period influence, positively or negatively, the physical growth process?***

PICOs

A.

P. Healthy child aged 6-24 months
I. Responsive Complementary Feeding
C. Compared to others feeding models
O. Does it involve a different physical growth in later ages?

B .

P. Healthy child aged 6-24 months
I. Non-responsive Complementary Feeding
C. Compared to others feeding models
O. Does it involve a different physical growth in later ages?

KEY WORDS

Population

I. No age limit
J. ([infant]/lim OR [child]/lim OR [preschool]/lim

Exposure Factors / Comparison

A. Infant Nutritional Physiological Phenomena [MeSH]
B. Weaning"[MeSH])
C. "Feeding Behavior"[MeSH]
D. "Feeding Methods"[MeSH]
E. "feeding practice"[All Fields]
F. “parenting style”
G. “feeding style” [All Fields]
H. “feeding patterns” [All Fields]
I. “responsive feeding” [All Fields]
J. “non responsive feeding” ”[All Fields]
K. “responsiveness”[All Fields]
L. “complementary feeding”[All Fields]

Outcomes

"Growth and Development"[Mesh]

"Growth"[Mesh])
"Growth Charts"[Mesh])
"Body Height"[Mesh])
"Body Weight"[Mesh])
"Body-Weight Trajectory"[Mesh])
"Body Weight Changes"[Mesh]

Guidelines search

PubMed

#1

("Growth and Development"[Mesh] OR "Growth"[Mesh]) OR "Growth Charts"[Mesh] AND ((Practice Guideline[ptyp] OR Guideline[ptyp]) AND "2014/08/11"[PDat] : "2021/03/15"[PDat] AND "humans"[MeSH Terms])

EMBASE

#1

('growth'/exp OR 'growth, development and aging'/exp) AND ('complementary feeding'/exp OR 'responsiveness' OR 'responsive feeding' OR 'non responsive feeding') AND ([adolescent]/lim OR [child]/lim OR [infant]/lim OR [preschool]/lim OR [school]/lim OR [young adult]/lim) AND [2014-2019]/py AND 'practice guideline'/de

UPTODATE https://www.uptodate.com/home

Society Guideline Links: *Breastfeeding and infant nutrition*

SOCIETY GUIDELINE LINKS:

National Guideline Clearinghouse (NGC) https://www.ahrq.gov/gam/index.html

Canadians Medical Association (CMA) https://www.cma.ca/clinicalresources/practiceguidelines

National Guideline Centre (NGC) - National Institute of Health and Care Excellence (NICE) https://www.rcplondon.ac.uk/about-us/what-we-do/national-guideline-centre-ngc

Scottish Intercollegiate Guidelines Network (SIGN) https://www.sign.ac.uk/our-guidelines.html

Australian Clinical Practice Guidelines (ACPG) https://www.clinicalguidelines.gov.au/

New Zealand Guidelines Group (NZGG) https://www.health.govt.nz/about-ministry/ministry-health-websites/new-zealand-guidelines-group

American Academy of Pediatrics (AAP) https://www.aap.org/en-us/Pages/Default.aspx

EPA/UNEPSA http://www.epa-unepsa.org/

Guidelines International Network https://g-i-n.net/

Società Italiana di Pediatria (SIP) http://www-sip.it/

Società Italiana di Pediatria Preventiva e Sociale (SIPPS) https://www.sipps.it/

Società Italiana di Endocrinologia e Diabetologia Pediatrica (SIEDP)
http://www.siedp.it/pagina/84/linee+guida%2C+raccomandazioni+e+consensus

Systematic Reviews search

COCHRANE LIBRARY

A. "Child Health/Develop-psych-learn problems" in Title Abstract Keyword
B. "Endocrine & Metabolic" in Title Abstract Keyword
K. "Weaning"

Custom date range Topics: 01.01.2014 - 15.03.2021

PubMed

#1

("responsive feeding"[All Fields] OR "non-responsive feeding"[All Fields] OR "responsiveness"[All Fields] OR "Weaning"[All Fields] OR "Infant Nutritional Physiological Phenomena"[MeSH] OR "complementary feeding"[All Fields] OR "Feeding Behavior"[All Fields]) AND ((((("Growth and Development"[Mesh]) OR "Growth"[Mesh]) OR "Growth Charts"[Mesh]) OR "Body Height"[Mesh]) OR "Body Weight"[Mesh]) OR "Body-Weight Trajectory"[Mesh]) OR "Body Weight Changes"[Mesh]) AND ((Meta-Analysis[ptyp] OR systematic[sb]) AND ("2014/08/13"[PDat] : "2021/03/15"[PDat])

EMBASE

#1

('complementary feeding'/exp OR 'weaning'/exp OR 'responsiveness' OR 'responsive feeding' OR 'non responsive feeding') AND ('growth'/exp OR 'growth, development and aging'/exp OR 'body weight'/exp OR 'body mass'/exp) AND [2014-2021]/py AND ([cochrane review]/lim OR [systematic review]/lim OR [meta analysis]/lim)

Primary Studies search (post- Spill SRs 2019 AJCN)

Cochrane Trials ("2017/01/01"[PDat] : "2021/03/15"[PDat])

A. "Child Health/Develop-psych-learn problems" in Title Abstract Keyword
B. "Endocrine & Metabolic" in Title Abstract Keyword

L. “Weaning”

PubMed

#1

(incentiv* OR indulgen*[tiab] OR authorita*[tiab] OR reward* OR control* OR pressur* OR restrict* OR monitor* OR respons* OR sooth*[tiab] OR encourag* OR discourage* OR uninvolv* OR disengage* OR parenting style* OR laissez-faire OR laissez faire* OR non-respons* OR nonrespons* OR force*) AND (feeding* OR fed[tiab] OR eat[tiab] OR eating OR "Feeding Methods"[Mesh:noexp] OR "Feeding Behavior"[Mesh:NoExp] OR satiety OR hunger OR hungry OR satiat*) AND (cue OR cues) OR feeding method* OR feeding practice* OR feeding pattern* OR feeding frequenc* OR infant feed* OR feeding behavior*[tiab] OR feeding style* OR feeding strategy*)AND (((("Growth and Development"[Mesh]) OR "Growth"[Mesh]) OR "Growth Charts"[Mesh]) AND (Clinical Trial[ptyp] OR Comparative Study[ptyp] OR Controlled Clinical Trial[ptyp] OR Observational Study[ptyp] OR Pragmatic Clinical Trial[ptyp] OR Randomized Controlled Trial[ptyp] OR Multicenter Study[ptyp]) AND ("2017/01/01"[PDat] : "2021/03/15"[PDat])

#2

("responsive feeding"[All Fields] OR "non-responsive feeding"[All Fields] OR “responsiveness”[All Fields] OR "Weaning"[All Fields] OR "Infant Nutritional Physiological Phenomena"[MeSH] OR “complementary feeding”[All Fields] OR "Feeding Behavior"[All Fields]) **AND** (((((("Growth and Development"[Mesh]) OR "Growth"[Mesh]) OR "Growth Charts"[Mesh]) OR "Body Height"[Mesh]) OR "Body-Weight Trajectory"[Mesh]) OR "Body Weight Changes"[Mesh]) **AND** (Clinical Trial[ptyp] OR Comparative Study[ptyp] OR Controlled Clinical Trial[ptyp] OR Observational Study[ptyp] OR Pragmatic Clinical Trial[ptyp] OR Randomized Controlled Trial[ptyp] OR Multicenter Study[ptyp]) AND ("2017/01/01"[PDat] : "2021/03/15"[PDat])

EMBASE

#1

('complementary feeding'/exp OR 'responsiveness' OR 'responsive feeding' OR 'non responsive feeding') AND ('growth'/exp OR 'growth, development and aging'/exp) AND [2017-2021]/py AND ('case control study'/de OR 'clinical trial'/de OR 'cohort analysis'/de OR 'comparative effectiveness'/de OR 'comparative study'/de OR 'controlled clinical trial'/de OR 'controlled study'/de OR 'cross-sectional study'/de OR 'double blind procedure'/de OR 'human'/de OR 'longitudinal study'/de OR 'multicenter study'/de OR 'observational study'/de OR 'prospective study'/de OR 'randomized controlled trial'/de OR 'retrospective study'/de) AND ([child]/lim OR [infant]/lim OR [preschool]/lim)

Figure a4.9. Guideline search flow diagram

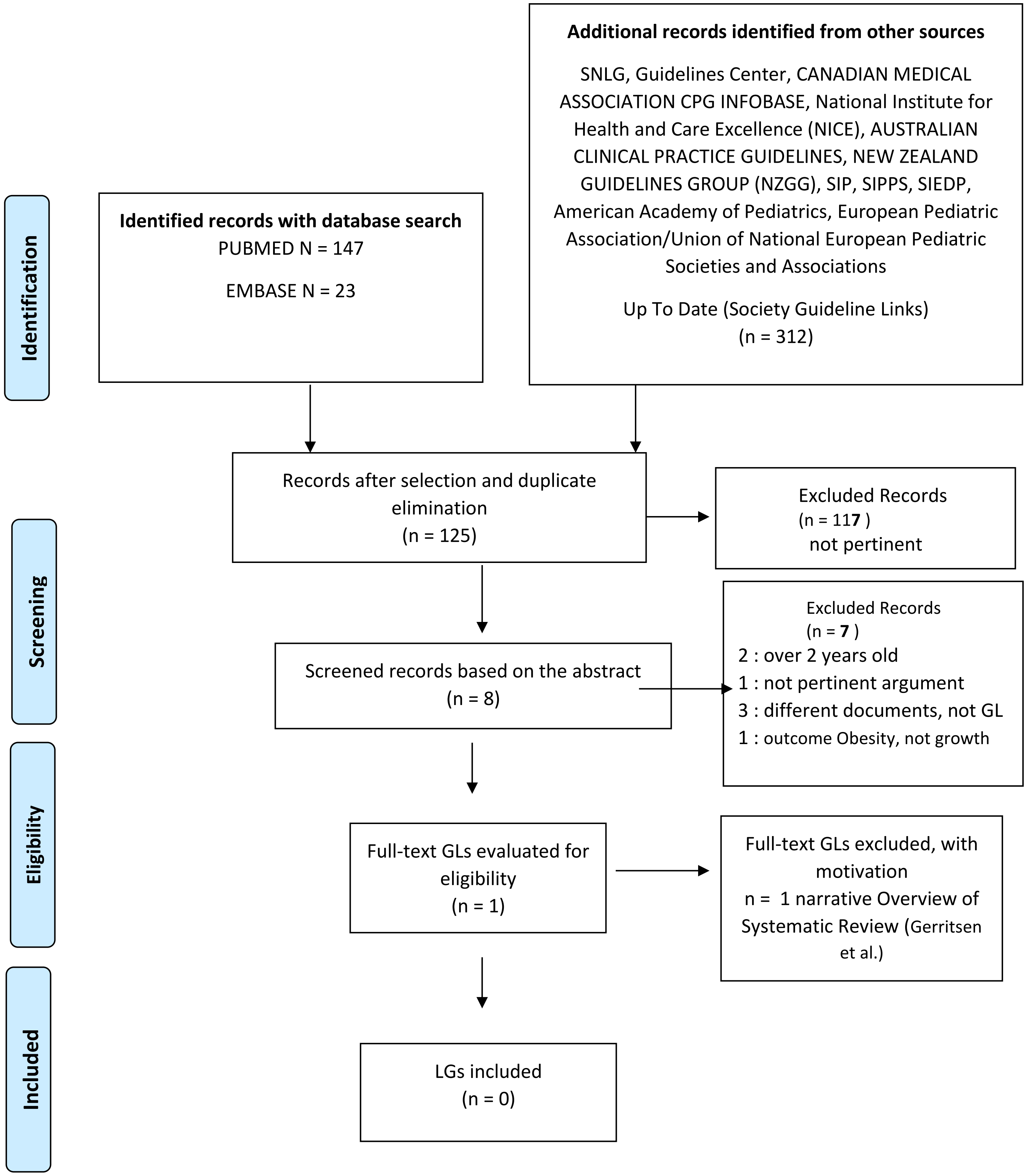

Figure a4.10. SRs search flow diagram

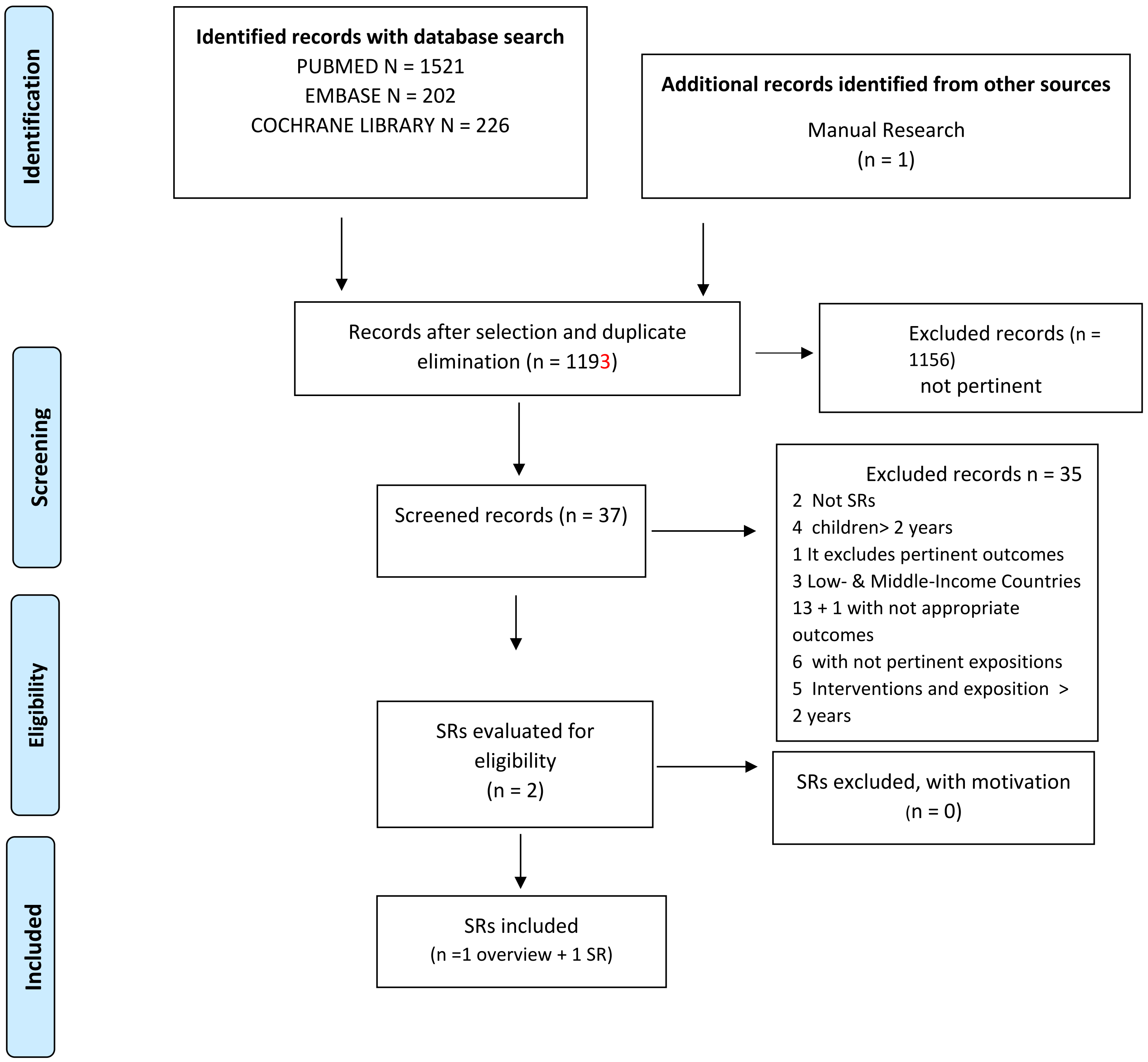

Figure a4.11. Studies search flow diagram. (**post-bibliographic search in the SR of Spill 2019 - January 2017)**

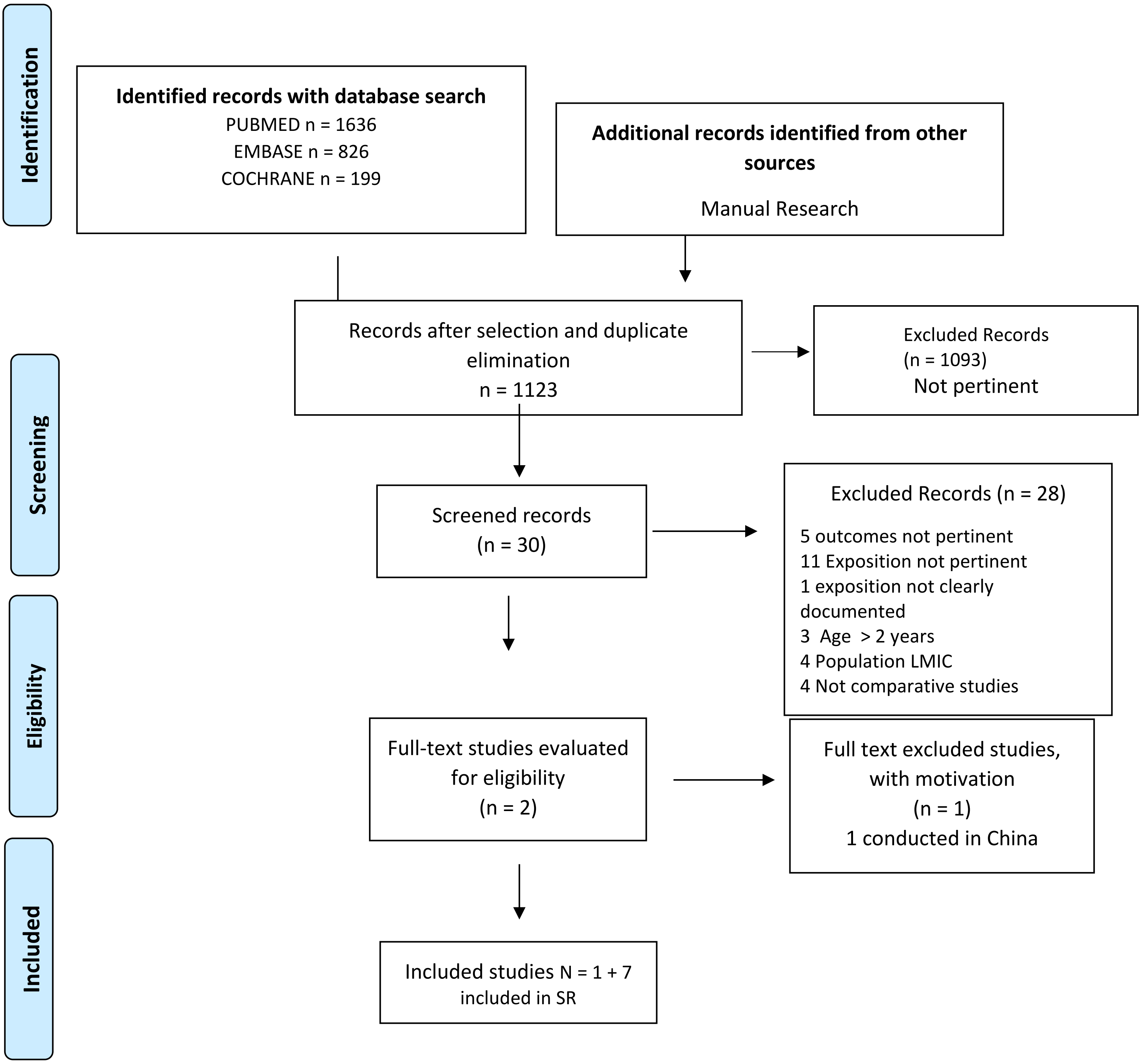

A4. METHODOLOGICAL ASSESSMENT

a4. ReCF/nReCF and Growth

Table a4.8. Evaluation of Systematic Review Overviews

**COCHRANE TOOL FOR OVERVIEWS OF REVIEWS**		Gerritsen et al. 2017 [9]
Objective	To summarize evidence from *systematic reviews* examining effects of interventions	Appropriate
Selection criteria	Describe inclusion and exclusion criteria for review	Appropriate
Search	Typically, look only for relevant Cochrane intervention reviews.	Appropriate (RCT and Cohort Studies if recent and important)
Data collection	From the included systematic review	Appropriate
Assessment of limitation	For included sistematic review	Appropriate
Quality of the evidences	As far as possible it should be based on evaluation reported in the included systematic review	Appropriate
Analysis	Summary of the results of the reviews; further analysis can be undertaken for comparisons between reviews, typically indirect comparisons of multiple interventions.	Appropriate (Summaries of results almost always only narrative)

Table a4.9. Appraisal of the Systematic Review

AMSTAR 2	Spill et al. 2019 [10]
**1. Did the research questions and inclusion criteria for the review include the components of PICO?**<br><br>**(Yes/No)**	Yes
**2. Did the report of the review contain an explicit statement that the review methods were established before the conduct of the review and did the report justify any significant deviations from the protocol?**<br><br>**(Yes/Partial Yes/No)**	Yes
**3. Did the review authors explain their selection of the study designs for inclusion in the review?**<br><br>**(Yes/No)**	Yes
**4. Did the review authors use a comprehensive literature search strategy?**<br><br>**(Yes/Partial Yes/No)**	Yes
**5. Did the review authors perform study selection in duplicate?**<br><br>**(Yes/No)**	Yes
**6. Did the review authors perform data extraction in duplicate?**<br><br>**(Yes/No)**	Yes
**7. Did the review authors provide a list of excluded studies and justify the exclusions?**<br><br>**(Yes/Partial Yes/No)**	Yes
**8. Did the review authors describe the included studies in adequate detail?**<br><br>**(Yes/Partial Yes/No)**	Yes
**9. Did the review authors use a satisfactory technique for assessing the risk of bias (RoB) in individual studies that were included in the review?**<br><br>**(Yes/Partial Yes/No/Includes only NRSI-RCT)**	Yes<br><br>Yes
**10. Did the review authors report on the sources of funding for the studies included in the review?**<br><br>**(Yes/No)**	No
**11. If meta-analysis was performed did the review authors use appropriate methods for statistical combination of results?**	///

**(Yes / No / No meta-analysis conducted)**	
**12. If meta-analysis was performed, did the review authors assess the potential impact of RoB in individual studies on the results of the meta-analysis or other evidence synthesis?**<br><br>**(Yes / No / No meta-analysis conducted)**	///
**13. Did the review authors account for RoB in individual studies when interpreting/ discussing the results of the review?**<br><br>**(Yes/No)**	Yes
**14. Did the review authors provide a satisfactory explanation for, and discussion of, any heterogeneity observed in the results of the review?**<br><br>**(Yes/No)**	Yes
**15. If they performed quantitative synthesis did the review authors carry out an adequate investigation of publication bias (small study bias) and discuss its likely impact on the results of the review?**<br><br>**(Yes / No / No meta-analysis conducted)**	///
**16. Did the review authors report any potential sources of conflict of interest, including any funding they received for conducting the review?**<br><br>**(Yes/No)**	Yes
**OVERALL EVALUATION**	**MODERATE QUALITY**

Figure a4.12. Risk of bias summary: review authors' judgements about each risk of bias item for each included study. [11,12,13]

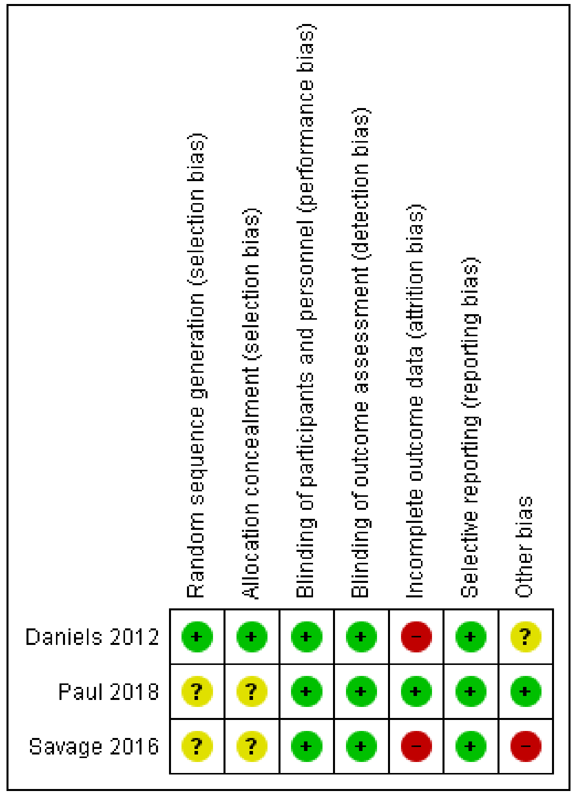

Figura a4.13. Risk of bias graph: review authors' judgements about each risk of bias item presented as percentages across all included studies

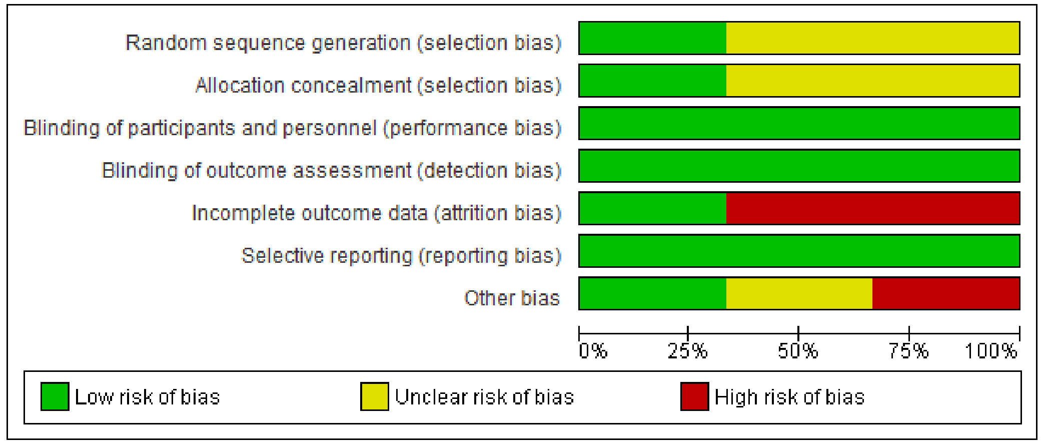

Table a4.10. Appraisal of the Studies

	Newcastle Quality Assessment Scale COHOTS STUDIES								
	**Selection**				**Comparability**	**Outcome**			
**Study**	**Representativeness of the exposed cohort**	**Selection of non-exposed cohort**	**Ascertainment of the exposure**	**Demonstration that the outcome of interest is not present at the start of the study**	**Comparability of cohorts based on design or analysis**	**Outcome evaluation**	**Was the follow-up long enough for the outcome to occur?**	**Adequacy of cohort follow-up**	**Total**
Wright et al. 2006 [14]	1a	1a	c	1a	1a<br>1b	1b	b	>40% (not described)	**7**
Chaidez et al. 2015 [15]	c	1a	1b	Presence of overweight in some	1a<br>1b	1b	1a	36% (description of the characteristics of the lost)	**6**
Dinkevich et al. 2015 [16]	1b	1a	1b<br>c	Presence of overweight in some		1b	1a	27% (description of the characteristics of the lost)	**7**
Hittner et al. 2016 [17]									
Stifter et al. 2015 [18]	c	1a	d	NO	1a<br>1b	1b	1a	16% (description of the characteristics of the lost)*	**6**

Table a4.11. Excluded studies with motivation.

**Excluded studies**	**Reason for exclusion**
Paul et al. 2011 [19]	Low methodological quality. Loss to follow-up > 20%

Daniels et al. 2013 [20] (follow-up di Daniels 2012)	Low methodological quality. Loss to follow-up > 20%
Daniels et al. 2015 [21] (follow-up di Daniels 2012)	Low methodological quality. Loss to follow-up > 20%
Worobey et al. 2009 [22]	Not pertinent, conducted on Hispanic and Black american children of low socioeconomic status
Ma et al. 2015 [23]	Not pertinent, conducted on Asiatic children
Shi et al. 2017 [24]	Not pertinent, conducted on Asiatic children

A4. RECOMMENDATIONS OF GLs, RESULTS IN SRs AND STUDIES

a4. ReCF / nReCF and growth

A. Can ReCF during CF period influence, positively or negatively, the physical growth process?<br><br>***B. Can nReCF during CF period influence, positively or negatively, the physical growth process?***	**a.**<br>**P.** Healthy child aged 6-24 months<br>**I.** Responsive Complementary Feeding<br>**C.** Compared to others feeding models<br>**O.** Does it involve a different physical growth in later ages?<br><br>**b.**<br>**P.** Healthy child aged 6-24 months<br>**I.** Non-responsive Complementary Feeding<br>**C.** Compared to others feeding models<br>**O.** Does it involve a different physical growth in later ages?

Table a4.12. Included SRs: Characteristics, Results, and Conclusions

Systematic Review	Population and purpose of the SR	Results	Conclusions
Gerritsen et al. 2017 [9]	Present the best evidence currently available on the effect of different eating behaviours ("how" we eat) on the diet itself and on body size.<br>Themes: breastfeeding, parental feeding practices and parenting styles, role of adult role models, responsive feeding, meal times and food culture.<br>The analyses, based on Systematic Reviews and primary studies, cover all periods of life, from pregnancy to adulthood.	**1. Responsive nutrition; based on RS:**<br>The most frequent finding (16/31) across the three age groups was an association with parental feeding control and weight gain / child status. Restriction of food intake was related to a higher BMI and / or overweight and pressure during feeding was related to a lower BMI / weight gain. There was a positive relationship between indulgent eating behaviour and BMI and / or overweight and a negative association between indulgent eating and fruit and vegetable intake by children (Hurley et al 2011).<br>Of the nine studies reviewed, three revealed associations with the size of feeding response as defined by the proposed model (Figure 8), but only one of these studies evaluated feeding interactions longitudinally, which DiSantis et al. found it necessary to truly assess the dynamic nature of feeding interactions between caregiver and infant and their impact on obesity outcomes. (DiSantis 2011).<br>Excluding, in the present review, the 3 papers included by Gerritsen et al. on the Baby-Led Weaning<br><br>**2.Parenting Practices of Nutrition and Parenting Style:**<br>Six systematic reviews since 2007 have reported that restrictive feeding and parental control practices (i.e., denying intake, either of overall energy or of particular foods and beverages) are associated with an increase in mass index body (BMI) in childhood. [**Shloim et al 2015; Hurley et al 2011; Ventura and Birch 2008; Clark et al 2007; Russell et al 2016; Fraser et al 2011**]	1. Parental awareness and recognition of hunger and satiety cues can lead to small improvements in babies' and children's diets, food preferences and eating behaviours, and can be protective against excessive weight gain. Responsive feeding. Grade B.<br><br>2. Parental restriction of the child's food intake (when he seems to eat too much) or pressure from the child to eat (when he seems to eat too little) are counterproductive, as these coercive practices can lead to behaviour unhealthy eating and weight gain. Parent feeding practices and parenting style. Grade A.

Table a4.13. Included SRs: Characteristics, Results, and Conclusions

Systematic Review	Population and purpose of the SR	Results	Conclusions
Spill et al. 2019 [10]	<u>Population</u> consisting of dyads *Parent-caregiver* and *infant-toddler*.<br><br><u>Purpose</u>: know the relationship between the different nutrition practices (of control, constriction, restriction, monitoring, responsiveness and non-responsiveness to the manifestations of hunger and satiety of the child) and the growth, size and body composition of children.<br><br><u>Studies</u>: controlled, randomized and non-randomized, prospective and retrospective observational studies, pre-post controlled studies, nested-case-case-control studies.	**<u>Responsive feeding</u>**<br><br>In the RCT by Daniels et al. there was a difference in weight results at 13.5 mo; however, there were no significant differences in weight gain indicators at 20 mo or 4.5 years of follow-up between the intervention and control groups.<br>The remaining 2 controlled studies, 1 RCT (18) and 1 non-randomized controlled study (21), had limitations that made them less informative to answer the question of the systematic review.<br><br>Pressure to finish food at 3 mo of age was associated with lower WAZ and lower odds of WAZ> 90th percentile at 3, 6, 9, 12, and 18 mo of age (7). Pressure to eat was associated with a lower WLZ in children aged 6 to 30 mo (25) and pressure to eat at 1 year of age predicted a weight of less than 2 years (9).<br><br>Another study measured the "maternal response to food refusal," which refers to a mother responding to a baby refusing food by encouraging the baby to eat or by offering additional food (35). In this study, the "maternal response to food refusal" at 8 mo of age was significantly associated with less weight gain from birth to 12 mo of age (35). Three other studies, however, found no significant association between pressure to eat and the baby's weight after adjusting for initial weight.<br><br>Thompson et al. (7) found an association between the use of restriction at 3 mo and WLZ at 3, 6, 9, 12, and 18 mo of age. Dinkevich et al. (25) reported an association between restrictive feeding practices and WLZ in infants aged 6 to 30 mo. Using data from a cohort of participants, 2 studies evaluated maternal restriction at 1 year of age and weight outcomes at 3 years of age (10, 11). After adjusting for the initial weight, Taveras et al. (11) found an association between maternal restriction and an increased likelihood of having a BMIZ between the 85th and 95th percentiles, but Rifas-Shiman et al. (10) found no association between maternal restriction and an increased likelihood of having a BMIZ> 95th percentile. On the other hand, Farrow et al. (9) found that greater restriction was associated with lower standardized weight scores.<br>Hittner et al. (28) found a significant interaction between maternal restriction and baby sex as a predictor of BMI change over time. For boys, greater restriction was associated with lower BMI, but for girls, greater restriction was associated with higher BMI (28). Gregory et al. (26) found no association between food restriction and the child's weight.<br><br>Worobey et al. (34) found that reactive feeding practices (being more sensitive to the baby's cues) were associated with less weight gain in the baby from 6 to 12 mo of age, but there was no association with weight gain. from 3 to 6 mo of age. The other 5 studies found no association between reactive feeding practices and the infant's weight, height, and / or head circumference (7, 27, 31-33).<br><br>Of the studies that found no association, 2 looked at the difference between feeding on the infant's demand versus feeding on a fixed schedule (27, 33). Morris et al. (32) found no association between on-demand feeding practices at 3 mo of age and the thickness of the skin folds of the triceps at 9 mo of age.<br><br>Thompson et al. (7) found no association between reactive feeding practices and WAZ or skin fold thickness using delayed models with measurements at 3, 6, 9, 12, and 18 mo of age. Finally, a study conducted in China, which included parents and grandparents as caregivers, found no association between reactive feeding practices and change in BMIZ from 12 to 18 mo of age in overweight children (31).<br><br>Three studies looked at feeding in the absence of hunger or using food to calm children (12, 32, 33). Stifter et al. (12) found that calming feeding was associated with greater weight gain from 6 to 18 mo of age based on observational measures, but found no association when using parental relationship measures.<br>The other 2 studies found no association between using food to calm or eating in the absence of hunger with change in BMIZ (31) or skin fold thickness (32).<br><br>Two studies investigated indulgent feeding practices, practices that satisfy the baby and offer minimal structure (7, 24). Chaidez et al. (24) found an association between greater indulgent feeding practices and greater changes in WAZ e BMIZ (24), while Thompson et al. (7) found no relationship between indulgent nutrition and WAZ or skin thickness.<br>Ma et al. (31) found that concern about children's food intake was associated with the change in BMIZ between 12 and 18 mo of age in overweight children.<br><br>Dinkevich et al. (25) found an association between mothers who were more concerned about their baby overeating and the likelihood of exhibiting restrictive eating behaviours and having a baby with a higher WLZ. Conversely, there was an association between mothers who were more concerned about their baby not eating and the likelihood of putting pressure on their babies to eat and having babies with a lower WLZ.	Moderate evidence from intervention studies suggests that providing mothers with reactive feeding guidance to recognize and respond appropriately to the baby's hunger and satiety cues can lead to "normal" weight gain and / or state "normal" weight in babies from birth to 24 weeks compared to babies whose mothers did not receive guidance on reactive feeding.<br>Moderate evidence from longitudinal cohort studies indicates an association between maternal feeding practices and infant weight status and / or weight gain, but the direction of effect has not been adequately studied.<br>Restrictive feeding practices are associated with weight gain and increased weight status, while pressing feeding practices are associated with less weight gain and lower weight status<br><br>- Evidence suggests that a mother's feeding practices are linked to concerns about her baby's body weight

		Farrow et al. (8) found that there was an interaction between maternal control; when maternal control was low or moderate, infants with slow weight gain from 0 to 6 mo tended to have accelerated weight gain from 6 to 12 mo, while infants with greater weight gain from 0 to 6 mo they had decelerated weight gain from 6 to 12 mo. When maternal control was high, the opposite was true.<br><br>**<u>Other feeding practices</u>** have not been associated with baby weight outcomes: monitoring (how much the mother tracks the amount of sweets, snacks, and high-fat foods her baby eats) (9), modelling (26), laissez-faire practices (7), and authoritative practices (offers structure, guidance, and positive modelling) (24). Because each of these feeding practices was only examined within a single study, consistency between studies could not be addressed.	

Table a4.14. Included studies: Characteristics and Results

Study	Study design	Population	Intervention/Exposition	Primary Outcome	Secondary Outcomes	Follow-up	Results
Daniels et al. 2012 [11]	RCT in open	698 dyads from primiparous mothers, of healthy infants, from consecutive enrolment	<u>Intervention:</u> 2 modules, with multiple components including responsiveness education, started at the age of 4 and 7 mo and 13-16 mo respectively. Each module included 6 interactive group sessions lasting from 1 to 1.5 hours over 12 weeks, each module followed by 6 monthly contacts via sms or email. Sessions facilitated by dietician and psychologist.<br>Control: standard management at local territorial services	- BMIZ; WAZ; RWG (Rapid Weight Gain)<br>- Food preferences<br><br>- eating behaviour<br><br>- Style and eating behaviour practices	Parental self-efficacy<br><br>Maternal BMI	At 13 mo of life<br>14% lost at follow-up (significantly different socio-demographic characteristics)	At 13 mo of age, the children in the intervention group had a lower BMIz score than the children in the control group: 0.23 ± 0.93 and 0.42 ± 0.85 respectively (p = 0.01)<br><br>Minor weight gain from 0 to 13 mo (OR = 1.6, 95% CI = 1.1 to 2.4; p = 0.008) and from 4 to 13 mo (OR = 1.5, 95% CI = 1.1 to 2.1; p = 0.014) in children of the intervention group
Savage et al. 2016 [13]	RCT in open	291 primiparous mother / healthy infant dyads	The active group received 5 sessions (4 at home within 40 weeks and 1 in outpatient clinic at 1 year) consisting of an intervention with multiple components including Responsibility Education.	ΔBMIZ At 3 years old	For the 1-year-old analysis in this publication:<br>- WLZ	At 1 years old<br><br>15% lost at follow-up	The children in the group of parents who had received the surgery had a lower WLZ at 1 year of life (p = 0.04) and were less likely to be overweight than the children in the control group (p = 0.05)

			The control group received the same number of visits, with standard recommendations		-% Overweight (WLZ ≥95th percentile) at 1 year		
Paul et al. 2018 [12] (follow-up of Savage 2016)	RCT in open	ΔBMIZ At 3 years old	The active group received 7 sessions (4 at home within 40 weeks and 3 in an outpatient clinic at 1, 2 and 3 years) consisting of an intervention with multiple components including Responsibility Education.<br>The control group received the same number of visits, with standard recommendations	ΔBMIZ At 3 years old	BMI z scores<br>Percentile BMI<br>% with accelerated WG at various follow-up times<br><br>% Overweight and Obese at 2 and 3 years (WLZ ≥95th percentile)	At 3 years old<br><br>20% lost at follow-up	ΔBMIZ at 3 years: - 0.28 in the active group (95% CI - 0.53 to -0.01; p = 0.04)<br><br>The differences in secondary outcomes related to growth were not significant<br>2-year BMIZ was -0.09 for the active group compared to 0.11 for the control group (ΔBMIZ absolute difference = -0.21 [95% CI, -0.65 to 0.06]; p = 0.10)

Wright et al. 2006 [14]	Cohort study	Neonatal cohort (n = 1029)	Pressure (Maternal response to food refusal)	Growth in Weight. Weight detected by a nurse at 13 mo. Weight-adjusted analysis, at various ages (Thrive Index)	Appetite, oromotor dysfunction, maternal anxiety during meals	12 mo of life Analysis on < 60%	Less weight gain from birth to 12 mo in infants of mothers who exerted pressure at 12 mo in response to refusal of food (p = 0.002)
Chaidez et al. 2014 [15]	Cohort study	94 mothers of children aged 12-24 mo	Indulgent<br><br>Authoritative	ΔWHZ<br>ΔWAZ<br>ΔBMIZ<br>Weights measured by staff	/	6 mo from the baseline Analysis on 36%	“Lenient” practices associated with greater changes in parameters in the period from 22 mo to 28 mo<br>DWHZ: p = 0.03<br>DBAZ: p = 0.05<br>DWAZ: p = 0.04.
Dinkevich et al. 2015 [16]	Cohort study	231 children belonging to a local clinic	Restriction<br><br>Pressure<br><br>Concern about under/overweight	Weight for length Zscore (WLZ)	/	At 30 mo of life Analysis on 27%	Positive association of restrictive feeding with higher WLZ from 6 to 30 mo of life (p = 0.036)<br>Association of Pressuring to Eat with lowest WLZ between 6 and 30 mo (p = 0.034)<br>Positive association between Concern for undereating and for Weight and greater WLZ (p = 0.011)<br>Concern style for overeating and for Weight significant predictor for weight gain (p = 0.008)
Hittner et al. 2016 [17]	Cohort study	Secondary analysis of a cohort study of 86 dyads (34% of a group of adoptive families)	Restriction	ΔBMI (including interactions with sex)		At 6 years old (% not explicated)	Significant association between mothers restrictive style at 1 year and changes in BMI from 2 to 6 years of life; in males, restrictions of varying intensity were associated with a decrease in BMI between 2 and 6 years, in females with an increase in BMI (see text)
Stifter et al. 2015 [18]	Cohort study	Neonatal cohort<br>160 dyads	Food to Soothe	Growth in weight from 6 to 18 mo (measured by staff)	/	At 18 mo of life Analysis on 16%	Positive association between the use of food to calm the baby at 6 mo and weight gain between 6 and 18 mo (p <0.001)

A4 . EVIDENCE PROFILE GRADE

a4. ReCF / nReCF. Growth

Table a4.15. ReCF. Growth.

[ReCF] compared to [other models of CF] in [healthy child, in the period 6-24 months], can influence, in positive or negative way, [the process of staturo-weight growth in later age]

Patient or population [healthy child in the period 6-24 months, in positive or negative way, the process of staturo-weight growth in later age]

Setting: Outpatient

Intervention: [ReCF]

Comparator: [other models of CF]

Certainty assessment							N of patient		Outcomes		Certainty	Importance
N of studies	Study design	Risk of distortion	Lack of reproducibility of results	Lack of generalizability	Imprecision	Further considerations	[ACRe]	[other models di CF]	Relative (95% CI)	Absolute (95% CI)		
**Growth 0-12 months. RCT (evaluated with: BMIZ, WLZ)**												
2 [1,2]	Randomized studies	Very serious [a]	Not important	Serious [b]	Not important	All plausible residual confounders could reduce the demonstrated effect	DANIELS 2012 Children in the intervention group had a lower BMIz score at 13 months of life compared to children in the control group: 0.23 ± 0.93 and 0.42 ± 0.85 respectively (p = 0.01) Less weight gain from 0 to 13 mo (OR = 1.6 , 95% CI = 1.1 to 2.4; p = 0.008) and 4 to 13 mo (OR = 1.5, 95% CI = 1.1 to 2.1; p = 0.014) in children of the SAVAGE 2016 intervention group.<br><br>The children of the parent group who had received the surgery had a lower WLZ at 1 year of age (p = 0.04)				⊕⊕◯◯ LOW	CRITICAL
**Growth. RCT (follow up: 3 years; evaluated with: ΔBMIZ)**												
1 [3]	Randomized studies	Serious [c,d]	Serious [c,d]	Not important	Not important	None	352	346	-	MD 0.19 Lower BMIZ<br><br>(0.32 less than 0.06 less)	⊕⊕◯◯ LOW	CRITICAL

CI: Confidence interval; **MD:** Mean difference

Explanations

a. Performance uncertainty (performance bias): the instructions provided to the caregivers of the active groups regarding ReCF were not the only dates, but were part of a multi-component intervention, with general instructions on the overall care of children; however, no instructions or information on nutritional aspects are described

b. In the SLIMTIME study and in the INSIGHT study, the interventions were initiated in times prior to the period of the CF, thus determining a condition of poor inherence (indirectness) since the effectiveness of the intervention may have been determined on a population that is not yet had reached the age of CF.

c. Loss to follow-up limit (20%)

d. Unique study

References

1. Daniels LA, Mallan KM,Battistutta D,et al.. Evaluation of an intervention to promote protective infant feeding practices to prevent childhood obesity: outcomes of the NOURISH RCT at 14 months of age and 6 months post the first of two intervention modules.. Int J Obes (Lond). 2012;36(10):1292-8; 2012.

2. Savage JS, Birch LL,Marini M,Anzman-Frasca S,Paul IM. Effect of the INSIGHT responsive parenting intervention on rapid infant weight gain and overweight status at age 1 year: a randomized clinical trial. JAMA Pediatr. 2016, 170(8):742-9, . Effect of the INSIGHT responsive parenting intervention on rapid infant weight gain and overweight status at age 1 year: a randomized clinical trial. . JAMA Pediatr. 2016;170(8):742-9; 2016.

3. Paul IM, Savage JS,Anzman-Frasca S,et al.. Effect of a Responsive Parenting Educational Intervention on Childhood Weight Outcomes at 3 Years of Age: The INSIGHT Randomized Clinical Trial. . JAMA. 2018;320(5):461-8; 2018.

Table a4.16. nReCF. Growth

[nReCF] compared to [other models of CF] in [healthy child, in the period 6-24 months, can influence, in positive or negative way, the process of staturo-weight growth in later age]

Patient or population: [healthy child, in the period 6-24 months, can influence, in positive or negative way, the process of staturo-weight growth in later age]

Setting: Outpatient

Intervention: [nReCF]

Comparator: [other models of CF]

Certainty assessment							Impact	Certainty	Importance
N of studies	Study design	Risk of distortion	Lack of reproducibility of results	Lack of generalizability	Imprecision	Further considerations			
**nReCF-Growth (follow up: interval 12 months to 6 years; evaluated with: BMIZ, WLZ, ΔBMI, ΔP)**									
5 [1,2,3,4,5]	Observational studies	Very serious [a,b]	Serious [a]	Not important	Not important	Strong association<br><br>All plausible residual confounders could reduce the demonstrated effect	Discordant data.<br>In one study, “lenient” practices are associated with greater changes in parameters over the period from 22 mo to 28 mo<br>DWHZ: p=0.03<br>DBAZ: p=0.05<br>DWAZ: p=0.04<br>In another study Positive association of restrictive feeding with higher WLZ from 6 to 30 mo of life (p = 0.036)<br>Association of Pressuring to Eat with lowest WLZ between 6 and 30 mo (p = 0.034)<br>In another study, significant association between mothers restrictive style at 1 year and changes in BMI from 2 to 6 years of life	⨁◯◯◯<br>VERY LOW	CRITICAL

CI: Confidence interval

Explanations

a. Discordant results

b. Low sample size

References

1. Wright CM, Parkinson KN,Drewett RF.. ow does maternal and child feeding behavior relate to weight gain and failure to thrive? Data from a prospective birth cohort. . HPediatrics. 2006;117(4):1262-9; 2006.
2. Chaidez V, McNiven S,Vosti SA,Kaiser LL.. Sweetened food purchases and indulgent feeding are associated with increased toddler anthropometry.. J Nutr Educ Behav. 2014;46(4):293-8; 2014.
3. Dinkevich E, Leid L,Pryor K,et al.. Mothers' feeding behaviors in infancy: do they predict child weight trajectories?. Obesity (Silver Spring). 2015;23(12):2470-6; 2015.
4. Hittner JB, Johnson C,Tripicchio G,Faith MS.. Infant emotional distress, maternal restriction at a home meal, and child BMI gain through age 6 years in the Colorado Adoption Project. . Eat Behav. 2016;21:135-41; 2016.
5. Stifter CA, Moding KJ.. Understanding and measuring parent use of food to soothe infant and toddler distress: A longitudinal study from 6 to 18 months of age. . Appetite. 2015;95:188-96; 2015.

A4. ReCF / nReCF and risk of overweight/obesity

PICOs

A.
P. Healthy child aged 6-24 months
I. Responsive Complementary Feeding
C. Compared to others feeding models
O. Does it involve a different risk of development overweight and obesity in later age?

B.
P. Healthy child aged 6-24 months
I. Non-responsive Complementary Feeding
C. Compared to others feeding models
O. Does it involve a different risk of development overweight and obesity in later age?

KEY WORDS

Population

M. No age limit
N. ([infant]/lim OR [child]/lim OR [preschool]/lim

Exposure Factors / Comparison

M. Infant Nutritional Physiological Phenomena [MeSH]
N. Weaning"[MeSH])
O. "Feeding Behavior"[MeSH]
P. ("Feeding Methods"[MeSH]
Q. "feeding practice"[All Fields]
R. “parenting style”
S. “feeding style” [All Fields]
T. “feeding patterns” [All Fields]
U. “responsive feeding” [All Fields]
V. “non responsive feeding” ”[All Fields]
W. “responsiveness”[All Fields]
X. “complementary feeding”[All Fields]

Outcomes
"Body Height"[Mesh])
"Body Weight"[Mesh])
"Body-Weight Trajectory"[Mesh])
"Body Weight Changes"[Mesh]
"Body Composition"[Mesh])
"Nutritional Status"[Mesh]
("Obesity"[Mesh]
"Pediatric Obesity"[Mesh])
"Overweight"[Mesh]

Guidelines search

PUBMED

#1

("Obesity"[Mesh] OR "Pediatric Obesity"[Mesh] OR "Overweight"[Mesh] OR "Body Mass Index"[Mesh] OR "Body Weight Changes"[Mesh] OR "Body Weight"[Mesh] OR "Body Composition"[Mesh] OR "Nutritional Status"[Mesh]) AND ((Practice Guideline[ptyp] OR Guideline[ptyp]) **AND** ("2014/09/15"[PDat] : "15.03.2021"[PDat])

EMBASE

#1

('obesity'/exp OR obesity) AND [2014-2021]/py AND 'practice guideline'/de AND ([child]/lim OR [infant]/lim)

SOCIETY GUIDELINE LINKS:

National Guideline Clearinghouse (NGC) https://www.ahrq.gov/gam/index.html

Canadians Medical Association (CMA) https://www.cma.ca/clinicalresources/practiceguidelines

National Guideline Centre (NGC) - National Institute of Health and Care Excellence (NICE) https://www.rcplondon.ac.uk/about-us/what-we-do/national-guideline-centre-ngc

Scottish Intercollegiate Guidelines Network (SIGN) https://www.sign.ac.uk/our-guidelines.html

Australian Clinical Practice Guidelines (ACPG) https://www.clinicalguidelines.gov.au/

New Zealand Guidelines Group (NZGG) https://www.health.govt.nz/about-ministry/ministry-health-websites/new-zealand-guidelines-group

American Academy of Pediatrics (AAP) https://www.aap.org/en-us/Pages/Default.aspx

EPA/UNEPSA http://www.epa-unepsa.org/

Guidelines International Network https://g-i-n.net/

European Childhood Obesity Group https://www.ecog-obesity.eu/

Società Italiana di Pediatria (SIP) http://www-sip.it/

Società Italiana di Pediatria Preventiva e Sociale (SIPPS) https://www.sipps.it/

Società Italiana di Endocrinologia e Diabetologia Pediatrica (SIEDP) http://www.siedp.it/pagina/84/linee+guida%2C+raccomandazioni+e+consensus

Systematic Reviews search

COCHRANE LIBRARY

'obesity’ in Title Abstract Keyword'
Custom date range Topics: 01.09.2014-15.03.2021

EMBASE

#1

('obesity'/exp OR 'body mass'/exp OR overweight) AND ('complementary feeding'/exp OR 'weaning'/exp OR 'responsiveness' OR 'responsive feeding' OR 'non responsive feeding') AND ([cochrane review]/lim OR [systematic review]/lim OR [meta analysis]/lim) AND [2014-2021]/py

PUBMED

#1

(((((((("Obesity"[Mesh] OR "Pediatric Obesity"[Mesh]) OR "Overweight"[Mesh]) OR "Body Mass Index"[Mesh]) OR "Body Weight Changes"[Mesh]) OR "Body Weight"[Mesh]) OR "Body Composition"[Mesh]) OR "Nutritional Status"[Mesh]) AND ("responsive feeding"[All Fields] OR "non-responsive feeding"[All Fields] OR “responsiveness”[All Fields] OR "Weaning"[All Fields] OR "Infant Nutritional Physiological Phenomena"[MeSH] OR “complementary feeding”[All Fields] OR "Feeding Behavior"[All Fields]) AND (Meta-Analysis[ptyp] OR systematic[sb]) **AND** ("2014/09/15"[PDat] : "2021/03/15"[PDat])

Studies search (**subsequent to SR of Spill et al. - January 2017)**

PUBMED

#1

(incentiv* OR indulgen*[tiab] OR authorita*[tiab] OR reward* OR control* OR pressur* OR restrict* OR monitor* OR respons* OR sooth*[tiab] OR encourag* OR discourage* OR uninvolv* OR disengage* OR parenting style* OR laissez-faire OR laissez faire* OR non-respons* OR nonrespons* OR force*) AND (feeding* OR fed[tiab] OR eat[tiab] OR eating OR "Feeding Methods"[Mesh:noexp] OR "Feeding Behavior"[Mesh:NoExp] OR satiety OR hunger OR hungry OR satiat*) AND (cue OR cues OR feeding method* OR feeding practice* OR feeding pattern* OR feeding frequenc* OR infant feed* OR feeding behavior*[tiab] OR feeding style* OR feeding strategy*) AND (Randomized Controlled Trial[ptyp] OR Pragmatic Clinical Trial[ptyp] OR Observational Study[ptyp] OR Multicenter Study[ptyp] OR Comparative Study[ptyp] OR Clinical Trial[ptyp] OR Controlled Clinical Trial[ptyp]) AND ("2017/01/01"[PDAT] : "2021/03/15"[PDAT]) AND ("infant"[MeSH Terms]).

#2

("responsive feeding"[All Fields] OR "non-responsive feeding"[All Fields] OR "responsiveness"[All Fields] OR "Weaning"[All Fields] OR "Infant Nutritional Physiological Phenomena"[MeSH] OR "complementary feeding"[All Fields] OR "Feeding Behavior"[All Fields]) AND ((((((("Obesity"[Mesh] OR "Pediatric Obesity"[Mesh]) OR "Overweight"[Mesh]) OR "Body Mass Index"[Mesh]) OR "Body Weight Changes"[Mesh]) OR "Body Weight"[Mesh]) OR "Body Composition"[Mesh]) OR "Nutritional Status"[Mesh]) AND (Clinical Trial[ptyp] OR Comparative Study[ptyp] OR Controlled Clinical Trial[ptyp] OR Observational Study[ptyp] OR Pragmatic Clinical Trial[ptyp] OR Randomized Controlled Trial[ptyp]) AND ("2017/01/01"[PDAT] : "2021/03/15"[PDAT])

EMBASE

#1

('complementary feeding'/exp OR ‘weaning OR 'responsiveness' OR 'responsive feeding' OR 'non responsive feeding') AND ('obesity'/exp OR 'body mass'/exp OR obesity) AND [2017-2021]/py AND ('case control study'/de OR 'clinical study'/de OR 'clinical trial'/de OR 'cohort analysis'/de OR 'comparative effectiveness'/de OR 'comparative study'/de OR 'controlled clinical trial'/de OR 'controlled study'/de OR 'cross-sectional study'/de OR 'double blind procedure'/de OR 'human'/de OR 'multicenter study'/de OR 'observational study'/de OR 'prospective study'/de OR 'randomized controlled trial'/de OR 'retrospective study'/de)

Figure a4.14. Guideline search flow diagram

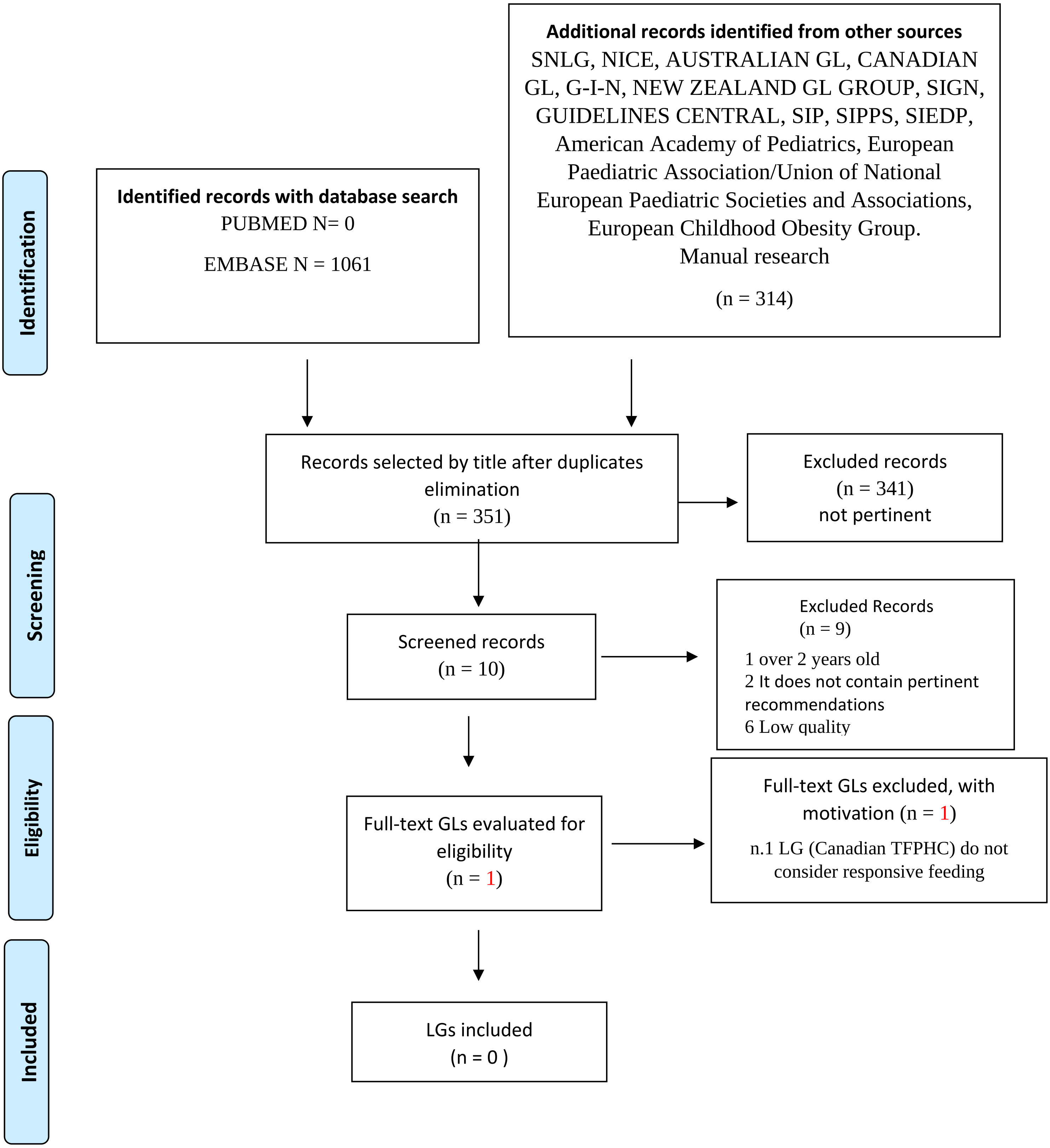

Figure a4.15. SRs search flow diagram

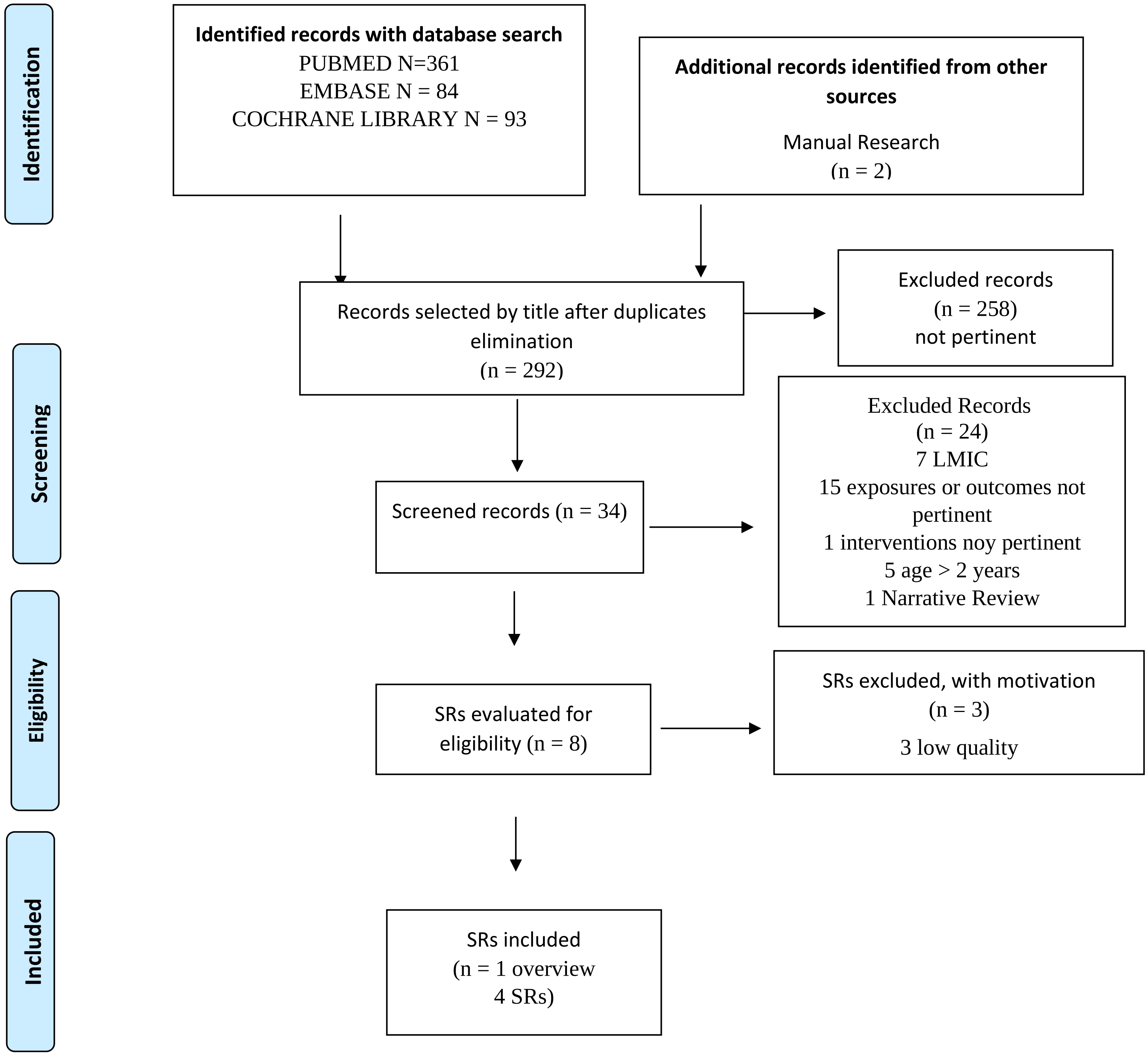

Figure a4.16. Studies flow diagram

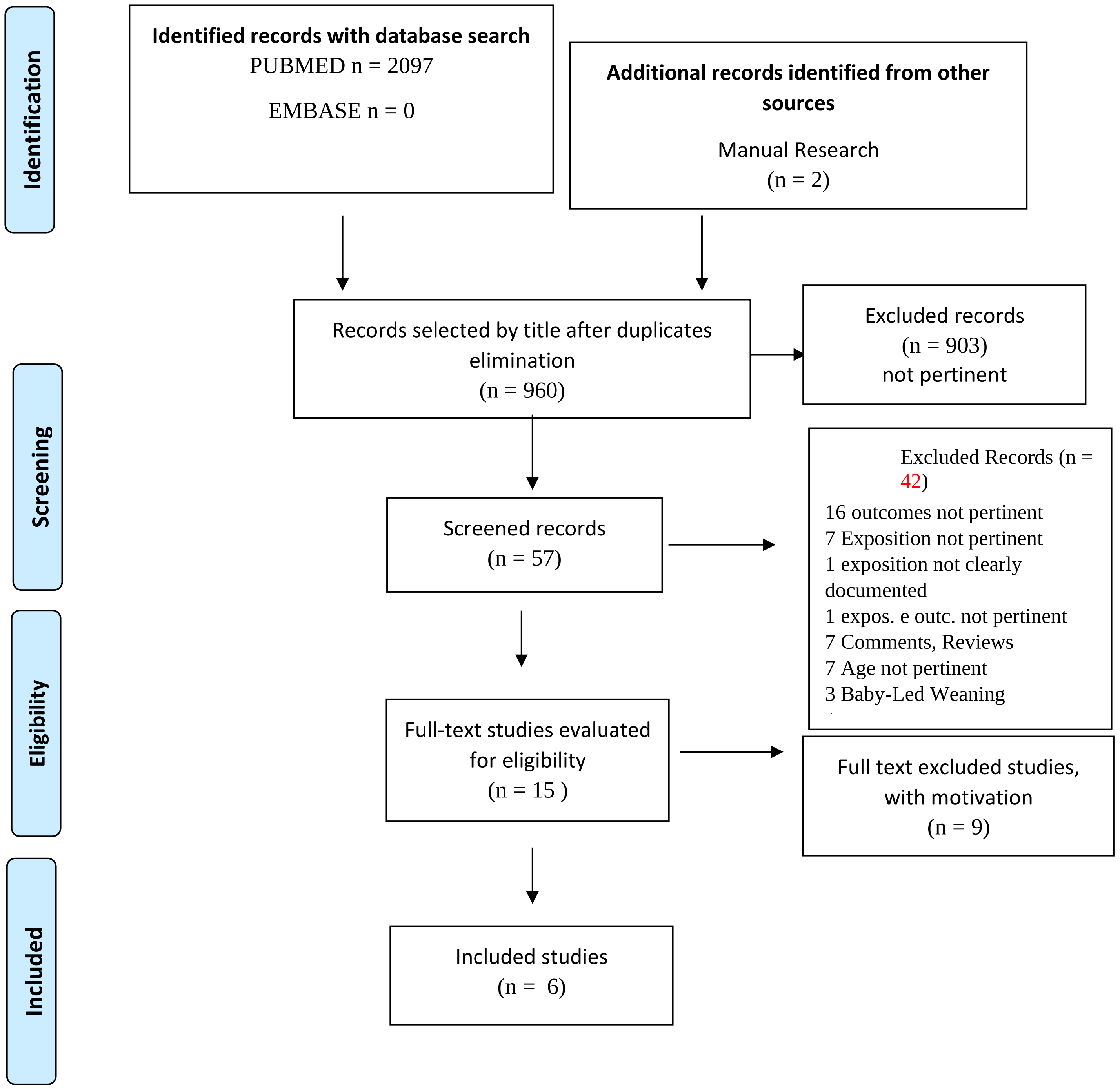

A4. METHODOLOGICAL ASSESSMENT

A4. ReCF/nReCF and risk of overweight/obesity

Table a4.17. Clinical Guidelines and Documents excluded.

Excluded GLs	Multidisciplinarity of the panel	Systematic search for evidence	Grading of recommendations	Reason for exclusion
Canada's Dietary Guidelines 2018 [25]	Limited to Nutritionists and Public Health Experts	NO	NO	Low methodological quality<br>It does not contain pertinent recommendations
Dereń et al. EAP ECOG 2019 [26]	NO	NO	NO	Low methodological quality
Fewtrell et al.. ESPGHAN 2017. Complementary feeding [27]	NO	Declared but not published	NO	Low methodological quality
Koletzko et al. 2019 The Early Nutrition Project Recommendations [28]	YES	YES but not explicated (they use SR already published: for the questions of this Consensus Patro-Golab et al. 2016)	NO Consent vote	Low methodological quality
NICE 2015 Preventing excess weight gain [29]	===	====	===	Interventions subsequent to the period of the CF
Romero-Velardea et al. 2016. Alimentation complementaria [30]	Limited to Pediatricians and Nutrition Experts	NO	NO	Low methodological quality
Schwarzenberg et al. 2018. AAP Policy Statement [31]	NO	NO	NO	Low methodological quality
USDA 2015-2020 [32]	YES	YES	Related to the quality of the evidence	It does not contain pertinent recommendations
SIEDP-SIP 2018 Obesity[33]	YES	NO, only MEDLINE	YES	Moderate methodological quality. It does not report relevant recommendations
Heyman et al. 2017 [34]	No	No	No	Low methodological quality.

Table a4.18. Appraisal of the Systematic Review Overviews

**COCHRANE TOOL FOR OVERVIEWS OF REVIEWS**		Gerritsen et al. 2017 [9]
Objective	To summarize evidence from *systematic reviews* examining effects of interventions	Appropriate
Selection criteria	Describe inclusion and exclusion criteria for review	Appropriate
Search	Typically, look only for relevant Cochrane intervention reviews.	Appropriate (RCT and Cohort Studies if recent and important)
Data collection	From the included systematic review	Appropriate
Assessment of limitation	For included systematic review	Appropriate
Quality of the evidences	As far as possible it should be based on evaluation reported in the included systematic review	Appropriate
Analysis	Summary of the results of the reviews; further analysis can be undertaken for comparisons between reviews, typically indirect comparisons of multiple interventions.	Appropriate (Summaries of results almost always only narrative)
**OVERALL EVALUATION**	**Good methodological quality.**	

Table a4.19. Appraisal of the Systematic Reviews.

**AMSTAR 2**	Redsell et al. 2016 [35]	Sokol et al. 2017 [36]	Matvienko-Sikar et al. 2018 [37]	Spill et al. 2019 [10]
**1. Did the research questions and inclusion criteria for the review include the components of PICO? (Yes/No)**	YES	NO	YES	YES
**2. Did the report of the review contain an explicit statement that the review methods were established before the conduct of the review and did the report justify any significant deviations from the protocol? (Yes/Partial Yes/No)**	YES	NO	PARTIAL YES	YES
**3. Did the review authors explain their selection of the study designs for inclusion in the review? (Yes/No)**	NO	YES	NO	YES
**4. Did the review authors use a comprehensive literature search strategy? (Yes/Partial Yes/No)**	PARTIAL YES	PARTIAL YES	PARTIAL YES	PARTIAL YES
**5. Did the review authors perform study selection in duplicate? (Yes/No)**	NO	YES	YES	YES
**6. Did the review authors perform data extraction in duplicate?(Yes/No)**	YES	YES	YES	YES
**7. Did the review authors provide a list of excluded studies and justify the exclusions?**<br>**(Yes/Partial Yes/No)**	NO	NO	NO	YES
**8. Did the review authors describe the included studies in adequate detail?**<br>**(Yes/Partial Yes/No)**	NO	YES	YES	PARTIAL YES
**9. Did the review authors use a satisfactory technique for assessing the risk of bias (RoB) in individual studies that were included in the review?**<br>**(Yes/Partial Yes/No/Includes only NRSI-RCT)**	NO	YES	YES<br>YES	PARTIAL YES<br>YES

**10. Did the review authors report on the sources of funding for the studies included in the review?(Yes/No)**	NO	NO	NO	YES
**11. If meta-analysis was performed did the review authors use appropriate methods for statistical combination of results? (Yes / No / No meta-analysis conducted)**	No Meta-Analysis	No Meta-Analysis	No Meta-Analysis	No Meta-Analysis
**12. If meta-analysis was performed, did the review authors assess the potential impact of RoB in individual studies on the results of the meta-analysis or other evidence synthesis? (Yes / No / No meta-analysis conducted)**	No Meta-Analysis	No Meta-Analysis	No Meta-Analysis	No Meta-Analysis
**13. Did the review authors account for RoB in individual studies when interpreting/ discussing the results of the review? (Yes/No)**	YES	YES	NO	YES
**14. Did the review authors provide a satisfactory explanation for, and discussion of, any heterogeneity observed in the results of the review? (Yes/No)**	YES	YES	YES	YES
**15. If they performed quantitative synthesis did the review authors carry out an adequate investigation of publication bias (small study bias) and discuss its likely impact on the results of the review? (Yes / No / No meta-analysis conducted)**	No Meta-Analysis	No Meta-Analysis	No Meta-Analysis	No Meta-Analysis
**16. Did the review authors report any potential sources of conflict of interest, including any funding they received for conducting the review? (Yes/No)**	YES	YES	YES	YES
**OVERALL EVALUATION**	**LOW QUALITY**	**LOW QUALITY**	**LOW QUALITY**	**MODERATE QUALITY**

Table a4.20. SRs excluded with motivation.

ESCLUDED	Reason for exclusion
Blake Lamb et al. 2016 (preventive interventions) [38]	Low quality (3 AMSTAR-2 critical items failed)
Bonilla et al. 2017 (SRs and studies overview) [39]	Low quality (Cochrane tool: 4 items on 7 not completely fulfilled)
Woo Baidal et al. 2016 (Risk factor) [40]	Low quality (3 AMSTAR-2 critical items failed)

Figure a4.17. Risk of bias summary: review authors' judgements about each risk of bias item for each included study. [41,12,13]

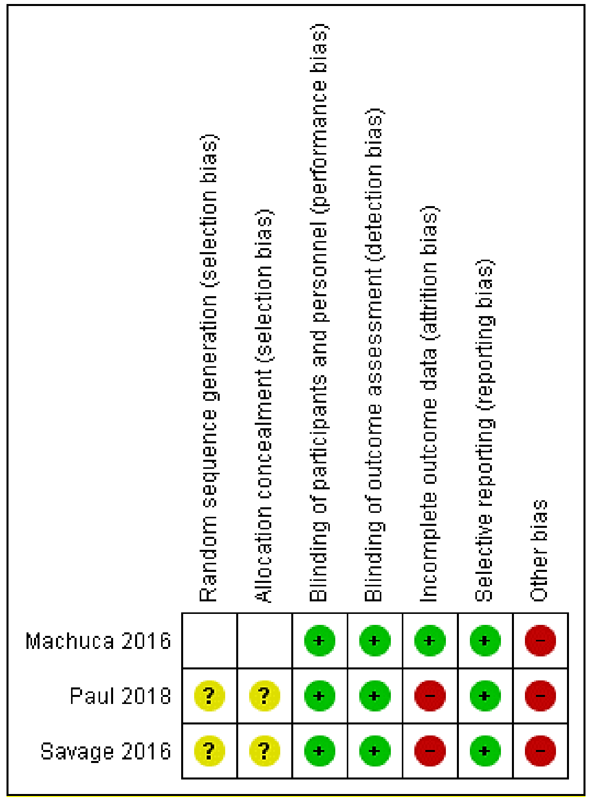

Figure a4.18. Risk of bias graph: review authors' judgements about each risk of bias item presented as percentages across all included studies

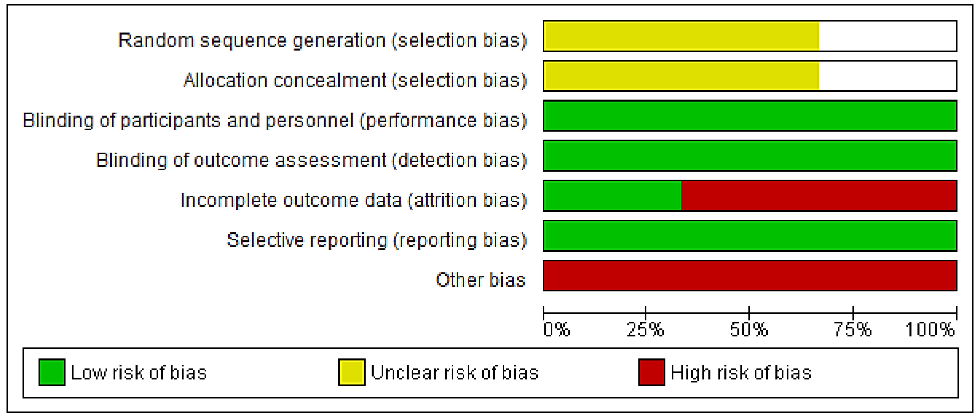

Table a4.21. Appraisal of the Studies

	Newcastle Quality Assessment Scale COHOTS STUDIES								
	**Selection**				**Comparability**	**Outcome**			
**Study**	**Representativeness of the exposed cohort**	**Selection of non-exposed cohort**	**Ascertainment of the exposure**	**Demonstration that the outcome of interest is not present at the start of the study**	**Comparability of cohorts based on design or analysis**	**Outcome evaluation**	**Was the follow-up long enough for the outcome to occur?**	**Adequacy of cohort follow-up**	**Total**
Rifas-Shiman et al. 2011 [42]	1a	1a	d	1a	1a 1b	1a	1a	1a 47% (description of the characteristics of the lost)	**8** **Good quality +**
Lumeng et al. 2012 [43]	1b	1a	1a	b	1a 1b	1a	1a	c 11% (no analysis on the lost)	**7** **Good quality**
Thompson et al. 2013 [44]	c	1a	d	b (BMI≤25 only in 26,7%)	1b	1a	1a	c 36% (no analysis on the lost)	**4** **Low quality**

Table a4.22. Excluded studies with motivation

EXCLUDED STUDIES	Reason for exclusion
Paul et al. 2011 [19]	Low methodological quality (Loss to follow-up > 20%)
Daniels et al. 2012 [11]	Not pertinent (overweight and obesity are not outcomes of the study)
Daniels et al. 2013 (follow-up di Daniels et al. 2012) [20]	Low methodological quality (Loss to follow-up > 20%)
Daniels et al. 2015 (follow-up di Daniels et al. 2012) [21]	Low methodological quality (Loss to follow-up > 20%)
Morandi et al. 2019 [45]	Low methodological quality (Loss to follow-up > 20%)
Farrow et al. 2008 [46]	Not pertinent (overweight and obesity are not outcomes of the study)
Gregory et al. 2011 [47]	Low methodological quality. Not pertinent (overweight and obesity are not outcomes of the study)
Dinkevich et al. 2015 [16]	Not pertinent (overweight and obesity are not outcomes of the study)
Shi et al. 2017 [24]	Not pertinent (conducted in China)

A4. RECOMMENDATIONS OF GLs, RESULTS IN SRs AND STUDIES

A4. ReCF / nReCF . Risk of overweight and obesity

- ***Can ReCF influence the development overweight and obesity in later age?***<br><br>- ***Can nReCF influence the development overweight and obesity in later age?***	**A**.<br>**P.** Healthy child aged 6-24 months<br>**I.** Responsive Complementary Feeding<br>**C.** Compared to others feeding models<br>**O.** Does it involve a different risk of development overweight and obesity in later age?<br><br>**B.**<br>**P.** Healthy child aged 6-24 months<br>**I.** Non-responsive Complementary Feeding<br>**C.** Compared to others feeding models<br>**O.** Does it involve a different risk of development overweight and obesity in later age?

Table a4.23. Included SRs: Characteristics, Results, and Conclusions.

**Systematic Review**	**Population and purpose of the SR**	**Results**	**Conclusions**
Redsell et al. 2016 [35]	Children under 2 years of age<br>Dedicated to active interventions aimed at intercepting the most significant risk factors previously identified (Wang 2013)<br><br>Outcomes: overweight and obesity at the age of 7	27 works<br>24 behavioural interventions of which 12 nutritional and 3 responsive feeding Paul 2011, Daniel 2012; 3 nutritional interventions; age less than 2 mo<br>Educational components for Responsive Nutrition	Interventions aimed at improving the nutritional practices of parents that include parental responsiveness in addition to the diet show promise in inducing changes in behaviour, but not so much in inducing a reduction in overweight

Sokol et al. 2017 [36]	Children of various ages<br>Prospective observational studies<br>Responsive and non-responsive, authoritative and authoritative styles	1 single study in which exposures were documented under 2 years of life (Ref. # 38)	Conflicting results seem to indicate that "authoritative" parenting styles are protective against the development of Overweight and Obesity in later life
Matvienko-Sikar et al. 2018 [37]	Children under 2 years of age<br>RCT only<br>Early educational interventions to obtain changes in the feeding styles of caregivers<br>Weight = secondary outcome of the review	6 studies related to responsive nutrition, 3 of which are not pertinent (French 2012: on the use of multi-intervention anticipatory guides - Gross 2016: multiple early interventions - Schroeder 2015: early interventions, especially nutritional)<br>Only 3 really relevant<br>(Daniels et al., 2012, 2013, 2014, 2015; Hohman et al., 2017 INSIGHT; Paul et al., 2011).<br>In RCTs that involved interventions with Power components<br>Responsive, low quality evidence for a short-term positive effect on weight outcomes, effect progressively lost in the 3-5 year follow-up (see Spill 2019)	Low-level evidence for the effects of educational interventions in the first 2 years of life on weight outcomes
Spill et al. 2019 [10]	<u>Population</u> consisting of dyads *Parent-caregiver* and *infant-toddler*.<br><u>Purpose</u>: know the relationship between the different nutrition practices (of control, constriction, restriction, monitoring, responsiveness and non-responsiveness to the manifestations of hunger and satiety of the child) and the growth, size and body composition of children.<br><u>Studies</u>: controlled, randomized and non-randomized, prospective and retrospective observational studies, pre-post controlled studies, nested-case-case-control studies.	Responsive feeding.<br>In the RCT by Daniels et al. there was a difference in weight results at 13.5 mo; however, there were no significant differences in weight gain indicators at 20 mo or 4.5 years of follow-up between the intervention and control groups.<br>The remaining 2 controlled studies, 1 RCT (Kavanagh 2008) and 1 non-randomized controlled study (De Carvalho M, 1983), had limitations that made them less informative to answer the question of the systematic review.<br>Pressure to finish food at 3 mo of age was associated with lower WAZ and lower odds of WAZ> 90th percentile at 3, 6, 9, 12, and 18 mo of age (Thompson, 2013). Pressure to eat was associated with a lower WLZ in children aged 6 to 30 mo (Dinkevich, 2015) and pressure to eat at 1 year of age predicted a weight of less than 2 years (Farrow, 2008) .<br>After adjusting for the initial weight, Taveras et al. (Taveras 2006) found an association between maternal restriction and an increased likelihood of having a BMIZ between the 85th and 95th percentiles, but Rifas-Shiman et al. (Rifas-Shiman 2011) found no association between maternal restriction and an increased likelihood of having a BMIZ> 95th percentile. On the other hand, Farrow et al. (Farrow, 2008) found that greater restriction was associated with lower standardized weight scores.<br>Of the studies that found no association, 2 examined the difference between feeding on demand from the infant versus feeding on a fixed schedule (Gubbels 2011, Saxon	Moderate evidence from intervention studies suggests that providing mothers with responsive feeding guidance to recognize and respond appropriately to their baby's cues of hunger and satiety can lead to "normal" weight gain and / or health of "normal" weight in infants from birth to 24 mo compared to infants whose mothers did not receive responsive feeding guidance.<br>Moderate evidence from longitudinal cohort studies indicates an association between maternal feeding practices and infant weight status and / or weight gain, but the direction of effect has not been adequately studied.<br>Restrictive feeding practices are associated with weight gain and increased weight status, while pressing feeding practices are associated with less weight gain and lower weight status.<br>Evidence suggests that a mother's feeding practices are linked to concerns about her baby's body weight

		2002). Morris et al. (Morris 1982) found no association between on-demand feeding practices at 3 mo of age and the thickness of the skin folds of the triceps at 9 mo of age.<br><br>Thompson et al. (Thompson, 2013) found no association between reactive feeding practices and WAZ or skin fold thickness using delayed models with measurements at 3, 6, 9, 12 and 18 mo of age.<br><br>The other 2 studies found no association between or skinfold thickness (Morris 1982).<br><br>Two studies investigated indulgent feeding practices that satisfy the child (Thompson, 2013, Chaidez 2014).<br><br>Chaidez et al. found an association between greater indulgent feeding practices and greater changes in WAZ and BMIZ (Chaidez<br><br>2014), while Thompson et al. (Thompson, 2013) found no relationship between indulgent nutrition and WAZ or skin fold thickness.<br><br>Other feeding practices have not been associated with baby weight outcomes: monitoring (how much the mother tracks the amount of sweets, snacks and high-fat foods her baby eats) (Farrow, 2009), modelling (Gregory, 2011), laissez-faire practices (Thompson, 2013), and authoritative practices (offers structure, guidance, and positive modelling) (Chaidez, 2014). Because each of these feeding practices was only examined within a single study, consistency between studies could not be addressed.	

Table a4.24. Included studies: Characteristics and Results

Study	Study design	Population	Test	Primary Outcome	Secondary Outcomes	Follow-up	Results
Savage et al. 2016 [13]	RCT in open	291 dyads from primiparous mothers, of healthy infants	The active group received 5 sessions (4 at home within 40 weeks and 1 in outpatient clinic at 1 year) consisting of an intervention with multiple components including education a Responsiveness.	ΔBMIZ at 3 years	For 1-year-old analyses in this publication:<br>- WLZ	At 1 years of life<br><br>**15%** lost at follow-up	The children in the group of parents who had receiv[illegible] the surgery had a lower WLZ at 1 year of life (p = 0.[illegible] and were less likely to be overweight than the childr[illegible] in the control group (p = 0.05).

			The control group received the same number of visits, with standard recommendations		-% Overweight (WLZ ≥95th percentile) at 1 year		
Paul et al. 2018 [13]<br>(follow-up di Savage et al. 2016)	RCT in open		The active group received 7 sessions (4 at home within 40 weeks and 3 in the outpatient clinic at 1, 2 and 3 years) consisting of an intervention with multiple components including education at Responsiveness.<br>The control group received the same number of visits, with standard recommendations	ΔBMIZ at 3 years	-% Overweight and Obese at 2 and 3 years (WLZ ≥95th percentile)<br>-BMI z scores<br>-BMI percentile<br>-% with accelerated WG at various follow-up times	At 3 years of life<br>20% lost at follow-up	ΔBMIZ at 3 years: - 0.28 in the active group (95% -0.53 to -0.01; p = 0.04)<br>The differences for all secondary outcomes were n significant
Machuca et al. 2016 [41]	Controlled study	187 children's dyads<br>Mothers chose which group to belong to	The children in the active group received 3 additional 2-hour group sessions with instructions on Responsive Feeding<br>The children in the active group received 3 additional 2-hour group sessions with instructions on Responsive Feeding	Overweght at 2 years (BMI ≥85° percentile)		The follow-up is not disclosed	Less likely to be overweight in children in the active group (2.1% vs. 15.0%; p = 0.02; OR 0.12; 95% CI 0.02-0.94)
Rifas-Shiman et al. 2011 [42]	Cohort study	1579 dyads enroled before birth	Restriction	Obesity at 3 years (BMI ≥95° percentile):	(Neonatal weight)<br>Subscapular folds<br>Triceps folds	Loss 47% at 3 years	The use of a restrictive style at the age of one year was not significantly associated with a higher probability of obesity at 3 years after adjustment for WLZ at 1 year
Lumeng et al. 2012 [43]	Cohort study	1364 dyads (from a larger population study) enroled from birth	Pressure to eat	BMIZ at 3 years	/	Loss 11% between 15 mo and 3 years	The use of a constricting style at the age of 15 mo w not significantly associated with a higher probability of obesity at 3 years after various adjustments
Thompson et al. 2013 [44]	Cohort study	217 dyads enroled at 3 mo	Responsive<br>Restriction<br>Indulgent<br>Laissez-faire<br>Pressure to eat	WAZ at 3, 6, 9, 12, 18 mo of life	Skinfold thickness	Loss36% at 18 mo	No significant association for all comparisons (for documented exposures ≥6 mo)

A4 . EVIDENCE PROFILE GRADE

A4. ReCF / nReCF. Risk of overweight and obesity

Table a4.25. ReCF. Risk of overweight and obesity

[ReCF] compared to [other models of CF] in [healthy child, in the period 6-24 months], can influence, in positive or negative way, [the development overweight and obesity in later age

Patient or population [healthy child in the period 6-24 months, in positive or negative way the development overweight and obesity in later age]

Setting: Outpatient

Intervention: [ReCF]

Comparator: [other models of CF]

Certainty assessment							N of patient		Outcomes		Certainty	Importance
N of studies	Study design	Risk of distortion	Lack of reproducibility of results	Lack of generalizability	Imprecision	Further considerations	[ACRe]	[other models di CF]	Relative (95% CI)	Absolute (95% CI)		
**Risk of overweight and obesity after 2 years. RCT (follow up: 3 years; assessed with:% of overweight / obesity children)**												
2 [1,2]	Randomized studies	Serious [a,b]	Serious [c]	Not important	Not important	All plausible residual confounders could reduce the demonstrated effect [b]	17/192 (8.9%)	53/286 (18.5%)	**RR 0.41** (0.24 a 0.71)	**109 - per 1.000** (from 141- to 54-)	⊕⊕⊕◯ MODERATE	IMPORTANT
**Risk of overweight and obesity. Observational (follow up: 9.5 years; assessed with:% overweight / obesity. BMIZ, ΔBMI, Skin fold.)**												
1 [3]	Observational studies	Very serious [d]	Not important	Not important	Not important	all plausible residual confounders would suggest a spurious effect, while no effect was observed	Responsive parenting at the age of less than 2 years was not associated with the risk of being overweight / obese.				⊕◯◯◯ VERY LOW	IMPORTANT

CI: Confidence interval; **RR:** Risk ratio

Explanations

a. Loss to follow-up limit (20%)

b. 1 non-randomized study ref # 2

c. Unique study

d. Low methodological quality, high loss at follow up, discordant data, inadequate analysis of confounding factorsReferences

1. Paul IM, Savage JS,Anzman-Frasca S,et al.. Effect of a Responsive Parenting Educational Intervention on Childhood Weight Outcomes at 3 Years of Age: The INSIGHT Randomized Clinical Trial. . JAMA. 2018;320(5):461-8; 2018.

2. Machuca H, Arevalo S,Hackley B,et al.. Well Baby Group Care: Evaluation of a Promising Intervention for Primary Obesity Prevention in Toddlers. . Child Obes. 2016 Jun;12(3):171-8. doi: 10.1089/chi.2015.0212. Epub 2016 Apr 1. ; 2016.

3. Agras WS, Hammer LD,McNicholas F,Kraemer HC. Risk factors for childhood overweight: a prospective study from birth to 9.5 years. The Journal of pediatrics. 2004, 145(1):20–25., . Risk factors for childhood overweight: a prospective study from birth to 9.5 years. . The Journal of pediatrics. 2004; 145(1):20–25.; 2004.

Table a4.26. nReCF. Risk of overweight and obesity

[nReCF] compared to [other models of CF] in [healthy child, in the period 6-24 months], can influence, in positive or negative way, [the development overweight and obesity in later age

Patient or population [healthy child in the period 6-24 months, in positive or negative way the development overweight and obesity in later age]

Setting: Outpatient

Intervention: [nReCF]

Comparator: [other models of CF]

Certainty assessment							Impact	Certainty	Importance
N of studies	Study design	Risk of distortion	Lack of reproducibility of results	Lack of generalizability	Imprecision	Further considerations			
**nReCF. Risk of overweight and obesity. Observational (follow up: interval 15 months to 20 months; assessed with:% overweight / obesity. BMIZ, ΔBMI, Skin fold.)**									
3 [1,2,3]	Observational studies	Serious [a,b,c]	Serious [c]	Not important	Not important	All plausible residual confounders could reduce the demonstrated effect	No significant association for all comparisons (for documented exposures ≥6 mo)	⨁◯◯◯ VERY LOW	IMPORTANT

CI: Confidence interval

Explanations

a. Risk of bias in assessing exposure in 2 out of 3 studies

b. high loss at follow-up in 2 out of 3 studies

c. Different parental styles evaluated, for some unique study: Pressure to eat Responsive Restriction Indulgent Laissez-faire. However, the results are generally consistent

References

1. Thompson AL, Adair LS,Bentley ME.. Pressuring and restrictive feeding styles influence infant feeding and size among a low-income African-American sample. . Obesity (Silver Spring). 2013;21(3):562-71; 2013.

2. Lumeng JC, Ozbeki TN,Appugliese DP,Kaciroti N,Corwyn RF,Bradley RH.. Observed assertive and intrusive maternal feeding behaviors increase child adiposity.. Am J Clin Nutr. 2012 Mar;95(3):640-7.; 2012.

3. Rifas-Shiman SL, Sherry B,Scanlon K,et al.. Does maternal feeding restriction lead to childhood obesity in a prospective cohort study? . Arch Dis Child. 2011;96(3):265-9; 2011.

A4. BLW/BLISS and choking risk.

- ***Do different CF models result in a different risk of choking?***
- ***Does the BLW/BLISS method during CF result in a different risk of choking?***

P. In a healthy baby aged 6-24 months
I. the Baby-Led Weaning (or BLISS method)
C. compared to other feeding models
O. result in a different risk of choking?

KEY WORDS

Population

([infant]/lim OR [child]/lim OR [preschool]/lim

Exposure Factors / Comparison

"self weaning"[All Fields]
"baby led weaning"[All Fields]
"Infant Nutritional Physiological Phenomena" [MeSH]
"Weaning"[MeSH])

Outcomes

"choking"[All Fields]
"gagging" [All Fields]
"Foreign Bodies"[Mesh]

Guidelines search

Filters applied: Guideline, Practice Guideline, in the last 5 years.

PUBMED https://www.ncbi.nlm.nih.gov/pubmed/

#1

("airway obstruction"[MeSH] OR "sudden airway obstruction"[All Fields] OR "acute airway obstruction"[All Fields] OR "choking"[All Fields] OR "gagging" OR "Foreign Bodies"[Mesh]) AND ("2014/09/16"[PDat] : "2021/03/15"[PDat])

EMBASE https://www.embase.com

#1

('airway obstruction'/exp OR 'choking' OR 'respiratory interruption' OR 'acute airway obstruction' OR 'sudden airway obstruction' OR 'foreign body'/exp) AND [2014-2021]/py AND 'practice

SOCIETY GUIDELINE LINKS:

National Guideline Clearinghouse (NGC) https://www.ahrq.gov/gam/index.html
Canadians Medical Association (CMA) https://www.cma.ca/clinicalresources/practiceguidelines
National Guideline Centre (NGC) - National Institute of Health and Care Excellence (NICE) https://www.rcplondon.ac.uk/about-us/what-we-do/national-guideline-centre-ngc
Scottish Intercollegiate Guidelines Network (SIGN) https://www.sign.ac.uk/our-guidelines.html
Australian Clinical Practice Guidelines (ACPG) https://www.clinicalguidelines.gov.au/
New Zealand Guidelines Group (NZGG) https://www.health.govt.nz/about-ministry/ministry-health-websites/new-zealand-guidelines-group
American Academy of Pediatrics (AAP) https://www.aap.org/en-us/Pages/Default.aspx
EPA/UNEPSA http://www.epa-unepsa.org/
Guidelines International Network https://g-i-n.net/
Irish National Clinical Guidelines https://www.gov.ie/en/collection/c9fa9a-national-clinical-guidelines/
European Society for Emergency Medicine. Paediatric Section https://eusem.org/sections-and-committees/sections/paediatric-section
Society for Academic Emergency Medicine. Pediatric Emergency Medicine https://community.saem.org/communities/community-home?CommunityKey=3dc973c2-35fd-42c2-9dcf-99e69a20d206
Società Italiana di Medicina di Emergenza ed Urgenza Pediatrica SIMEUP https://www.simeup.it/
Ministero della Salute Italia http://www.salute.gov.it/imgs/C_17_pubblicazioni_2618_allegato.pdf

Systematic Reviews search

Cochrane Databases

Cochrane Reviews matching choking OR gagging in Title Abstract Keyword - with Cochrane Library publication date Between Jan 2018 and Jan 2021, in Cochrane Reviews, Trials (Word variations have been searched)

PubMed

#1

("airway obstruction"[MeSH] OR "sudden airway obstruction"[All Fields] OR "acute airway obstruction"[All Fields] OR "choking"[All Fields] OR "gagging"[All Fields] OR "Foreign Bodies"[Mesh]) AND ((Meta-Analysis[ptyp] OR systematic[sb]) AND "2009/09/12"[PDat] : "2021/03/15"[PDat]) AND "infant"[MeSH Terms]

EMBASE

#1

('airway obstruction'/exp OR 'choking' OR 'respiratory interruption' OR 'acute airway obstruction' OR 'sudden airway obstruction' OR 'foreign body'/exp) AND [2009-2021]/py AND ([child]/lim OR [infant]/lim OR [preschool]/lim) AND ([systematic review]/lim OR [meta analysis]/lim)

Studies search (subsequent to SR of D'Auria et al. 2018)

PubMed

#1

("self-weaning"[All Fields] OR "self weaning"[All Fields] OR "baby led weaning"[All Fields] OR "baby-led weaning"[All Fields]) AND ("airway obstruction"[MeSH] OR "sudden airway obstruction"[All Fields] OR "acute airway obstruction"[All Fields] OR "choking"[All Fields] OR "gagging"[All Fields]) AND ((Clinical Trial[ptyp] OR Comparative Study[ptyp] OR Multicenter Study[ptyp] OR Observational Study[ptyp] OR Pragmatic Clinical Trial[ptyp] OR Randomized Controlled Trial[ptyp]) AND ("2018/03/01"[PDAT] : "2021/03/15"[PDAT]) AND "infant"[MeSH Terms])

EMBASE

#1

('baby led weaning'/exp OR 'baby led weaning' OR 'baby led' OR 'self weaning' OR autoweaning) AND ([infant]/lim OR [preschool]/lim) AND [2018-2021]/py AND ('airway obstruction'/exp OR 'choking' OR 'respiratory interruption' OR 'acute airway obstruction' OR 'sudden airway obstruction')

Cochrane Databases

Cochrane Reviews matching choking OR gagging in Title Abstract Keyword - with Cochrane Library publication date Between Jan 2018 and Jan 2021, in Cochrane Reviews, Trials (Word variations have been searched)

Figure a4.19. Guidelines search flow diagram.

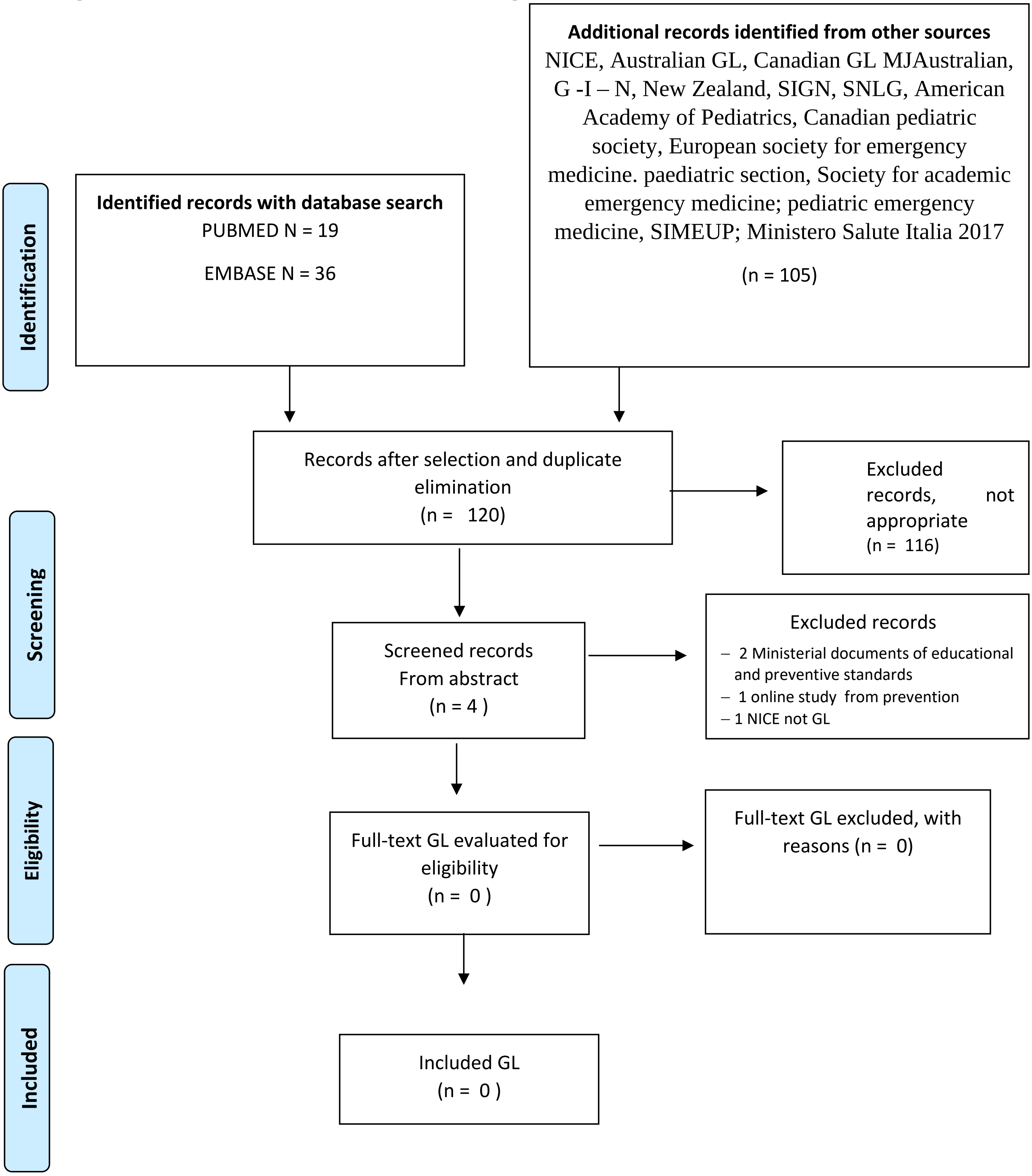

Figure a4.20. SRs search flow diagram.

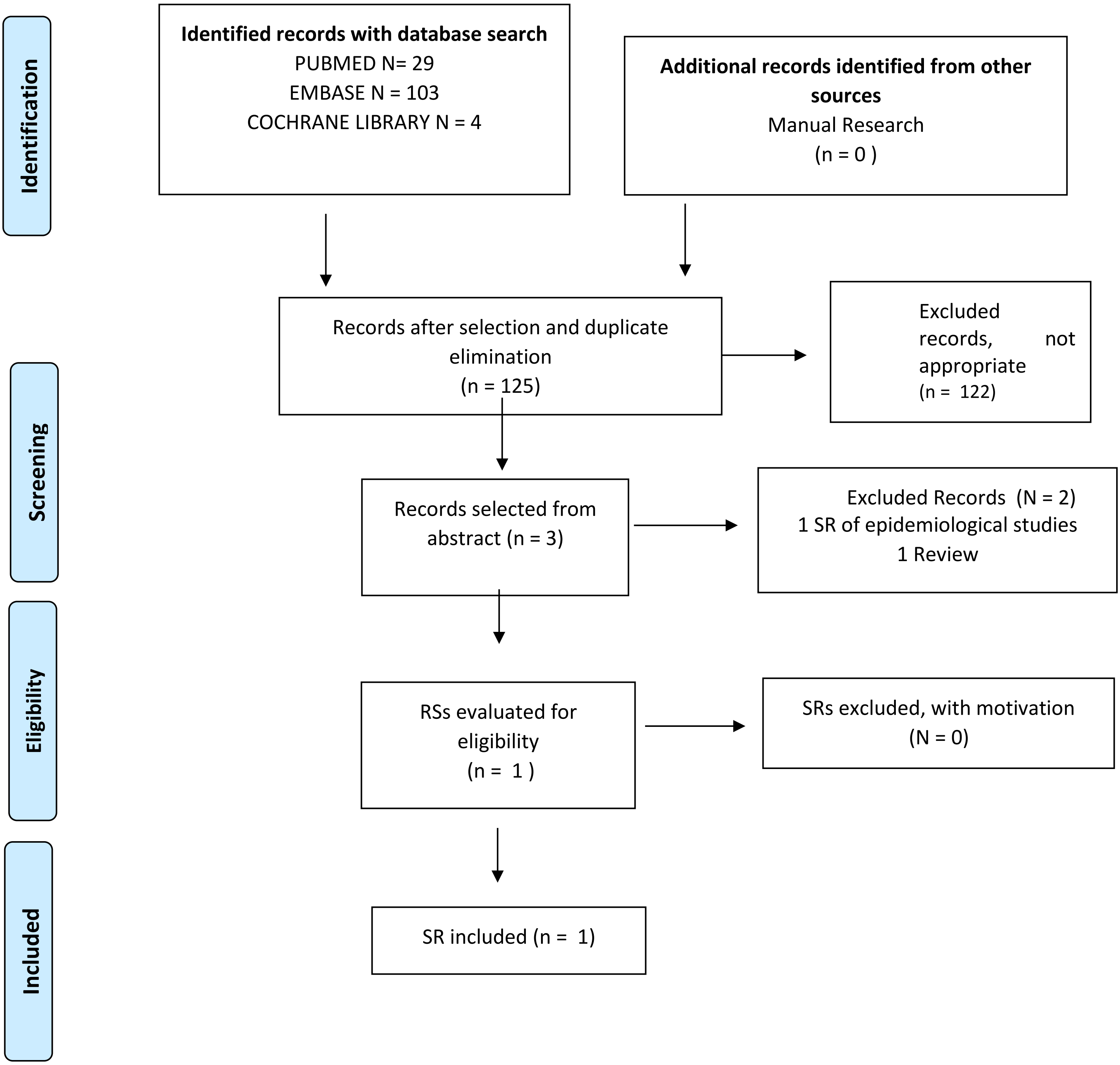

Figure a4.21. Studies flow diagram

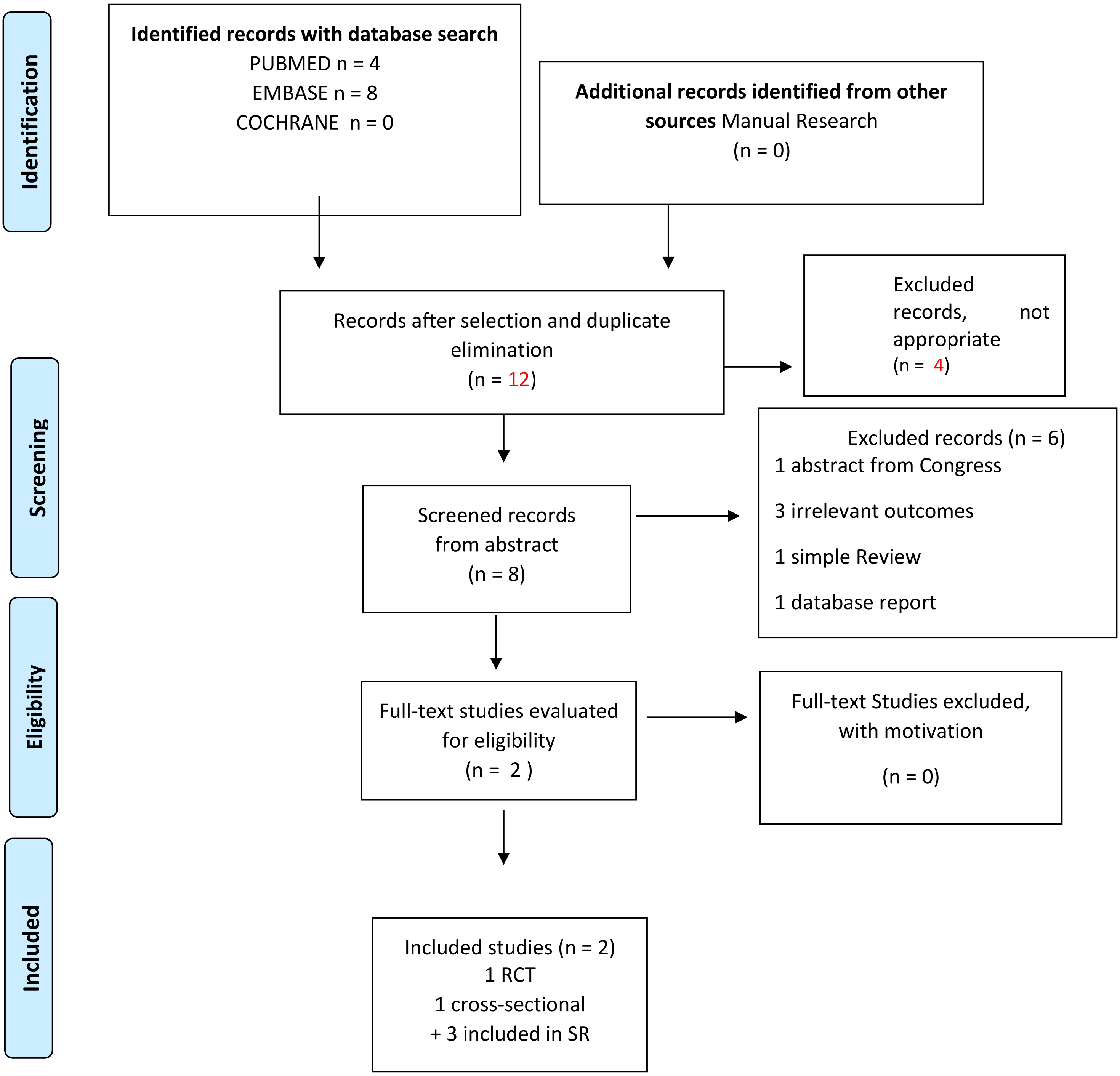

A4. ACRe and other models of CF and chocking.

P. In a healthy child aged 6-24 months
I. Different patterns of CF
C. compared with other feeding patterns
O. result in a different risk of choking?

KEY WORDS

Population

"Infant"[Mesh]
"Child"[Mesh]
"Child, Preschool"[Mesh]

Exposure Factors / Comparison

"responsive feeding"[All Fields]
"non-responsive feeding"[All Fields]
“responsiveness”[All Fields]
"Weaning"[All Fields] O
"Infant Nutritional Physiological Phenomena"[MeSH]
“complementary feeding”[All Fields]
"Feeding Behavior"[All Fields]
"Foreign Bodies"[Mesh]

Outcomes
"airway obstruction"[MeSH]
"sudden airway obstruction"[All Fields]
"acute airway obstruction"[All Fields]
"choking"[All Fields]
"gagging"[MeSH Terms]

Guidelines search

PUBMED https://www.ncbi.nlm.nih.gov/pubmed/

("airway obstruction"[MeSH] OR "sudden airway obstruction"[All Fields] OR "acute airway obstruction"[All Fields] OR "choking"[All Fields] OR “gagging” OR "Foreign Bodies"[Mesh]) AND ("2014/09/16"[PDat] : "2021/03/15"[PDat])

EMBASE https://www.embase.com

#1

(('complementary feeding'/exp OR 'responsiveness' OR 'responsive feeding' OR 'non responsive feeding') AND ('airway obstruction'/exp OR 'choking' OR 'respiratory interruption' OR 'acute

airway obstruction' OR 'sudden airway obstruction')) AND ([infant]/lim OR [preschool]/lim) AND [2014-2021]/py

SOCIETY GUIDELINE LINKS:

National Guideline Clearinghouse (NGC) https://www.ahrq.gov/gam/index.html
Canadians Medical Association (CMA) https://www.cma.ca/clinicalresources/practiceguidelines
National Guideline Centre (NGC) - National Institute of Health and Care Excellence (NICE) https://www.rcplondon.ac.uk/about-us/what-we-do/national-guideline-centre-ngc
Scottish Intercollegiate Guidelines Network (SIGN) https://www.sign.ac.uk/our-guidelines.html
Australian Clinical Practice Guidelines (ACPG) https://www.clinicalguidelines.gov.au/
New Zealand Guidelines Group (NZGG) https://www.health.govt.nz/about-ministry/ministry-health-websites/new-zealand-guidelines-group
American Academy of Pediatrics (AAP) https://www.aap.org/en-us/Pages/Default.aspx
EPA/UNEPSA http://www.epa-unepsa.org/
Guidelines International Network https://g-i-n.net/
Irish National Clinical Guidelines https://www.gov.ie/en/collection/c9fa9a-national-clinical-guidelines/
European Society for Emergency Medicine. Paediatric Section https://eusem.org/sections-and-committees/sections/paediatric-section
Society for Academic Emergency Medicine. Pediatric Emergency Medicine https://community.saem.org/communities/community-home?CommunityKey=3dc973c2-35fd-42c2-9dcf-99e69a20d206
Società Italiana di Medicina di Emergenza ed Urgenza Pediatrica SIMEUP https://www.simeup.it/
Ministero della Salute Italia http://www.salute.gov.it/imgs/C_17_pubblicazioni_2618_allegato.pdf

Systematic Review search

PUBMED

#1

(("responsive feeding"[All Fields] OR "non-responsive feeding"[All Fields] OR “responsiveness”[All Fields] OR "Weaning"[All Fields] OR "Infant Nutritional Physiological Phenomena"[MeSH] OR “complementary feeding”[All Fields] OR "Feeding Behavior"[All Fields]) AND ("airway obstruction"[MeSH] OR "sudden airway obstruction"[All Fields] OR "acute airway obstruction"[All Fields] OR "choking"[All Fields] OR “gagging”)) AND ("2014/09/16"[PDat] : "2021/03/15"[PDat]) AND (systematic[sb] OR Meta-Analysis[ptyp])

#2

((((((((((((incentiv* OR indulgen*[tiab] OR authorita*[tiab] OR reward* OR control* OR pressur* OR restrict* OR monitor* OR respons* OR sooth*[tiab] OR encourag* OR discourage* OR uninvolv* OR disengage* OR parenting style* OR laissez-faire OR laissez faire* OR non-respons* OR nonrespons* OR force*) AND (feeding* OR fed[tiab] OR eat[tiab] OR eating))) OR ("Feeding Methods"[Mesh:noexp] OR "Feeding Behavior"[Mesh:NoExp] OR

((satiety OR hunger OR hungry OR satiat*) AND (cue OR cues)) OR feeding method* OR feeding practice* OR feeding pattern* OR feeding frequenc* OR infant feed* OR feeding behavior*[tiab] OR feeding style* OR feeding strategy*))))

AND ("2014/09/16"[PDat] : "2021/03/15"[PDat]))))

AND ((systematic[sb] OR Meta-Analysis[ptyp]

AND ((((infant* OR baby OR babies OR toddler* OR newborn*[tiab] OR "Child, Preschool"[Mesh] OR preschool*[tiab] OR pre-school*[tiab] OR “early childhood”[tiab] OR early year*[tiab] OR pre-k[tiab] OR pre-primary[tiab] OR under five*[ti] OR young child*[ti] OR prekindergarten[tiab] OR pre-kindergarten[tiab] OR weanling* OR “first two years” OR “first 2 years”)))))

AND (("airway obstruction"[MeSH] OR "sudden airway obstruction"[All Fields] OR "acute airway obstruction"[All Fields] OR "choking"[All Fields]))

EMBASE

#1

(('complementary feeding'/exp OR 'responsiveness' OR 'responsive feeding' OR 'non responsive feeding') AND ('airway obstruction'/exp OR 'choking' OR 'respiratory interruption' OR 'acute airway obstruction' OR 'sudden airway obstruction')) AND ([infant]/lim OR [preschool]/lim) AND [2014-2021]/py

COCHRANE LIBRARY https://www.cochranelibrary.com

Cochrane Reviews matching choking OR gagging in Title Abstract Keyword - with Cochrane Library publication date Between Jan 2011 and Jan 2021, in Cochrane Reviews, Trials (Word variations have been searched

Studies search

PUBMED

#1

(("responsive feeding"[All Fields] OR "non-responsive feeding"[All Fields] OR “responsiveness”[All Fields] OR "Weaning"[All Fields] OR "Infant Nutritional Physiological Phenomena"[MeSH] OR “complementary feeding”[All Fields] OR "Feeding Behavior"[All Fields]) AND ("airway obstruction"[MeSH] OR "sudden airway obstruction"[All Fields] OR "acute airway obstruction"[All Fields] OR "choking"[All Fields] OR “gagging”) AND (Randomized Controlled Trial[ptyp] OR Pragmatic Clinical Trial[ptyp] OR Observational Study[ptyp] OR Multicenter Study[ptyp] OR Comparative

Study[ptyp] OR Clinical Trial[ptyp] OR Controlled Clinical Trial[ptyp])) AND ("1979/01/01"[PDat] : "2021/03/15"[PDat])

#2

((((((((((((((incentiv* OR indulgen*[tiab] OR authorita*[tiab] OR reward* OR control* OR pressur* OR restrict* OR monitor* OR respons* OR sooth*[tiab] OR encourag* OR discourage* OR uninvolv* OR disengage* OR parenting style* OR laissez-faire OR laissez faire* OR non-respons* OR nonrespons* OR force*) AND (feeding* OR fed[tiab] OR eat[tiab] OR eating))) OR ("Feeding Methods"[Mesh:noexp] OR "Feeding Behavior"[Mesh:NoExp] OR ((satiety OR hunger OR hungry OR satiat*) AND (cue OR cues)) OR feeding method* OR feeding practice* OR feeding pattern* OR feeding frequenc* OR infant feed* OR feeding behavior*[tiab] OR feeding style* OR feeding strategy*))))
AND ("1979/01/01"[PDat] : "2021/03/15"[PDat])
AND Randomized Controlled Trial[ptyp] OR Pragmatic Clinical Trial[ptyp] OR Observational Study[ptyp] OR Multicenter Study[ptyp] OR Comparative Study[ptyp] OR Clinical Trial[ptyp] OR Controlled Clinical Trial[ptyp]))))
AND ((((infant* OR baby OR babies OR toddler* OR newborn*[tiab] OR "Child, Preschool"[Mesh] OR preschool*[tiab] OR pre-school*[tiab] OR “early childhood”[tiab] OR early year*[tiab] OR pre-k[tiab] OR pre-primary[tiab] OR under five*[ti] OR young child*[ti] OR prekindergarten[tiab] OR pre-kindergarten[tiab] OR weanling* OR “first two years” OR “first 2 years”)))))
AND (("airway obstruction"[MeSH] OR "sudden airway obstruction"[All Fields] OR "acute airway obstruction"[All Fields] OR "choking"[All Fields]))

#3

(("airway obstruction"[MeSH] OR "sudden airway obstruction"[All Fields] OR "acute airway obstruction"[All Fields] OR "choking"[All Fields] OR "gagging"[All Fields]) AND (Case Reports[ptyp] OR Clinical Trial[ptyp] OR Comparative Study[ptyp] OR Guideline[ptyp] OR Meta-Analysis[ptyp] OR Multicenter Study[ptyp] OR Observational Study[ptyp] OR Pragmatic Clinical Trial[ptyp] OR Randomized Controlled Trial[ptyp]))
AND ("1979/01/01"[PDat] : "2021/03/15"[PDat])
AND ("infant"[MeSH Terms])

EMBASE

#1

(('complementary feeding'/exp OR 'responsiveness' OR 'responsive feeding' OR 'non responsive feeding') **AND** ('airway obstruction'/exp OR 'choking' OR 'respiratory interruption' OR 'acute airway obstruction' OR 'sudden airway obstruction')) **AND** ([infant]/lim OR [preschool]/lim) **AND** [1979-2021]/py

COCHRANE LIBRARY

Trials matching choking OR gagging in Title Abstract Keyword - with Cochrane Library publication date Between Between Jan 2011 and Jan 2021, in Trials

Figure a4.22. Guidelines search flow diagram - General Choking.

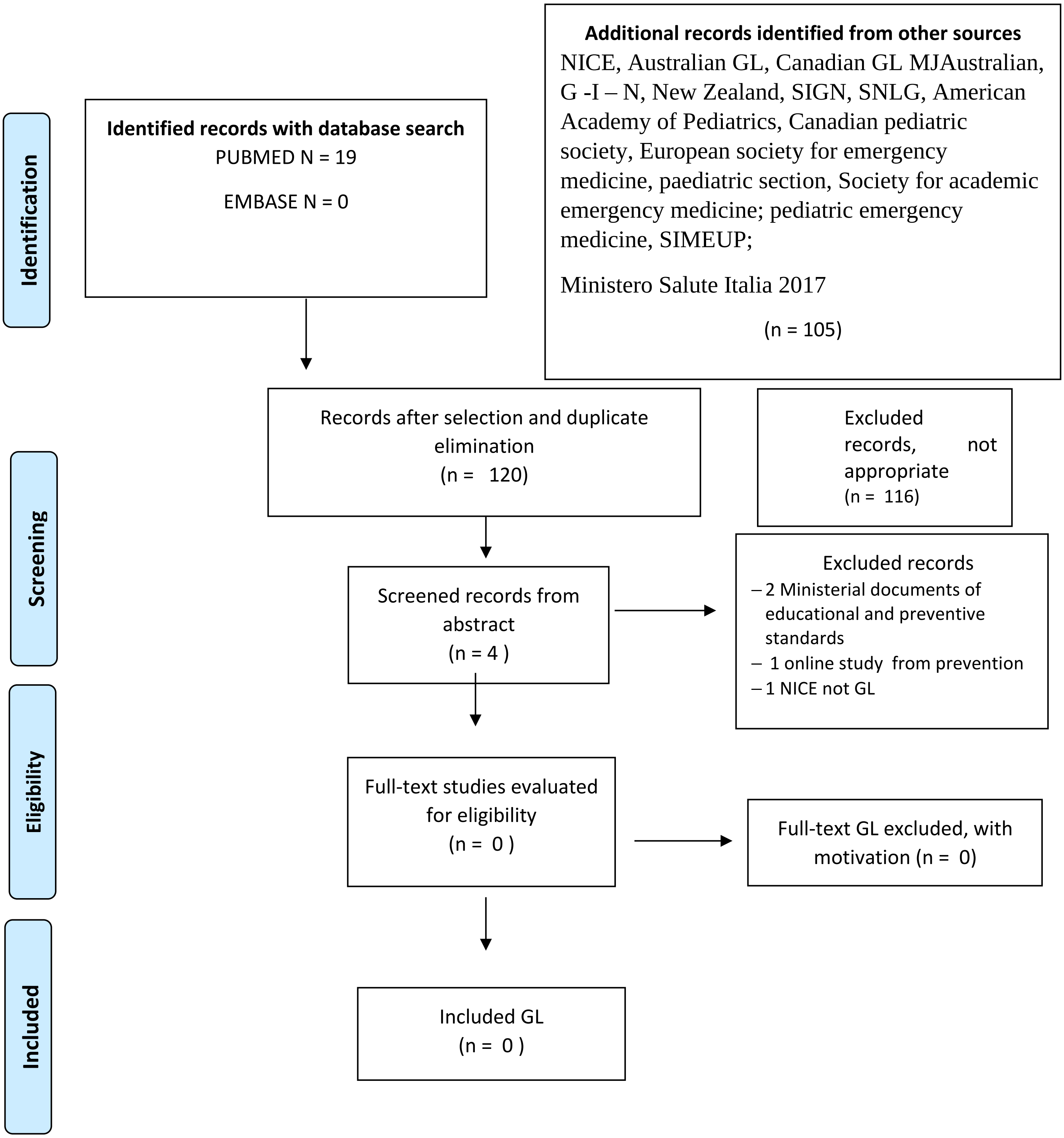

Figure a4.23. SRs search flow diagram. - General Choking.

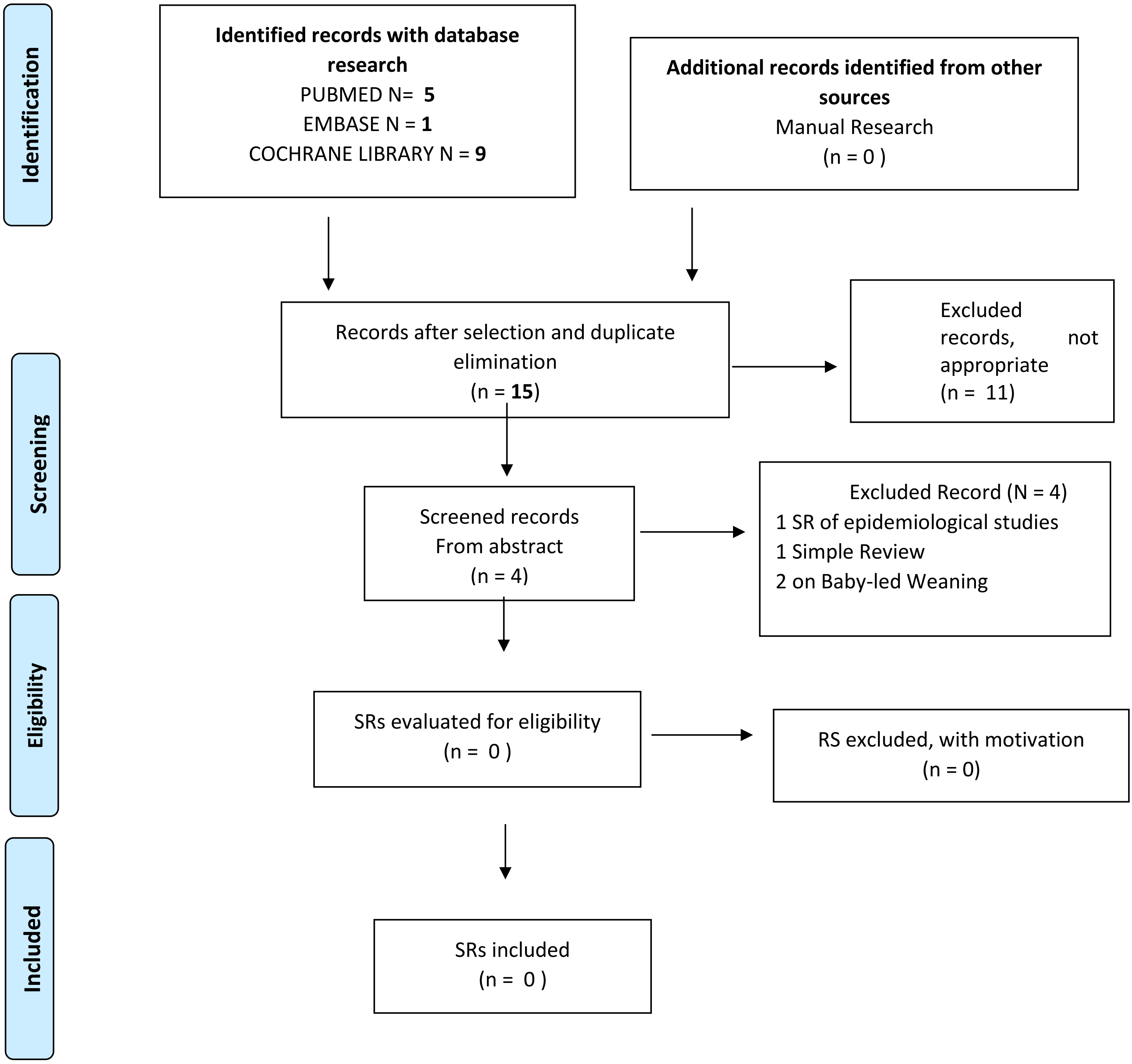

Figure a4.24. Studies flow diagram – General Choking.

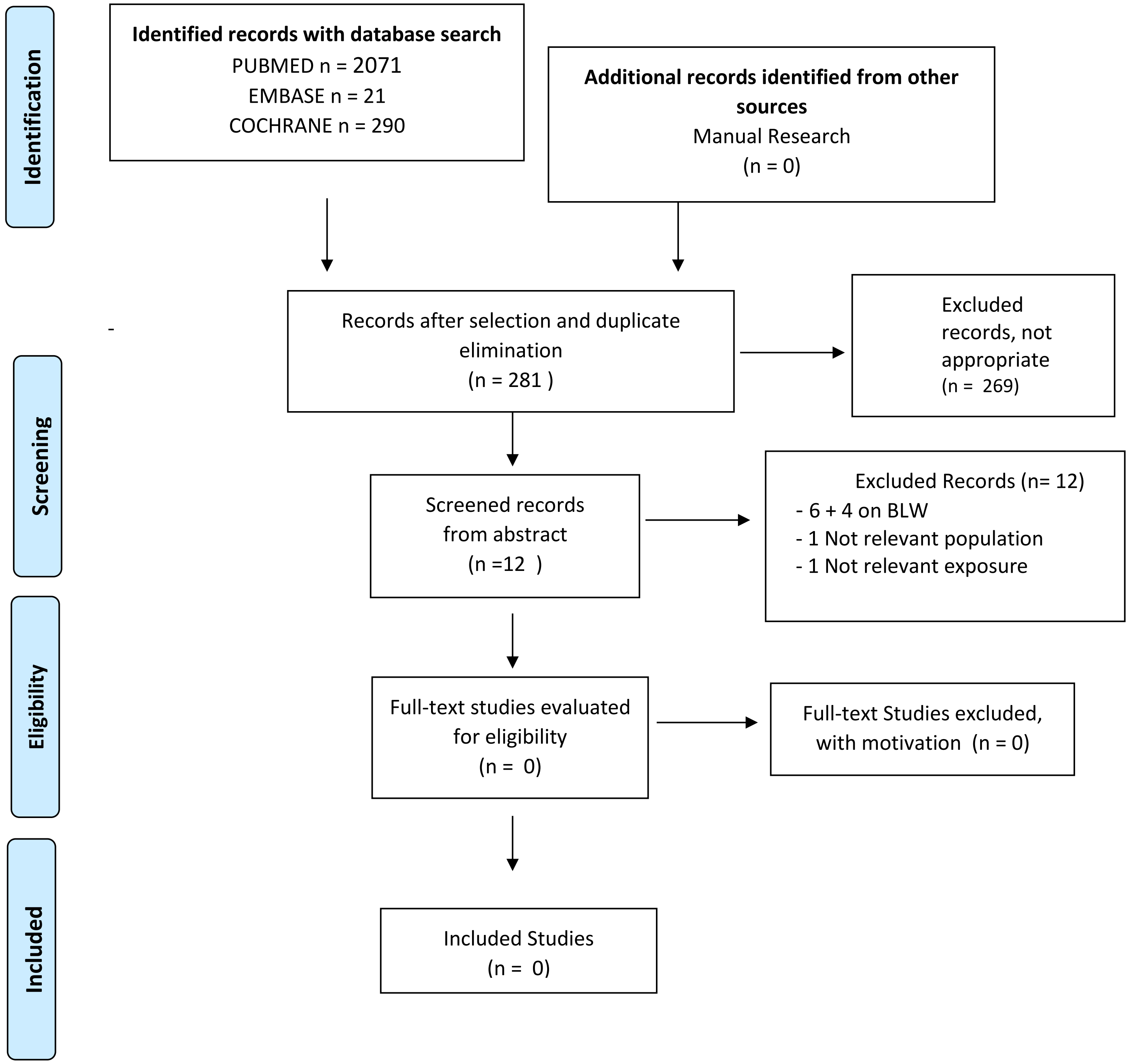

A4. METHODOLOGICAL ASSESSMENT

A4. BLW/BLISS and choking risk.

Table a4.27. Appraisal of the Systematic Reviews.

AMSTAR 2	D'Auria et al. 2018 [1]
**1. Did the research questions and inclusion criteria for the review include the components of PICO?**<br>**(Yes/No)**	Yes
**2. Did the report of the review contain an explicit statement that the review methods were established before the conduct of the review and did the report justify any significant deviations from the protocol?**<br>**(Yes/Partial Yes/No)**	Partial Yes
**3. Did the review authors explain their selection of the study designs for inclusion in the review?**<br>**(Yes/No)**	Yes
**4. Did the review authors use a comprehensive literature search strategy?**<br>**(Yes/Partial Yes/No)**	Partial Yes
**5. Did the review authors perform study selection in duplicate?**<br>**(Yes/No)**	Yes
**6. Did the review authors perform data extraction in duplicate?**<br>**(Yes/No)**	Yes
**7. Did the review authors provide a list of excluded studies and justify the exclusions?**<br>**(Yes/Partial Yes/No)**	Yes
**8. Did the review authors describe the included studies in adequate detail?**<br>**(Yes/Partial Yes/No)**	Yes
**9. Did the review authors use a satisfactory technique for assessing the risk of bias (RoB) in individual studies that were included in the review?**<br>**(Yes/Partial Yes/No/Includes only NRSI-RCT)**	Yes<br>Yes
**10. Did the review authors report on the sources of funding for the studies included in the review?**<br>**(Yes/No)**	NO
**11. If meta-analysis was performed did the review authors use appropriate methods for statistical combination of results?**<br>**(Yes / No / No meta-analysis conducted)**	No metanalysis performed.

**12. If meta-analysis was performed, did the review authors assess the potential impact of RoB in individual studies on the results of the meta-analysis or other evidence synthesis?**<br><br>**(Yes / No / No meta-analysis conducted)**	No metanalysis performed.
**13. Did the review authors account for RoB in individual studies when interpreting/ discussing the results of the review?**<br><br>**(Yes/No)**	Yes
**14. Did the review authors provide a satisfactory explanation for, and discussion of, any heterogeneity observed in the results of the review?**<br><br>**(Yes/No)**	Yes
**15. If they performed quantitative synthesis did the review authors carry out an adequate investigation of publication bias (small study bias) and discuss its likely impact on the results of the review?**<br><br>**(Yes / No / No meta-analysis conducted)**	Nessuna Metanalisi
**16. Did the review authors report any potential sources of conflict of interest, including any funding they received for conducting the review?**<br><br>**(Yes/No)**	Yes
**OVERALL EVALUATION**	**MODERATE QUALITY**

Figure a4.25. Risk of bias summary: review authors' judgements about each risk of bias item for each included study. [7,41]

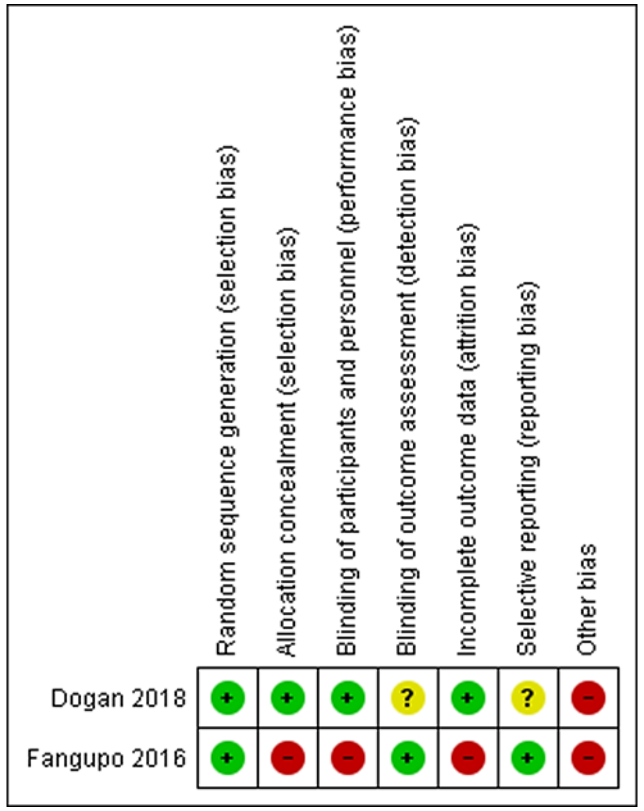

Figure a4.26. Risk of bias graph: review authors' judgements about each risk of bias item presented as percentages across all included studies.

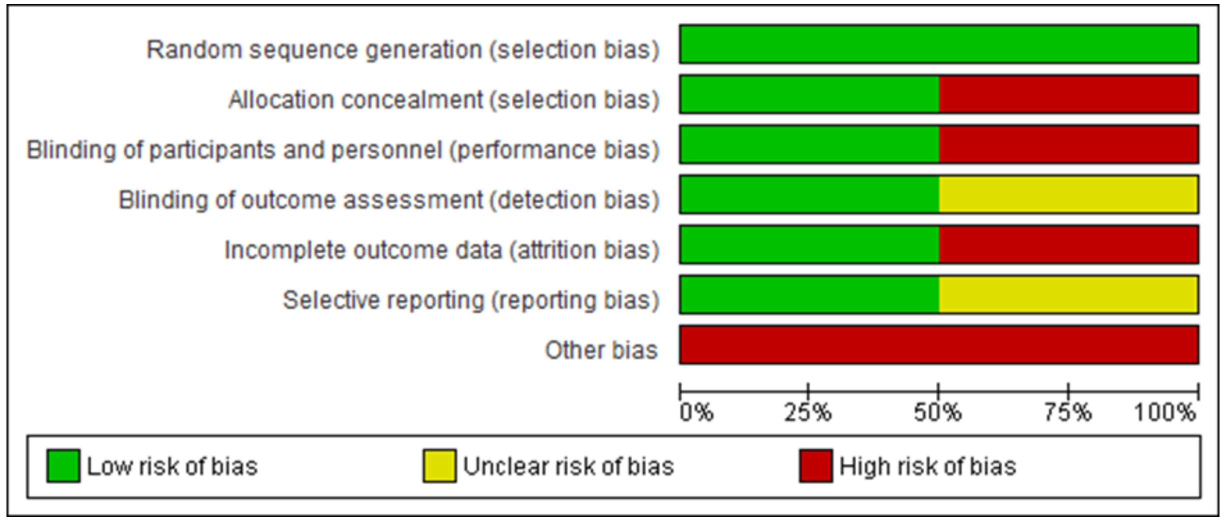

-

Table a4.28. Appraisal of the Studies

	Newcastle Quality Assessment Scale STUDI CROSS-SECTIONAL							
	**Selection**				**Comparability**	**Outcome**		
**Study**	**Representativeness of the sample**	**Sample size:**	**Non-respondents**	**Ascertainment of the exposure (max 2)**	**Comparability between groups, confounders are controlled (Maximum 2 stars)**	**Outcome evaluation (max 2)**	**Statistical test**	**Total**
Cameron et al. 2013 [49]	1b	b	1a	1b	1a<br>1b	c	1a	**6**<br>**Good quality**
Brown et al. 2017 [50]	c	b	c	1b	1a<br>1b	c	1a	**4**<br>**Good quality**
Fu et al. 2018 [51]	c	1a	c	1b	c	c	1a	**3**<br>**Good quality**

Table a4.29. Excluded studies with motivation

EXCLUDED STUDIES	Reason for exclusion
Özyüksel et al. 2019 [52]	High risk of exposure ascertainment bias (assessed self-feeding, but not ascertained BLW/BLISS mode).

A4. RECOMMENDATIONS OF GLs, RESULTS IN SRs AND STUDIES

A4. BLW/BLISS and choking risk.

- ***Does the BLW/BLISS method during CF result in a different risk of choking?***	**P.** In a healthy baby aged 6-24 months<br>**I.** the Baby-Led Weaning (or BLISS method)<br>**C.** compared to other feeding models<br>**O.** result in a different risk of choking?

Table a4.30. Included SRs: Characteristics, Results, and Conclusions.

Systematic Review	Population and purpose of the SR	Results	Conclusions
D'Auria et al. 2018 (SRs of RCT and observational studies) [1]	CF-age children fed using the BLW method compared with children fed using traditional methods.<br>Long-term health outcomes: risk of choking.<br>(auxological, metabolic parameters, relational indicators).	No significant differences in the risk of choking, in the 2 observational studies .<br><br>No significant difference in the randomized trial that used the BLISS method.	The methodological quality of the studies is poor and no firm conclusions can be drawn, despite the concordance of results.

Table a4.31. Included studies: Characteristics and Results.

Study (First Author, year, Country/Setting)	Study design	Population (sample size, baseline characteristics)	Intervention/exposure	Primary Outcome	Secondary Outcomes	Follow-up	Results
Cameron et al. 2013 [49]	Cross-sectional study.<br><br>Data from self-completed questionnaire.	N = 199 mothers of children aged 6-7 mo.	BLW vs traditional CF	Dietary behaviors and preferences<br><br>Frequency of choking and gagging episodes	/	/	No significant difference in choking and "gagging" episodes between groups.
Brown et al. 2017 [50]	Cross-sectional study.<br><br>Data from self-completed questionnaire.	N = 1151 mothers of children aged 4 -12 mo.	BLW vs traditional CF	Frequency of choking and gagging episodes.	/	/	No significant difference in choking and "gagging" episodes between groups.
Fu et al. 2018 [51]	Cross-sectional study.<br><br>Data from self-completed questionnaire.	From 6 to 36 mo.	BLW vs traditional CF	Frequency of "fussy eating," weight, choking risk.	/	/	Frequency of choking ranging from 0% to 2% across groups, without significance.
Fangupo et al. 2016 (BLISS method) [41]	Open RCT	N = 206 healthy women ((105 BLISS, 101 traditional CF)	BLISS vs traditional CF	BMI z-score at 12 and 24 mo	Frequency of choking episodes.<br>Caloric and micronutrient intakes.	12 mo of follow-up	No significant difference in the no. of choking episodes<br>-35% of children at least one episode between 6 and 8 mo of age
Dogan et al. 2018 (BLISS method) [7]	Open RCT	302 children aged 5-6 mo (156 BLISS, 146 traditional CF)	BLISS vs traditional CF	Weight, length and CC.	Choking, haematological parameters and eating behaviours, at 12 mo	12 mo. of life (N = 280)	No significant difference in the no. of choking episodes.

A4 . EVIDENCE PROFILE GRADE

A4. BLW/BLISS and choking risk.

[BLW/BLISS] compared to [traditional CF] in [baby aged 6-24 months result in a different risk of choking]

Patient or population: [baby aged 6-24 months]

Setting: Outpatient

Intervention: [BLW/BLISS]

Comparator: [traditional CF]

Table a4.32. BLW/BLISS and choking risk. RCT.

Certainty assessment							Impact				Certainty of evidence	Importance
№ of studies	Study Design	Distortion risk	Lack of reproducibility of results	Lack of generalisability	Inaccuracy	Further considerations						
**BLW/BLISS. Choking risk. RCT (Follow up: interval 4 months to 12 months; assessed with: incidence of choking episodes).**												
2 [1,2]	Randomized Studies	Serious [a,b]	Not relevant	Not relevant	Serious [c]	All plausible residual confounders could reduce the demonstrated effect	71/217 (32.7%)	70/207 (33.8%)	**RR 0.93** (0.85 to 1.03)	**24 minus per 1,000** (from 51 minus to 10 plus)	⊕⊕⊕◯ MODERATE	CRITICAL

Table a4.32. BLW/BLISS. Choking risk. Observational studies.

3 [3,4,5]	Observational studies	Very serious [d]	Serious [d]	Not relevant	Not relevant	All plausible residual confounders could reduce the demonstrated effect	Very low quality of evidence, however consistent results. even with RCTs. No statistically significant difference.	⊕◯◯◯ VERY LOW	CRITICAL

CI: Confidence interval; **RR:** Risk ratio

Explanations

a. Alto rischio di bias per perdite al follow up: Alto rischio (perdita 14% e 21,5% a 12 e 24 mesi; ITT non eseguito) Alto (perdita del 7,3%; analisi non ITT).

b. In Dogan 2018, choking is a secondary outcome.

c. Number of events per sample very different in the 2 studies.

d. Self-reported cases. Sample numbering not calculated, Lost to follow-up not described.

References

1. Dogan E, Yilmaz G,Caylan N,et al.. Baby-led complementary feeding: Randomized controlled study. . Pediatrics International 2018, 60: 1073-1080.; 2018.

2. Fangupo LJ, Heath AM,Williams SM,et al.. Baby-Led Approach to Eating Solids and Risk of Choking. . Pediatrics. 2016 Oct;138(4).; 2016.

3. 2017., Brown,A.,No,difference,in,self-reported,frequency,of,choking,between,infants,introduced,to,solid,foods,using,a,baby-led,weaning,or,traditional,spoon-feeding,approach.,J,Hum,Nutr,Diet.. .

4. Cameron SL, Taylor RW,Heath AL. Parent-led or baby-led? Association between complementary feeding practices and health-related behaviours in a survey of New Zealand families. BMJ Open. 2013, 3:e003946, . .

5. Fu X., Conlon C.A.,Haszard J.J.,Beck K.L.,von Hurst P.R.,Taylor R.W. & Heath A.-L.M.,Food fussiness and early feeding characteristics of infants following Baby-Led Weaning and traditional spoon-feeding in New Zealand: An internet survey,Appetite (2018).. .

A4. Responsive and non-responsive complementary feeding and DM2 development.

- ***Is ReCF able to influence the development of type 2 diabetes mellitus at later ages?***
- ***Is traditional AC able to influence the development of type 2 diabetes mellitus at later ages?***

PICOs

P In a healthy infant

I responsive complementary feeding during the period of Complementary Feeding

C compared to traditional complementary feeding during the Complementary Feeding period

O does it result in a different risk of developing type 2 diabetes mellitus at later ages?

KEY WORDS

Population

[infant]/lim
[child]/lim
[preschool]/lim
[adolescent]/lim

Exposure Factors / Comparison

"responsive feeding"[All Fields]
"non-responsive feeding"[All Fields]
"responsiveness"[All Fields]
"Weaning"[All Fields]
"Infant Nutritional Physiological Phenomena"[MeSH]
"complementary feeding"[All Fields]
"Feeding Behavior"[All Fields])

Outcomes

"Diabetes Mellitus"[Mesh]
"Glucose Intolerance"[Mesh]
"Hyperglycemia"[Mesh]

Guidelines search

SOCIETY GUIDELINE LINKS:

National Guideline Clearinghouse (NGC) https://www.ahrq.gov/gam/index.html

Canadians Medical Association (CMA) https://www.cma.ca/clinicalresources/practiceguidelines

National Guideline Centre (NGC) - National Institute of Health and Care Excellence (NICE) https://www.rcplondon.ac.uk/about-us/what-we-do/national-guideline-centre-ngc

Scottish Intercollegiate Guidelines Network (SIGN) https://www.sign.ac.uk/our-guidelines.html

Australian Clinical Practice Guidelines (ACPG) https://www.clinicalguidelines.gov.au/

New Zealand Guidelines Group (NZGG) https://www.health.govt.nz/about-ministry/ministry-health-websites/new-zealand-guidelines-group

American Academy of Pediatrics (AAP) https://www.aap.org/en-us/Pages/Default.aspx

European Paediatric Association/Union of National European Paediatric Societies and Associations EPA/UNEPSA http://www.epa-unepsa.org/

Guidelines International Network https://g-i-n.net/

Società Italiana di Pediatria (SIP) http://www-sip.it/

Società Italiana di Pediatria Preventiva e Sociale (SIPPS) https://www.sipps.it/

Società Italiana di Endocrinologia e Diabetologia Pediatrica (SIEDP) http://www.siedp.it/pagina/84/linee+guida%2C+raccomandazioni+e+consensus

American Diabetes Association https://www.diabetes.org/
Canadian Diabetes Association - https://rarediseases.org/organizations/canadian-diabetes-association/ - https://guidelines.diabetes.ca/
Association of Children’s Diabetes Clinicians http://www.a-c-d-c.org/
International Society for Pediatric and Adolescent Diabetes (ISPAD) https://www.ispad.org/

PUBMED

#1

(("Diabetes Mellitus"[Mesh] OR "Diabetes Mellitus/prevention and control"[Mesh] OR "Glucose Intolerance"[Mesh]) OR "Hyperglycemia"[Mesh]) AND ((Practice Guideline[ptyp] OR Guideline[ptyp]) AND "2014/09/15"[PDat] : "2021/03/15"[PDat] AND ("infant"[MeSH Terms] OR "child, preschool"[MeSH Terms] OR [adolescent]/lim))

#2

(("Diabetes Mellitus"[Mesh] OR "Diabetes Mellitus/prevention and control"[Mesh] OR "Glucose Intolerance"[Mesh]) OR "Hyperglycemia"[Mesh]) AND (("responsive feeding"[All Fields] OR "non-responsive feeding"[All Fields] OR "responsiveness"[All Fields] OR "Weaning"[All Fields] OR "Infant Nutritional Physiological Phenomena"[MeSH] OR "complementary feeding"[All Fields] OR "Feeding Behavior"[All Fields]) OR ("self-weaning"[All Fields] OR "self weaning"[All Fields] OR "baby led weaning"[All Fields] OR "baby-led weaning"[All Fields])) AND ((Practice Guideline[ptyp] OR Guideline[ptyp]) AND "2014/09/15"[PDat] : "2021/03/15"[PDat])

EMBASE

#1

('diabetes mellitus'/exp OR 'hyperglycemia'/exp) AND ('complementary feeding'/exp OR 'weaning'/exp OR ‘baby led weaning’/exp OR ‘baby led weaning’ OR ‘baby led’ OR ‘weaning’/exp OR weaning OR ‘self weaning’ OR autoweaning’ OR “responsiveness” OR “responsive feeding” OR “non responsive feeding”) AND [2014-2021]/py AND ([adolescent]/lim OR [child]/lim OR [preschool]/lim OR [school]/lim OR [young adult]/lim) AND 'practice guideline'/de

Systematic Reviews search

COCHRANE LIBRARY

“Diabetes mellitus” in Title Abstract Keyword

Custom date range Topics: 15.09.2014-15.03.2021

EMBASE

#1

('diabetes mellitus'/exp OR 'hyperglycemia'/exp) AND ('complementary feeding'/exp OR 'weaning'/exp OR “responsiveness” OR “responsive feeding” OR “non responsive feeding”) AND [2009-2021]/py AND ([adolescent]/lim OR [child]/lim OR [preschool]/lim OR [school]/lim OR [young adult]/lim) AND ('meta analysis'/de OR 'systematic review'/de)

PUBMED

#1

(("Diabetes Mellitus"[Mesh] OR "Diabetes Mellitus/prevention and control"[Mesh] OR "Glucose Intolerance"[Mesh]) OR "Hyperglycemia"[Mesh]) AND (("responsive feeding"[All Fields] OR "non-responsive feeding"[All Fields] OR "responsiveness"[All Fields] OR "Weaning"[All Fields] OR "Infant Nutritional Physiological Phenomena"[MeSH] OR "complementary feeding"[All Fields] OR "Feeding Behavior"[All Fields]) AND ((Meta-Analysis[ptyp] OR systematic[sb]) AND "2009/09/15"[PDat] : 2021/03/15 [PDat] AND ("infant"[MeSH Terms] OR "child"[MeSH Terms] OR "adolescent"[MeSH Terms]))

Studies search

PUBMED

#1

(("Diabetes Mellitus"[Mesh] OR "Diabetes Mellitus/prevention and control"[Mesh] OR "Glucose Intolerance"[Mesh]) OR "Hyperglycemia"[Mesh]) AND (("responsive feeding"[All Fields] OR "non-responsive feeding"[All Fields] OR "responsiveness"[All Fields] OR "Weaning"[All Fields] OR "Infant Nutritional Physiological Phenomena"[MeSH] OR "complementary feeding"[All Fields] OR "Feeding Behavior"[All Fields]) AND ((Clinical Trial[ptyp] OR Comparative Study[ptyp] OR Controlled Clinical Trial[ptyp] OR Multicenter Study[ptyp] OR Observational Study[ptyp] OR Pragmatic Clinical Trial[ptyp] OR Randomized Controlled Trial[ptyp]) AND ("infant"[MeSH Terms] OR "child"[MeSH Terms] OR "adolescent"[MeSH Terms]))

EMBASE

#1

('diabetes mellitus'/exp OR 'hyperglycemia'/exp) AND ('complementary feeding'/exp OR 'weaning'/exp OR “responsiveness” OR “responsive feeding” OR “non responsive feeding”) AND ('clinical trial'/de OR 'cohort analysis'/de OR 'comparative study'/de OR 'controlled clinical trial'/de OR 'controlled study'/de OR 'cross-sectional study'/de OR 'multicenter study'/de OR 'observational study'/de OR 'prospective study'/de OR 'randomized controlled trial'/de OR 'retrospective study'/de) AND ([adolescent]/lim OR [child]/lim OR [infant]/lim OR [preschool]/lim OR [school]/lim OR [young adult]/lim)

Figure a4.27. Guidelines search flow diagram. Responsive and non-responsive complementary feeding and DM2 development.

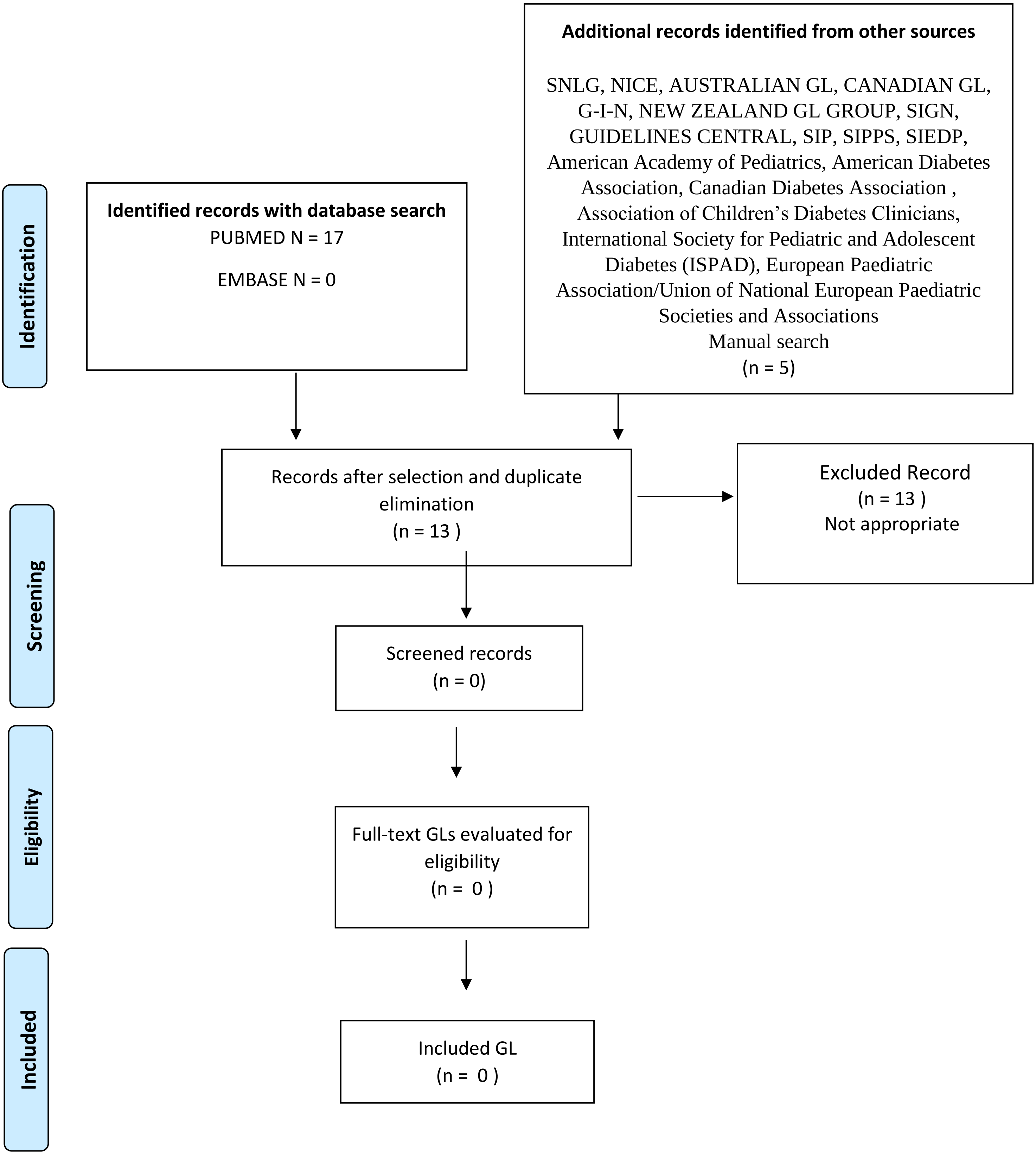

Figure a4.28. SRs search flow diagram. Responsive and non-responsive complementary feeding and DM2 development.

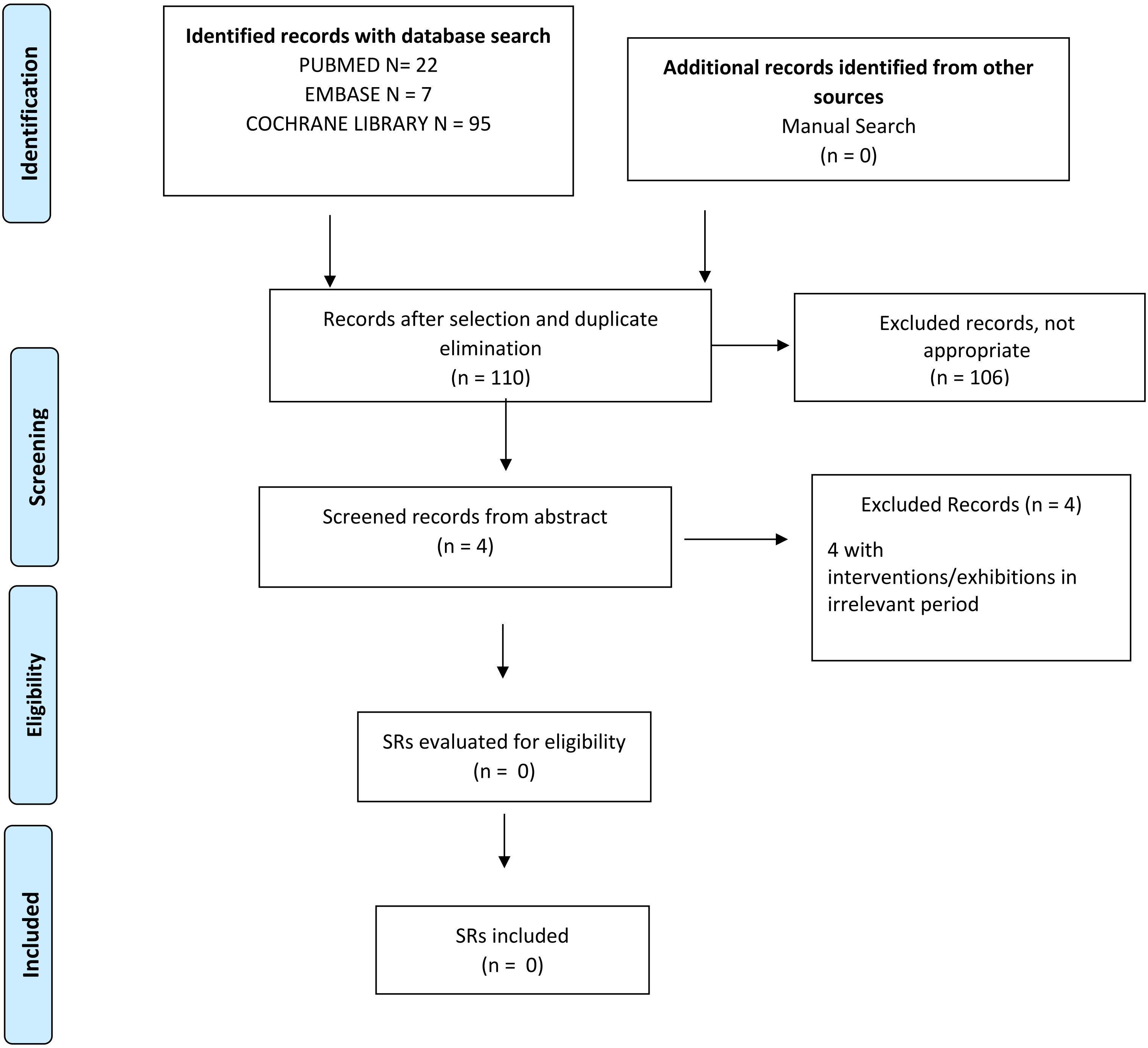

Figure a4.29. Studies flow diagram. Responsive and non-responsive complementary feeding and DM2 development.

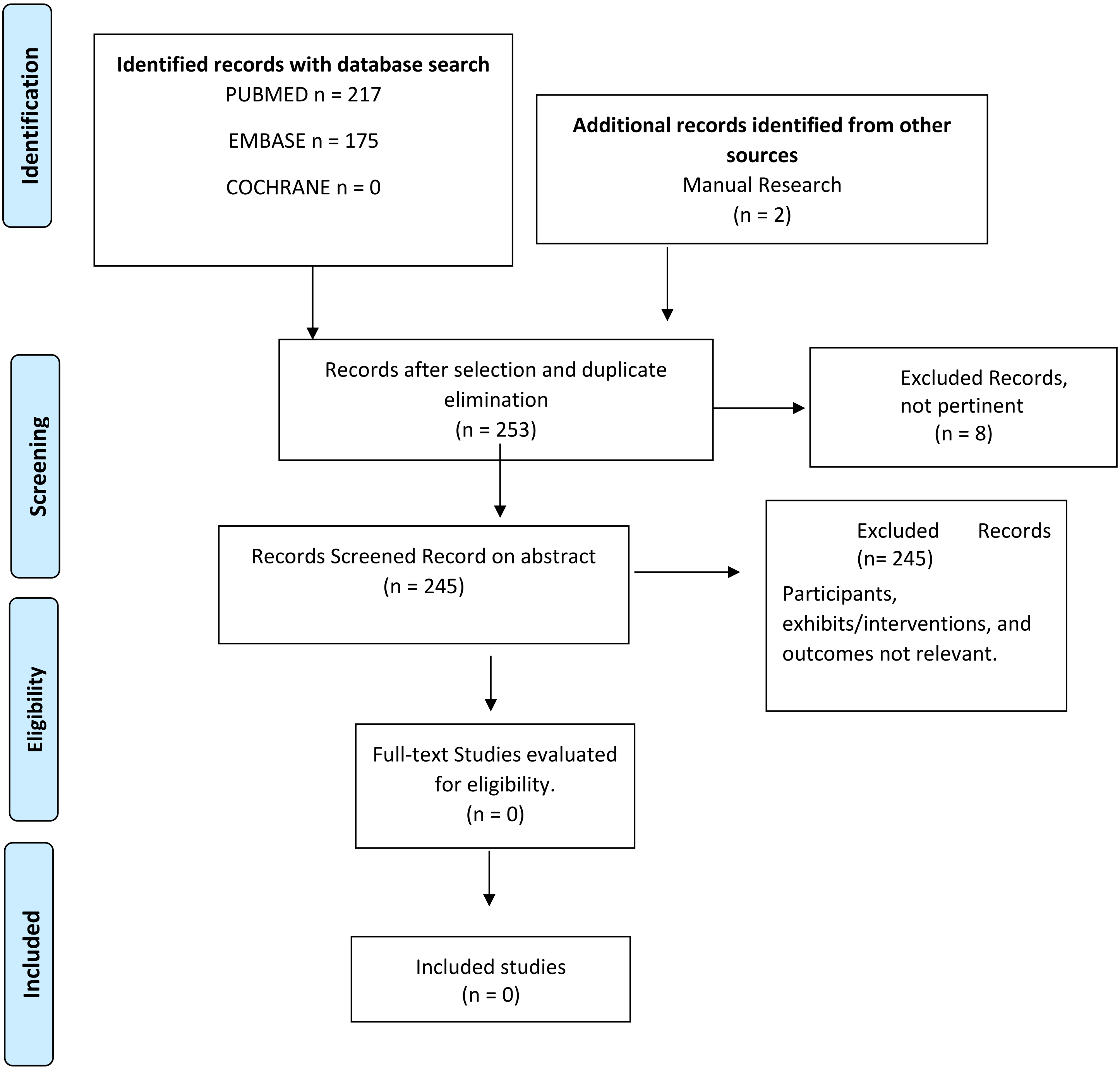

A4. Responsive and Non-Responsive Complementary Feeding. Hypertension.

- Is ReCF able to impact the development of hypertension at later ages?

- Is traditional CF able to affect the development of hypertension at later ages?

PICOs

P In a healthy infant
I responsive complementary feeding during the Complementary Feeding period
C compared to traditional complementary feeding during the period of Complementary Feeding
O results in a different risk of developing Hypertension at later ages?

KEY WORDS

Population

[infant]/lim
[child]/lim
[preschool]/lim
[adolescent]/lim

Exposure Factors / Comparison

"responsive feeding"[All Fields]
"non-responsive feeding"[All Fields]
"responsiveness"[All Fields]
"Weaning"[All Fields]
"Infant Nutritional Physiological Phenomena"[MeSH]
"complementary feeding"[All Fields]
"Feeding Behavior"[All Fields])
"Risk"[Mesh]
"Primary Prevention"[Mesh]
"prevention and control" [Subheading]
OR ("self-weaning"[All Fields] OR "self weaning"[All Fields] OR "baby led weaning"[All Fields] OR "baby-led weaning"[All Fields]))

Outcomes

"Hypertension"[Mesh]
"arterial hypertension"[All Fields]
"elevated blood pressure"[All Fields])

Guidelines search

SOCIETY GUIDELINE LINKS:

National Guideline Clearinghouse (NGC) https://www.ahrq.gov/gam/index.html
Canadians Medical Association (CMA) https://www.cma.ca/clinicalresources/practiceguidelines
National Guideline Centre (NGC) - National Institute of Health and Care Excellence (NICE) https://www.rcplondon.ac.uk/about-us/what-we-do/national-guideline-centre-ngc
Scottish Intercollegiate Guidelines Network (SIGN) https://www.sign.ac.uk/our-guidelines.html
Australian Clinical Practice Guidelines (ACPG) https://www.clinicalguidelines.gov.au/
New Zealand Guidelines Group (NZGG) https://www.health.govt.nz/about-ministry/ministry-health-websites/new-zealand-guidelines-group
American Academy of Pediatrics (AAP) https://www.aap.org/en-us/Pages/Default.aspx
EPA/UNEPSA http://www.epa-unepsa.org/
Guidelines International Network https://g-i-n.net/
Società Italiana di Pediatria (SIP) http://www-sip.it/
Società Italiana di Pediatria Preventiva e Sociale (SIPPS) https://www.sipps.it/
European Society of Hypertension (ESH) https://www.eshonline.org/
Hypertension Canada https://hypertension.ca/
Japanese Society of Hypertension (JSH) https://www.jpnsh.jp/index_e.html

PUBMED

#1

("Hypertension"[Mesh] OR "arterial hypertension"[All Fields] OR "elevated blood pressure"[All Fields]) AND ("Risk"[Mesh] OR ("Primary Prevention"[Mesh]) OR "prevention and control"[Subheading]) AND ((Guideline[ptyp] OR Practice Guideline[ptyp]) AND ("infant"[MeSH Terms] OR "child"[MeSH Terms] OR "adolescent"[MeSH Terms])) AND "2014/09/15"[PDat] : "2021/03/15"[PDat]

"Hypertension"[Mesh] OR "arterial hypertension"[All Fields] OR "elevated blood pressure"[All Fields] **AND** ("responsive feeding"[All Fields] OR "non-responsive feeding"[All Fields] OR "responsiveness"[All Fields] OR "Weaning"[All Fields] OR "Infant Nutritional Physiological Phenomena"[MeSH] OR "complementary feeding"[All Fields] OR "Feeding Behavior"[All Fields]) AND ((Guideline[ptyp] OR Practice Guideline[ptyp]) AND "2014/09/15"[PDat] : "2021/03/15"[PDat]

EMBASE

#1

('hypertension'/exp OR 'blood pressure'/exp OR 'elevated blood pressure'/exp) AND ('complementary feeding'/exp OR 'weaning'/exp OR weaning OR 'responsiveness' OR 'responsive feeding' OR 'non responsive feeding') AND [2014-2021]/py AND ([adolescent]/lim OR [child]/lim OR [infant]/lim OR [preschool]/lim OR [school]/lim OR [young adult]/lim) AND 'practice guideline'/de

#2

('hypertension'/exp OR 'blood pressure'/exp OR 'elevated blood pressure'/exp) AND [2014-2021]/py AND 'practice guideline'/de AND ([adolescent]/lim OR [child]/lim OR [infant]/lim OR [preschool]/lim OR [school]/lim)

Systematic Reviews search

COCHRANE LIBRARY

“Hypertension” in Title Abstract Keyword
Custom date range Topics: 15.09.2014-15.03.2021

EMBASE

#1

('hypertension'/exp OR 'blood pressure'/exp OR 'elevated blood pressure'/exp) AND ('complementary feeding'/exp OR 'weaning'/exp OR weaning OR 'responsiveness' OR 'responsive feeding' OR 'non responsive feeding') AND ([cochrane review]/lim OR [systematic review]/lim OR [meta analysis]/lim) AND [2009-2021]/py AND ([adolescent]/lim OR [child]/lim OR [infant]/lim OR [preschool]/lim OR [school]/lim OR [young adult]/lim)

PUBMED

#1

("Hypertension"[Mesh] OR "arterial hypertension"[All Fields] OR "elevated blood pressure"[All Fields] AND ("responsive feeding"[All Fields] OR "non-responsive feeding"[All Fields] OR "responsiveness"[All Fields] OR "Weaning"[All Fields] OR "Infant Nutritional Physiological Phenomena"[MeSH] OR "complementary feeding"[All Fields] OR "Feeding Behavior"[All Fields]) AND ((Meta-Analysis[ptyp] OR systematic[sb]) AND "2009/09/09"[PDat] : "2021/03/15"[PDat] AND ("infant"[MeSH Terms] OR "child"[MeSH Terms] OR "adolescent"[MeSH Terms]))

Studies search

PUBMED

#1

"Hypertension"[Mesh] OR "arterial hypertension"[All Fields] OR "elevated blood pressure"[All Fields] AND ("responsive feeding"[All Fields] OR "non-responsive feeding"[All Fields] OR "responsiveness"[All Fields] OR "Weaning"[All Fields] OR "Infant Nutritional Physiological Phenomena"[MeSH] OR "complementary feeding"[All Fields] OR "Feeding Behavior"[All Fields]) AND ((Clinical Study[ptyp] OR Clinical Trial[ptyp] OR Comparative Study[ptyp] OR Controlled Clinical Trial[ptyp] OR Multicenter Study[ptyp] OR Observational Study[ptyp] OR Pragmatic Clinical Trial[ptyp] OR Randomized Controlled Trial[ptyp]) AND ("infant"[MeSH Terms] OR "child"[MeSH Terms] OR "adolescent"[MeSH Terms]))

EMBASE

#1

('hypertension'/exp OR 'blood pressure'/exp OR 'elevated blood pressure'/exp) AND ('complementary feeding'/exp OR 'weaning'/exp OR weaning OR 'responsiveness' OR 'responsive feeding' OR 'non responsive feeding') AND ('clinical study'/de OR 'clinical trial'/de OR 'cohort analysis'/de OR 'comparative effectiveness'/de OR 'comparative study'/de OR 'controlled clinical trial'/de OR 'controlled study'/de OR 'cross-sectional study'/de OR 'double blind procedure'/de OR 'multicenter study'/de OR 'observational study'/de OR 'prospective study'/de OR 'randomized controlled trial'/de OR 'randomized controlled trial (topic)'/de OR 'retrospective study'/de) AND ([adolescent]/lim OR [child]/lim OR [infant]/lim OR [preschool]/lim OR [school]/lim OR [young adult]/lim)

COCHRANE

hypertension in Title Abstract Keyword - in Trials with 'Child Health' in Cochrane Groups (Word variations have been searched)

Figure a4.30. Guidelines search flow diagram. Responsive and Non-Responsive Complementary Feeding. Hypertension.

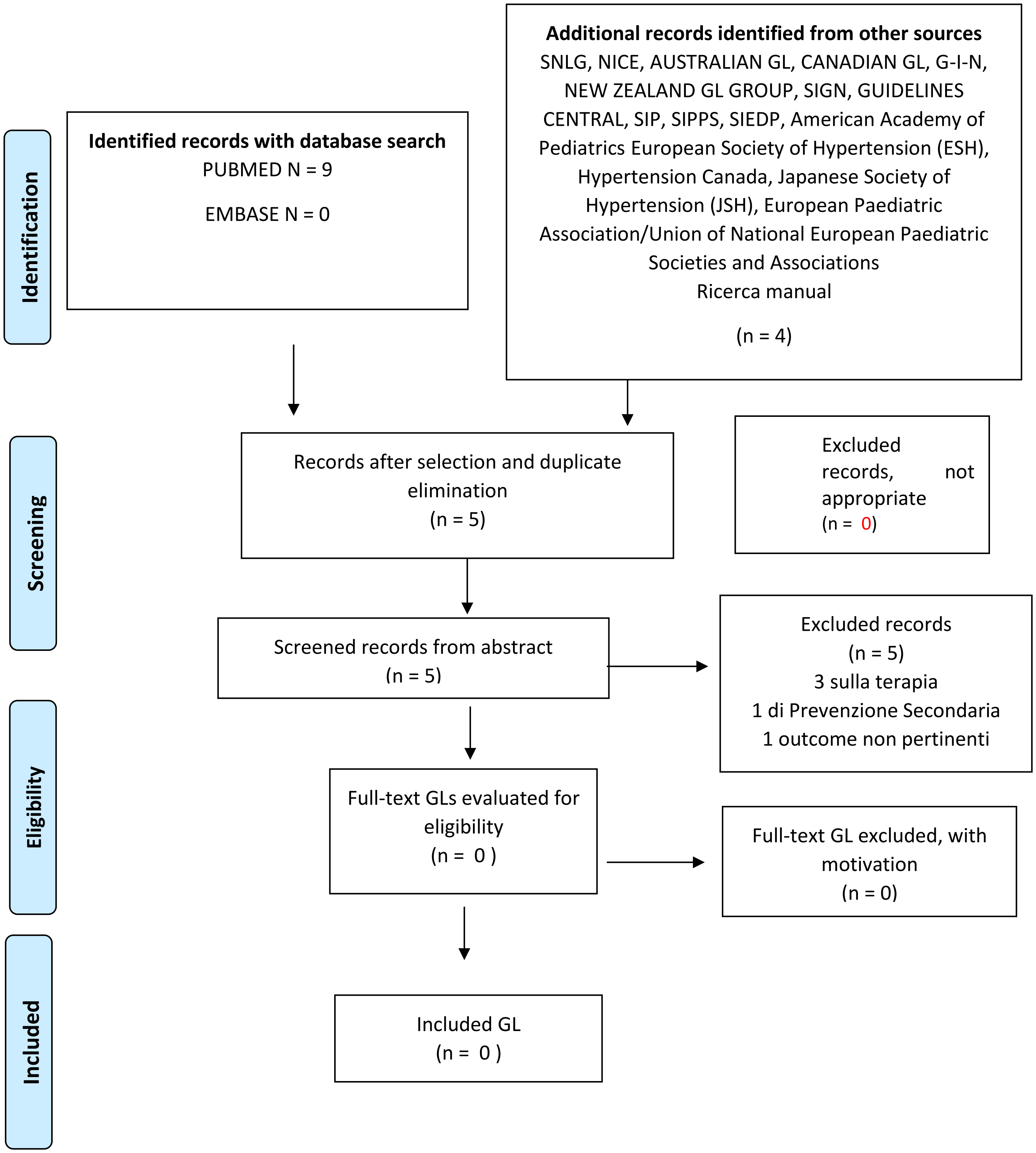

Figure a4.31. SRs search flow diagram. Responsive and Non-Responsive Complementary Feeding. Hypertension.

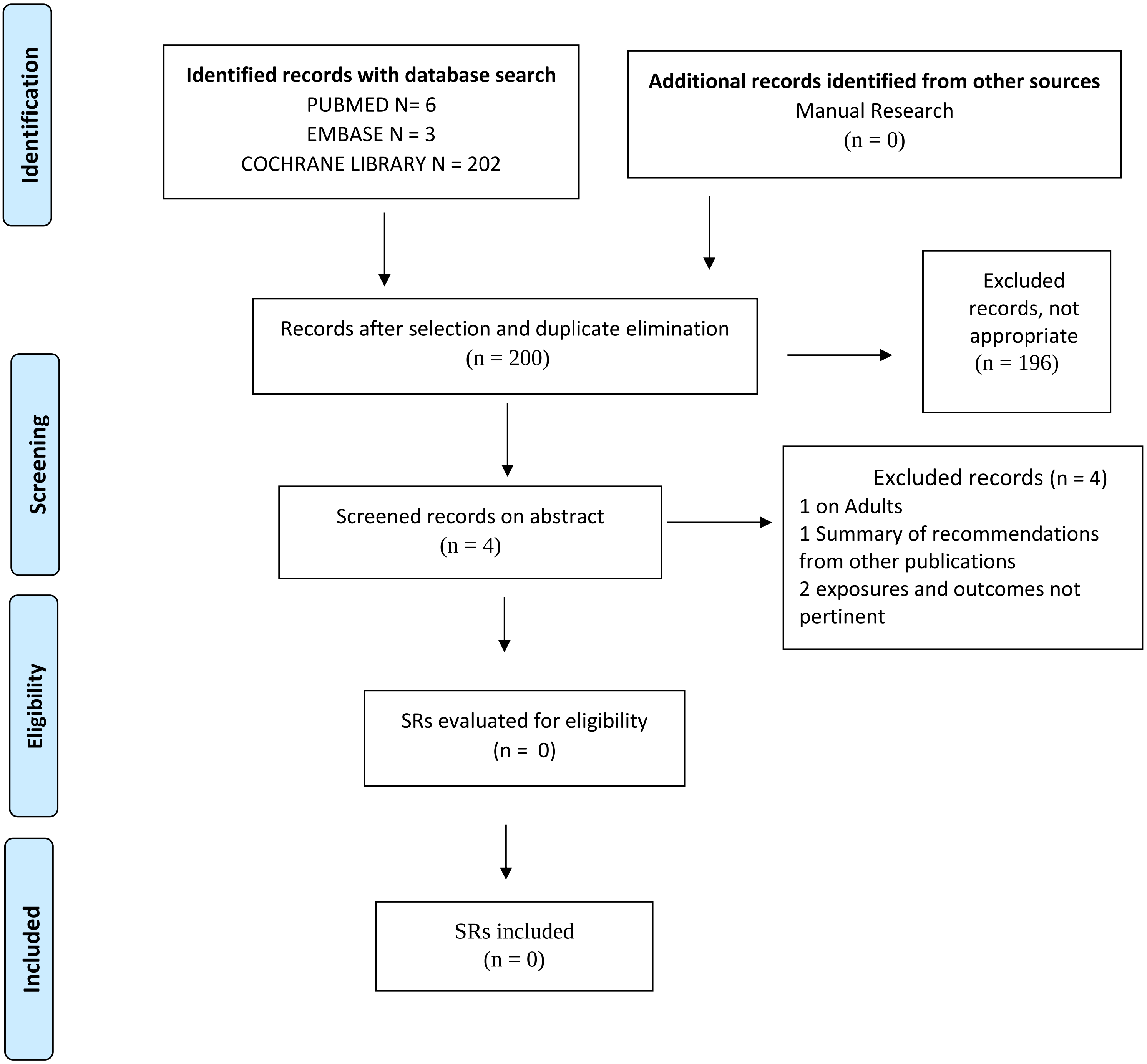

Figure a4.32. Studies flow diagram. Responsive and Non-Responsive Complementary Feeding. Hypertension.

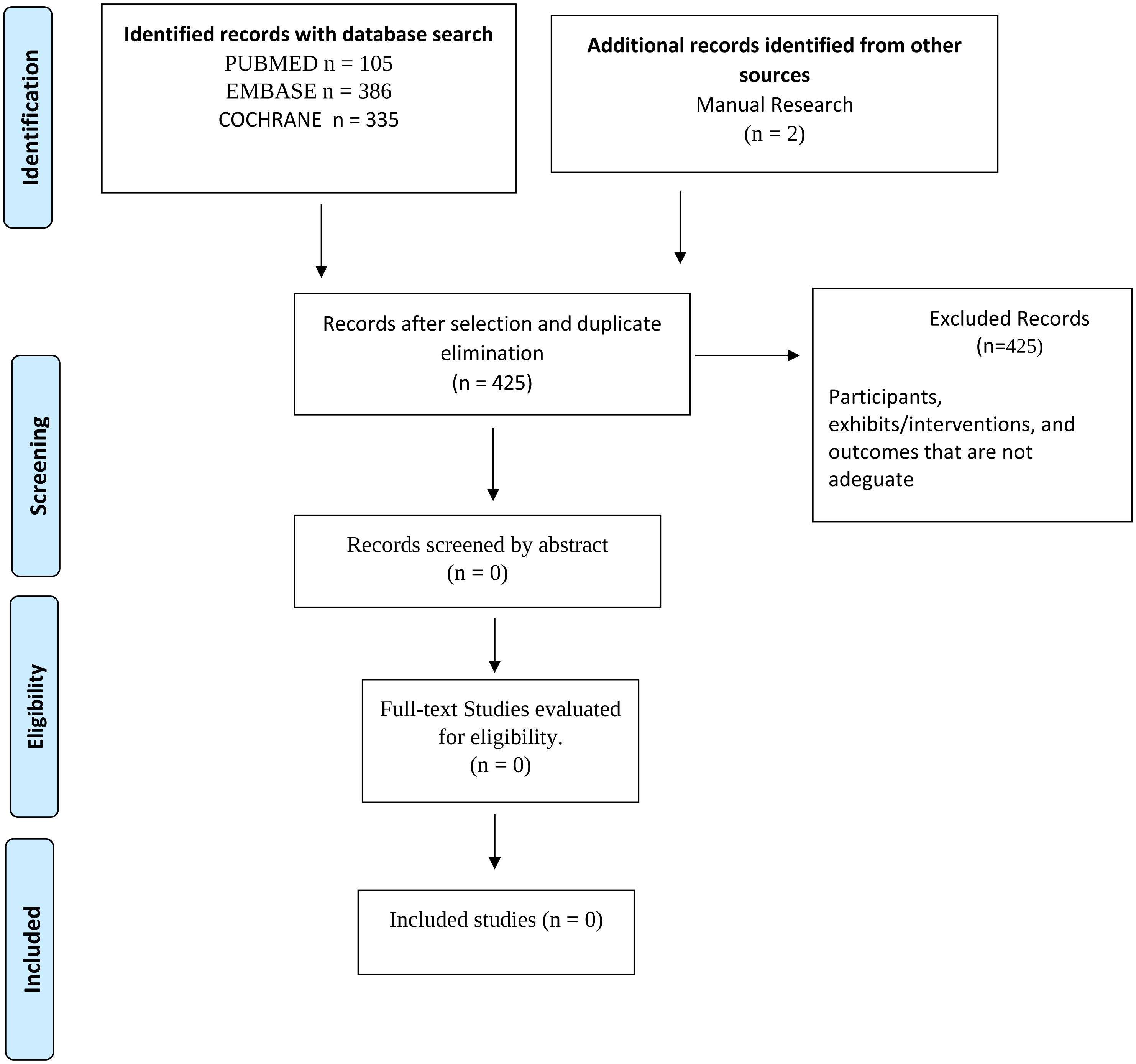

A4. Responsive and non-responsive complementary feeding. Caries.

- ***Is responsive complementary feeding able to influence the development of dental caries in later life?***
- ***Is traditional complementary feeding able to influence the development of dental caries in later life?***

PICOs

P In the healthy infant
I responsive complementary feeding
C compared with traditional complementary feeding
O results in a different risk of developing dental caries in later ages?

KEY WORDS

Population

"infant"[MeSH Terms]
"child"[MeSH Terms]
"adolescent"[MeSH Terms]

Exposure Factors / Comparison

"responsive feeding"[All Fields]
"non-responsive feeding"[All Fields]
“responsiveness”[All Fields]
"Weaning"[All Fields] OR
"Infant Nutritional Physiological Phenomena"[MeSH]
“complementary feeding”[All Fields]
"Feeding Behavior"[All Fields])

Outcomes

"Dental Caries"[Mesh])
"DMF Index"[Mesh]

Guidelines search

“caries”
“caries children”

Official sites of scientific or institutional societies

Società Italiana Odontoiatria Infantile https://www.sioi.it/
Ministero della Salute. Italia.
https://www.salute.gov.it/portale/news/p3_2_1.jsp?lingua=italiano&menu=notizie&area=Sorriso%20salute
American Academy of Pediatric Dentistry https://www.aapd.org/
American Academy of Pediatrics (AAP) https://www.aap.org/en-us/Pages/Default.aspx

SOCIETY GUIDELINE LINKS:

National Guideline Clearinghouse (NGC) https://www.ahrq.gov/gam/index.html
Canadians Medical Association (CMA) https://www.cma.ca/clinicalresources/practiceguidelines
National Guideline Centre (NGC) - National Institute of Health and Care Excellence (NICE) https://www.rcplondon.ac.uk/about-us/what-we-do/national-guideline-centre-ngc
Scottish Intercollegiate Guidelines Network (SIGN) https://www.sign.ac.uk/our-guidelines.html
Australian Clinical Practice Guidelines (ACPG) https://www.clinicalguidelines.gov.au/
New Zealand Guidelines Group (NZGG) https://www.health.govt.nz/about-ministry/ministry-health-websites/new-zealand-guidelines-group
EPA/UNEPSA http://www.epa-unepsa.org/
Guidelines International Network https://g-i-n.net/

PUBMED

#1

(("DMF Index"[Mesh] OR "Dental Caries"[Mesh]) OR "Dental Caries Susceptibility"[Mesh] OR "early childhood caries"[All Fields] OR "ECC"[All Fields]) OR "caries prevention" OR "dental caries prevention" OR "dental caries risk" OR "dental caries children" AND ("child"[MeSH Terms] OR "child"[All Fields] OR "children"[All Fields])) AND ((Guideline[ptyp] OR Practice Guideline[ptyp]) AND "2014/09/15"[PDat] : "2021/03/15"[PDat])

EMBASE

#1

('dental caries'/exp OR 'caries prevention'/exp) AND [2014-2021]/py AND ([adolescent]/lim OR [child]/lim OR [preschool]/lim OR [school]/lim OR [young adult]/lim) AND 'practice guideline'/de

Systematic Reviews search

COCHRANE LIBRARY

"Dental caries" in Title and Abstract

Custom date range Topics: 15.09.2009-15.03.2021

EMBASE

#1

('dental caries'/exp OR 'dental caries' OR 'caries prevention'/exp OR 'caries prevention') AND ('complementary feeding'/exp OR 'complementary feeding' OR 'feeding behavior'/exp OR 'feeding behavior' OR 'weaning'/exp OR 'weaning' OR 'responsiveness'/exp OR 'responsiveness' OR 'responsive feeding' OR 'non responsive feeding') AND [2009-2021]/py AND ([systematic review]/lim OR [meta analysis]/lim) AND ([adolescent]/lim OR [child]/lim OR [preschool]/lim OR [school]/lim OR [young adult]/lim)

- ('dental caries'/exp OR 'caries prevention'/exp) AND ('risk'/exp OR 'risk factor'/exp) AND [2009-2021]/py AND ([systematic review]/lim OR [meta analysis]/lim) AND ([adolescent]/lim OR [child]/lim OR [preschool]/lim OR [school]/lim OR [young adult]/lim)

PUBMED

#1

("Risk"[Mesh] AND ("2009/08/24"[PDat] : "2021/03/15"[PDat] AND ("infant"[MeSH Terms] OR "child"[MeSH Terms] OR "adolescent"[MeSH Terms]))) AND (((("DMF Index"[Mesh] OR "Dental Caries"[Mesh]) OR "Dental Caries Susceptibility"[Mesh] OR "early childhood caries"[All Fields] OR "ECC"[All Fields]) OR "caries prevention"[All Fields] OR "dental caries prevention"[All Fields] OR "dental caries risk"[All Fields] OR ("dental"[All Fields] AND "caries"[All Fields]) OR "dental caries"[All Fields]) AND ("child"[MeSH Terms] OR "child"[All Fields] OR "children"[All Fields]))) AND ((systematic[sb] OR Meta-Analysis[ptyp])

#2

("responsive feeding"[All Fields] OR "non-responsive feeding"[All Fields] OR "responsiveness"[All Fields] OR "Weaning"[All Fields] OR "Infant Nutritional Physiological Phenomena"[MeSH] OR "complementary feeding"[All Fields] OR "Feeding Behavior"[All Fields]) AND (("DMF Index"[Mesh] OR "Dental Caries"[Mesh]) OR "Dental Caries Susceptibility"[Mesh] OR "early childhood caries"[All Fields] OR "ECC"[All Fields] OR "caries prevention"[All Fields] OR "dental caries prevention"[All Fields] OR "dental caries risk"[All Fields] OR (("dental caries"[MeSH Terms] OR ("dental"[All Fields] AND "caries"[All Fields]) OR "dental caries"[All Fields]) AND ("child"[MeSH Terms] OR "child"[All Fields] OR "children"[All Fields]))) AND ((systematic[sb] OR Meta-Analysis[ptyp]) AND "2009/09/15"[PDat] : "2021/03/15"[PDat])

Studies search

PUBMED

#1

("DMF Index"[Mesh] OR "Dental Caries"[Mesh]) OR "Dental Caries Susceptibility"[Mesh] OR "early childhood caries"[All Fields] OR "ECC"[All Fields] OR "dental caries prevention"[All Fields] OR "dental caries risk"[All Fields] OR (("dental caries"[MeSH Terms] OR ("dental"[All Fields] AND

"caries"[All Fields]) OR "dental caries"[All Fields]) **AND** ("child"[MeSH Terms] OR "child"[All Fields] OR "children"[All Fields])) **AND** ("responsive feeding"[All Fields] OR "non-responsive feeding"[All Fields] OR "responsiveness"[All Fields] OR "Weaning"[All Fields] OR "Infant Nutritional Physiological Phenomena"[MeSH] OR "complementary feeding"[All Fields] OR "Feeding Behavior"[All Fields]) **AND** ((Clinical Study[ptyp] OR Clinical Trial[ptyp] OR Comparative Study[ptyp] OR Controlled Clinical Trial[ptyp] OR Multicenter Study[ptyp] OR Observational Study[ptyp] OR Pragmatic Clinical Trial[ptyp] OR Randomized Controlled Trial[ptyp]) AND ("infant"[MeSH Terms] OR "child"[MeSH Terms] OR "adolescent"[MeSH Terms]))

EMBASE

#1

('dental caries'/exp OR 'dental caries' OR 'caries prevention'/exp OR 'caries prevention') **AND** ('complementary feeding'/exp OR 'complementary feeding' OR 'feeding behavior'/exp OR 'feeding behavior' OR 'weaning'/exp OR 'weaning' OR 'responsiveness'/exp OR 'responsiveness' OR 'responsive feeding' OR 'non responsive feeding') **AND** ([adolescent]/lim OR [child]/lim OR [infant]/lim OR [preschool]/lim OR [school]/lim OR [young adult]/lim) **AND** ('case control study'/de OR 'clinical trial'/de OR 'cohort analysis'/de OR 'comparative study'/de OR 'controlled clinical trial'/de OR 'controlled study'/de OR 'cross-sectional study'/de OR 'double blind procedure'/de OR 'evidence based dentistry'/de OR 'longitudinal study'/de OR 'observational study'/de OR 'prospective study'/de OR 'randomized controlled trial'/de OR 'retrospective study'/de)

Figure a4.33. Guidelines search flow diagram. Responsive and non-responsive complementary feeding. Caries.

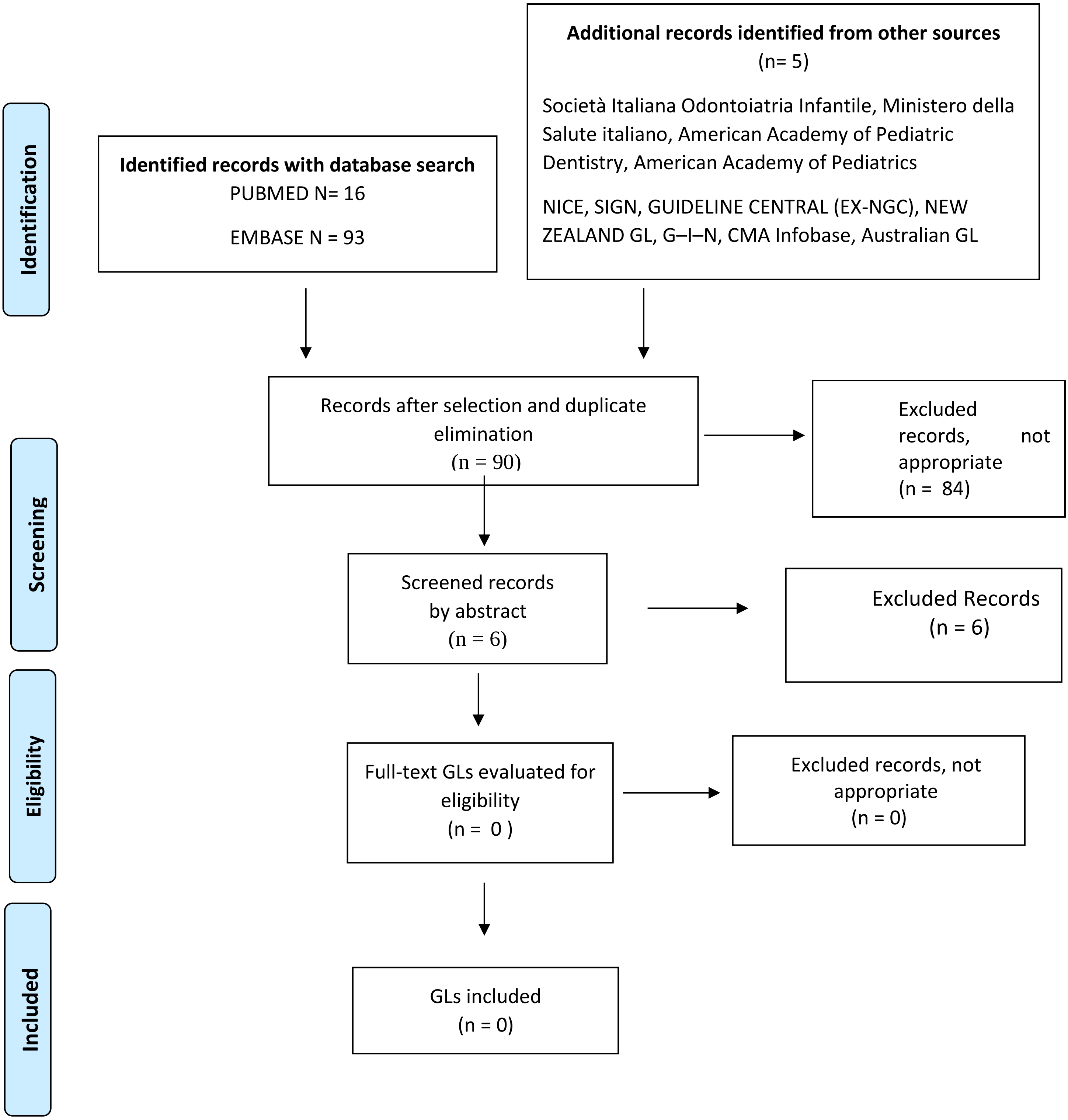

Figura a4.34. SRs search flow diagram. Responsive and non-responsive complementary feeding. Caries.

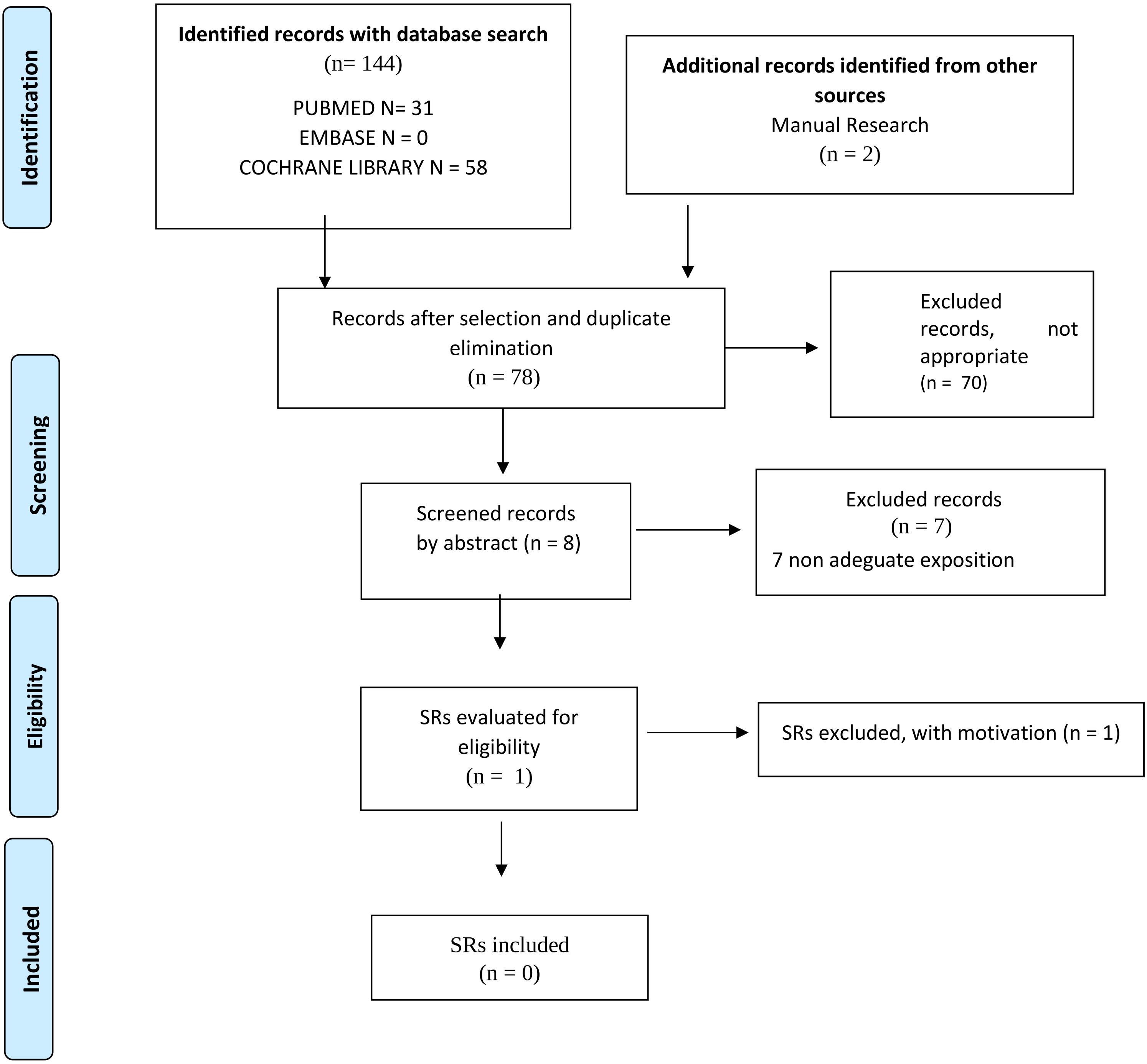

Figure a4.35. Studies flow diagram. Responsive and non-responsive complementary feeding. Caries.

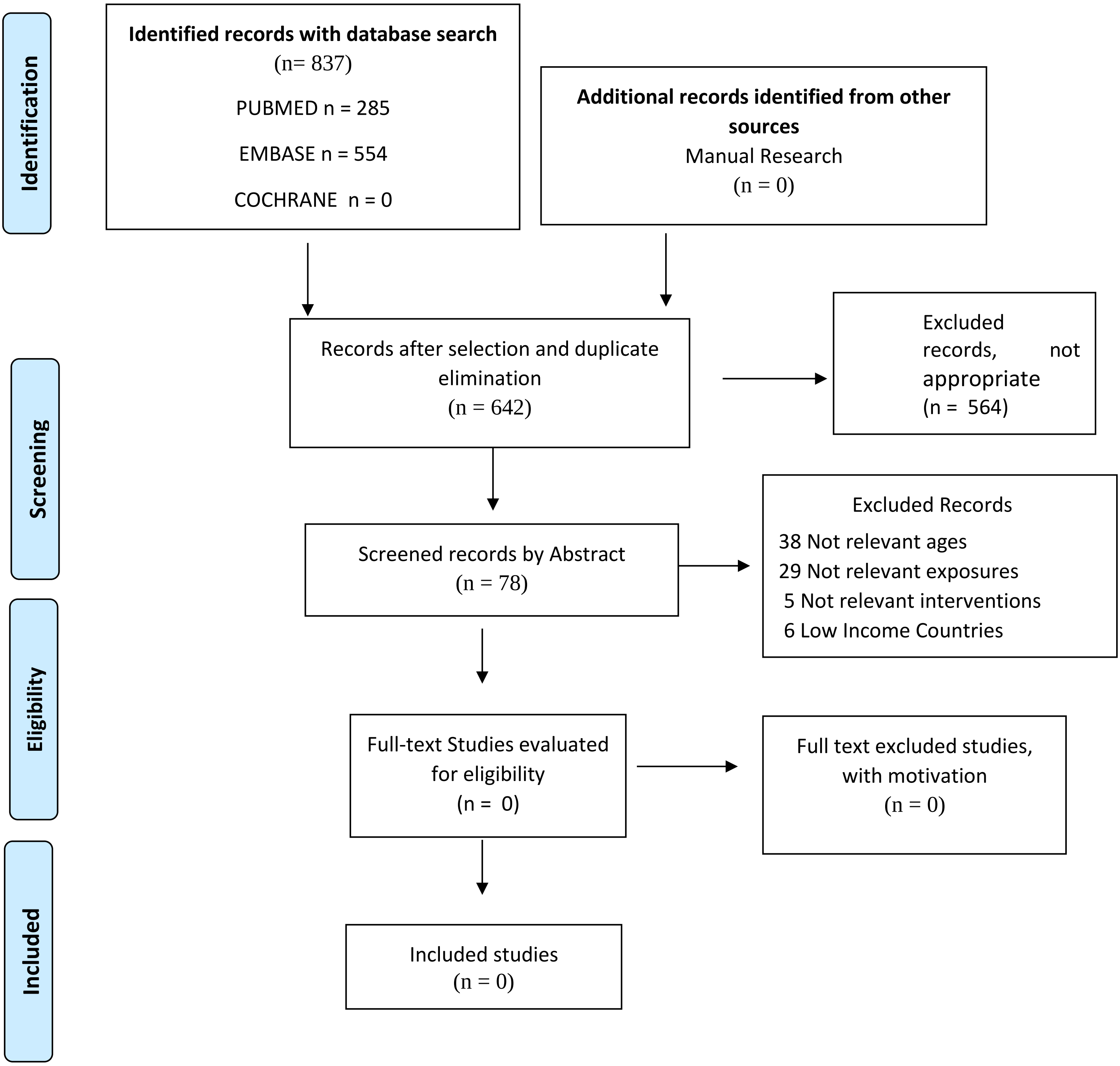

A4. METHODOLOGICAL ASSESSMENT

A4. ReCF / nReCF and caries risk.

- ***Is responsive complementary feeding able to influence the development of dental caries in later life?***
- ***Is traditional complementary feeding able to influence the development of dental caries in later life?***

Responsive and Non-Responsive Complementary Feeding
P In the healthy infant
I responsive complementary feeding
C compared with traditional complementary feeding
O results in a different risk of developing dental caries in later ages?

Clinical Guidelines and Guidance Documents Appraisal, Systematic reviews and Studies

None included.

Table a4.33. SRs excluded with motivation.

SRs excluded	Reason for exclusion
Leong et al. 2013 [53]	Does not include work with Exhibits or Behavioral Interventions.

Appendix 4. References

Recommendations 19-21. BLW/BLISS, growth, and risk of overweight/obesity

1. D'Auria E, Bergamini M, Staiano A, et al.; Italian Society of Pediatrics. Baby-led weaning: what a systematic review of the literature adds on. Ital J Pediatr. 2018 May 3;44(1):49
2. Harrison M, Brodribb W, Hepworth J. A qualitative systematic review of maternal infant feeding practices in transitioning from milk feeds to family foods. Matern Child Nutr. 2017 Apr;13(2):e12360.
3. Arikpo D, Edet ES, Chibuzor MT, Odey F, Caldwell DM. Educational interventions for improving primary caregivercomplementary feeding practices for children aged 24 months and under. Cochrane Database of Systematic Reviews 2018, Issue 5. Art.No.: CD011768
4. Gomez M.S., Novaes A.P.T., da Silva J.P., Guerra L.M., de Fátima Possobon R. Baby-led weaning, an overview of the new approach to food introduction: Integrative literature review.. Revista Paulista de Pediatria 2020
5. Townsend E, et al. Baby knows best? The impact of weaning style on food preferences and body mass index in early childhood in a case-controlled sample. BMJ OPEN 2012;2:e000298.
6. Brown A, Lee DW, Early influences on child satiety-responsiveness: the role of weaning style. Pediatr Obes. 2015 Feb;10(1):57-66.
7. Dogan E, Yilmaz G, Caylan N, et al. Baby-led complementary feeding: randomized controlled study. Pediatr Int. 2018;60(12):1073-1080.
8. Taylor RW, Williams SM, Fangupo LJ, et al. Effect of a baby-led approach to complementary feeding on infant growth and overweight: a randomized clinical trial. JAMA Pediatr. 2017;171:838-46.

Recommendations 22-23. ReCF/nReCF and growth

9. Gerritsen S., Wall C. How We Eat – Reviews of the evidence on food and eating behaviours related to diet and body size. A report commissioned by the Ministry of Health. 31 May 2017. Available at https://www.health.govt.nz/publication/how-we-eat-reviews-evidence-food-and-eating-behaviours-related-diet-and-body-size
10. Spill MK, Callahan EH, Shapiro MJ, et al. Caregiver feeding practices and child weight outcomes: a systematic review. Am J Clin Nutr. 2019;109(Suppl.7):990S-1002S
11. Daniels LA, Mallan KM, Battistutta D, et al. Evaluation of an intervention to promote protective infant feeding practices to prevent childhood obesity: outcomes of the NOURISH RCT at 14 months of age and 6 months post the first of two intervention modules. Int J Obes (Lond). 2012;36(10):1292-8
12. Paul IM, Savage JS, Anzman-Frasca S, et al. Effect of a Responsive Parenting Educational Intervention on Childhood Weight Outcomes at 3 Years of Age: The INSIGHT Randomized Clinical Trial. JAMA. 2018;320(5):461-8.
13. Savage JS, Birch LL, Marini M, Anzman-Frasca S, Paul IM. Effect of the INSIGHT responsive parenting intervention on rapid infant weight gain and overweight status at age 1 year: a randomized clinical trial. JAMA Pediatr. 2016;170(8):742-9
14. Wright CM, Parkinson KN, Drewett RF. How does maternal and child feeding behavior relate to weight gain and failure to thrive? Data from a prospective birth cohort. Pediatrics. 2006;117(4):1262-9
15. Chaidez V, McNiven S, Vosti SA, Kaiser LL. Sweetened food purchases and indulgent feeding are associated with increased toddler anthropometry. J Nutr Educ Behav. 2014;46(4):293-8
16. Dinkevich E, Leid L, Pryor K, et al. Mothers' feeding behaviors in infancy: do they predict child weight trajectories? Obesity (Silver Spring). 2015;23(12):2470-6
17. Hittner JB, Johnson C, Tripicchio G, Faith MS. Infant emotional distress, maternal restriction at a home meal, and child BMI gain through age 6 years in the Colorado Adoption Project. Eat Behav. 2016;21:135-41

18. Stifter CA, Moding KJ. Understanding and measuring parent use of food to soothe infant and toddler distress: A longitudinal study from 6 to 18 months of age. Appetite. 2015;95:188-96
19. Paul IM, Savage JS, Anzman SL, et al. Preventing obesity during infancy: a pilot study. Obesity (Silver Spring). 2011;19(2):353-61
20. Daniels LA, Mallan KM, Nicholson JM, Battistutta D, Magarey A. Outcomes of an early feeding practices intervention to prevent childhood obesity. Pediatrics. 2013;132(1):e109-18
21. Daniels LA, Mallan KM, Nicholson JM, et al. An early feeding practices intervention for obesity prevention. Pediatrics. 2015;136(1):e40-9
22. Worobey J, Lopez MI, Hoffman DJ. Maternal behavior and infant weight gain in the first year. J Nutr Educ Behav. 2009;41(3):169-75
23. Ma JQ, Zhou LL, Hu YQ, Liu SS, Sheng XY. Association between feeding practices and weight status in young children. BMC Pediatr. 2015;15:97
24. Shi C, Li N, Dong J, et al. Association between maternal nonresponsive feeding practice and child's eating behavior and weight status: children aged 1 to 6 years. Eur J Pediatr. 2017;176(12):1603-12

Recommendations 24-25. ReCF/nReCF and risk of overweight/obesity

25. Canada's Dietary Guidelines for Health Professionals and Policy Makers. https://food-guide.canada.ca/static/assets/pdf/CDG-EN-2018.pdf
26. Dereń K, Weghuber D, Caroli M, et al. Consumption of Sugar-Sweetened Beverages in Paediatric Age: A Position Paper of the European Academy of Paediatrics and the European Childhood Obesity Group. Ann Nutr Metab. 2019;74(4):296-302.
27. Fewtrell M, Bronsky J, Campoy C, et al. Complementary Feeding: A Position Paper by the European Society for Paediatric Gastroenterology, Hepatology, and Nutrition (ESPGHAN). Committee on Nutrition. J Pediatr Gastroenterol Nutr. 2017 Jan;64(1):119-132
28. Guideline: Sugars intake for adults and children. Geneva: World Health Organization; 2015.
29. Koletzko B, Godfrey KM, Poston L, et al. Nutrition During Pregnancy, Lactation and Early Childhood and its Implications for Maternal and Long-Term Child Health: The Early Nutrition Project Recommendations. Ann Nutr Metab. 2019;74(2):93-106. doi:10.1159/000496471
30. NICE guideline 2015. Preventing excess weight gain. nice.org.uk/guidance/ng7
31. Romero-Velardea E, Villalpando-Carrión S, Pérez-Lizaur AB, et al. Consenso para las prácticas de alimentación complementaria en lactantes sanos. Bol Med Hosp Infant Mex. 2016;73(5):338---356
32. Schwarzenberg SJ, Georgieff MK, AAP Committee on Nutrition. Advocacy for Improving Nutrition in the First 1000 Days To Support Childhood Development and Adult Health. Pediatrics. 2018;141(2):e20173716
U.S. Department of Health and Human Services and U.S. Department of Agriculture. 2015–2020 Dietary Guidelines for Americans. 8th Edition. December 2015. Available at http://health.gov/dietaryguidelines/2015/guidelines/.
33. Valerio G, Saggese G, Maffeis C, et al. Diagnosi, trattamento e prevenzione dell'obesità del bambino e dell'adolescente. http://www.siedp.it/files/Doc.ConsensusObesita__2017.pdf
34. Heyman MB, Abrams SA, AAP Section on Gastroenterology, Hepatology, and Nutrition, AAP Committee on Nutrition. Fruit Juice in Infants, Children, and Adolescents: Current Recommendations. Pediatrics. 2017;139(6):e20170967
35. Redsell SA, Edmonds B, Swift JA, et al. Systematic review of randomised controlled trials of interventions that aim to reduce the risk, either directly or indirectly, of overweight and obesity in infancy and early childhood. Matern Child Nutr. 2016 Jan;12(1):24-38
36. Sokol RL, Qin B, Poti JM. Parenting styles and body mass index: a systematic review of prospective studies among children. Obes Rev. 2017 Mar;18(3):281-292.
37. Matvienko-Sikar K, Toomey E, Delaney L, et al.; Choosing Healthy Eating for Infant Health (CHErIsH) study team. Effects of healthcare professional delivered early feeding interventions on feeding practices and dietary intake: A systematic review. Appetite. 2018 Apr 1;123:56-71.
38. Woo Baidal JA, Locks LM, Cheng ER, Blake-Lamb TL, Perkins ME, Taveras EM. Risk Factors for Childhood Obesity in the First 1,000 Days: A Systematic Review. Am J Prev Med. 2016 Jun;50(6):761-779.

39. Bonilla C, Híjar G, Márquez D, Aramburú A, Aparco JP, Gutiérrez EL. Intervenciones para prevenir la aparición de sobrepeso y obesidad en niños menores de cinco años [Interventions to prevent the development of overweight and obesity in children younger than five years]. Rev Peru Med Exp Salud Publica. 2017 Oct-Dec;34(4):682-689. Spanish.
40. Blake-Lamb TL, Locks LM, Perkins ME, Woo Baidal JA, Cheng ER, Taveras EM. Interventions for Childhood Obesity in the First 1,000 Days A Systematic Review. Am J Prev Med. 2016 Jun;50(6):780-789.
41. Machuca H, Arevalo S, Hackley B, et al. Well Baby Group Care: Evaluation of a Promising Intervention for Primary Obesity Prevention in Toddlers. Child Obes. 2016;12(3):171-8
42. Rifas-Shiman SL, Sherry B, Scanlon K, et al. Does maternal feeding restriction lead to childhood obesity in a prospective cohort study? Arch Dis Child. 2011;96(3):265-9
43. Lumeng JC, Ozbeki TN, Appugliese DP, Kaciroti N, Corwyn RF, Bradley RH. Observed assertive and intrusive maternal feeding behaviors increase child adiposity. Am J Clin Nutr. 2012 Mar;95(3):640-7.
44. Thompson AL, Adair LS, Bentley ME. Pressuring and restrictive feeding styles influence infant feeding and size among a low-income African-American sample. Obesity (Silver Spring). 2013;21(3):562-71
45. Morandi A, Tommasi M, Soffiati F, et al. Prevention of obesity in toddlers (PROBIT): a randomised clinical trial of responsive feeding promotion from birth to 24 months. Int J Obes (Lond). 2019 Jul 3.
46. Farrow
47. Gregory JE, Paxton SJ, Brozovic AM. Maternal feeding practices predict fruit and vegetable consumption in young children. Results of a 12-month longitudinal study. Appetite. 2011 Aug;57(1):167-72.

Recommendation 26. BLW/BLISS and choking risk

48. Fangupo LJ, Heath AM, Williams SM, Erickson Williams LW, Morison BJ, Fleming EA, Taylor BJ, Wheeler BJ, Taylor RW. A Baby-Led Approach to Eating Solids and Risk of Choking. Pediatrics. 2016 Oct;138(4):e20160772.
49. Cameron SL, Taylor RW, Heath AL. Parent-led or baby-led? Association between complementary feeding practices and health-related behaviours in a survey of New Zealand families. BMJ Open. 2013;3:e003946
50. Brown A. No difference in self-reported frequency of choking between infants introduced to solid foods using a baby-led weaning or traditional spoon-feeding approach. J Hum Nutr Diet. 2017.
51. Fu X., Conlon C.A., Haszard J.J., Beck K.L., von Hurst P.R., Taylor R.W. & Heath A.-L.M., Food fussiness and early feeding characteristics of infants following Baby-Led Weaning and traditional spoon-feeding in New Zealand: An internet survey, Appetite (2018).
52. Özyüksel G, Soyer T, Üzümcügil F, Yalçln Ş, Ekinci S, Karnak I, Çiftçi A.Ö, Tanyel F.C. Foreign Body Aspiration in Infants: Role of Self-Feeding. Pediatric Allergy Immunology and Pulmonology (2019) 32:2 (52-55).

Recommendation 29. ReCF / nReCF and T2D risk.
None
Recommendation 30. ReCF / nReCF and hypertension risk.
None

Recommendation 31. ReCF / nReCF and caries risk.

53. Leong PM, Gussy MG, Barrow SY, de Silva-Sanigorski A, Waters E. A systematic review of risk factors during first year of life for early childhood caries. Int J Paediatr Dent. 2013 Jul;23(4):235-50.
