# Supplementary material for: Recommendations on Complementary Feeding as a Tool for Prevention of Non-Communicable Diseases (NCDs)—Paper Co-Drafted by the SIPPS, FIMP, SIDOHaD, and SINUPE Joint Working Group
Source: Nutrients. 2022 Jan 7;14(2):257. doi: 10.3390/nu14020257 (PMC8778394; doi:10.3390/nu14020257)
Supplement: Supplementary file 1 [file nutrients-14-00257-s001.zip › nutrients-1513088-supplementary/Supplementary File S5.pdf]

Appendix 5

Recommendations 32-34

A5. Complementary Feeding and Celiac Disease

Key Questions

- Can the period of gluten introduction affect the development of celiac disease?

- Is the development of celiac disease affected by the CF/mode of breastfeeding ratio?

PICOs

a.
P In healthy infants
I the early (before 6th month) or delayed (after 10th-12th month) gluten introduction
C compared with the same timing of introduction for all foods (6th-7th month)
O can it affect the development of celiac disease?
b.
P In healthy infants
I the introduction of gluten associated with breast milk intake
C compared with the introduction of gluten associated with formula intake
O can it affect the development of celiac disease?

KEYWORDS

Population

A. Infant
B. Child

Exposure Factors / Comparison

MeSH Terms/ Text word: weaning, infant, nutritional physiological phenomena; eating; bottle feeding: bottle fed; breast feeding; Glutens; Milk, Human"[Mesh], Breast Milk Expression, Milk Substitutes, Feeding Behavior

A. Feeding, Breast
B. Breastfeeding
C. Breast Feeding, Exclusive
D. Exclusive Breast Feeding
E. Breastfeeding, Exclusive
F. Exclusive Breastfeeding
G. Bottle feeding duration
H. Breast feeding duration
I. Solid food
J. Complementary feeding
K. Glutens
L. "early gluten introduction"

M. “delayed gluten introduction”

Outcomes

"Celiac Disease" [MeSH]

Guidelines search

<u>Temporal limitation</u>: 2014-2019

PUBMED https://www.ncbi.nlm.nih.gov/pubmed/

#1

(("Glutens"[MeSH Terms] OR "Weaning"[MeSH Terms] OR "early gluten introduction"[All Fields] OR (("delay"[All Fields] OR "delayed"[All Fields] OR "delaying"[All Fields] OR "delays"[All Fields]) AND ("Glutens"[MeSH Terms] OR "Glutens"[All Fields] OR "gluten"[All Fields]) AND ("introduction"[All Fields] OR "introductions"[All Fields]))) AND "Celiac Disease"[MeSH Terms]) AND (guideline[Filter] OR practiceguideline[Filter])

#2

((("Weaning"[All Fields]) OR "Infant Nutritional Physiological Phenomena"[MeSH]) OR "complementary feeding"[All Fields]) AND ("Celiac Disease"[Mesh]) AND "2014/05/08"[PDat]:"2021/06/23"[PDat] AND "infant"[MeSH Terms])

#3

((((("Glutens"[Mesh]) OR "Milk, Human"[Mesh]) OR "Breast Feeding"[Mesh]) OR "Breast Milk Expression"[Mesh]) OR "Bottle Feeding"[Mesh]) AND "Celiac Disease"[Mesh] AND "2014/05/08"[PDat]:"2021/06/23"[PDat]) AND (guideline[Filter] OR practiceguideline[Filter])

#4

("Weaning"[All Fields] OR "Infant Nutritional Physiological Phenomena"[MeSH Terms] OR "complementary feeding"[All Fields]) AND "Celiac Disease"[MeSH Terms]

EMBASE https://www.embase.com

#1

('complementary feeding'/exp OR 'weaning'/exp OR weaning) AND ('celiac disease'/exp OR 'celiac disease' OR 'gluten free diet'/exp OR 'gluten free diet' OR 'gluten introduction') AND [2016-2021]/py AND ('practice guideline'/exp OR 'practice guideline' OR 'guideline'/exp OR guideline)

#2

('bottle feeding'/exp OR 'bottle feeding' OR 'bottle feeding duration' OR 'breast feeding'/exp OR 'breast feeding' OR 'breast feeding duration'/exp OR 'breast feeding duration') AND ('weaning'/exp OR 'weaning' OR 'complementary feeding'/exp OR 'complementary feeding' OR 'early weaning' OR

'early complementary feeding') AND [2016-2021]/py AND ('practice guideline'/exp OR 'practice guideline' OR 'guideline'/exp OR guideline)

UPTODATE https://www.uptodate.com/home

Society Guideline Links: *breastfeeding and infant nutrition*; *celiac disease*

SOCIETY GUIDELINE LINKS: *complementary feeding, weaning, celiac disease, gluten, breast feeding*

National Guideline Clearinghouse (NGC) https://www.ahrq.gov/gam/index.html

Canadians Medical Association (CMA) https://www.cma.ca/clinicalresources/practiceguidelines

National Guideline Centre (NGC) - National Institute of Health and Care Excellence (NICE) https://www.rcplondon.ac.uk/about-us/what-we-do/national-guideline-centre-ngc

Scottish Intercollegiate Guidelines Network (SIGN) https://www.sign.ac.uk/our-guidelines.html

Australian Clinical Practice Guidelines (ACPG) https://www.clinicalguidelines.gov.au/

New Zealand Guidelines Group (NZGG) https://www.health.govt.nz/about-ministry/ministry-health-websites/new-zealand-guidelines-group

American Academy of Pediatrics (AAP) https://www.aap.org/en-us/Pages/Default.aspx
DateRange (01/01/2013-03/19/2019) AND ((complementary feeding) OR (weaning)) AND (Guideline)

North American Society for Pediatric Gastroenterology, Hepatology and Nutrition (NASPGHAN) https://www.naspghan.org/

European Society for Paediatric Gastroenterology Hepatology and Nutrition (ESPGHAN) http://www.espghan.org/

Società Italiana di Nutrizione Umana (SINU) http://www.sinu.it

Società Italiana di Pediatria (SIP) http://www-sip.it/

Società Italiana di Pediatria Preventiva e Sociale (SIPPS) https://www.sipps.it/

Società Italiana di Nutrizione Pediatrica (SINUPE) https://www.sip.it/2017/09/21/sinupe-societa-italiana-di-nutrizione-pediatrica/

Società Italiana di Gastroenterologia Epatologia e Nutrizione Pediatrica (SIGENP) http://www.sigenp.org

Systematic Reviews search

COCHRANE LIBRARY

#1

"celiac disease" in Title Abstract Keyword - with Publication Year from 2011 to 2021, with Cochrane Library publication date Between Jan 2011 and Jun 2021 Cochrane Review matching

PUBMED

#1

((("Weaning"[All Fields]) OR "Infant Nutritional Physiological Phenomena"[MeSH]) OR "complementary feeding"[All Fields]) AND ("Celiac Disease"[Mesh])

EMBASE

#1

('complementary feeding'/exp OR 'weaning'/exp OR weaning) AND ('celiac disease'/exp OR 'celiac disease' OR 'gluten free diet'/exp OR 'gluten free diet' OR 'gluten introduction') AND [2011-2021]/py AND ([cochrane review]/lim OR [systematic review]/lim OR [meta analysis]/lim)

Studies search

PUBMED

#1

("Weaning"[All Fields] OR "Infant Nutritional Physiological Phenomena"[MeSH Terms] OR "complementary feeding"[All Fields]) AND "Celiac Disease"[MeSH Terms]

EMBASE

#1

('complementary feeding'/exp OR 'weaning'/exp OR weaning) AND ('celiac disease'/exp OR 'celiac disease' OR 'gluten free diet'/exp OR 'gluten free diet' OR 'gluten introduction') AND [2015-2021]/py AND ([controlled clinical trial]/lim OR [randomized controlled trial]/lim)

COCHRANE LIBRARY

#1

celiac disease in Title Abstract Keyword - with Publication Year from 2015 to 2021, in Trials
(Word variations have been searched)

Figure a5.1. Guidelines search flow diagram.

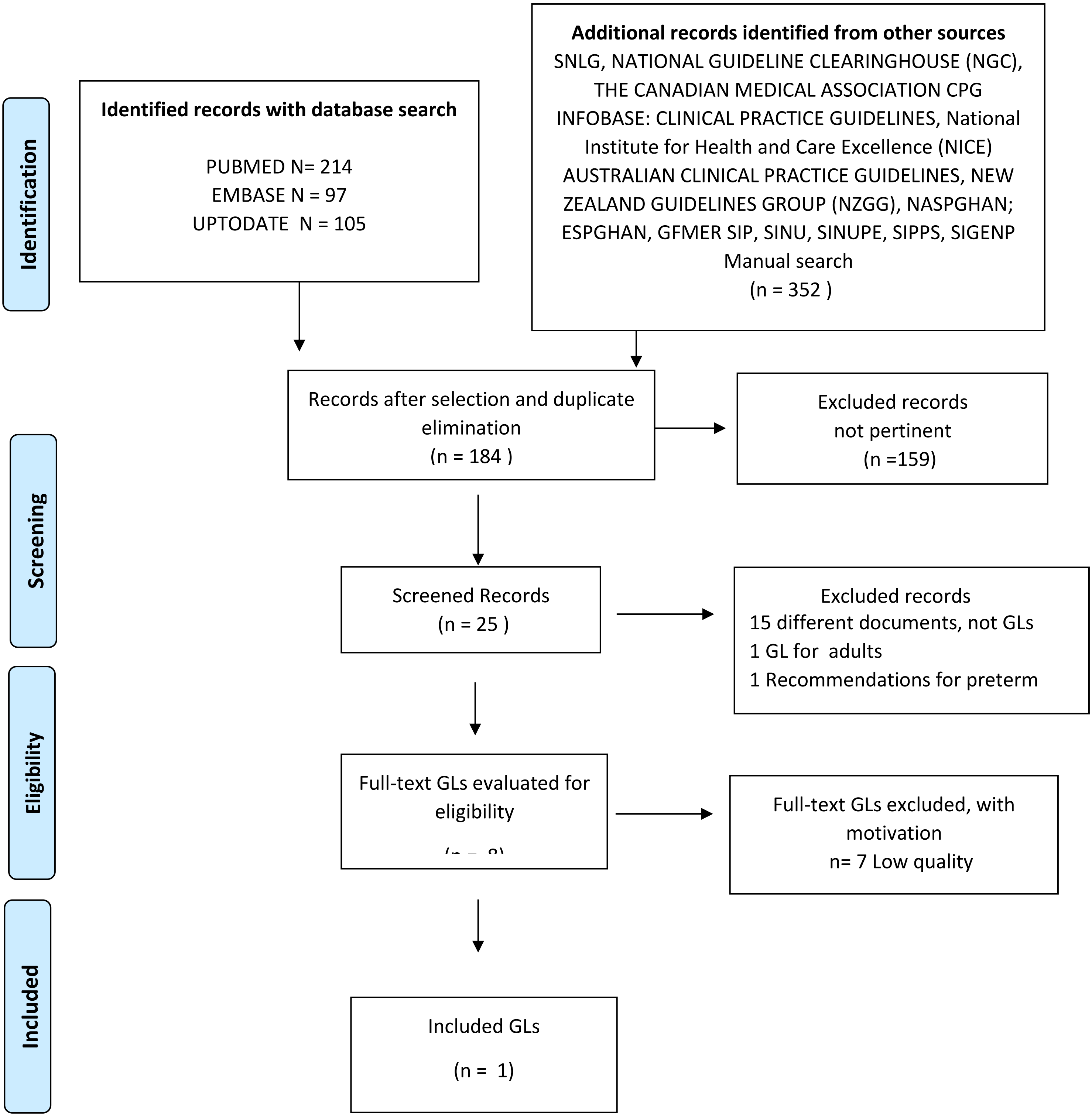

Figure a5.2. SRs search flow diagram.

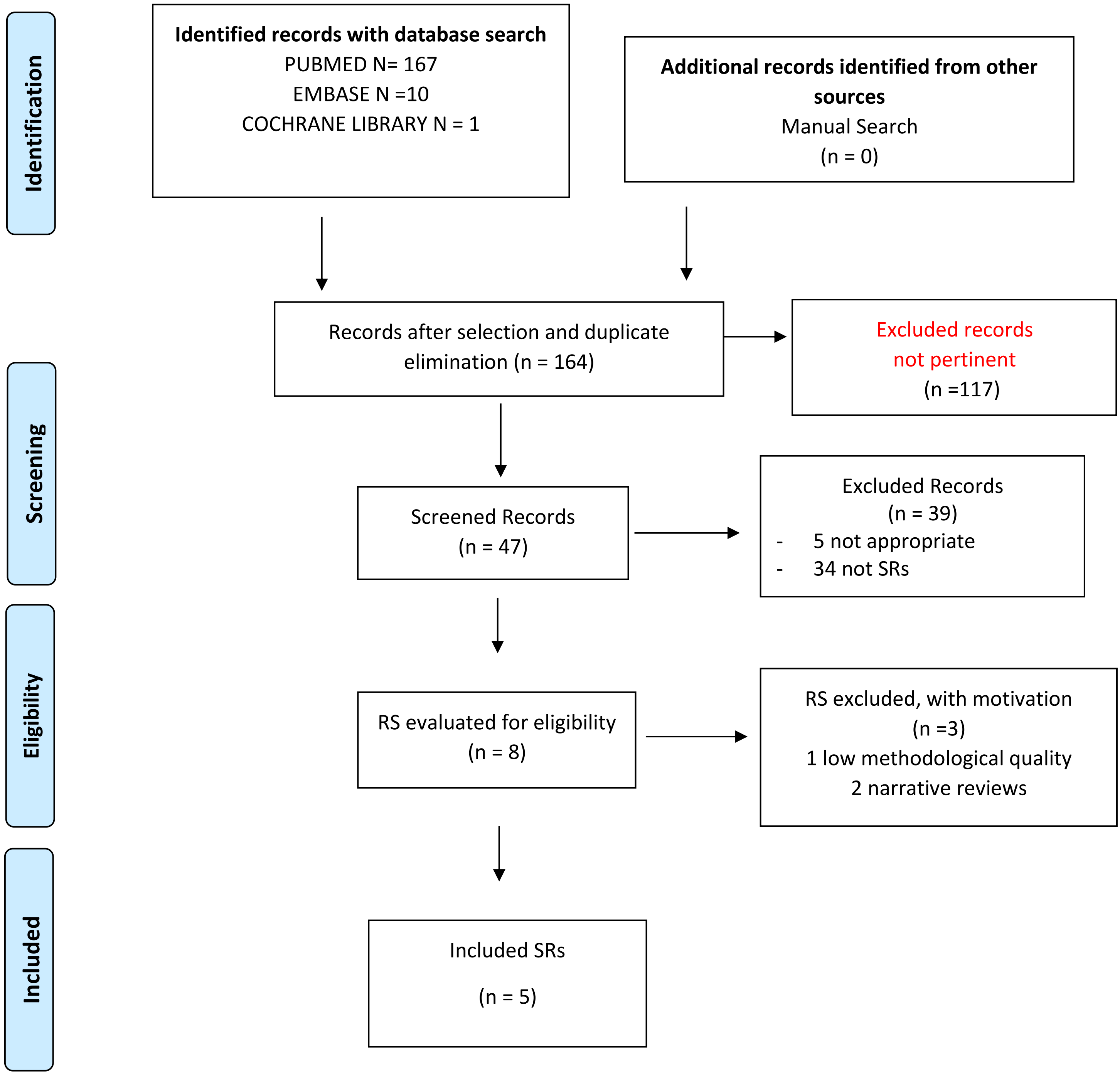

Figure a5.3. Studies search flow diagram.

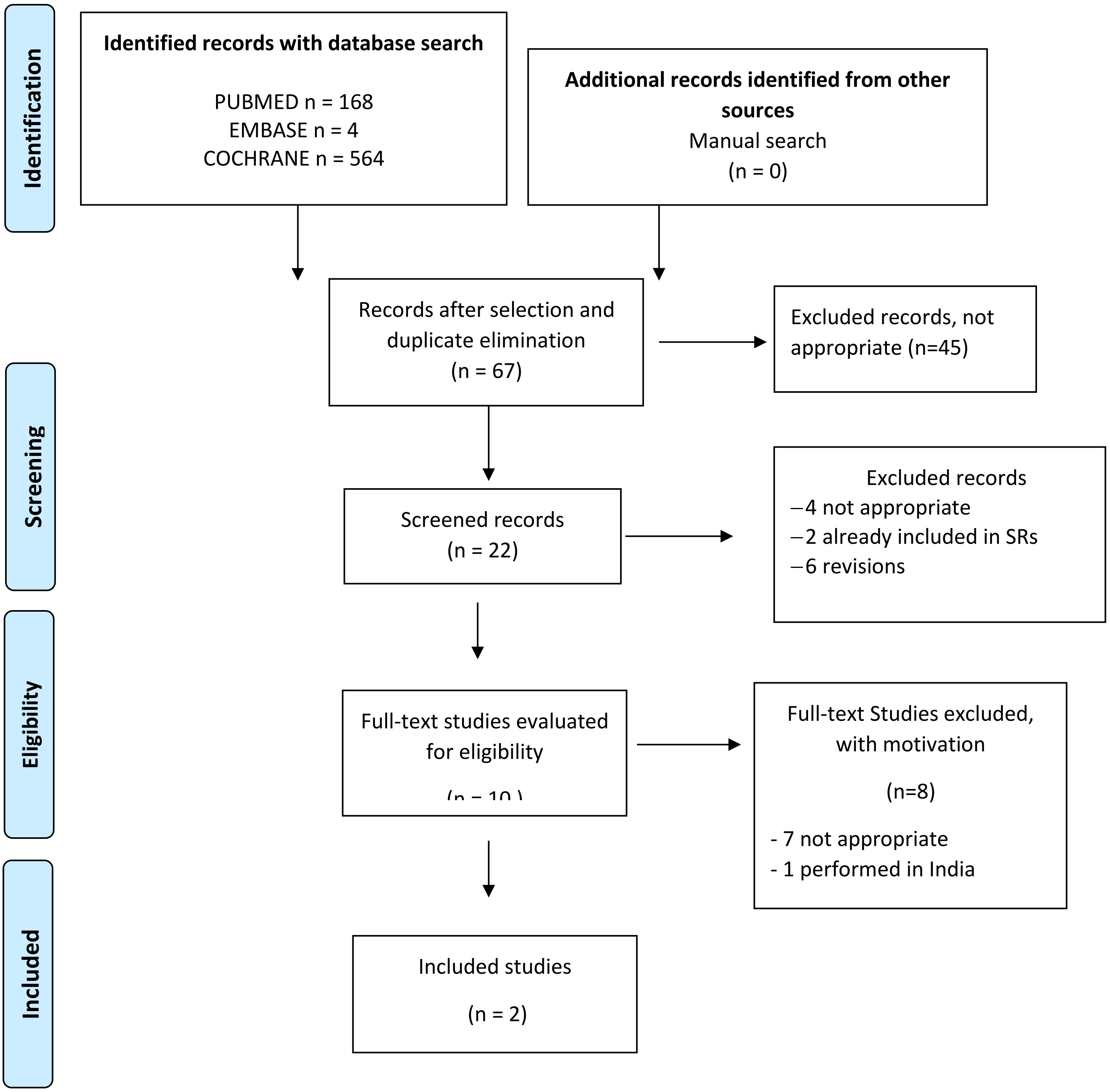

A5. METHODOLOGICAL EVALUATION OF EVIDENCE

Table a5.1. Appraisal of the Guidelines and Clinical Documents

Position Paper	Methodological evaluation			
	**Multidisciplinary panel**	**Systematic evidence search**	**Grading of recommendations**	**LG Overall Rating**
Szajewska et al. ESPGHAN 2016<br>Gluten introduction [1]	YES	YES	YES	Good methodological quality

Table a5.2. Guidelines and Clinical Documents excluded with motivation.

LG and Excluded Documents	Multidisciplinary panel	Systematic evidence search	Grading of recommendations	Reason for exclusion
Alvisi et al. 2015 [2]	Yes	No, not specified, the methodology is not specified	No	Low methodological quality
BAI 2016 WGO [3]	No	No	No	Low methodological quality
Fewtrell et al.. ESPGHAN 2017. Complementary feeding [4]	Yes	Declared in the methods part	No	Low methodological quality
Lebwohl et al. 2016 NASSCD [5]	No	No	No	Low methodological quality
Koninckx Currant 2015<br>Consensus Asociación Española de Pediatría [6]	Yes	No	No	Low methodological quality
Romero Velarde et al. 2016<br>Consenso para las prácticas de alimentación	Not specified	No	No	Low methodological quality

complementaria en lactantes sanos [7]				
Turck et al. 2015<br>Diversification alimentaire : évolution des<br>concepts et recommandations [8]	NO	NO	NO	Low methodological quality

Table a5.3. Appraisal of the Systematic Review

**AMSTAR 2**	Heriksson et al. 2013 [9]	Szajewska et al. 2015 [10]	Silano et al. 2016 [11]	Pinto-Sánchez et al. 2016 [12]	EFSA 2019 [13]
**1. Did the research questions and inclusion criteria for the review include pico components? (Yes No)**	Yes	Yes	Yes	Yes	Yes
**2. Did the SR report contain an explicit statement that the methods of the review had been established before conducting the review and did the report justify any significant deviations from the protocol? (Yes / Partial Yes / No)**	Partial yes	Yes	Partial yes	Yes	Yes
**3. Did the authors of the review justify their selection of study designs to be included in the review? (Yes No)**	Yes	Yes	Yes	Yes	yes
**4. Did the authors of the review use a comprehensive bibliographic research strategy? (Yes / Partial Yes / No)**	Partial yes	Partial yes	Partial yes	Partial yes	Partial yes
**5. Did the authors of the review perform the selection of studies in duplicate? (Yes No)**	No	Yes	Yes	Yes	Yes
**6. Did the authors of the revision perform double data extraction? (Yes No)**	No	Yes	Yes	Yes	Yes
**7. Did the authors of the review provide the list of excluded studies and justify the exclusions? (Yes / Partial Yes / No)**	No	Yes	No	Yes	Yes (?)
**8. Did the authors describe the included studies in sufficient detail? (Yes / Partial Yes / No)**	Partial yes	Yes	Partial yes	Yes	Yes
**9. Did the authors use a satisfactory technique to assess the risk of bias (RoB) in individual studies included in SR? (Yes / Partial Yes / No / Includes NRSI-RCT only)**	Partial yes	Yes	Yes	Yes	Yes
**10. Did the authors report the sources of funding for the studies included in the review? (Yes No)**	No	No	No	No	No
**11. If a meta-analysis was performed, did the authors use appropriate methods for the statistical consolidation of the results? (Yes / No / No meta-analysis conducted)**	Non-conducted meta-analysis	Yes	Non-conducted meta-analysis	Yes	Yes

**12. If the meta-analysis was performed, did the authors evaluate the potential impact of RoB in individual studies on the results of meta-analysis or other evidence summaries? (Yes / No / No meta-analysis conducted)**	Non-conducted meta-analysis	Yes	Non-conducted meta-analysis	Yes	Yes
**13. Did the authors take RoB into account in individual studies when interpreting/discussing the results of the review? (Yes No)**	Yes	Yes	Yes	Yes	Yes
**14. Did the authors provide a satisfactory explanation and discuss any heterogeneity observed in the results of the review? (Yes No)**	Yes	Yes	Yes	Yes	Yes
**15. If they performed a quantitative synthesis, did the authors conduct adequate research of publication biases (bias of small studies) and discuss its likely impact on the results of the review? (Yes / No / No meta-analysis conducted)**	Non-conducted meta-analysis	Yes	Non-conducted meta-analysis	Yes	< 10 studies in the meta-analysis
**16. Did the authors report potential sources of conflict of interest, including any funding received for conducting the review? (Yes No)**	Yes	Yes	Yes	Yes	Yes
**OVERALL RATING**	**MODERATE QUALITY**	**QUALITY**<br>**HIGH**	**MODERATE QUALITY**	**HIGH QUALITY**	**MODERATE/HIGH QUALITY**
***** presence of 1 critical item and 2 failed non-critical items (no. 3, 15, and 16)					

Table a5. 4. SRs excluded with motivation.

EXCLUDED	Reason for exclusion
Chmielewska et al. 2013 [14]	Not declared in the title, methods, or other parts of the text the fact that it is an SR.
Raanan Shamir et al. 2016 [15]	It is not an SR.
Martín-Masot et al. 2020 [16]	It is not an SR.

Table a5.5. Appraisal of the Studies

	Newcastle Quality Assessment Scale<br>CASE-CONTROL STUDIES							
	**Selection**				**Comparability**	**Exhibition**		
**Study**	**The case definition is adequate**	**Representativeness of cases**	**Control selections (community)**	**Defining Controls (no outcome)**	**Comparability of cases and controls based on drawing or analysis**	**Exposure assessment**	**Same exposure for cases and controls**	**Non-response rate**
Simre et al. 2016 [17]<br>(case-control on accident cases)	1	1	1	1	1a	1b	1	0<br>>20% dropout<br>Not described

RCT

Figure a5.4. Risk of bias summary: review authors' judgments about each risk of bias item for each included study. [18]

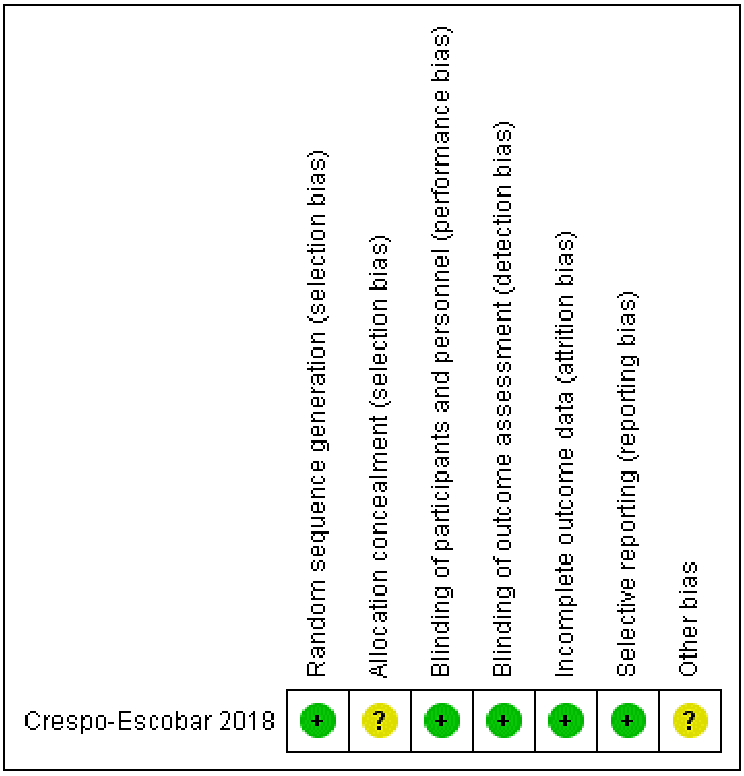

Table a5. 6. Studies excluded with motivation.

**EXCLUDED**	**Reason for exclusion**
Hyytinen et al. 2017 [19]	Not applicable: evaluate PLV-free formulas
Lionetti et al. 2017 [20]	Objective study: prevalence of CD according to the mode of delivery.
Barroso et al. 2018 [21]	Irrelevant. Evaluate dietary patterns from the 1st year of life
Uusitalo et al. 2018 [22]	Irrelevant. Introduction gluten and development of Islet autoimmunity (diabetes)

Hummel et al. 2021 [23]	Irrelevant. Follow up Uusitalo 2018
Vajpayee et al. 2016 [24]	A study conducted in India
Welander et al. 2014 [25]	Irrelevant. The objective of the study: to verify whether the fact that mothers have CDs influences how they feed their children
Hård af Segerstad et al. 2018 [26]	Irrelevant<br><br>Evaluate milk powder intake as a risk factor

A5. RECOMMENDATIONS OF GLs, RESULTS IN SRs AND STUDIES

- ***Can the period of introduction of gluten affect the development of celiac disease?***	**P** In a healthy infant<br>**I** the early (before the 6th month) or delayed (after the 10th-12th month) introduction of gluten<br>**C** compared to the same introduction timing for all foods (6th-7th month)<br>**O** can affect the development of celiac disease?

Table a5.7. Recommendations, Guidelines, and Other Documents

Guidelines	population	Recommendations	Grading
Szajewska et al. ESPGHAN 2016<br>Gluten introduction [1]	healthy infant, children < 3-5 years	**Introducing gluten at 4-6 mo versus > 6 mo of age.**<br>Introducing gluten at 4 to 6 s versus introducing gluten at > 6 mo of age does not reduce the cumulative incidence of CDA or CD in childhood.	An RS identified an RCT applicable to the target population with a similar risk of developing autoimmunity for CD or celiac disease at 3 years in the gluten versus placebo population.<br>**RS** *Szajewska H,2015*<br>**RCT** *Vriezinga SL, 2014*<br>**3 Observational studies** for the development of autoimmunity for Celiac disease*(Hummel S 2007; Norris JM 2005; Aronsson CA 2015)* and **3 Observational studies** *(Aronsson CA 2015; Størdal K 2013)* for the development of celiac disease applicable to the population studied. [100% agreement].
		**Introducing gluten at 6 mo versus 12 mo of age**<br>In children at high risk of CD, introducing gluten at 6 mo versus introducing gluten at 12 mo does not reduce the cumulative incidence of CDA or CD, but leads to an earlier manifestation of CD	**1 RCT:** Lionetti E 2014.<br>**1 RS:** Szajewska H 2015.<br>**[97%** agreement**].**
		**Introducing gluten at <3-4 mo versus** 4-6 **mo of age**<br>It is unclear whether introducing gluten at <3-4 mo versus introducing gluten at 4-6 mo of age affects the risk of developing CDA or CD.	**3 Observational studies** (*Aronsson CA 2015;<br>Størdal K, 2013; Welander A, 2010*) :<br>no difference in CD risk for infants exposed to gluten at 3-4 mo vs 6 mo

		**Introducing gluten at <3-4 mo versus >6 mo of age**<br><br>It is unclear whether introducing gluten at <3-4 mo versus introducing gluten at> 6 mo of age affects the risk of developing CDA or CD	**4 Observational studies** (Norris JM 2005;<br>*Aronsson CA 2015; Størdal K 2013;*<br>Welander A 2010): no difference in the risk of celiac disease autoimmunity for infants exposed to gluten at 3-4 mo vs 6 mo.<br><br>**Conditional recommendation**<br>**87,5%** agreement
			**3 Observational studies** (*Aronsson CA 2015;*<br>*Størdal K, 2013; Welander A, 2010*): no difference in CD risk for infants exposed to gluten at 3-4 mo vs 6 mo
		**Glutine a <6 mesi rispetto al glutine a> 6 mesi di età.**<br>It is unclear whether introducing gluten at <6mo versus introducing gluten at> 6 mo of age affects the risk of developing CDA or CD<br>.	**4 studi osservazionali** (Norris JM 2005;<br>*Aronsson CA 2015; Størdal K 2013;*<br>Welander A 2010): no difference in the risk of celiac disease autoimmunity for infants exposed to gluten at 3-4 mo vs 6 mo.<br>**Conditional recommendation**<br>**91% agreement**
		**Summary recommendation:** gluten can be introduced into the diet of the child between the ages of 4 and 12 mo completed. The age of introduction of gluten in infants in this age group does not appear to influence the absolute risk of developing CDA or CD in childhood.<br>.	**5 observational studies:**<br>*Greco L 1988;*<br>*Norris JM 2005;*<br>*Aronsson CA 2015;*<br>*Størdal K 2013;*<br>**Raccomandazione condizionale.**<br>**[87,5% agreement]**

Table a5.8. Included SRs: Characteristics, Results, and Conclusions

**Systematic Review**	**Population and purpose of the SR**	**Results**	**Conclusions**
Szajewska et al. 2015 [10]<br><br>*MEDLINE, EMBASE, and the Cochrane Library were searched*<br>*From July 2012 to November 2014, February 2015.*	**Population:** For prospective studies: Infants at risk of developing CD (defined by HLA status and/or first degree relative with CD or diabetes mellitus 1).<br><br>For retrospective studies: children or adults with the biopsy-proven CD or CD positive autoantibodies. (e.g. anti TTG or anti endomysium) indicating CD-related autoimmunity.<br>**Aim:** development of CD or CD-related autoimmunity.	**Timing introduction gluten**<br>Interventional trials:<br>- Vriezinga SL 2014 (PREVENTCD): the introduction of 100 mg of immunologically active gluten at 16-24 weeks leads to a similar risk of CD at the age of 3 years (gluten at 4-6 mo vs gluten> 6 mo and overt CD at 3 years RR 1.21 (0.79-1-84); 3-year autoimmunity CD 0.81 (0.49-1.32).<br>- Lionetti E,2014 (CELIPREV): the introduction of gluten at 6 mo compared to the introduction at 12 mo increases the risk of overt celiac disease and autoimmunity for CD at 2 years of age (for celiac disease RR 2.36 (1.27-4.36, for autoimmunity RR 2.25 (1.34-3.79), but has no risk on autoimmunity (RR 1.06 (0.74-1.52) and overt celiac disease (1.02 (0.76-1.56) at the age of 5.<br>- Sellitto M, 2012: gluten introduction at 6 vs 12 mo and development of autoimmunity for celiac disease at 2 years (RR 2.33 (0.10-53.03) and 3 years of age (RR 2.33 (0.10-53.03): no difference.<br>- Hummel, 2011: gluten introduction at 6 vs 12 mo and autoimmunity for CD at 3 years (RR 1.35 (0.54-3.37)<br>- Beyerlein 2014:: gluten introduction at 6 vs 12 mo and autoimmunity for CD at 13 years RR 1.66 (0.74-1.52).<br>**Observational studies:**<br>- Norris 2005: gluten at < 3 mo and > 7 mo of age in children at risk for CD and diabetes I increases R of CD autoimmunity.<br>- Falth-Magnusson K, 1996, Ivarsson A, 2002, Peters U, 2001, Welander A,2010, Ziegler AG 2003: no relationship between gluten introduction and R of celiac disease<br>- Jansen MA 2014 (Generation R study) the introduction of gluten from 6 mo, compared with an early introduction, was not significantly associated with positive TTGs (CD autoimmunity) (adjusted OR: 0.64, 95% CI: 0.31–1.31).<br>- Størdal K, 2013: introduction of gluten> 6 mo vs <6 mo is associated with an increase in the R of CD, however with borderline results (adjusted OR: 1.27, 95% CI: 1.01–1.65).<br>- Hummel S, 2007 (BABYDIAB): no R of autoimmunity for CD due to the introduction of gluten under or above 3 mo of age<br>- Aronsson CA, 2015 (TEDDY study): gluten introduction <17 weeks, between 17 and 26 weeks,> 17 weeks: no difference in autoimmunity R for CD.<br>- Ivarsson A, 2013 (ETICS study): significant difference in the prevalence of CD in two cohorts of births before and after the epidemic: 1993 (introduction of gluten from 6 mo of age) and 1997 (introduction of gluten in small quantities, from 4-6 mo)	The introduction of gluten in specific periods (4 mo, 6 mo of age, and from 6 to 12 mo) does not affect the development of Celiac disease at 3 and 5 years respectively<br>.

Silano et al. 2016 [11]<br><br>*The search was performed in November 2014 and repeated in December 2014 and in September 2015, following the guidelines of the preferred reporting articles for systematic reviews and meta-analysis group (PRISMA) by MEDLINE, via PubMed (http://www.ncbi.nlm.nih.gov/pubmed) EMBASE and Web of Science*	**Population:** the prospective studies included were to include infants/children with increased risk of developing CD. The risk of developing CD was defined by HLA DQ2/8 positivity and/or at least one first degree relative with CD or type 1 diabetes mellitus (T1DM).<br>For retrospective studies, participants had to be children or adults diagnosed with CD small intestine biopsy or serological positivity (anti-tissue antibody transglutaminase (tTG);<br>Furthermore, to be included in the analysis, the studies had to have assessed the risk of CD in the people with the following features:<br>‣ always breastfed versus those never breastfed<br>‣ breastfeeding for different periods<br>‣ breastfeeding at the time of the first introduction of gluten during weaning compared to those who were not<br>‣ introduction of gluten for the first time during weaning at different mo of age<br>**Primary outcome**: development of autoimmunity associated with CD (anti-tTG antibodies) and/or CD tested for biopsy	**Timing of the introduction of gluten and CD risk:**<br>- Lionetti E, 2014: gluten introduction at 6 vs 12 mo. HR 0.9 (95% CI 0.6 to 1.4) (**no difference in CD at 5 years of age**, in children who introduced gluten at 6 vs 12 mo).<br>- Vriezinga et al, 2014: gluten introduction at 16–24 weeks: HR 1.23 (95% CI 0.79 to 1.91) (**no difference in CD at 3 years of age**)<br>- Jansen 2014: R generation study concludes that there are no differences in the development of autoimmunity for CD whether gluten is introduced before or after 6 mo of age<br>- Aronsson CA 2015 (TEDDY): **neither early (<17 weeks) nor delayed (> 26 weeks) introduction represent a risk factor** for the subsequent development of autoimmunity associated with CD and CD proven by biopsies (<17 weeks: HR 0.59 (95% CI 0.33 to 1.04); 17–26 (reference) >26 weeks: HR 0.90 (95% CI 0.69 to 1.18).<br>- Størdal et al: reported a slightly increased risk for children who received gluten with the onset of CF after 6 mo of age (OR 1.27; 95% CI 1.01 to 1.65), but not for those who introduced it before 4 mo of age<br>- Norris 2005: **increased risk of developing CD-related autoimmunity** in two groups of children who introduced gluten before and after the reference period, respectively (4-7 mo of age). Group> 7 mo: HR 1.87 (95% CI 0.97 to 3.60), group 1-3 mo: HR 5.17 (95% CI 1.44 to 18.57). For these last two papers (Størdal et al and Norris 2005) they report moderate risk indices with possible additional risk factors and a great variability, showing a low statistical significance.	The age of introduction of gluten during the weaning process does not affect the development of CD
Pinto-Sánchez et al. 2016 [12]<br><br>*The search was performed until January 2014*	**Population:** Pediatric population, in particular infants in which the development of celiac disease has been verified in concern to the introduction of gluten. Intervention and control population based on the different areas studied:	**Timing of the introduction of gluten:** 15 included studies<br><br>**<u>6 vs 12 mo:</u>**<br><br>- Sellitto M, 2012 e Hummel S 2011 (RCT) together reported 18 cases of CD in 183 patients. <u>No statistically significant risk</u> for CD development associated with the "standard" introduction of gluten (5-6 mo) vs "delayed" (12 mo) (RR, 1.41; 95% CI, 0.59-3.39). However, it should be noted that: the Hummel study has high RoB (blindness and high rate of non-compliance - 30%) while Sellitto has a high RoB imbalance due to dropout and unclear randomization and allocation concealment.	The results of the meta-analysis support only a moderate increase in risk in the late introduction, but not for the early introduction of gluten, towards the development of CD. (Note: From the discussion of the article it is not clear why the authors make this statement after this difference does not emerge from the analysis they performed, not even from the post hoc analysis.)

	- **Timing of gluten introduction**: the intervention group included any gluten-containing products (e.g. cereals, flour, or other foods containing gluten, preparations produced for research purposes) introduced early (<4 mo) or late (> 7 mo) and the control group included subjects in whom gluten was introduced between 4-6 mo of age.<br>- For the "**gluten dose**" and "**method of introduction**", the intervention group was considered to consist of those who received a large amount of gluten in the control group a standard intake. The modality of gluten introduction was considered: "Gradual" in the intervention group and "Normal" in the control group.<br>- The intervention group breastfed for any period vs never breastfed. An alternative definition: group of nursed vs non-breastfed infants during weaning.<br>**Primary outcome:** development of autoimmunity for CD (TTG or EMA positive) and/or CD verified by biopsy.	**<u><4 mesi vs recommended timing (5-6 mo) or later (> 6 mo):</u>**<br>- 4 cohort studies: Norris JM 2005; Welander A, 2010; Stordal K, 2013; Hummel S, 2007 they compared:<br>○ <u>gluten introduction <4 mo vs> 6 mo.</u> Total population of 50 451 children and 282 events. Result for pooled analysis (early vs late introduction of gluten): no difference for CD risk (RR, 1.08; 95% CI, 0.76-1.54; P = 0.68).<br>○ <u>introduction of gluten <4 mo vs recommended age</u> (5-6 mo): no significant difference (RR, 1.27; 95% CI, 0.86-1.86; P = 0.38).<br>- 1 case control study: Ivarsson A 2002; gluten introduction in 491 children with CD and 781 controls without CD: no difference in the introduction of gluten at 1-4 mo vs 5-12 mo (OR, 0.70; 95% CI, 0.48-1.03; P = 0.07).<br>**<u>4-6 mo (recommended timing) vs> 6 mo:</u>**<br>- 5 cohort studies: (Norris JM 2005; Welander A, 2010; Stordal K, 2013; Hummel S, 2007, Ivarsson A, 2013): a total of 240 patients with CD compared to 534 controls. <u>Each study reported no differences in CD</u>	
EFSA 2019 [13]	**Population:** infants or children, generally healthy in	**Celiac disease and gluten: early vs late introduction.**	Risk for developing celiac disease and autoimmunity for CD not influenced by the age of introduction of gluten.

	the period of the start of complementary feeding, both term, and preterm.<br>**Primary Outcome:** Gluten with the R of celiac disease.	Andren Aronsson et al., 2015: age of the population 1.7 - 8.8 years, gluten <4 mo vs 4-6 mo: HR 0.59 (95% CI: 0.33-1.05); gluten 4-6 mo vs> 6 mo: HR1.11 (0.85-1.44);<br>- Norris et al., 2005: average age of the population 4.8 years; gluten <3 mo vs 4-6 mo: HR 22.87 (4.53-115.46); gluten 4-6 mo and> 6 mo: HR 0.25 (0.07-0.87);<br>- Stordal et al., 2013: population 2-12 years: gluten ≤4 mo vs 5-6 mo: HR 1.05 (0.70-1.59); gluten 5-6 mo vs ≥ 6 mo: 0.79 (0.62-1.00).<br>- Welander et al., 2010: average population 8 years: gluten: 3-4 mo vs 5-6 mo: HR 1.00 (0.30-3.32); Gluten 5-6 mo> 6 mo: 0.91 (0.50-1.68).<br>Meta-analysis of the results of these studies: risk for celiac disease not significant. [HR 0.94 (0.48-1.82)]<br><br>**Celiac disease and gluten: early vs late introduction** (retrospective studies)<br>Auricchio et al., 1983: average age 15 mo; ≤ 2 vs ≥ 2 mo: HR 1.46 (0.83-2.58)<br>- Greco et al., 1983: average age up to 2 years; ≤ 2 vs ≥ 2 mo HR 1.46 (0.93; 2.30)<br>- Ivarsson et al., 2002: average age up to 2 years; ≤4 mo vs 5-6 mo: HR 0.71 [0.43; 1.18]; ≤ 4 mo vs > 6 mo: HR 1.32 [0.72; 2.43]<br>- Peters et al., 2001: average age 6.4 years: ≤3 years vs > 3 years: HR 1.38 [0.56; 3.39].<br><br>Meta-analysis of the results of these studies: risk for celiac disease not significant. [HR 1,20 (0.80; 1.81)].<br><br>**Autoimmunity for celiac disease and gluten: early vs late introduction**<br>- Andren Aronsson et al., 2015: age of the population 1.7 - 8.8 years, gluten <4 mo vs 4-6 mo: HR 1.06 [0.79; 1.42]; gluten 4-6 mo vs> 6 mo: HR1.03 [0.87; 1.22];<br>- Chmiel et al., 2015 age up to 16 years, gluten < 3 mo; ≥ 3 mo: HR 1.26 [0.17; 9.29];<br>- Jansen et al., 2014: average age 6 years, gluten ≤6 mo vs ≥ 6 mo: HR 1.56 [0.76; 3.22];<br>- Norris et al., 2005; average age of the population 4.8 years; gluten < 3 mo vs 4-6 mo: HR 5.17 [1.44; 18.57]; gluten 4-6 mo and > 6 mo: HR 0.53 [0.28; 1.02];<br><br>Meta-analysis of the results of these studies: risk for Autoimmunity for CD not significant (HR 1.09 [0.63; 1.87].	

Table a5.9. Included studies: Characteristics and Results.

Study	Study design	Population	Test	Primary outcome	Secondary outcome	Follow-up	Results
Simre et al. 2016 [17]	Case-control on incident cases	Original at-risk cohorts (HLA pos.): 258 Estonian and 305 Finnish children, followed **from birth at 3 years (BC)** + 1363 (81%) and 1384 (88%) children followed from 3 to 5 years (YCC) From these 29 children developed CD compared with 29 control children without CD, selected for haplotype, age, and residence	Comparison by age of introduction of gluten, breastfeeding, and infections from 6 to 36 mo	Compare the cumulative incidence of CD between Estonian and Finnish children up to 5 years and identify the factors that may be involved in modulating the incidence, paying particular attention to early feeding and infections<br>.		3 years	**In BC**: the age at which wheat, barley, or rye were introduced was not statistically different between the CD and the control groups. The mean age at which complimentary food was introduced was similar in both groups (4.4 mo). No information was collected in the YCC on age at introduction of cereals or complementary foods
Crespo-Escobar et al. 2018 [18]	Double-blind RCTs versus placebo	225 children at risk of celiac disease, Spanish cohort of the PREVENT CD study [Vriezinga 2014]	Comparison by age of introduction of gluten, 4-6 mo (early, n = 116) vs> 6 mo (7-12, late, n = 109)	Incidence of CD at 10 years	Effect of the *gluten intake pattern* in the first 3 years of life on CD development	10 years	N ° cases CD entire cohort= 26/225<br>N° cases CD early introduction 16/116<br>N° cases CD late introduzction 10/109<br><br>Gluten 1.0 (reference)<br>HR Placebo 0.9 (0.72-1.82) p=0.66

- ***The development of celiac disease is influenced by the CF/mode of breastfeeding?***	**P** In a healthy infant<br>**I** the introduction of gluten associated with the intake of breast milk<br>**C** compared to the introduction of gluten associated with the intake of formula<br>**O** can affect the development of celiac disease?

Table a5.10. Recommendations, Guidelines, and Other Documents

Guidelines	Population	Recommendations	Grading
Szajewska et al. ESPGHAN 2016<br>Gluten introduction [1]	healthy infant, children < 3-5 years	**Breastfeeding (BF) versus no breastfeeding.**<br>BF compared to no BF has not been shown to reduce the risk of developing CD in childhood	**7 observational studies:**<br>*Vriezinga SL, 2014;*<br>*Lionetti E 2014;*<br>*Greco L, 1988*<br>*Hummel S 2007;*<br>*Roberts SE, 2009;*<br>*Auricchio S, 1983;*<br>*Decker E, 2010;*<br>High heterogeneity between studies,<br>Low quality of evidence
		**Breastfeeding when gluten is introduced.**<br>BF upon introduction of gluten, compared to the introduction of gluten upon cessation of BF, has not been shown to reduce the risk of developing CD in childhood	**2 randomized interventional trials:**<br>*Vriezinga SL, 2014;*<br>*Lionetti E 2014;*<br>Note: the two studies were not specifically designed to evaluate the effect of BF on CD development.<br>Note: low quality of evidence
		**Recommendation**<br>**The introduction of gluten during breastfeeding cannot be recommended as a means of reducing the risk of developing CD**<br>**BF recommendations should not be changed due to CD prevention considerations**	Conditional recommendation; low quality of evidence [100% agreement]<br>Conditional recommendation; low quality of evidence. [97% Agreement]

Table a5.11. Included SRs: Characteristics, Results, and Conclusions

Systematic Review	Population and purpose of the SR	Results	Conclusions
Heriksson et al. 2013 [9]<br>*Previous research: observational studies 1966-2004. Update 2004-2011. Pubmed, EMBASE E Cinahl*	**Population:** children with CD, variable age 14 mo-8.4 years breastfed or not, with different duration of breastfeeding. CD diagnosis made with a biopsy, exposure data collected with a questionnaire or parental interview.<br><br>**Purpose:** comparing the risk of CD in breastfed infants with risk in those who were not breastfed or comparing the risk of CD by the duration of breastfeeding	4 observational studies *(Norris 2005, Román 2010, Radlovic 2010, D'Amico 2005).* No studies have compared breastfed infants to formula-fed infants<br>.<br><br>**Breastfeeding duration and CD risk**<br><br>2 (Radlovic 2010, D'Amico 2005) of the 3 studies that examined the duration of breastfeeding and CD reported significant associations between the longer duration of breastfeeding and the subsequent onset of CD<br><br>*Norris 2005* – no association associazione<br><br>**Breastfeeding during the introduction of gluten**<br><br>2 (Román 2010, Radlovic 2010) of 3 included studies reported that breastfeeding during the introduction of gluten significantly delayed the onset of CD.<br>*Norris 2005* does not report statistically significant differences<br>*D'Amico 2005* does not report results for this outcome	Breastfeeding appears to offer protection against the development of CD in predisposed infants.<br>Breastfeeding at the time of gluten introduction and total duration of breastfeeding appear to be the two most significant variables in reducing the risk
Silano et al. 2016 [11]<br>*The search was performed in November 2014, and repeated in December 2014 and in September 2015, following the guidelines of the preferred reporting articles for systematic reviews and meta-analysis group (PRISMA) by MEDLINE, via PubMed (http://www.ncbi.nlm.nih.gov /pubmed) EMBASE and Web of Science*	**Popolazione:** the prospective studies included were to include infants/children with increased risk of developing CD. The risk of developing CD was defined by HLA DQ2/8 positivity and/or at least one first degree relative with CD or type 1 diabetes mellitus (T1DM).<br>For retrospective studies, participants had to be children or adults diagnosed with CD small intestine biopsy or serological positivity (anti-tissue antibody transglutaminase (tTG);<br>Furthermore, to be included in the analysis, the studies had to have assessed the risk of CD in the people with the following features:<br>‣ always breastfed versus those never breastfed<br>‣ breastfeeding for different periods<br>‣ breastfeeding at the time of the first introduction of gluten during weaning compared to those who were not<br>‣ introduction of gluten for the first time during weaning at different mo of age	**Breastfeeding and CD risk**<br><br>10 of the 16 articles that studied the effect of breastfeeding and the risk of CD concluded that **the duration of breastfeeding did not show a preventive effect on the development of CD** (*Lionetti E, 2014; Vriezinga SL,2014; Ziegler AG,2003; Norris JM,2005; Aronsson CA, 2015; Welander A,2010; Peters U, 2001; Decker E, 2010; Roberts SE, 2009; Jansen MA, 2014)*<br><br>*Størdal K, 2013:* positive correlation between prolonged breastfeeding for beyond the first year of age and increased incidence of CD<br><br>*Auricchio S,1983; Greco L,1988; Fälth-Magnusson K,1996; Peters U,2001:* protective effect of breastfeeding on the development of CD<br>**Breastfeeding when gluten is introduced**<br>Protective effect (retrospective studies):<br>- Fälth-Magnusson, 1996: patients with CD (biopsy proven)=72; controls=264. Result: breastfeeding >2.5 mo is protective for CD ($p<0.0002$)<br>- Peters, 2001: patients with CD (biopsy proven)=143; controls=137: breastfeeding >2 mo is protective for CD. OR 0.37 (95% CI 0.21 to 0.64)	The data from the included studies report some a protective effect, others no effect. Studies reporting **no effect on CD development** are the most recent, have the highest GRADE, and lowest risk of bias.<br><br>Therefore, all the prospective studies included, except one, conclude that the duration of breastfeeding (exclusive or complementary) and/or the introduction of gluten while the baby is still breastfed have **no impact on the development of CD**.

	**Aim:** Primary outcome development of autoimmunity associated with CD (anti-tTG antibodies) and/or CD tested for biopsy	Non-protective effect (reported in 5 prospective studies):<br>- Lionetti E, 2014: 832 patients (117 develop autoimmunity for CD, including 86 biopsy-proven celiac disease). **Effect of breastfeeding: none**. Autoimmunity for CD: OR=1.0 (95% CI 0.9 to 1.0) Celiac Disease: OR=1 (95% CI 0.9 to 1.1).<br>- Vriezinga et al, 2014: 944 patients. Development of CD at 0 month OR 0.90 (95% CI 0.22 to 3.6) <3 mo OR 1.3 (95% CI 0.41 to 4.1) 4–5 mo OR 1.5 (95% CI 0.57 to 4.1)<br>- Norris 2005: 1560 (51 develop autoimmunity for CD); **Effect of breast milk during the introduction of gluten: none**. OR=1.32 (95%, CI 0.76 to 2.28)<br>- Aronsson CA 2015: 6434 (773 develop autoimmunity for CD, 307 CD proven by biopsies). **Effect of breast milk during the introduction of gluten: none.** Overt CD: OR=1.13 (95% CI 0.88 to 1.46).<br>- Ivarsson A 2002: CD proven by biopsies =627, controls=1254; **Protective effect of brestfeeding.** OR 0.55 (95% CI 0.4 to 0.77).<br>- Størdal K 2013: CD proven by biopsies =324; controls=81	
Pinto-Sánchez et al. 2016 [12]<br><br>*Search until January 2014*	**Population:** Pediatric population, in particular infants in which the development of celiac disease has been verified with the introduction of gluten.<br>Intervention and control population based on the different areas studied:<br>- **Timing of gluten introduction**: the intervention group included any gluten-containing products (e.g. cereals, flour, or other foods containing gluten, preparations produced for research purposes) introduced early (<4 mo) or late (> 7 mo) and the control group included subjects in whom gluten was introduced between 4-6 mo of age.<br>- For the "**gluten dose**" and "**method of introduction**", the intervention group was considered to consist of those who received a large amount of gluten in the control group a standard intake. The modality of gluten introduction was considered: "Gradual" in the intervention group and "Normal" in the control group.<br>- The intervention group breastfed for any period vs never breastfed. An alternative definition: group of nursed vs non-breastfed infants during weaning.<br>**Aim**: Primary outcome: development of autoimmunity for CD (TTG or EMA positive) and/or CD verified by biopsy.	**Breastfeeding at the time of the introduction of gluten and risk of CD**<br>3 cohort studies (impossible to perform meta-analyses for different outcomes between studies):<br>- Stordal 2013: increased R of CD in nursing infants> 12 mo vs nursing infants <6 mo (OR, 1.49; 95% CI, 1.01-2.21; P = 0.04); No difference between nursing infants> 1 month and <1 month during weaning (RR, 1.04; 95% CI, 0.66-1.03).<br>- Norris 2005: no difference in CD between 1560 breastfed during the introduction of gluten and not breastfed (RR, 1.23; OR, 0.72-2.11)<br>- Ivarsson A, 2002: compare the duration of the breastfeeding period in a population born in 1993 vs 1997, finding that in the population born in 1997 who had been breastfed for a longer period there was a lower risk of CD than in the 1993 population: reduced risk of CD with longer duration of breastfeeding (protective BM).<br>5 studies evaluated infants who were breastfed vs never breastfed (or for <1 month): a total of 172,011 participants, including 851 infants with CD (Auricchio S, 1983; Greco L, 1988; Challacombe DN, 1997; Decker E, 2010; Roberts SE, 2009. Overall: 433 of 851 infants with CD (51%) were breastfed, compared with 119,034 of 171,160 controls (70%). The meta-analysis showed a nonsignificant trend towards a lower proportion of breastfed infants in the CD group (OR, 0.55; 95% CI, 0.28-1.10; P = 0.09).<br>6 studies with a total of 48,845 participants, including 926 infants with CD, assessed whether they were breastfed at weaning (Norris JM 2005, Ivarsson A 2002; Stordal K, 2013; Peters U, 2001; Falth-Magnusson K, 1996; Ascher H 1997). Overall: 479 of 926 patients with CD (52%) were breastfed during weaning, compared with 40,789 of 47,919 controls (85%). (OR, 0.70; 95% CI, 0.45-1.10; P = 0.12)	Trend not significant for a potential benefit of breastfeeding towards the development of CD<br>.

Szajewska et al. 2015 [10]*Research based on MEDLINE,*<br>*EMBASE and Cochrane Library*<br>*From July 2012 to November 2014,*<br>*and again in February 2015.*	**Population:** For prospective studies: Infants at risk of developing CD (defined by HLA status and/or first degree relative with CD or diabetes mellitus 1).<br><br>For retrospective studies: children or adults with biopsy-proven CD or CD positive autoantibodies. (e.g. anti TTG or anti endomysium) indicating CD-related autoimmunity.<br>**Aim:** development of CD or CD-related autoimmunity.	**Breastfeeding and CD risk**<br>Interventional trials:<br>- Vriezinga SL 2014 (PREVENTCD): Exclusive breastfeeding, or any type of breastfeeding, does not significantly affect the risk of developing CD<br>- Lionetti E,2014 (CELIPREV): the duration of breastfeeding is similar for children who develop CD and for those who do not<br><br>Observational studies:<br>- Størdal K, 2013 (a prospective study on a cohort of 107,000 births, information on nutrition up to 6 and 18 mo of age, main outcome: development of CD) shows that breastfeeding> 12 mo was associated with a <u>modest increase in the risk of CD</u>.<br>- Jansen MA, 2014 (Generation R study: a prospective cohort study, 1679 Dutch children positive for HLA-DQ2 / DQ8. Outcome: timing of gluten introduction and breastfeeding influence CD<u>) breastfeeding> 6 mo does not reduce the risk of autoimmunity for CD</u> in children aged 6 years<br>- Hummel S, 2007 BABYDIAB (a prospective cohort study. 1511 children, followed up to the age of 7.6 years. Outcome: the natural history of pancreatic islet autoimmunity and CD autoimmunity): <u>No association between breastfeeding duration and R of CD</u><br>- Aronsson CA 2015, TEDDY STUDY: assessed breastfeeding duration, <u>it was not related to the development of CD</u><br>- Ivarsson A, 2013 ETICS study (screening of 13,000 children born in 1993 and 1997. Outcome: duration of breastfeeding, age of introduction of gluten in the diet): compares the duration of breastfeeding, age of introduction of gluten, amount of gluten introduced, and breastfeeding during the introduction in two cohorts of children born in 1993 and 1997. Conclusion: the introduction of small amounts of gluten during breastfeeding influences the development of celiac disease at least up to the age of 12 (<u>protective effect</u>).<br><br>**Pooled results of these observational studies:** any duration of breastfeeding compared to non-breastfeeding does not affect the development of CD. (OR: 0.69, 95% CI: 0.30–1.59).<br><br>**Breastfeeding at the time of the introduction of gluten and risk of CD**<br>Interventional trials:<br>- -Vriezinga SL 2014 (PREVENTCD): breastfeeding during the introduction of gluten does not significantly influence the development of CD.<br>- Lionetti E, 2014 (CELIPREV): no protective effect due to the introduction of gluten during breastfeeding<br>Observational studies:<br>- -Størdal K, 2013 (prospective study on a cohort of 107,000 births, information on nutrition up to 6 and 18 mo of age, main outcome: development of CD) does not support a protective effect of breastfeeding at the time of the introduction of gluten on the risk of CD.	There is no evidence to support that the duration of breastfeeding or the continuation of lactation during the introduction of gluten affects the risk of celiac disease.

		- Aronsson CA 2015, TEDDY STUDY: no difference in the development of autoimmunity for CD or Celiac Disease regardless of the long or short term duration (> 1 vs. ≤1 month) of breastfeeding after the introduction of gluten or discontinuation of ' breastfeeding before the introduction of gluten<br>- Norris, 2005 DAISY study (N=1560 (51 with disease CDA): HR 1.32 (0.76 to 2.28).<br>- Hummel 2007 (BABYDIAB) (N=1511 (N=63 CDA): not significant (data not reported).<br><br>**Pooled analysis:** Breastfeeding at the time of gluten introduction has no risk for the development of CD compared to breastfeeding with formula (OR: 0.88, 95% CI: 0.52–1.51).	
EFSA 2019 [13]	Evaluation of data on celiac disease in subjects born at term or in mixed populations	**Breastfeeding and CD risk**<br>Interventional trials:<br><br>- Vriezinga SL 2014 (PREVENTCD): Exclusive breastfeeding, or any type of breastfeeding, does not significantly affect the risk of developing CD.<br>RR = 1.31 (95% CI = 0.77 - 2.23)<br><br>Observational studies:<br><br>Størdal K, 2013 (prospective study on a cohort of 107,000 births, information on feeding up to 6 and 18 mo of age, main outcome: development of CD) data on 45,156: no association was found between continued breastfeeding at the time of introduction of gluten ≤ 6 mo of age and the risk of developing celiac disease. Conversely, breastfeeding> 12 mo was associated with a modest increase in the risk of CD.	The duration of breastfeeding or the continuation of breastfeeding during the introduction of gluten does not affect the risk of celiac disease

Table a5.12. Included studies: Characteristics and Results.

Study	Study design	Population	Test	Primary outcome	Secondary outcome	Follow-up	Results
Simre et al. 2016 [17]	Case-control on incident cases	Original at-risk cohorts (HLA pos.):<br>258 Estonian and 305 Finnish children, followed from birth at 3 years (BC) + 1363 (81%) and 1384 (88%) children followed from 3 to 5 years (YCC)<br>From these 29 children developed CD compared with 29 control children without CD, selected for	Comparison by age of introduction of gluten, breastfeeding, and infections from 6 to 36 mo	Compare the cumulative incidence of CD between Estonian and Finnish children up to 5 years and identify the factors that may be involved in modulating the incidence, paying particular attention to early feeding and infections<br>.		3 years	There was no statistical difference in the total duration of breastfeeding between infants with CD and control infants in BC or YCC.<br><br>Nor was there any significant difference in the duration of exclusive breastfeeding between the groups in the BC<br>.

		haplotype, age, and residence					

A5. EVIDENCE PROFILE GRADE

Table a5.13. Age of Gluten introduction **4-6 mo vs. ≥6 months**

[Gluten introduction at 4-6 months] versus **[**Gluten introduction up to 6 months] to [prevent the development of Celiac Disease]

Patient or population: [to prevent the development of Celiac Disease]

Setting: Outpatient

Intervention: [Gluten introduction at 4-6 months].

Comparator: [Gluten introduction up to 6 months]

Certainty assessment							No of patients		Effect		Certainty	Importance
N of studies	Study Design	Distortion risk	Lack of reproducibility of results	Lack of generalisability	Inaccuracy	Further considerations	[Gluten introduction at 4-6 months]	[Gluten introduction up to 6 months]	Relative (95% CI)	Absolute (95% CI)		
Development of celiac disease (**Celiac Disease - CD) (follow up: 3 years; evaluation: n°** of at-risk children developing **CD)**												
1 [1]	randomized trials	Not relevant	Not relevant	Not relevant	Not relevant	none	44/475 (9.3%)	36/469 (7.7%)	**HR 1.23** (0.79 a 1.91)	**17 more for 1000** (from 16 minus to 65 plus)	⊕⊕⊕⊕ HIGHT	CRITICAL
1[2]	Observational studies	serious [a]	Not relevant	Not relevant	Not relevant	all plausible residual confounding would reduce the demonstrated effect	There was no statistical difference in the total duration of breastfeeding between infants with CD and control infants in BC or YCC.				⊕⊕◯◯ Low	CRITICAL
Development of **CD (follow up: 10 years; evaluated by n°** of at-risk children developing **CD**												
1 [3]	randomized trials	Not relevant	Not relevant	Not relevant	Not relevant	none	16/116 (13.8%)	10/109 (9.2%)	**HR 0.90** (0.72 a 1.82)	**9 minus for 1.000** (from 25 minus to 69 plus)	⊕⊕⊕⊕ HIGHT	CRITICAL

CI: Confidence interval; **HR:** Hazard Ratio

Explanations

a. wide 95% CI

References

1. 2014, Vriezinga
2. 2016, Simre
3. 2018, Crespo-Escobar (coorte,spagnola,PREVENT CD study)

Table a5.14. Age of Gluten introduction **6 months vs. 12 months**

[Gluten introduction at 6 months] versus [Gluten introduction at 12 months] to prevent the development of Celiac Disease]

Patient or population: [to prevent the development of Celiac Disease]

Setting: Outpatient

Intervention: [Gluten introduction at 6 months].

Comparator: [Gluten introduction at 12 months]

Certainty assessment							№ of patients		Effect		Certainty	Importance
**N of studies**	**Study Design**	**Distortion risk**	**Lack of reproducibility of results**	**Lack of generalisability**	**Inaccuracy**	**Further considerations**	[Gluten introduction at 6 months]	[Gluten introduction at 12 months]	**Relative (95% CI)**	**Absolute (95% CI)**		
Development of **CD (follow up: 3 years; evaluated by: n°** of events)												
2 [1,2]	randomized trials	Not relevant	Not relevant	Not relevant	serious [a]	none	11/94 (11.7%)	7/86 (8.1%)	**RR 1.43** (0.60 a 3.41)	**35 plus for 1.000** (from 33 minus to 196 plus)	⊕⊕⊕◯ MODERATE	CRITICAL
Development of **CD (follow up: 5 years; evaluated by: n°** of events)												
1 [3]	randomized trials	Not relevant	serious [b]	Not relevant	Not relevant	none	50/236 (21.2%)	53/215 (24.7%)	**RR 1.06** (0.74 a 1.52)	**15 plus per 1.000** (from 64 minus to 128 plus)	⊕⊕⊕◯ MODERATE	CRITICAL
Development of **CD – observational studies (evaluated by: n° of events)**												
3 [4,5,6]	Observational studies	serious [c,d]	Not relevant	Not relevant	serious [a]	none	305/45163 (0.7%)	326/45153 (0.7%)	**OR 1.14** (0.75 a 1.75)	**1 plus per 1.000** (from 2 minus to 5 plus)	⊕◯◯◯ VERY LOW	CRITICAL

CI: Confidence interval; **RR:** Risk ratio; **OR:** Odds ratio

Explanations

a. wide 95% CI

b. only one study

c. non-concordant results

d. High heterogeneity (82%)

References

1. 2012, Sellitto. .
2. 2011, Hummel. .
3. 2014, Lionetti. .
4. 2014, Aronsson. .
5. 2013, Størdal. .
6. 2010, Welander. .

Table a5.15. Age of Gluten introduction **<4 months vs ≥ 6 months (7-12 months**

[Gluten introduction at < 4 months] versus [gluten introduction at ≥ 6 months (7-12 months)] to [prevent celiac disease]

Patient or population: [to prevent the development of Celiac Disease]

Setting: Outpatient

Intervention: [**Gluten introduction at < 4 months**]

Comparator: [**gluten introduction at ≥ 6 months (7-12 months)**])]

Certainty assessment							№ of patients		Effetto		Certainty	Importance
N of studies	Study Design	Distortion risk	Lack of reproducibility of results	Lack of generalisability	Inaccuracy	Further considerations	[gluten introduction at < 4 months]	[gluten introduction at ≥ 6 months (7-12 months)]	Relative (95% CI)	Absolute (95% CI)		
Development of **CD evaluated by: n° of events**												
3 [1,2,3]	Observational studies	Not relevant	Not relevant	Not relevant	serious [a]	none	44/7681 (0.6%)	326/45153 (0.7%)	**OR 0.94** (0.69 a 1.30)	**0 minus per 1.000** (from 2 minus to 2 plus)	⨁◯◯◯ VERY LOW	CRITICAL

CI: Confidence interval; **OR:** Odds ratio

Explanations

a. wide 95% CI

References

1. 2010, Welander.
2. 2013, Stordal.
3. 2014, Aronsson.

Table a5.16. exclusive breastfeeding vs no breastfeeding

[exclusive breastfeeding vs no breastfeeding] to [prevent celiac disease]

Patient or population: [to prevent the development of Celiac Disease]

Setting: Outpatient

Intervention: [**exclusive breastfeeding**]

Comparator: [**no breastfeeding**]

Certainty assessment							№ of patients		Effect		Certainty	Importance
N of studies	Study Design	Distortion risk	Lack of reproducibility of results	Lack of generalisability	Inaccuracy	Further considerations	[exclusive breastfeeding]	[no breastfeeding]	Relative (95% CI)	Absolute (95% CI)		
Development of **CD (evaluated by: n° of cases)**												
1 [1]	randomized trials	serious [a]	Not relevant	Not relevant	serious [b]	none	4/30 (13.3%)	4/31 (12.9%)	**RR 1.03** (0.28 a 3.76)	**4 plus per 1.000** (from 93 minus to 356 plus)	⊕⊕○○ LOW	CRITICAL
Development of **CD – prospective studies and case-control**												
5 [2,3,4,5,6]	Observational studies	serious [c]	Not relevant	not relevant	serious [b]	none	0 cases 0 controls		**OR 0.69** (0.30 a 1.59)	-	⊕○○○ VERY LOW	CRITICAL
							-	0.0%		**0 minus per 1.000** (from 0 minus to 0 minus)		

CI: Confidence interval; **RR:** Risk ratio; **OR:** Odds ratio

Explanations

a. risk of bias
b. wide 95% CI
c. High heterogeneity I2= 93%

References

1. 2014, Vriezinga. .
2. 1988, Greco. .
3. 2007, Hummel. .
4. 2009, Roberts. .
5. 1983, Auricchio. .
6. 2010, Decker. .

Table a5.17. gluten introduction during breastfeeding

[gluten introduction during breastfeeding] versus [gluten introduction without breastfeeding] to [prevent celiac disease]

Patient or population: [to prevent the development of Celiac Disease]

Setting: Outpatient

Intervention: [**gluten introduction during breastfeeding**]

Comparator: [**gluten introduction without breastfeeding**]

Certainty assessment							№ of patients		Effect		Certainty	Importance
N of studies	Study Design	Distortion risk	Lack of reproducibility of results	Lack of generalisability	Inaccuracy	Further considerations	[gluten introduction during breastfeeding]	[gluten introduction without breastfeeding]	Relative (95% CI)	Absolute (95% CI)		
Development of **CD – observational studies (evaluated by: n° of cases)**												
8 [1,2,3,4,5,6,7,8]	Observational studies	serious [a]	Not relevant	Not relevant	serious [b]	all plausible residual confounders could reduce the demonstrated effect	28428 cases 21645 controls 535/1500 exposed 27893/48573 not exposed		**OR 0.88** (0.52 a 1.51)	-	⊕◯◯◯ VERY LOW	CRITICAL
							-	0.0%		**0 minus per 1.000** (from 0 minus to 0 minus)		
Development of **CD - RCT (evaluated by: n° of cases)**												
1 [9]	Randomized trials	serious [c]	Not relevant	Not relevant	Not relevant	none	33/339 (9.7%)	20/269 (7.4%)	**RR 1.31** (0.77 a 2.23)	**23 plus per 1.000** (from 17 minus to 91 plus)	⊕⊕⊕◯ MODERATE	CRITICAL

CI: Confidence interval; **OR:** Odds ratio; **RR:** Risk ratio

Explanations

a. Inconsistency of results, especially between cohort and case-control studies
b. wide 95% CI
c. Study not designed for this intervention

References

1. 2001, Peters. .

2. 2005, Norris. .
3. 1997, Ascher. .
4. 1996, Falth-,Magnusson. .
5. 2013, Ivarsson. .
6. 2013, Stordal. .
7. 2014, Aronsson. .
8. 2014, Lionetti. .
9. 2014, Vriezinga. .

Table a5.18. Duration of breastfeeding

[longer duration of breastfeeding] versus [shorter Duration of breastfeeding]] to [prevent celiac disease]

Patient or population: [to prevent the development of Celiac Disease]

Setting:

Intervention: [**longer duration of breastfeeding**]

Comparator: [shorter **Duration of breastfeeding**]

Certainty assessment							№ of patients		Effect		Certainty	Importance
N of studies	Study Design	Distortion risk	Lack of reproducibility of results	Lack of generalisability	Inaccuracy	Further considerations	[longer duration of breastfeeding]	[shorter Duration of breastfeeding]	Relative (95% CI)	Absolute (95% CI)		
Development of **CD (evaluated by: n° of cases)**												
2 [1,2]	Randomized trials	serious [a]	Not relevant	Not relevant	serious [b]	none	163/1776 they developed celiac disease. OR for BF duration > 6 mo in 2 studies = 1-1.13 (95%CI wider = 0.6-2.3)				⊕⊕○○ LOW	CRITICAL
Development of **CD- observational studies (for every duration BF vs no BF) (evaluated by: n° of cases)**												
8 [3,4,5,6,7,8,9,10]	Observational studies	serious [c]	Not relevant	Not relevant	molto serious [b,c]	all plausible residual confounders could reduce the demonstrated effect	Remarkable heterogeneity (I2=89%), and mismatch of results between case-control studies and OR cohort studies for all observational studies = 0.69 (95% CI= 0.30-1.59)				⊕○○○ VERY LOW	CRITICAL

CI: Confidence interval; **OR:** Odds ratio

Explanations

a. RCT not designed for this outcome

b. 95% CI wide

c. Remarkable inconsistency of results between case-control studies and cohort studies

References

1. 2014, Lionetti. .
2. 2014, Vriezinga. .
3. 2007, Hummel. .
4. 2010, Decker. .
5. 1983, Auricchio. .
6. 2009, Roberts. .
7. 1988, Greco. .
8. 2010, Radlovich. .
9. 2005, D'Amico. .
10. 2005, Norris. .

Appendix 5. References

1. Szajewska, H., Shamir, R., Mearin, L et al. Gluten Introduction and the Risk of Coeliac Disease: A Position Paper by the European Society for Pediatric Gastroenterology, Hepatology, and Nutrition. J Pediatr Gastroenterol Nutr. 2016 Mar;62(3):507-13
2. Alvisi P, Brusa S, Alboresi S, et al. Recommendations on complementary feeding for healthy, full-term infants. Ital J Pediatr. 2015 Apr 28;41:36.
3. Bai JC, Ciacci C, Corazza GR, et al. World Gastroenterology Organisation Global Guidelines: Celiac Disease. https://www.worldgastroenterology.org/guidelines/global-guidelines/celiac-disease68
4. Fewtrell M, Bronsky J, Campoy C, et al. Complementary Feeding: A Position Paper by the European Society for Paediatric Gastroenterology, Hepatology, and Nutrition (ESPGHAN). Committee on Nutrition. J Pediatr Gastroenterol Nutr. 2017 Jan;64(1):119-132
5. Lebwohl B, Murray JA. Gluten Introduction, Breastfeeding, and Celiac Disease: Back to the Drawing Board. Statement Prepared by the Executive Council of the North American Society for the Study of Celiac Disease (NASSCD). Am J Gastroenterol. 2016 January; 111(1): 12–14.
6. Ribes Koninckxa C, Dalmau Serrab J, Moreno Villares JM, et al. The introduction of gluten into the infant diet. Expert group recommendation. An Pediatr (Barc). 2015;83(5):355.e1-355.e7
7. Romero-Velarde E, Villalpando-Carrión S, Pérez-Lizaur AB, et al. Consenso para las prácticas de alimentación complementaria en lactantes sanos. Bol Med Hosp Infant Mex. 2016;73(5):338---356
8. Turck D, Dupont C, Vidailhet M, et al. Diversification alimentaire : évolution des concepts et recommandations. Archives de Pédiatrie 2015;22:457-460
9. Henriksson C, Boström A-M, Wiklund IE. What effect does breastfeeding on coeliac disease? A systematic review update. Evid Based Med. 2013 Jun;18(3):98-103
10. Szajewska H, Systematic review with meta-analysis: early infant feeding and coeliac disease--update 2015. Aliment PharmacolTher. 2015 Jun,41(11):1038-54
11. Silano M, Agostoni C, Sanz Y, et al. Infant feeding and risk of developing celiac disease: a systematic review. BMJ Open. 2016 Jan 25;6(1):e009163.
12. Pinto-Sánchez MI, Verdu EF, Liu E, et al. Gluten Introduction to Infant Feeding and Risk of Celiac Disease: Systematic Review and Meta-Analysis. J Pediatr. 2016 Jan;168:132-43
13. EFSA Panel on Nutrition, Novel Foods and Foods Allergies (NDA), Castnmiller J, de Henauw S, Hirsch-Ernst KI et al. Appropriate age range for introduction of complementary feeding into an infant´s diet. EFSA Journal 2019; 17, (9):5780.
14. Chmielewska A, Szajewska H, Shamir R. Celiac Disease – Prevention Strategies through Early Infant Nutrition. World Rev Nutr Diet. Basel, Karger, 2013, vol 108, pp 91–97
15. Shamir R. The Benefits of Breast Feeding. In Bhatia J, Shamir R, Vandenplas Y (eds): Protein in Neonatal and Infant Nutrition: Recent Updates. Nestlé Nutr Inst Workshop Ser, vol 86, pp 67–76
16. Martín-Masot R, Diaz-Castro J, Moreno-Fernandez J, Navas-López VM, Nestares T. The Role of Early Programming and Early Nutrition on the Development and Progression of Celiac Disease: A Review. Nutrients. 2020 Nov 8;12(11):3427.
17. Simre K, Uibo O, Peet A, et al. Exploring the risk factors for differences in the cumulative incidence of coeliac disease in two neighboring countries: the prospective DIABIMMUNE study. Dig Liver Dis. 2016;48(11):1296-1301. doi:10.1016/j.dld.2016.06.029
18. Crespo Escobar, P., Castillejo, G., Martínez-Ojinaga, E, et al. (2018). Ten years of follow-up of the Spanish cohort of the European PreventCD study: the lessons learned. *Revista espanola de enfermedades digestivas : organo oficial de la Sociedad Espanola de Patologia Digestiva*, *110*(8), 493–499.
19. Hyytinen M, Savilahti E, Virtanen SM, et al.. Avoidance of Cow's Milk-Based Formula for At-Risk Infants Does Not Reduce Development of Celiac Disease: A Randomized Controlled Trial. Gastroenterology. 2017 Oct;153(4):961-970.e3.
20. Lionetti E, Castellaneta S, Francavilla R, et al. Mode of Delivery and Risk of Celiac Disease: Risk of Celiac Disease and Age at Gluten Introduction Cohort Study. J Pediatricians. 2017 May;184:81-86.e2.
21. Barroso M, Beth SA, Voortman T, et al. Dietary Patterns After the Weaning and Lactation Period Are Associated With Celiac Disease Autoimmunity in Children. Gastroenterology. 2018 Jun;154(8):2087-2096.e7.
22. Uusitalo U, Lee HS, Andrén Aronsson C, et al. Early Infant Diet and Islet Autoimmunity in the TEDDY Study. Diabetes Care. 2018 Mar;41(3):522-530.
23. Hummel S, Weiß A, Bonifacio E, et al. TEDDY Study Group. Associations of breastfeeding with childhood autoimmunity, allergies, and overweight: The Environmental Determinants of Diabetes in the Young (TEDDY) study. Am J Clin Nutr. 2021 Apr 8:nqab065.

24. Vajpayee S, Sharma SD, Gupta R, et al. Early Infant Feeding Practices May Influence the Onset of Symptomatic Celiac Disease. Pediatr Gastroenterol Hepatol Nutr. 2016 Dec;19(4):229-235.
25. Welander A, Montgomery S, Ludvigsson J, et al. Breast-feeding duration and gluten introduction among mothers with celiac disease. J Pediatr Gastroenterol Nutr. 2014 Jul;59(1):89-92.
26. Hård Af Segerstad EM, Lee HS, Andrén Aronsson C, et al. Daily Intake of Milk Powder and Risk of Celiac Disease in Early Childhood: A Nested Case-Control Study. Nutrients. 2018 Apr 28;10(5).
