# Supplementary material for: Recommendations on Complementary Feeding as a Tool for Prevention of Non-Communicable Diseases (NCDs)—Paper Co-Drafted by the SIPPS, FIMP, SIDOHaD, and SINUPE Joint Working Group
Source: Nutrients. 2022 Jan 7;14(2):257. doi: 10.3390/nu14020257 (PMC8778394; doi:10.3390/nu14020257)
Supplement: Supplementary file 1 [file nutrients-14-00257-s001.zip › nutrients-1513088-supplementary/Supplementary File S6.pdf]

APPENDIX 6

Recommendations 35-38

A6. Complementary Feeding (introduction of potentially allergenic foods) and Food allergy

Key questions

- Can the period of introduction of potentially allergenic foods affect the development of Food Allergy?

PICOs

a.
P In the healthy infant exclusively breastfed or formula-fed
I the introduction of potentially allergenic foods with the start of complementary nutrition
C compared to late introduction (≥12 months of age)
O does it carry a different risk of food allergies?

b.
P In the healthy infant exclusively breastfed or formula-fed
I the introduction of potentially allergenic foods before the start of complementary nutrition
C compared to the introduction with the start of complementary nutrition
O does it carry a different risk of food allergies?

Figure a6.1. GRADE ADOLOPMENT

FROM: *Journal of Clinical Epidemiology* 2017 81, 101-110 DOI: 10.1016/j.jclinepi.2016.09.

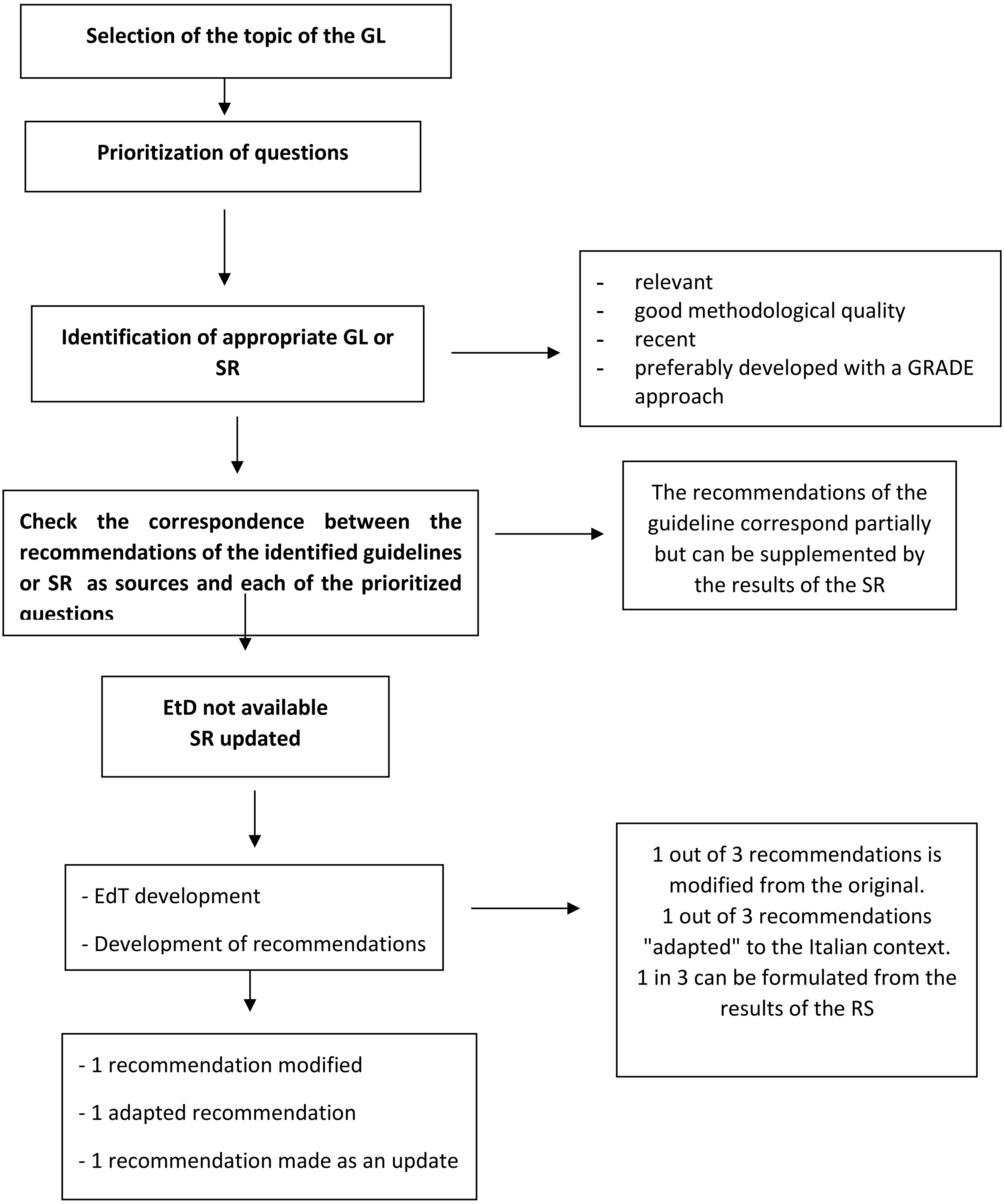

A6. METHODOLOGICAL ASSESSMENT

Table a6.1. Evaluation of guidelines and clinical documents

**GUIDELINES**	**Methodological evaluation AGREE II**		**Evaluators n = 3**		
**EAACI 2021 [1]**	**Score**		**Score**		**Score**
**DOMAIN 1. SCOPE AND PURPOSE**	**80%**	**DOMAIN 2. STAKEHOLDER INVOLVEMENT**	**61%**	**DOMAIN 3. RIGOUR OF DEVELOPMENT**	**76%**
1. The overall objective(s) of the guideline is (are) specifically described.	18	4. The guideline development group includes individuals from all the relevant professional groups.	16	7. Systematic methods were used to search for evidence.	18
2. The health question(s) covered by the guideline is (are) specifically described.	16	5. The views and preferences of the target population (patients, public, etc.) have been sought	10	8. The criteria for selecting evidence are clearly described.	18
3. The population (patients, public, etc.) to whom the guideline is meant to apply is specifically described. .	18	6. The target users of the guideline are clearly defined	16	9. The strengths and limitations of the body of evidence are clearly described.	15
				10. The methods for formulating the recommendations are clearly described.	17
				11. The health benefits, side effects, and risks have been considered in formulating the recommendations.	15
				12. There is an explicit link between the recommendations and the supporting evidence.	18
				13. The guideline has been externally reviewed by experts before its publication.	15
				14. A procedure for updating the guideline is provided.	17

**DOMAIN 4. CLARITY OF PRESENTATION**	**87%**	**DOMAIN 5. APPLICABILITY**	**74%**	**DOMAIN 6. EDITORIAL INDEPENDENCE**	**92%**

15. The recommendations are specific and unambiguous.	19	18. The guideline is supported with tools for application.	17	22. The views of the funding body have not influenced the content of the guideline	20
16. The different options for management of the condition are presented	17	19. The potential organizational barriers in applying the recommendations have been discussed.	17	23. Competing interests of guideline development group members have been recorded and addressed.	19
17. Key recommendations are easily identifiable<br>.	20	20. The potential cost implications of applying the recommendations have been considered.	17		
		21. The guideline presents key review criteria for monitoring and/ or audit purposes.	17		

**TOTAL SCORE**	**67%**
**Would you recommend the use of this LG?**	**YES**<br>Moderate methodological quality

Table a6.2. GLs e Documents excluded

Excluded GLs	Multidisciplinarity of the panel	Systematic search for evidence	Grading of recommendations	Reason for exclusion
AAAAI-CSACI 2021 [2]	NO	Based on SR 2016, not updated	YES	Intercompany Consensus<br>Low methodological quality
Fewtrellet al. 2017. ESPGHAN Complementary feeding [3]	NO	Declared but not published	NO	Low methodological quality
Romero-Velardea et al. 2016. Alimentation complementaria [4]	Limited to Pediatricians and Nutrition Experts	NO	NO	Low methodological quality
Canada's Dietary Guidelines 2018 [5]	Limited to Nutritionists and Public Health Experts	NO	NO	Low methodological quality<br>It does not contain relevant recommendations
Schwarzenberg et al. 2018. AAP Policy Statement [6]	NO	NO	NO	Low methodological quality
USDA 2015-2020 [7]	YES	YES	Related to the quality of the evidence	It does not contain relevant recommendations

Table a6.3. Appraisal of the Systematic Review

**AMSTAR 2**	De Silva et al. 2020 [8]
**1. Did the research questions and inclusion criteria for the review include the components of PICO? (Yes/No)**	YES
**2. Did the report of the review contain an explicit statement that the review methods were established before the conduct of the review and did the report justify any significant deviations from the protocol? (Yes/Partial Yes/No)**	YES
**3. Did the review authors explain their selection of the study designs for inclusion in the review? (Yes/No)**	YES
**4. Did the review authors use a comprehensive literature search strategy? (Yes/Partial Yes/No)**	YES
**5. Did the review authors perform study selection in duplicate? (Yes/No)**	YES
**6. Did the review authors perform data extraction in duplicate? (Yes/No)**	YES
**7. Did the review authors provide a list of excluded studies and justify the exclusions?** **(Yes/Partial Yes/No)**	NO
**8. Did the review authors describe the included studies in adequate detail?** **(Yes/Partial Yes/No)**	PARTIAL YES
**9. Did the review authors use a satisfactory technique for assessing the risk of bias (RoB) in individual studies that were included in the review?** **(Yes/Partial Yes/No/Includes only NRSI-RCT)**	YES

**10. Did the review authors report on the sources of funding for the studies included in the review? (Yes/No)**	NO
**11. If meta-analysis was performed did the review authors use appropriate methods for statistical combination of results? (Yes / No / No meta-analysis conducted)**	No Meta-Analysis conducted
**12. If meta-analysis was performed, did the review authors assess the potential impact of RoB in individual studies on the results of the meta-analysis or other evidence synthesis? (Yes / No / No meta-analysis conducted)**	No Meta-Analysis conducted
**13. Did the review authors account for RoB in individual studies when interpreting/ discussing the results of the review? (Yes/No)**	YES
**14. Did the review authors provide a satisfactory explanation for, and discussion of, any heterogeneity observed in the results of the review? (Yes/No)**	YES
**15. If they performed quantitative synthesis did the review authors carry out an adequate investigation of publication bias (small study bias) and discuss its likely impact on the results of the review? (Yes / No / No meta-analysis conducted)**	No Meta-Analysis conducted
**16. Did the review authors report any potential sources of conflict of interest, including any funding they received for conducting the review? (Yes/No)**	YES
**OVERALL EVALUATION**	**MODERATE QUALITY**
**presence of 1 critical item and 1 failed non-critical item (n.7, 10)**	

A6. RECOMMENDATIONS OF GLs, RESULTS IN SRs AND STUDIES

- Can the period of introduction of potentially allergenic foods affect the development of Food Allergy?	**a.**<br>**P** In the healthy infant exclusively breastfed or formula-fed<br>**I** the introduction of potentially allergenic foods with the start of complementary nutrition<br>**C** compared to late introduction (≥12 months of age)<br>**O** does it carry a different risk of food allergies?<br><br>**b.**<br>**P** In the healthy infant exclusively breastfed or formula-fed<br>**I** the introduction of potentially allergenic foods before the start of complementary nutrition<br>**C** compared to the introduction with the start of complementary nutrition<br>**O** does it carry a different risk of food allergies?

Table a6.4. Recommendations of the GLs EAACI 2021 [1]

3.1.2 | Introduction of chicken egg in the infant's diet.

The EAACI Task Force suggests introducing well-cooked chicken eggs, but not raw or uncooked pasteurized eggs, into the infant's diet as part of complementary feeding to prevent egg allergy in infants.

Reason for recommendation

The SR included two studies on the introduction of cooked egg and three on a raw or pasteurized egg, conducted on children at risk and not at risk of allergy. A further subgroup analysis from one of the cooked egg studies was published later. This evidence suggests that introducing small quantities of cooked, but not raw or pasteurized, eggs as part of the complementary diet probably reduces the risk of egg allergy.
The benefits of introducing the well-cooked egg likely outweigh the potential harm.
The Task Force does not support the early introduction of raw egg or raw pasteurized egg because the potential harms may outweigh the benefits. The studies reported adverse reactions, including anaphylaxis (11,12,20,21).

Strength of recommendation

This guideline supports the introduction of the well-cooked egg into the child's diet but is not a strong recommendation because the certainty of the evidence is moderate. There were only a small number of the cooked egg studies, their results were inconsistent, and there was only moderate to low certainty about the effect on egg allergy.
Evidence on raw egg or raw pasteurized egg was scarce. Available studies have had inconsistent results.

Practical implications

Healthcare professionals in countries where egg allergy is a problem could encourage families with normal or increased risk children to introduce about half a well-cooked small egg twice a week as part of complementary feeding from four to 6 mo of age. This is by the recent EFSA statement. This amount of egg is based on a study that showed that eating at least 2 grams of egg white protein per week prevents egg allergy. Another study successfully prevented egg allergy in smaller quantities [PETIT study]. Studies have used very cooked (10-15 minutes) boiled egg, but we consider that equivalent amounts of egg in well-cooked foods would also be appropriate.

3.1.3 | Introduce peanuts into the infant's diet

In populations with a high prevalence of peanut allergy, the EAACI Task Force suggests introducing peanuts at an appropriate age as part of complementary feeding to prevent peanut allergy in infants and young children.

Reason for recommendation

Our systematic review included three studies, one in infants at general risk level and two in infants at increased risk.
The SR found that in populations with a high prevalence of peanut allergy, introducing regular peanut consumption at 4 to 11 mo of age in infants at increased risk likely results in a large reduction in early childhood peanut allergy compared to peanut allergy completely avoiding peanuts for the first 5 years.
The benefits likely outweigh the potential harms.
Data from the studies included in the review and further observational studies suggest that it is safe to introduce age-appropriate forms of peanuts into the diet in the first year of life. Some adverse reactions, mostly mild, have been reported.

Strength of recommendation

This guideline supports the introduction of peanuts into the infant's diet in populations with a high prevalence of peanut allergy but is not a strong recommendation because the certainty of the evidence is moderate. The studies included in our review used different outcomes, population risk levels, and interventions, the certainty of the evidence about their effect on peanut allergy was moderate to low.
There was some inconsistency in the results. One study evaluated peanuts alongside five other foods. Two studies were conducted in very high-risk infants and compared to complete withdrawal from peanuts for 5 years, rather than compared to a more common exposure.
All studies were conducted in the UK. The generalizability of the results is therefore uncertain, and this led to a conditional recommendation.

Practical implications

In counties where peanut allergy is prevalent, health professionals could encourage families to introduce peanuts as part of their complementary diet. Professionals should support introducing peanuts in an age-appropriate form, simultaneously with breastfeeding.
It seems that the most effective age for introduction is 4 to 6 mo of life. Evidence of benefit is primarily for children at much-increased risk, but early introduction could also be encouraged in those with general risk levels because many cases of peanut allergy are seen in this lower risk group.
Peanuts should be introduced in an age-appropriate form to avoid any risk of choking or inhalation. For example, you could give children a heaping teaspoon of diluted peanut butter (2g of peanut protein) every week.
We suggest that peanuts should not be the first solid food to be introduced into the infant's diet.

The EAACI Task Force makes no recommendations for countries with a low prevalence of peanut allergy. In these countries, peanuts should be included in the diet according to normal eating habits and local recommendations.

Table a6.5. Characteristics and results of the most recent studies on the introduction of potentially allergenic foods

Study	Study design	Food(s)	Population and Sample size	Intervention and comparator	Outcome and follow up	Results	Conclusions
LEAP [9]	RCT	peanut	640 infants with severe atopic dermatitis and/or egg allergy and PTS for peanuts ≤ 4 mm (high-risk population)	Early introduction at 4-11 mo (median 7.8 mo) vs late introduction from 3 years	Prevalence of peanut allergy at 5 years	Not pre-sensitized to peanut<br>Prevalence of allergy between exposed and non-exposed (1.9% vs 13.7%, absolute difference in risk 11.8% (95% CI, 3.4-20.3; p <0.001)<br><br>Pre-sensitized to peanut<br>Prevalence of allergy between exposed and non-exposed (10.6% vs 35.3%, absolute difference in risk 24.7% (95% CI, 4.9-43.3; p <0.004)	Confirms that delayed exposure (> 12 mo) carries an increased risk of peanut allergy.<br>Subsequently, the follow-up study (Leap-on Study) (23) showed that the avoidance of peanut consumption for 12 mo, in children exposed to this food in the previous trial, did not lead to an increase in the prevalence of peanut allergy suggesting a long-term effect of early peanut exposure.
STAR [10]	RCT	egg	86 infants with moderate to severe eczema (high-risk population)	freeze-dried egg or placebo (powdered rice) from 4 to 8 mo of age	Prevalence of egg allergy at 1 year	Infants who have taken the egg<br>31% had an allergic reaction<br><br>At 12 mo, reduced prevalence of egg allergy in the treated group compared to placebo (33% vs 51%), statistically not significant	Concluded due to lack of funds without being able to reach the predetermined sample size.<br>The work confirmed, however, the increased risk of egg allergy in infants with moderate-severe atopic eczema and consequently the need to apply caution when introducing this food in these subjects.
STEP [11]	RCT	egg	820 infants between 4 and 6.5 mo of age, with atopic mother but without eczema (moderate-risk population)	Administration of powdered egg or placebo up to 10 mo of age	Prevalence of egg allergy at 1 year	Prevalence of egg allergy = 7% in the intervention group versus 10% in the control group (intention to treat analysis) (p = 0.20, relative risk [RR], 0.75; 95% CI, 0.48 at 1.17).	There is no evidence that regular egg intake from 4-6, 5 mo to 10 mo modifies the risk of egg allergy in the first year of life in children with atopic family history and without eczema; the low dose of egg administered (0.4 g of protein/day, equal to ½ egg/week) may not have been sufficient to induce tolerance.
HEAP [12]	RCT	egg	383 infants from the general population between 4 and 6 mo of age, not sensitized per egg (IgEs <0.35 KU)	Administration of powdered egg or placebo up to 12 mo of age	Prevalence of egg sensitization at 1 year (primary outcome)<br><br>Prevalence of egg allergy at 1 year (secondary outcome)	Prevalence of sensitization in the group that took the double egg but not statistically significant (p = 0.24, relative risk [RR], 2.20; 95% CI, 0.68-7.14)<br><br>Prevalence of egg allergy in newly sensitized patients = 2.1% in the egg-group vs 0.6% in the placebo group (p = 0.35, relative risk [RR], 3.30; 95% CI, 0, 35-31.32).	At 4-6 mo, 5.7% of patients were already sensitized to the egg and, as such, excluded from the study and 3.9% were already allergic to egg, 2/3 of them with anaphylactic reactions to DBPCFC. The authors, therefore, conclude that early exposure to the egg between 4-6 mo of life has not proved to be an effective or safe preventive strategy.
BEAT [13]	RCT	egg	319 infants at 4 mo of age, with atopic family history and SPT ≤ 2 mm per albumen	Egg powder or placebo up to 8 mo of age.	Prevalence of sensitization at 12 mo (primary outcome)	Lower percentage of sensitization for egg white (SPT ≥ 3 mm) at 12 mo in infants who introduced the egg at 4 mo (10.7%) compared to those who	No efficacy has been demonstrated on the clinically relevant outcome (documented allergy)

				After 8 mo, all study participants were encouraged to introduce cooked egg into the diet, except for those who had reacted to pasteurized egg powder or who had SPT for albumen ≥ 5 mm.	"Probable" egg allergy at 12 mo, i.e. a convincing history of allergic reaction to egg powder or introduction of egg into the diet after 8 mo and SPT ≥ 3 mm at 12 mo or reaction to egg challenge at 12 mo or SPT for albumen ≥ 5 mm.	introduced it after 8 mo (placebo group) (20 , 5%) (p = 0.03, odds ratio (OR), 0,46; 95% CI, 0,22-0,95)<br>No difference at 12 mo in the two groups for "probable" egg allergy	
PETIT [14]	RCT	egg	147 6-month-old infants with eczema<br><br>The recruitment was terminated early based on the preliminary analysis of the first 100 participants which shows a significant difference between the two groups	Administration of cooked egg powder or placebo up to 12 mo of life<br>From a very low dose: 25 mg of protein/day (equal to 0.2 g of the boiled whole egg for 15 minutes) from 6 to 9 mo and 125 mg of protein/day (equal to 1.1 g of boiled whole egg) from 9 to 12 mo.<br>Eczema was aggressively treated upon entry into the study and throughout the intervention period, with topical cortisone, to avoid exacerbations.	Prevalence of egg allergy at 1 year, determined by open challenge	First 100 participants<br>4/47 (9%) had egg allergy in the egg group vs 18/47 (38%) in the placebo group (risk ratio [RR] 0.222; 95% CI, 0.081-0.607, p = 0.0012).<br>The intention to treat analysis shows a significant reduction in egg allergy in the egg group (5/60 (8%) vs placebo group 23/61 (38%) (risk ratio [RR] 0.221 [0.090-0.543]; p = 0.0001) with preserved significance, in the subgroup analysis, only in that of patients pre-sensitized to enrollment (9% vs 43%, p = 0.001) (secondary prevention).	No acute reaction was described at the administration of the first dose of powder at 6 and 9 mo, demonstrating the safety of the approach.
EAT [15]	RCT	milk, egg, peanuts, sesame, fish, and wheat	1303 breastfed infants (general population)	Early exposure, 3-6 mo (median 19.6 weeks) (EIG - Early Introduction Group)<br><br>Late exposure at 6 mo completed (SIG)	Food allergy at 12 and 36 mo	Intention-to-treat analysis<br>Prevalence of food allergy to one or more of the six mentioned foods is not statistically different between EIG and SIG	The study is burdened by very low compliance with the intervention (<40%) and a protocol that does not allow the correct registration of all cases of allergy.<br>These factors limit the reliability of the results but also highlight the difficulty of starting weaning so early (before 4 mo).
PEAAD (not completed)	nRCT	peanut	460 infants between 5 and 30 mo of age, at high risk and with atopic dermatitis, to which o.<br>460 infanti tra i 5 e i 30 mesi di vita, ad alto rischio e con dermatite atopica, a cui è stata indicata o.	Peanut administration or free diet for the year following enrollment	Prevalence of peanut-mediated IgE allergy 12 mo after enrollment		
PreventADALL (not completed)	RCT	peanut, milk, wheat, and chicken egg	5200 infants among the general population	Open-label with 4 groups: observation, early introduction from 4 mo of age, skincare, combination of early introduction, and skincare.	Evaluate the impact on the development of a food allergy given by the introduction of 4 allergenic foods from 4 mo of age and/or the use of emollients up to 9 mo of age.		

A6. EVIDENCE PROFILE GRADE

Table a6.6. Early introduction (before 4-6 months) of most commonly allergenic foods

[introduction of more commonly allergenic foods early, before 4-6 months] compared to [introduction together with other complementary foods, at 6 months] for [Prevent allergy to the most common allergenic foods in children at risk and not at risk for food allergy]

Patient or population: [Prevent allergy to the most common allergenic foods in children at risk and not at risk for food allergy]

Setting: Outpatient

Intervention: [introduction of foods most commonly allergenic early, before 4-6 months]

Comparator: [introduction together with other complementary foods, at 6 months]

Certainty assessment							N of patient		Outcomes		Certainty	Importance
N of studies	Study design	Risk of distortion	Lack of reproducibility of results	Lack of generalizability	Imprecision	Further considerations	[introduction of most commonly allergenic foods early, before 4-6 months]	[introduction together with other complementary foods, at 6 months]	Relative (95% CI)	Absolute (95% CI)		
**Food allergy risk (follow up: mean 3 years; assessed with:% of AA at 12 and 36 months)**												
1 [1]	Randomized studies	Very serious [a,b]	Serious [c]	Not important	Not important	highly suspicious publication bias<br>all plausible residual confounders would suggest a spurious effect, while no effect was observed [a]	32/567 (5.6%)	42/595 (7.1%)	**RR 0.80** (0.51 at 1.25)	**14 - per 1.000** (from 35 - to 18 +)	⊕◯◯◯ VERY LOW	CRITIC

CI: Confidence interval; **RR:** Risk ratio

Explanations

a. Important detection bias: allergy cases between 3 and 6 average age in the treated group were not recorded

b. very low adherence to the protocol in the treated group (35%)

c. Unique study

References

1. Perkin MR, Logan K, Tseng A, et al. Randomized trial of intro¬duction of allergenic foods in breast-fed infants. Randomized trial of intro¬duction of allergenic foods in breast fed infants. . N Engl J Med 2016;374:1733-43.; N Engl J Med 2016;374:1733-43.

Table a6.7. Introduction raw or pasteurized egg together with other complementary foods before 9 months

[introduction of raw or pasteurized egg together with other complementary foods before 9 months] versus [delayed introduction after 9-12 months] for [Prevent egg allergy in children at risk and not at risk of food allergy]

Patient or population: [Prevent egg allergy in at-risk and non-food allergy-prone children]

Setting: Outpatient

Intervention:: [introduction of raw or pasteurized egg together with other complementary foods before 9 months]

Comparator: [delayed introduction after 9-12 months]

Certainty assessment							N of patient		Outcomes		Certainty	Importance
N of studies	Study design	Risk of distortion	Lack of reproducibility of results	Lack of generalizability	Imprecision	Further considerations	[introduction of raw or pasteurized egg together with other complementary foods before 9 months]	[delayed introduction after 9-12 months]	Relative (95% CI)	Absolute (95% CI)		
**Egg protein allergy (follow up: average 12 months; assessed with:% of children who develop egg protein allergy)**												
3 [1,2,3]	Randomized studies	Serious [a]	Very serious [b]	Not important [b]	Not important	all plausible residual confounders would suggest a spurious effect, while no effect was observed	43/555 (7.7%)	58/568 (10.2%)	**RR 0.66** (0.42 a 1.02)	**35 - per 1.000** (from 59 – to 2 +)	⊕⊕◯◯ LOW [c]	CRITIC

CI: Confidence interval; **RR:** Risk ratio

Explanations

a. 1 study (Bellach 2017) on the general population, 2 studies (Palmer 2013 and 2017) on patients at risk

b. conflicting results

c. Low certainty of the evidence, may not reduce

References

1. Palmer DJ, Metcalfe J,Makrides M ,et al.. Early regular egg exposure in infants with eczema: a randomized controlled trial. . J Allergy Clin Immunol 2013;132(2):387-392.; 2013.

2. Palmer DJ, Sullivan TR, Gold MS, et al.. Randomized controlled trial of early regular egg intake to prevent egg allergy. J. Allergy Clin Immunol 2017;139(5):1600-1607.; 2017.

3. Bellach J, Schwarz V,Ahrens B,et al.. Randomized placebo-controlled trial of hen's egg consumption for primary prevention in infants. . J Allergy Clin Immunol 2017;139(5):1591-1599.; 2017.

Table a6.8. Introduction of the cooked egg together with other complementary foods before 9 months

[introduction of the cooked egg together with other CAs before 9 months] versus [delayed introduction after 9-12 months] for [Preventing UFO in children at risk and not at risk of AA]

Patient or population: [Preventing APU in children at risk and not at risk for AA]

Setting: Outpatient

Intervention:: [introduction of egg cooked together with the other BC before 9 months]

Comparator:: [delayed introduction after 9-12 months]

Certainty assessment							N of patient		Outcomes		Certainty	Importance
N of studies	Study design	Risk of distortion	Lack of reproducibility of results	Lack of generalizability	Imprecision	Further considerations	[introduction of egg cooked together with the other BC before 9 months]	[delayed introduction after 9-12 months]	Relative (95% CI)	Absolute (95% CI)		
**Egg protein allergy (Early introduction of cooked egg (follow up: average 12 months; assessed with:% of cases)**												
1 [1]	Randomized studies	Serious [a]	Not important	Serious [b]	Not important	highly suspicious publication bias<br>all plausible residual confounders could reduce the demonstrated effect [a]	5/60 (8.3%)	23/61 (37.7%)	**RR 0.22**<br>(0.09 a 0.54)	**294 - per 1.000**<br>(from 343 - to 173 -)	⊕⊕◯◯<br>LOW	CRITIC

CI: Confidence interval; **RR:** Risk ratio

Explanations

a. recruitment interrupted before reaching the sample size

b. Unique study

References

1. Natsume O, Kabashima S,Nakasato J,et al.. Two-step egg in¬troduction for prevention of egg allergy in high-risk infants with eczema (PETIT): a randomized, double-blind, placebo-controlled trial. . Lancet 2017;389:276-86.; 2017.

Table a6.9. Introduction of peanuts together with other complementary foods before 9 months

[peanut introduction before 9 months] versus [delayed introduction after 12 months] for [Prevent peanut allergy in at-risk and non-food allergy-prone children]

Patient or population: [Prevent peanut allergy in children at risk and not at risk for food allergy]

Setting: Outpatient

Intervention: [introduction of peanuts before 9 months]

Comparator: [delayed introduction after 12 months]

Certainty assessment							N of patient		Outcomes		Certainty	Importance
N of studies	Study design	Risk of distortion	Lack of reproducibility of results	Lack of generalizability	Imprecision	Further considerations	[introduction of peanuts before 9 months]	[delayed introduction after 12 months]	Relative (95% CI)	Absolute (95% CI)		
**Peanut allergy - general population (follow up: mean 5 patient-years; assessed with:% of cases of peanut allergy)**												
1 [1]	Randomized studies	Not important	Serious [a]	Serious [b]	Not important	strong association	5/266 (1.9%)	36/263 (13.7%)	**RR 0.14** (0.05 a 0.34)	**118 - per 1.000** (from 130 - to 90 -)	⊕⊕⊕◯ MODERATE	CRITIC
**Peanut allergy - sensitized to peanut (follow up: average patient 5 years; assessed with:% peanut allergy at 5 years)**												
1 [1]	Randomized studies	Not important	Serious [c,d]	Not important	Serious [e]	strong association	5/41 (12.2%)	18/51 (35.3%)	**RR 0.35** (0.14 a 0.85)	**229 - per 1.000** (from 304 - to 53 -)	⊕⊕⊕◯ MODERATE	CRITIC

CI: Confidence interval; **RR:** Risk ratio

Explanations

a. confirmed only by the EAT study, of very low-quality

b. studies conducted only in the UK

c. unique study in at-risk, sensitized children

d. low sample size to make a strong recommendation

e. 95% broad CI

References

1. Du Toit G, Sayre PH,Roberts G,et al. N Engl J Med 2016, 374:1435-43., . Effect of avoidance on peanut allergy after early peanut consumption. . N Engl J Med 2016;3

Appendix 6. References

1. Halken S, Muraro A, de Silva D, et al.; European Academy of Allergy and Clinical Immunology Food Allergy and Anaphylaxis Guidelines Group. EAACI guideline: Preventing the development of food allergy in infants and young children (2020 update). Pediatr Allergy Immunol. 2021 Jul;32(5):843-858.
2. Fleischer DM, Chan ES, Venter C, et al. A Consensus Approach to the Primary Prevention of Food Allergy Through Nutrition: Guidance from the American Academy of Allergy, Asthma, and Immunology; American College of Allergy, Asthma, and Immunology; and the Canadian Society for Allergy and Clinical Immunology. J Allergy Clin Immunol Pract. 2021 Jan;9(1):22-43.e4.
3. Fewtrell M, Bronsky J, Campoy C, et al. Complementary Feeding: A Position Paper by the European Society for Paediatric Gastroenterology, Hepatology, and Nutrition (ESPGHAN). Committee on Nutrition. J Pediatr Gastroenterol Nutr. 2017 Jan;64(1):119-132
4. Romero-Velardea E, Villalpando-Carrión S, Pérez-Lizaur AB, et al. Consenso para las prácticas de alimentación complementaria en lactantes sanos. Bol Med Hosp Infant Mex. 2016;73(5):338---356
5. Canada's Dietary Guidelines for Health Professionals and Policy Makers. https://food-guide.canada.ca/static/assets/pdf/CDG-EN-2018.pdf
6. Schwarzenberg SJ, Georgieff MK, AAP Committee on Nutrition. Advocacy for Improving Nutrition in the First 1000 Days To Support Childhood Development and Adult Health. Pediatrics. 2018;141(2):e20173716
7. U.S. Department of Health and Human Services and U.S. Department of Agriculture. 2015–2020 Dietary Guidelines for Americans. 8th Edition. December 2015. Available at http://health.gov/dietaryguidelines/2015/guidelines/.
8. de Silva, D., Halken, S., Singh, C., et al. … European Academy of Allergy, Clinical Immunology Food Allergy, Anaphylaxis Guidelines Group (2020). Preventing food allergy in infancy and childhood: Systematic review of randomized controlled trials. *Pediatric allergy and immunology: official publication of the European Society of Pediatric Allergy and Immunology*, *31*(7), 813–826. https://doi.org/10.1111/pai.13273
9. Du Toit G, Graham R, Sayre P, et al. Randomized trial of peanut consumption in infants at risk for peanut allergy. N Engl J Med 2015;372:803-13.
10. Palmer DJ, Metcalfe J, Makrides M, et al. Early regular egg exposure in infants with eczema: A randomized controlled trial. *J Allergy Clin Immunol* 2013; 132(2):387-392.
11. Palmer DJ, Sullivan TR, Gold MS, et al. Randomized controlled trial of early regular egg intake to prevent egg allergy. J Allergy Clin Immunol 2017;139:1600-07
12. Bellach J, Schwarz V, Ahrens B, et al. Randomized placebo-controlled trial of hen's egg consumption for primary prevention in infants. J Allergy Clin Immunol 2017;139:1591-9.
13. Wei-Liang Tan J, Valerio C, Barnes EH, et al. A randomized trial of egg introduction from 4 months of age in infants at risk for egg allergy. J Allergy Clin Immunol 2017;139:1621-8.
14. Natsume O, Kabashima S, Nakasato J, et al. Two-step egg introduction for prevention of egg allergy in high-risk infants with eczema (PETIT): a randomized, double-blind, placebo-controlled trial. Lancet 2017;389:276-86.
15. Perkin MR, Logan K, Tseng A, et al. Randomized trial of introduction of allergenic foods in breast fed infants. N Engl J Med 2016;374:1733-43.
